# Chiral Co_3_Y Propeller-Shaped Chemosensory Platforms Based on ^19^F-NMR

**DOI:** 10.1021/acs.inorgchem.2c03737

**Published:** 2023-01-30

**Authors:** Gabrielle Audsley, Harry Carpenter, Nsikak B. Essien, James Lai-Morrice, Youssra Al-Hilaly, Louise C. Serpell, Geoffrey R. Akien, Graham J. Tizzard, Simon J. Coles, Cristina Pubill Ulldemolins, George E. Kostakis

**Affiliations:** † Department of Chemistry, School of Life Sciences, University of Sussex, Brighton BN1 9QJ, UK; ‡ Sussex Neuroscience, School of Life Sciences, University of Sussex, Brighton BN1 9QG, UK; § Department of Chemistry, Lancaster University, Lancaster LA1 4YB, UK; ∥ UK National Crystallography Service, Chemistry, University of Southampton, Southampton SO1 71BJ, UK; ⊥ Chemistry Department, College of Science, Mustansiriyah University, Baghdad 10001, Iraq

## Abstract

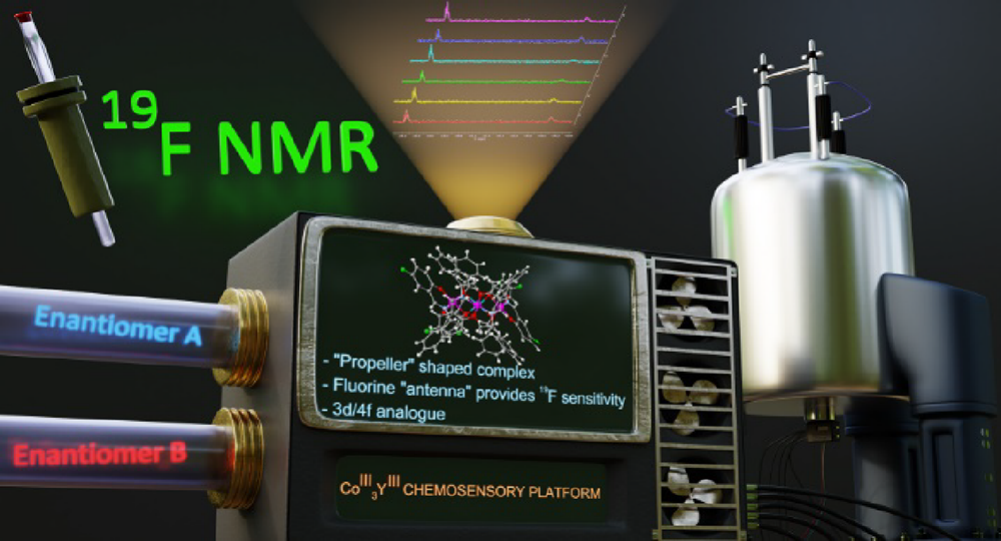

Two propeller-shaped chiral Co^III^_3_Y^III^ complexes built from fluorinated ligands are synthesized and characterized by single-crystal X-ray diffraction (SXRD), IR, UV–vis, circular dichroism (CD), elemental analysis, thermogravimetric analysis (TGA), electron spray ionization mass spectroscopy (ESI-MS), and NMR (^1^H, ^13^C, and ^19^F). This work explores the sensing and discrimination abilities of these complexes, thus providing an innovative sensing method using a ^19^F NMR chemosensory system and opening new directions in 3d/4f chemistry. Control experiments and theoretical studies shed light on the sensing mechanism, while the scope and limitations of this method are discussed and presented.

## Abstract 2

This work explores the sensing and discrimination abilities of propeller-shaped chiral Co^III^_3_Y^III^ complexes built from fluorinated ligands, thus providing an innovative sensing method using a ^19^F NMR chemosensory system and opening new directions in 3d/4f chemistry. Control experiments and theoretical studies shed light on the sensing mechanism, while the scope and limitations of this method are discussed and presented.

## Introduction

The detection and differentiation of chiral enantiomers are important in synthetic and pharmaceutical chemistry, but they can prove challenging.^1−6^ Two enantiomers may possess different chemical and physical properties; thus, misidentifying their configuration can jeopardize biological and pharmacological activities. For example, the stereochemistry of drugs can significantly affect their activity due to the inherent chirality found in the environments of biological systems. New biologically active chiral compounds are ever-increasing, where approximately 60% of all pharmaceutical drugs are chiral. Developments into asymmetric synthesis routes have stemmed from this increase in the production of chiral compounds; however, a complete stereochemical analysis including the absolute configuration, enantiomeric excess (*ee*), and total concentration has limited the discovery process.

Conventional high-performance liquid chromatography (HPLC) separates the enantiomers, which are subsequently stereochemically analyzed. The HPLC technique involves a chiral column packed with a chiral stationary phase where enantiomers can be separated. Chiral additives can also be added to the mobile phase to separate enantiomers or form diastereomers beforehand; however, the extensive cost of these chiral columns is a limiting factor for scientific developments. Therefore, the discovery of other ease methods is in need.

The stereochemical discrimination can occur using spectroscopic methods, including circular dichroism (CD) and fluorescence,^7−12^ by monitoring absorbance intensity change and NMR, presenting chemically shifted signals for different chiral molecules or complexes.^13−16^ For the latter, a host–guest complex consisting of a chiral substrate sample interacts with a chiral detector molecule, transferring chiral information and inducing a change in the chiral environment, observed as split signals of precise chemical shifts in the corresponding NMR spectrum. NMR chemosensors for chiral discrimination are typically limited to aromatic-based compounds, facilitating signal splitting by inducing a significant shielding effect. ^1^H NMR dominates the differentiation of organic or biologically relevant molecules, but an increase in the size of these molecules and structural complexity leads to overlapping resonances on an already narrow spectral range, thus making discrimination impossible. Known ^1^H NMR analytical methods for chiral determination include the addition of chiral solvating agents (CSAs),^17−19^ chiral lanthanide shift reagents (CLSRs),^20^ and chiral derivatizing agents (CDAs).^21^ For example, Pirkle’s alcohol^22^ determines the absolute configuration and enantiomeric purity of chiral molecules. Although Pirkle’s alcohol is still regularly used to analyze chiral molecules, issues have arisen related to the inaccurate determination of *ee* due to resonance overlap present in the ^1^H NMR spectra—a result of a narrow spectral width accompanied by the complicated spectra that arise with large organic compounds.

**Scheme 1:**
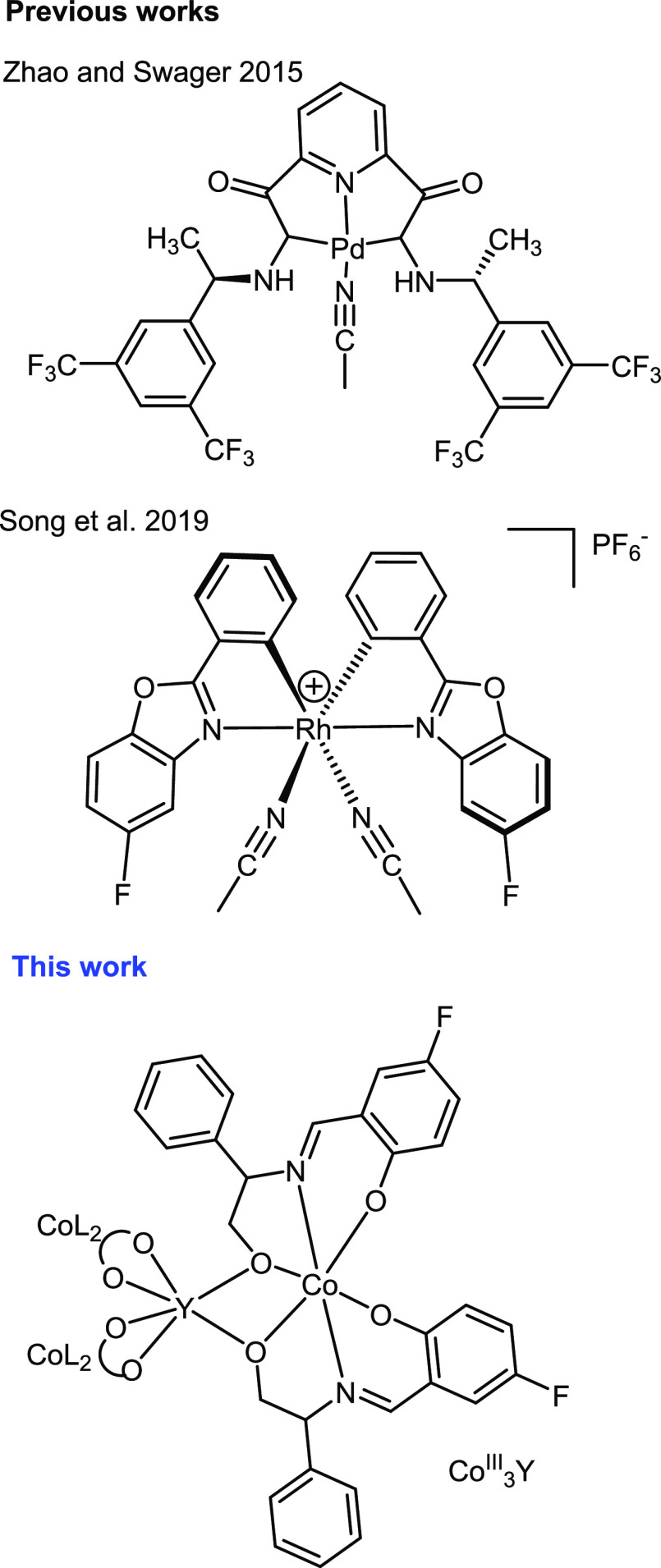
Previous and Current Complexes Used for Amine Sensing with ^19^F NMR

Hence, the development of heteronuclear-based sensors represents a reasonable alternative to overcome these hindrances. Methods incorporating molecules that bear phosphorous atoms are developed to monitor changes with ^31^P{^1^H} NMR due to observable splitting of signals and a broad spectral range compared to ^1^H NMR spectra.^15,23^ Moreover, the ^19^F nucleus of spin quantum number 1/2 has 83% the sensitivity of the ^1^H nucleus and is found in 100% natural abundance; with great receptivity, it yields strong signals on an NMR spectrum.^24−31^^19^F can also be easily incorporated into organic compounds as it mimics the ^1^H nucleus in many environments and benefits from a lack of background interference due to its low natural occurrence;^32,33^ thus, it can be used to probe the structure and dynamics of many large and complex biomolecules such as proteins.^34^ The broad detection window of ^19^F NMR is conducive to easily distinguishable split signals and narrow overlapping resonances, increasing spectral resolution and providing the simpler deconvolution of complicated spectra. In 2015, Zhao and Swager^35^ presented a ^19^F chemosensory method to detect multiple chiral amines. The sensing strategy consisted of a palladium complex built from a chiral pincer ligand, of which the scaffold is facile in preparation alongside having well-known coordination chemistry (Scheme 1, upper).^36,37^ When varying the substituent R groups of the ligand, the authors envisioned an optimized design by moving the fluorine atoms closer to the analyte, confining the chiral pocket. This change successfully demonstrated more pronounced differences in the chemical shifts of the analytes, allowing for more time-efficient detection with improved resolution. In addition to the assignment of absolute configuration, integrating these ^19^F NMR spectra gave values in agreement with the actual enantiopurities of the chosen analytes. Following Zhao and Swager’s work, Song et al. designed a chiral sensor based on octahedral rhodium complexes containing fluorine (Scheme 1, middle).^38^ The advantage of this complex is its two-coordinating site, which permits the sensing of monoamines, diamines, and amino acids. The scaffold was previously used as a successful chiral catalyst in asymmetric catalytic reactions.^39,40^ Screening tests identified DMSO-d_6_ as the optimum solvent, presenting a broader chemical shift difference of 0.21 ppm..

**Scheme 2:**
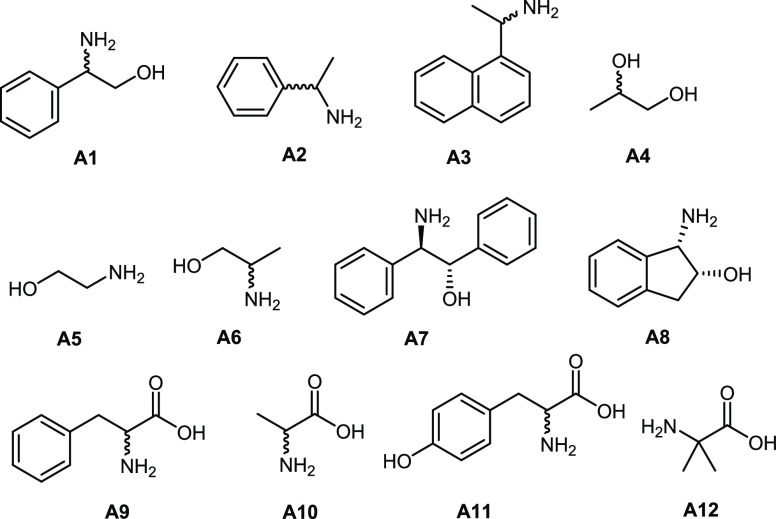
The Type of Analytes Tested in This Work

Complexes formed from organic ligands, transition metal (3d) ions, and/or lanthanide (4f) ions constitute a large class of materials esteemed for their wide range of properties, including luminescence,^41−43^ magnetism,^44−47^ and catalysis.^48−55^ Synthetic-wise, in 3d–4f chemistry, the 3d or 4f metal ions can be targeted and selectively substituted by 3d or 4f ions with similar coordination properties, often *without altering the topology of the core*. This synthetic alteration has been found in numerous 4f–4f ion and 3d–3d ion substitutions^56−60^ but is proven difficult for the 4f–4f ion exchange due to the lanthanide contraction. The simultaneous presence of 3d and 4f elements in a single molecule represents an elegant advantage for studying mechanisms and rationalizing experimental data. For example, Y^III^ can be included in this category because it has a size and Lewis acidity similar to Ho^III^ and its diamagnetic character permits monitoring with NMR (^1^H, ^13^C, ^15^N, ^19^F, or ^89^Y). It may also be possible to exchange 3d ions in the same oxidation state without distorting the core topology and permitting monitoring with UV–vis, EPR, or NMR. Similarly, the 4f entity may be replaced with Gd^III^ (^8^S_7/2_ ground state) so that electron paramagnetic resonance can identify changes in the coordination environment within the complex; this is a process that we have successfully demonstrated in the past.^48,51^

Recently, we identified the Lewis acidic catalytic efficacy of two chiral propeller-shaped [Co^III^_3_Y^III^L_6_] complexes built from a known Schiff base ligand; other groups investigated the magnetic properties of these components.^61−67^ These two compounds retain their structure and chirality in solvent media for a prolonged period, over 6 months. The Y metal sits in the center with the Co^III^ centers and associated pairs of organic ligands forming the propeller wings. Each Co^III^ center sits in the center of an almost perfect octahedron, while the central Y^III^ center bonds to six O atoms in a trigonal antiprismatic geometry. Given (a) the high ionic radii of 4f ions, thus being difficult to control their ligand exchange with solvent substrates, (b) that amines are known to coordinate to lanthanide metal centers in organic media,^68,69^ (c) that the coordination geometry of the Co centers is fulfilled, and (d) that the 4f ion is captured in a trigonal antiprism, which limits access to solvent-substrate molecules only via axial sites, thus replicating the coordination sphere of the non-labile Pd and Rh examples,^35,38^ we envisioned these propeller-shaped molecules as an ideal platform for limited ligand exchange with the solvent-lattice systems. Moreover, the development of ^19^F-NMR chemosensors relies on modifying already well-understood species; thus, we embarked on a project modifying the ligand used in our previous studies^67^ by adding a fluorine antenna on the organic scaffold to assemble a 3d–4f entity that can serve as a chiral ^19^F-NMR chemosensory platform. ^19^F NMR can then be used to monitor the sensing ability of the system, boasting a broad spectral range and avoidance of complicated and overlapping resonances, the key to efficiency, simplicity, and rapidity with a chiral detection method. The scope and limitations of our approach are discussed herein.

## Results and Discussion

### Ligand and Complex Synthesis

The ligands (H_2_L^R^, H_2_L^S^ and its racemic version H_2_L, see Table S1) can be synthesized in one high-yielding condensation reaction from commercial fluorinated salicylaldehyde and the corresponding amino alcohol (see the ESI). These ligands have never been used in coordination chemistry to our knowledge. Following our synthetic protocol, the assembly of the ligands with nitrate or chloride Co^II^/Y^III^ salts in CH_3_CN or EtOH solvent media and the presence of triethylamine would harvest the tetranuclear propeller-shaped entity; however, screening tests (Table S1) were carried out to optimize the synthetic procedure and obtain the targeted complexes in high purity. Both protic and aprotic, polar, and non-polar solvents were screened as the ligand can form a hydrogen bond, and it was unclear whether or not this would benefit or disadvantage the crystallization process. X-ray-quality crystals were only grown in EtOH, MeOH, and MeCN. Preliminary crystallographic characterization studies (see the ESI) identified that EtOH was not to be the solvent of choice; an additional non-coordinating ligand can be found in the lattice along with the expected tetranuclear targeted species{[Co^III^_3_Y^III^L_6_][H_2_L]·*x*(EtOH)}. MeOH and MeCN produced single crystals of [Co^III^_3_Y^III^L_6_]·*x*(MeOH) and [Co^III^_3_Y^III^L_6_]·*x*(MeCN), respectively, but comparatively, MeCN produced a higher yield and higher-purity crystals and additionally presented the same crystal product for both *S* and *R* enantiomers when using the chloride salts of both yttrium and cobalt sources. Then, the synthesis was repeated with nitrate salts of yttrium and cobalt instead of the chloride, and these reactions gave the highest yields. Last but not least, reactions at higher concentrations proved to be less efficient.

### Single X-ray Diffraction Characterization

**Figure 1:**
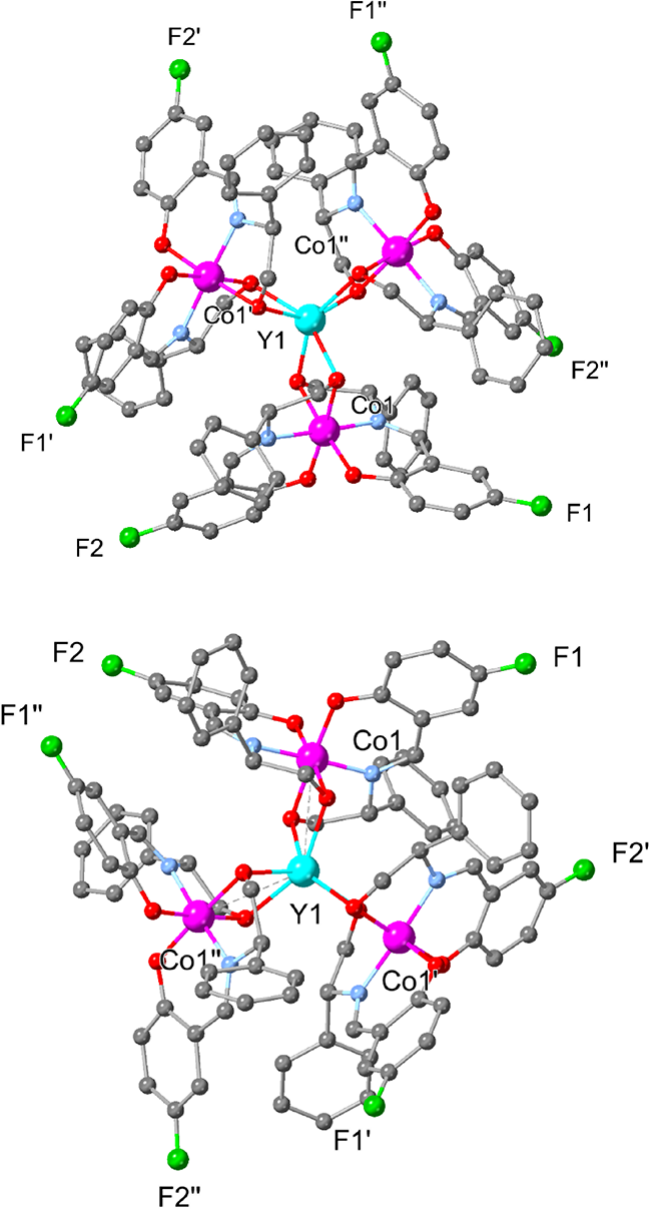
X-ray structures of **C^R^** (upper) and **C^S^** (lower). Lattice (CH_3_CN) molecules and H atoms are omitted for clarity. Color code: Y (light blue), Co (pink), C (gray), N (blue), O (red), F (green).

Both compounds **C^R^** and **C^S^** crystallized from acetonitrile were characterized at the NCS UK facility. Both crystals diffract poorly at higher angles; therefore, an exact allocation of the lattice solvent molecules proves challenging, but a co-crystallized ligand, as was the case in the EtOH samples, cannot be identified. Both compounds are isostructural and possess a general formula [Co^III^_3_Y^III^L_6_]·*x*(MeCN), but the lattice acetonitrile molecules slightly deviate (*x* is 5.5 for ^R^ (**C^R^**) and 5.25 for S (**C^S^**), respectively). The relevant crystallographic tables, including crystal data, structure refinement, and bond lengths, are found in the Supplementary Material. Both compounds provide the targeted propeller-shaped structure in which the Y^III^ center sits in the middle of a trigonal antiprism (Figure 1). The propeller itself is chiral, and this motif has been seen in other tetranuclear complexes and molecular compounds.^66,70−78^ Notably, in both compounds, the Flack parameter, an indication of chirality retention,^79,80^ deviates from the typical values. This notion indicates that the racemization process may occur during crystallization (13 and 21% for **C^R^** and **C^S^**, respectively), as the crystals are formed and collected after 1 week. This racemization process may be an outcome of the ligands or the propeller shape,^73^ reversing their chirality. We collected better-quality data for the **C^R^** derivative using a Cu source to validate this notion further (Table S3). The analysis provides a Flack parameter of 7%, which is very close to 5% of the enantiomerically pure Fe_4_ compound,^75^ indicating that the enantiomeric purity of both **C^R^** and **C^S^** is of good levels. Given that in both cases (**C^R^** and **C^S^**), racemization is unavoidable under the current synthetic circumstances and the results presented in the following sections, we did not attempt to validate the Flack parameter of **C^S^**.

### NMR Characterization

As **C^S^** and **C^R^** are diamagnetic, retrieving ^1^H NMR without complications was possible. Comparing the ^1^H NMR spectra of both *S*- and *R*-derivatives of H_2_**L** and **C**, there is a clear upfield shift of the imine protons on the transformation of ligand to complex (Figure S2), consistent with a lesser deshielding effect present from the nitrogen now coordinating to cobalt with electronegativity drawn further away from the imine carbon. The C–H proton on the adjacent C to the N has shifted downfield, indicating an increase in shielding due to cobalt coordination. Additionally, the OH peak is lost due to ligand-to-metal coordination.^7^

The ^19^F NMR spectra (Figure S3) of both *S*- and *R*-derivatives of H_2_**L** and **C** also demonstrated a change in the chemical shift of the resultant resonance, confirming the formation of a new species. Notably, both the ^1^H and ^19^F NMR spectra of each pair of derivatives overlay, thus demonstrating an identical chemical shift pattern.

### CD

**Figure 2:**
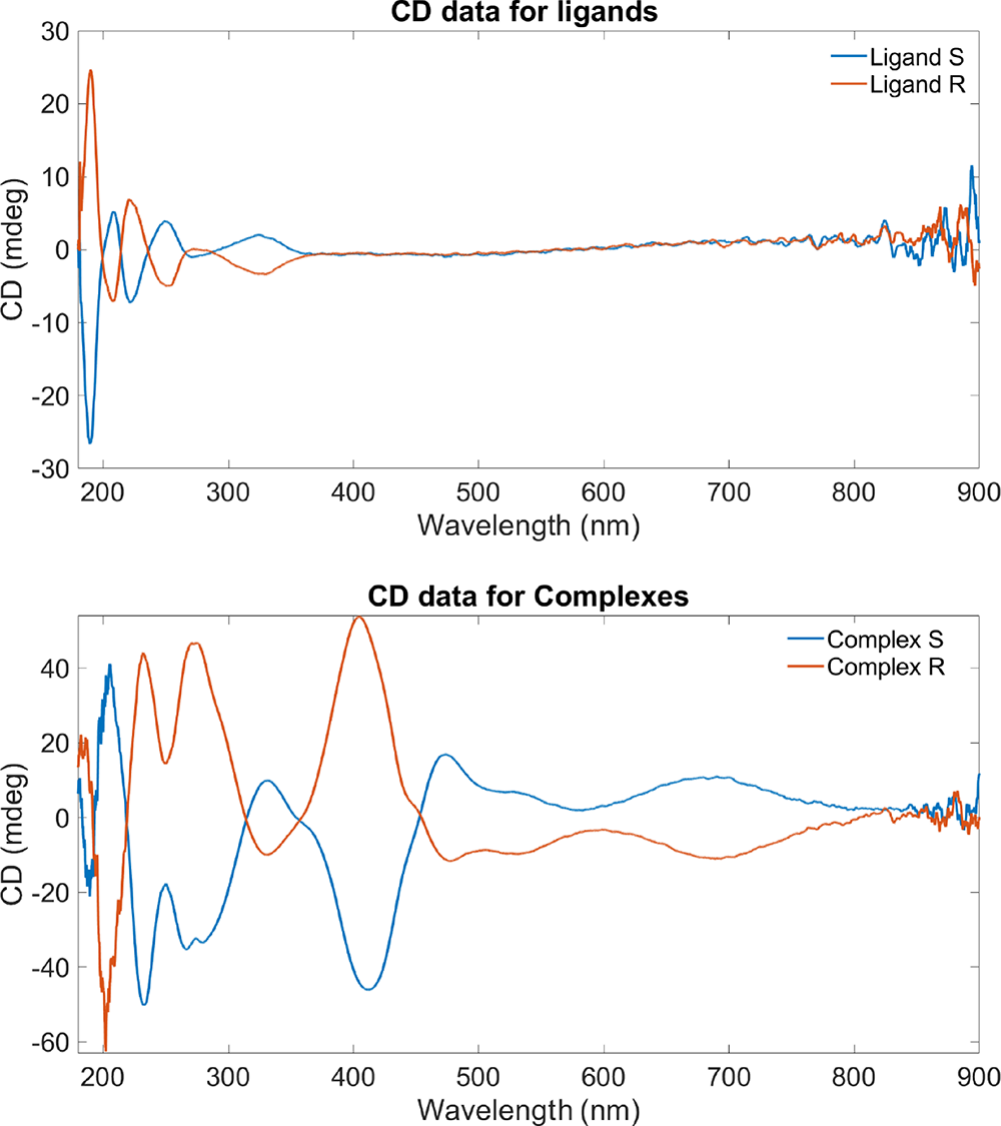
Circular dichroism spectra of H_2_**L^S^** versus H_2_**L^R^** (upper); **C^S^** versus **C^R^** (lower).

CD is a light absorption spectroscopy that “quantifies” chirality by measuring the differential absorption of optically active molecules in left- and right-handed circularly polarized light; thus, it is possible to investigate the structural features of optically active chiral molecules. CD measurements of enantiomers should be inequivalent but opposite. The spectra of both pairs of H_2_L and **C** in solution, acetonitrile 1 mM, confirmed active optical species, demonstrating opposite rotation (Figure 2). However, minor differences in the spectra (i.e., minima and maxima at 285, 310, 410 nm for **C^S^**) may be attributed to the racemization process of the propeller or the ligands, evidenced by the Flack parameter (see above).

### Thermogravimetric Analysis

We conducted a thermogravimetric analysis (under a N_2_ atmosphere) of both compounds. Both complexes are thermally stable up to 220 °C (Figure S4). The initial weight loss (∼8%, 100 °C) corresponds to lattice CH_3_CN and possibly absorbed H_2_O molecules. Above 220 °C, a three-step decomposition is taking place. Taking into account the molecular formula of the crystal structure {**C^S^** 5.33 (CH_3_CN)} and that the anticipated theoretical value of [Co^III^_3_Y^III^O_6_] is 18.72%, we envisage that decomposition completes possibly beyond 1000 °C.

### Titration Studies

The exploration of new ^19^F NMR chemosensory systems relies on repurposing already known systems;^35,38^ thus, our initial aim was to introduce a broad scope of chiral analytes used in previously known sensing procedures^35,38^ and assess the sensing ability of **C^S^** and **C^R^**. To reiterate how the ^19^F NMR chemosensor works, introducing an analyte to the sensing system induces a change in the environment of the complex, forming a new complex {**C^S^** + A}, which will be subsequently recognized as a new, second signal in the ^19^F NMR spectra. The type of sensor–analyte interaction, covalent bond, hydrogen bond, or aromatic interactions is unclear until further evidence is obtained. From previous works, amines are known to coordinate to lanthanide metal centers^68^ as well as the recent chiral sensing studies.^15,35,38^ It is suggestive that the amines will coordinate with the Y^III^ center; however, due to the nature of the ligand with O and NH moieties, hydrogen bonding interactions between the complex and the analyte cannot be excluded.

We used a range of chiral and non-chiral analytes. Preliminary titration tests were performed to standardize the process. Stock solutions of both enantiomers of the analytes were prepared in the following concentrations: 1, 2, 10, and 20 mM, following known protocols.^35,38^ Each concentration was trialed to decipher optimum reacting conditions—looking for intensity of signals and the chemical shift difference between enantiomeric pairs in the corresponding ^19^F NMR spectra. 1 mM stock solutions of both **C^S^** and **C^R^** were prepared in 10 mL CDCl_3_, and 350 μL of **A1** with each complex derivative (in all possible combinations) was then added directly to an NMR tube and recorded instantaneously.

Preliminary testing began with investigating the stability of **C^S^** and **C^R^** in both CDCl_3_ and CD_3_OD, looking for any degradation or change in the spectrum over some time (Tables S5 and S6). Both solvents had been favored in previous works^35,38^ with their use dependent on the dissolving analyte—whether they required a protic or an aprotic environment. As demonstrated in Tables S5 and S6, there was no change in the ^19^F NMR spectra of either complex; however, a slight shift of the principal peak −130.06 ppm (CDCl_3_) over −131.22 ppm (CD_3_OD) is observed. Notably, ^19^F NMR spectra recorded after 1 week or month proved complex stability, representing a significant advantage to this sensing method; thus, stock solutions can be prepared and stored. In CD_3_OD, a coordinating solvent, an additional peak was found at approximately −139 ppm, which may correspond to {**C**-(CD_3_OD)_*x*_}, where *x* = 1 or 2 species.

We then used **C^S^** to detect **A1** in 64 scans, which requires approximately 2 min of running time in CDCl_3_ and at 30 °C (Table 1). Initial efforts of 1:1 and 1:2 ratios, as seen to be most successful in previous studies,^35,38^ did not present a split signal; therefore, increasing the amine loading was necessary. In fact, this was found to be an advantage as an analyte signal was achievable using less complex. Both the 1:10 and 1:20 ratios demonstrated amine peaks, but the chemical shift difference between enantiomeric pairs for 1:20 was broader, allowing for more direct discrimination and boasting the advantage of requiring less complex. Therefore, we chose to use the ratio 1:20 for further experimental studies. Then, we examined if additional scans (128 vs 64) shall improve the sensing performance and incorporate compound **C^R^** (Table S6). A 20 mM concentration of **A1** (in all possible combinations) in CDCl_3_ at 30 °C with **C^S^** and **C^R^** was run for 128 scans (Table S7). **C^S^** allowed for better chiral discrimination with a broader enantiomeric difference in chemical shift compared to **C^R^**. However, as the chemical shift differences were not too dissimilar, 64 scans were deemed more efficient at half the running time. Repeats were then undertaken using **A1**, but changing the solvent system from CDCl_3_ to CD_3_OD; however, no analyte response can be detected at *T* = 0 (Table S8). An analyte peak appeared at *T* = 96 h, suggesting that CD_3_OD can be used for the detection studies but not with an immediate effect.

**Table 1:**
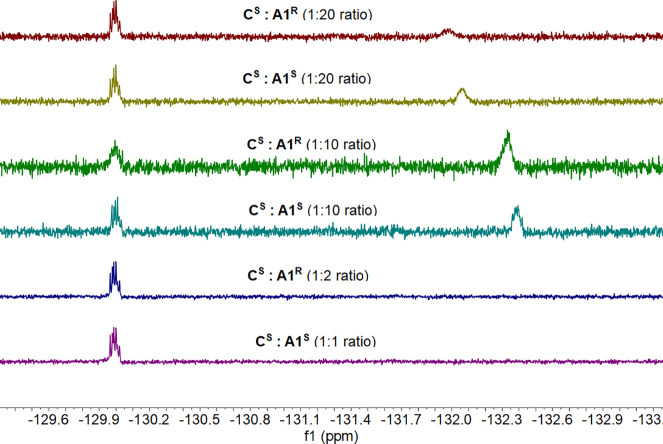
^19^F NMR Spectra Using a Different Sensor (1 mM, CDCl_3_): Amine Ratios (1:1, 1:2, 1:10, 1:20, CDCl_3_) (*T* = 303 K)

After discovering that the sensor can work at large amine-to-complex ratios, the ratio was increased from 1:20 to 1:50 to explore how far this factor may be extended (Table S9). The resulting ^19^F NMR spectra confirmed the sensing of the **A1** analyte. However, the signals were slightly less prominent than with the 1:20 ratio; the chemical shift differences improved by approximately 0.1 ppm in comparison. These enantiomeric differences were better than or comparable to previous works of chiral sensing using ^19^F sensors,^35,38^ thus making this complex a competitor against current well-defined metal complex sensors. This notion may suggest that a sensor:amine ratio of 1:50 allows for more rapid discrimination of further analytes.

### Analyte Scope and Limitations

After achieving successful results for the sensing of **A1**, further analytes were introduced (Scheme 2). No additional analyte peak was present for compounds **A2**, **A3** (monoamines), and **A4** (diol). Then, we incorporated other amino alcohols (**A5–A8**) and amino acids (**A9**–**A12**) with the same 1,2-amino alcohol backbone compared to **A1**. For the amino acids, we used CD_3_OD instead of CDCl_3_ and NaOMe for dissolving purposes, as demonstrated in previous studies.^38^ Amino alcohols **A5**–**A7** were successfully sensed but required different ratios (1:100, 1:50, and 1:20, respectively, Table 2), as in some instances, the amine signal was not intense enough to account for a resolved signal.

**Table 2:**
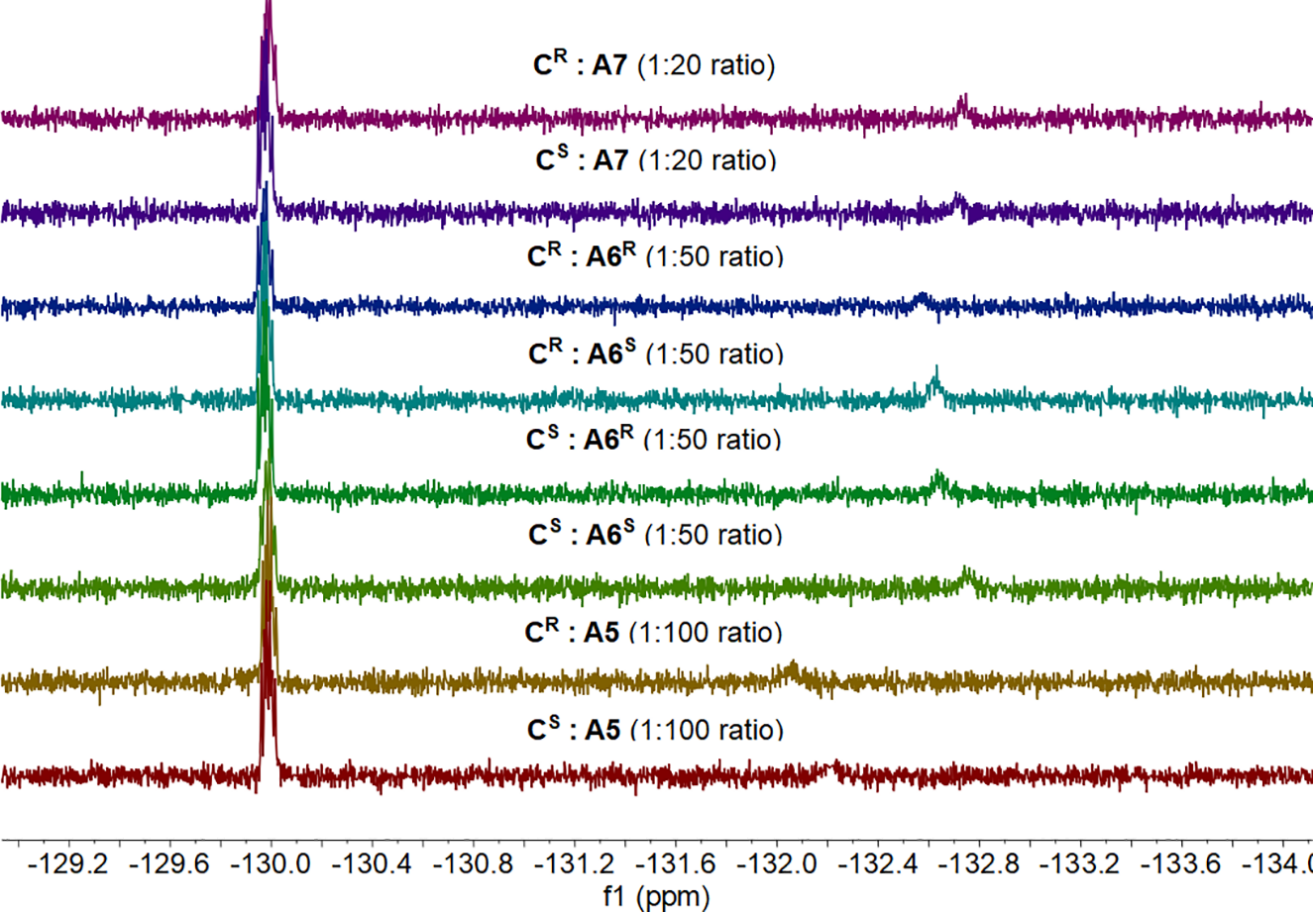
^19^F NMR Spectra of Complex (1 mM, CDCl_3_) and different Amino Alcohols (xx mM, CDCl_3_, *T* = 303 K)^a^

complex	analyte	ratio	time (h)	complex peak	complex + analyte peak	difference
**C^S^**	**A5**	1:100	0	–129.98	–132.22	
**C^R^**	**A5**	1:100	0	–129.98	–132.06	
**C^S^**	**A6^S^**	1:50	0	–129.98	**–132.72**	**0.10**
**C^S^**	**A6^R^**	1:50	0	–129.98	**–132.62**	
**C^R^**	**A6^S^**	1:50	0	–129.98	**–132.65**	**0.09**
**C^R^**	**A6^R^**	1:50	0	–129.98	**–132.56**	
**C^S^**	**A7**	1:20	0	–129.98	–132.70	
**C^R^**	**A7**	1:20	0	–129.98	–132.72	
**C^R^**	**A8**	1:20	0	–129.98	no peak	
**C^S^**	**A8**	1:20	0	–129.98	no peak	

a The corresponding ^19^F NMR spectra are given in the order of the table. Data for **A8** are omitted for clarity.

**Scheme 3:**
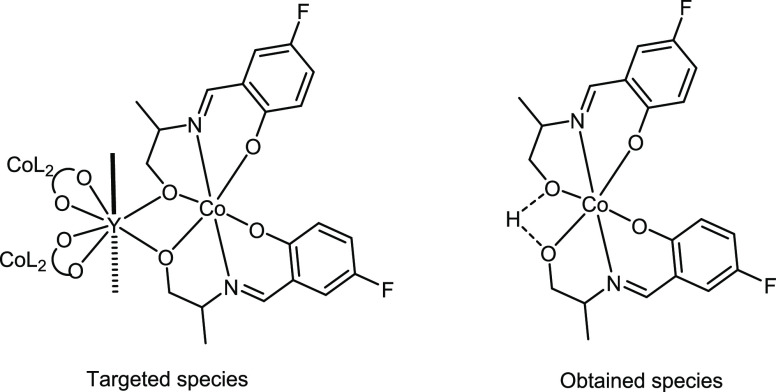
The Anticipated and Obtained Species Following the Synthetic Recipe Described in This Work

All amino acids (**A9**–**A12**, Table 3) were sensed successfully using a 1:50 ratio. Both complexes at 0 h are stable when an excess of amino acid is added. The sensed amino alcohol and amino acid analytes (^19^F NMR spectra found in Tables S10 and S11) were both *chiral* and *non-chiral* species. While the chiral species demonstrated how **C** can successfully sense and discriminate between the *S*- and *R*-configurations of each analyte by a chemical shift difference, the non-chiral species (**A5** and **A12**) were introduced to assess the sensing ability of this complex concerning molecular recognition. A split signal correspondent of a non-chiral species introduces a further use for these complexes and chiral discrimination, which is the sensing of species that do not require chiral discrimination by their configurational derivative or induce changes in the chiral environment. For example, this can be developed to detect and recognize specific organic or biological molecules. **A8** was not sensed, implying that the five-membered ring holding the 1,2-amino alcohol motif sterically hinders the ability of the motif to approach, and thus interact with, the sensor. To overcome this obstacle, we attempted to obtain the corresponding propeller-shaped structure (Scheme 3), replacing the bulky phenyl ring with a methyl group. Large red block-shaped crystals were instantaneously formed, almost quantitively; however, preliminary and sole single X-ray diffraction characterization (Figure S6) identified the formation of the neutral Co^III^ species, as shown in Scheme 3. Future efforts will optimize the reaction conditions to obtain the targeted propeller-based structure.

**Table 3:**
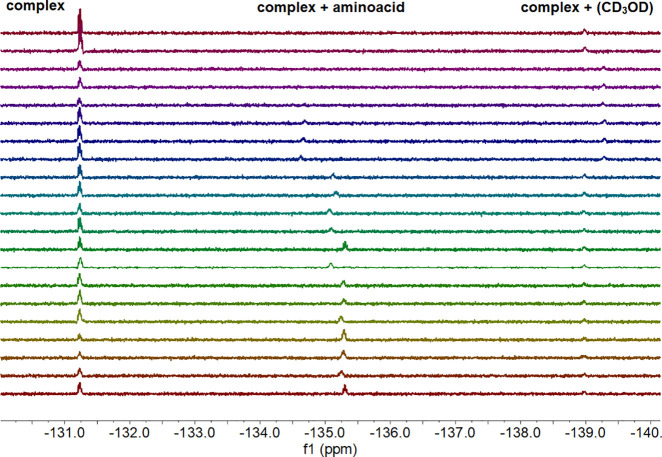
^19^F NMR Spectra of Complex (1 mM, CD_3_OD) and Different Amino Acids (50 mM, CD_3_OD)^a^

complex	amine	ratio	solvent	complex peak	complex + analyte peak	complex + solvent upfield peak	enantiomer difference
**C^S^**			CD_3_OD	–131.23		–138.99	
**C^R^**			CD_3_OD	–131.23		–138.99	
**C^S^**	**A9^D^**	1:50	CD_3_OD	–131.23		–139.28	
**C^S^**	**A9^L^**	1:50	CD_3_OD	–131.23		–139.28	
**C^S^**	**A9^D/L^**	1:50	CD_3_OD	–131.23		–139.28	
**C^R^**	**A9^D^**	1:50	CD_3_OD	–131.23	**–134.69**	–139.28	**0.03**
**C^R^**	**A9^L^**	1:50	CD_3_OD	–131.23	**–134.66**	–139.28	
**C^R^**	**A9^D/L^**	1:50	CD_3_OD	–131.23	–134.62	–139.28	
**C^S^**	**A10^D^**	1:50	CD_3_OD	–131.23	**–135.13**	–138.99	**0.03**
**C^S^**	**A10^L^**	1:50	CD_3_OD	–131.23	**–135.16**	–138.99	
**C^S^**	**A10^D/L^**	1:50	CD_3_OD	–131.23	–135.07	–138.99	
**C^R^**	**A10^D^**	1:50	CD_3_OD	–131.23	**–135.09**	–138.99	**0.22**
**C^R^**	**A10^L^**	1:50	CD_3_OD	–131.23	**–135.31**	–138.99	
**C^R^**	**A10^D/L^**	1:50	CD_3_OD	–131.23	–135.09	–138.99	
**C^S^**	**A11^D^**	1:50	CD_3_OD	–131.23	**–135.29**	–138.99	**0.00**
**C^S^**	**A11^L^**	1:50	CD_3_OD	–131.23	**–135.29**	–138.99	
**C^S^**	**A11^D/L^**	1:50	CD_3_OD	–131.23	–135.24	–138.99	
**C^R^**	**A11^D^**	1:50	CD_3_OD	–131.23	**–135.29**	–138.99	**0.00**
**C^R^**	**A11^L^**	1:50	CD_3_OD	–131.23	**–135.29**	–138.99	
**C^R^**	**A11^D/L^**	1:50	CD_3_OD	–131.23	–135.27	–138.99	
**C^S^**	**A12**	1:50	CD_3_OD	–131.23	–135.31	–138.99	

a The corresponding ^19^F NMR spectra are given in the order of the table (*T* = 303 K).

### Plausible Sensing Mechanisms

**Scheme 4:**
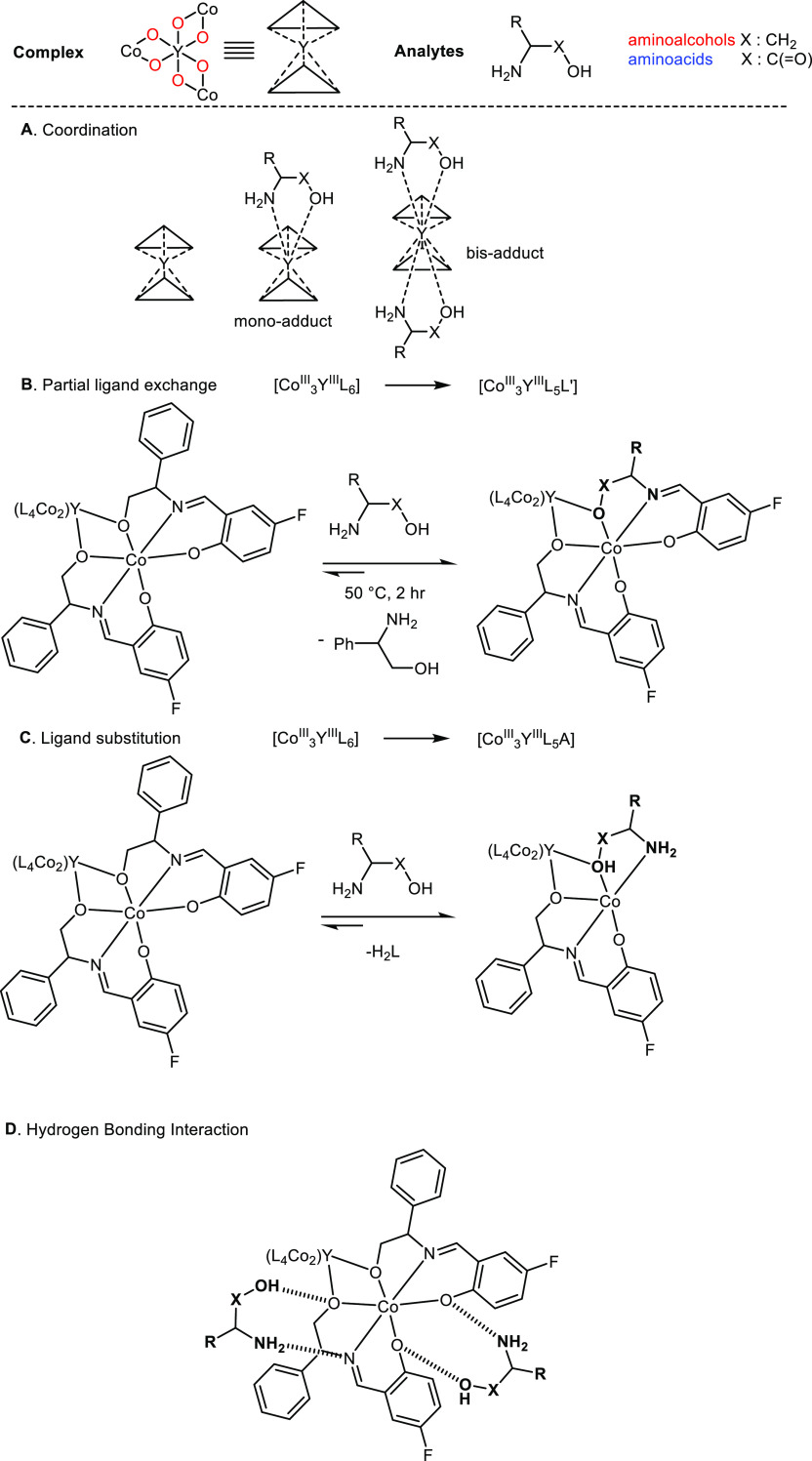
Plausible Sensing Mechanisms

Recognition of chiral and non-chiral analytes makes the mechanism for this complex–analyte interaction interesting. The 1,2-amino alcohol motif must be present on the analyte for sensing to occur. Further, it is unclear whether the split signal is representative of an induced change in the chiral environment affecting the inherent chirality of the [Co^III^_3_Y^III^L_6_] by coordination to the central Y^III^ or instead supports the notion that a ligand exchange interaction facilitates this additional peak or a weak H-bonding interaction occurs, as shown in Scheme 4. In the first case (A, Scheme 4), the resultant mono-adduct holds a capped trigonal antiprism geometry and would correspond to a split signal in the ^19^F NMR spectrum, differentiating from the original peak. As Y^III^ tends to favor a coordination number of 7 or 8, coordination of a further analyte to the Y^III^ metal center from the remaining plane cannot be excluded and would correspond to a bis-adduct. Here, the geometry becomes bicapped trigonal antiprismatic and would correspond to an additional signal in the ^19^F NMR spectrum. It is relevant to reiterate the additional signal that accompanied the **C** peak in CD_3_OD (see Table 3 and S6) at approximately −139 ppm. Coordination of CD_3_OD to the Y^III^ metal center can form {Co^III^_3_Y^III^L_6_(CD_3_OD)_*x*_} (*x* = 1 or 2) and, with the further introduction of an analyte, would observe three individual peaks corresponding to {Co^III^_3_Y^III^L_6_}, {Co^III^_3_Y^III^L_6_(analyte)}, and {Co^III^_3_Y^III^L_6_ (CD_3_OD)_x_}, similar to Table 4. In the second and third scenarios (B and C, Scheme 4), given that all analytes bear the same 1,2-amino alcohol motif, the additional signal found in the ^19^F NMR spectrum can be the result of a partial (B) or full (C) ligand exchange process that yields the formation of a new compound {Co^III^_3_Y^III^L_5_L′}, as this has been proposed in a previous work.^38^ Last but not least, numerous weak hydrogen bonding interactions between the complex and the analyte can occur in the solutions (a representative example, D, is drawn), which may cause the appearance of a second peak in the ^19^F NMR spectrum.

**Table 4:** Computed ^19^F NMR Chemical Shifts for the Resulting {Co^III^_3_Y^III^L_6_(Analyte)_*x*_} Complexes According to the Mechanisms in Scheme 4 and Comparison to the Reported Experimental NMR Values in This Work

	complex with analyte	mechanism	solvent	experimental ^19^F NMR shift (ppm)	computed ^19^F NMR shift (ppm)^a^^,^^b^	agreement (%)
1	complex only (Co_3_YL_6_)	none	MeOD	–131.23	–130.30	99.3%
2	complex only^c^ (Co_3_YL_6_)	none	MeOD	–131.23	–133.32	98.4%
3	A1R	partial ligand exchange	CDCl_3_	–132.05	–129.99	>99.9%
4	A1S	partial ligand exchange	CDCl_3_	–132.12	–130.43	98.7%
5	A1S^c^	partial ligand exchange	CDCl_3_	–132.12	–127.81	96.7%
6	A6R	partial ligand exchange	CDCl_3_	–132.72	–130.45	98.4%
7	A6S	partial ligand exchange	CDCl_3_	–132.62	–130.64	98.4%
8	A6S^c^	partial ligand exchange	CDCl_3_	–132.62	–127.92	97.6%
9	A9R	partial ligand exchange	MeOD	–134.66	–129.29	96%
10	A9S	partial ligand exchange	MeOD	–134.69	–129.64	96.2%
11	A1R	6× partial ligand exchange^d^	CDCl_3_	–132.05	–129.11	99.3%
12	A1S	6× partial ligand exchange	CDCl_3_	–132.12	–130.43	98.7%
13	A6R	6× partial ligand exchange	CDCl_3_	–132.72	–130.64	98.5%
14	A6S	6× partial ligand exchange	CDCl_3_	–132.62	–131.64	99.2%
15	A1R	ligand substitution	CDCl_3_	–132.05	–131.25	99.1%
16	A1S	ligand substitution	CDCl_3_	–132.12	–131.11	99.2%
17	A6R	ligand substitution	CDCl_3_	–132.72	–131.38	99.1%
18	A6S	ligand substitution	CDCl_3_	–132.62	–131.30	98.9%
19	A9R	ligand substitution	MeOD	–134.66	–129.21	96%
20	A9S	ligand substitution	MeOD	–134.69	–129.36	92/9%

a Calculations were performed at the B3LYP/SDD level of theory with the polarizable continuum model (PCM) as the implicit solvent model.

b Fluorobenzene (C_6_H_5_F) was used as the ^19^F NMR reference calculated at the same level of theory.

c This calculation was performed at the M06/Def2-TZVP level.

d Saturated system with six ligands partially exchanged.

### Control Experiments and Theoretical Studies

We performed computational calculations; recorded ^1^H, ^89^Y, and additional ^19^F NMR data; and used other analytes to probe the mechanistic path.

**Figure 3:**
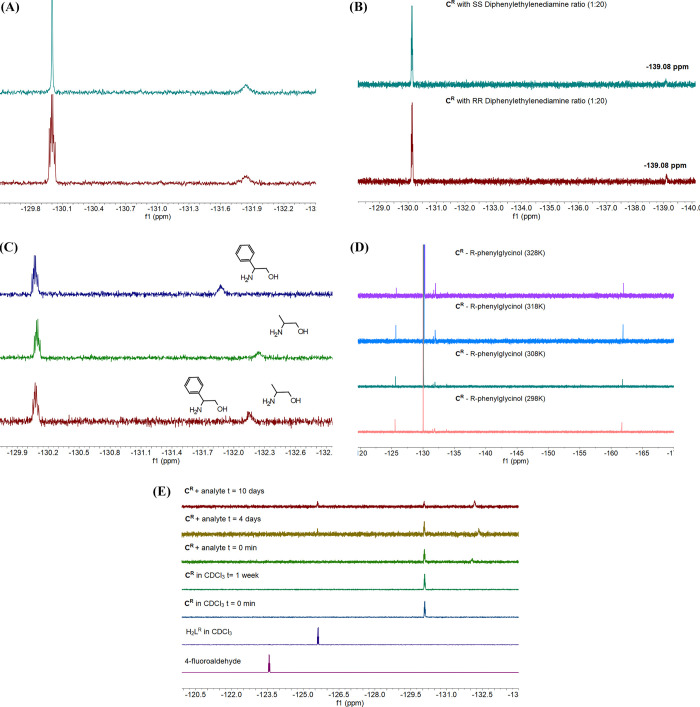
(A) A comparison of the ^1^H-decoupled ^19^F NMR of the **C^R^** complex with phenylglycinol. (B) The ^19^F NMR spectra of the **C^R^** complex with SS or RR diphenylethylenediamine in a 1:20 ratio in the CDCl_3_:DMSO 9:1 ratio. (C) The ^19^F NMR of the **C^R^** complex with different amino alcohols in the 1:100 ratio at 1 mM concentration. (D) Variable-temperature ^19^F NMR data indicating that the increase in the temperature facilitates the ligand exchange. (E) ^19^F NMR data of complex (1 mM, CDCl_3_) + analyte (1 mM, CDCl_3_) samples left undisturbed after 4 and 10 days, and other compounds for comparison at *T* = 303 K.

Initially, we performed ^1^H-decoupled ^19^F NMR experiments to validate the shape and nature of the observed peaks (Figure 3A). The data were recorded with the same number of scans, and the samples had the same concentration. In the decoupled spectrum, the multiplet peak of the complex appears as single with the same intensity; however, the “analyte+complex” peak retains its broad character, indicative of a chemical exchange and several types of interactions. Using the chiral analytes containing the N–N motif such as SS or RR diphenylethylenediamine in the 1:20 ratio (Figure 3B) provided a second peak at −139.08 ppm. These data were recorded with the inevitable use of coordinating d^6^-DMSO solvent for solubility purposes. Comparing the data in Table 4, this peak can be attributed to the {Co^III^_3_Y^III^L_6_(DMSO)_*x*_} (*x* = 1 or 2) species; therefore, sensing of analytes with the NN pocket proves challenging. Then, we recorded a sample in CDCl_3_ (Figure 3C) containing **C^R^**, (*R*)-(−)-2-phenylglycinol, and (*S*)-(+)-2-amino-1-propanol in a 1:100:100 ratio at 1 mM concentration. The samples of **C^R^** with (*R*)-(−)-2-phenylglycinol and (*S*)-(+)-2-amino-1-propanol in a 1:100 ratio were also recorded and shown for convenience. These data indicate that **C^R^** cannot be used to discriminate between two analytes with similar pocket sizes since only one, possibly average, peak at −132.15 ppm is observed. Next, our studies concentrated on increasing the temperature to facilitate ligand exchange, if any, or alter the H-bonding interactions, thus providing better spectral resolution and improved chemical shift differences (Figure 3D). These data indicate that a full ligand (mechanism C, Scheme 4) exchange process takes place since two new peaks appear; one corresponds to the free ligand (−126 ppm) and the second (∼163 ppm) to an unknown species. Then, we recorded ^1^H–^89^Y data for the complex and the complex with an excess of phenylglycinol (Figure S7). The data of the complex show an interaction of the Y^III^ center with the methylenic protons of the ligand (Figure S7 up), which is retained when an excess of phenylglycinol is present (Figure S7 down); however, no other peak that would suggest the presence of a {Co^III^_3_Y^III^L_6_(analyte)_*x*_}, since the analyte is in excess, is observed. Last, we recorded the data of the same sample (complex + analyte ratio 1:20) after 4 and 10 days, and three peaks can be identified (Figure 3E). These three peaks can be attributed to the free ligand (H_2_L), the complex (C), and the complex + analyte. After 10 days, the intensity of the complex peak significantly drops, whereas the intensity of the peak that corresponds to the free ligand significantly increases, signifying that a dynamic ligand substitution is responsible for the sensing process. We then performed theoretical calculations, trialed B3LYP/SDD and M06/Def2-TZVP levels in the recently reported Rh system,^38^ for the first time, to evaluate their efficacy (Figure S8, Tables S10 and S11, respectively), and identified optimum performance with the M06/Def2-TZVP level (Table S10). Encouraged by the excellent agreement of the computed ^19^F NMR chemical shifts, we computed the ^19^F NMR chemical shifts for the four plausible mechanisms shown in Scheme 4. As depicted in Table 4, there is an excellent agreement between the computed ^19^F NMR chemical shifts of the {Co^III^_3_Y^III^L_6_} complex alone in MeOD for both levels of theory employed (entries 1 and 2) but slightly better for the B3LYP/SDD system (entry 1).

Our attempts to compute the complex with the mono- and bis-adduct, which means {Co^III^_3_Y^III^L_6_ + (analyte)_*x*_, where *x* = 1 or 2, and analyte (**A1**, **A6**, and **A9**) (Scheme 4, mechanism A) suggest that complex {Co^III^_3_Y^III^L_6_} is very stable in all cases (Figure S9, Table S12). The computed ^19^F NMR chemical shifts for the resulting complex analytes from the partial ligand exchange mechanism (Scheme 4, mechanism B) are in very good agreement with the experimental values (Table 4, entries 3–10). A slightly better agreement was observed for the computed NMR values for the {Co^III^_3_Y^III^L_6_} complexes when a complete (six times) ligand exchange takes place (Table 4, entries 11–14). Under the experimental evidence gathered in this work, we can argue that the best agreement can be met with the resulting {Co^III^_3_Y^III^L5A} species from the ligand substitution mechanism (C, Scheme 4, Table 4, entries 15 to 20). Bearing all these in mind, and considering that complex **C** is incapable of sensing diols and diamines, the latter due to solubility issues, our chemical intuition inclines mechanism C, Scheme 4, as the most probable.

### Comparison with Previous Methods

Referring to the previous work of Zhao and Swager,^35^ utilizing a Pd-pincer complex and ^19^F NMR for the sensing of chiral amines, it is vital to explore the differences in their chiral chemosensory system as opposed to our system. First, the Pd sensor is unsuitable for amino acids; it allows discrimination of amino alcohols using 1:1 and 1:2 ratios of sensor:analyte with chemical shift differences of enantiomeric pairs ranging from 0.1 to 1 ppm. Comparatively, the discrimination of amino alcohol enantiomeric pairs using these Co^III^_3_Y^III^ complexes presented a range of 0.02–0.18 ppm, with the higher results of a sensor:amine ratio 1:50. In addition to the advantages—the need to use less amount of the sensor to detect and discriminate between the analytes and boasting broader chemical shift differences—the window for detection, which is the shift difference between the original sensor peak and analyte peak, is broader. For the Pd sensor, the maximum window is 2.5 ppm, whereas using the **C^S^** or **C^R^**, this difference increases to 8 ppm. This increased difference benefits from a more straightforward interpretation of the resultant spectra and differentiation between existing complex and new analyte signals. Limitations arise with the complex where it cannot sense monoamines, as they lack the significant 1,2-amino alcohol motif present in both amino alcohol and amino acid analogues—the discrimination of monoamines is possible using the Pd complex; however, it again requires a more extensive complex loading with a ratio of 1:1 or 1:2. Moreover, *ee* determination is possible using the palladium sensor but not doable for **C^S^** or **C^R^**.

Song et al.^38^ used fluorinated Rh complex and discriminated chiral enantiomers of monoamines, diamines, and amino acids, thus outperforming **C^S^** or **C^R^** with enantiomeric differences of up to 0.21 ppm. The ratio of the sensor to the analyte is 1:1. They also found that changing the solvent from CDCl_3_ to DMSO-d_6_ increases the chemical shift difference between the two signals of an enantiomeric pair reproduced in other literature using ^19^F chemosensing systems.^16^ However, examining the subsequent ^19^F NMR spectra for both amino alcohols and amino acids in this Rh system, a pair of fluorine resonances can be found per analyte, as on coordination of the analyte to the Rh sensor, the sensor becomes asymmetric with each fluorine inequivalent. Consequently, it is unclear which signal of each pair of signals should be used to compare the enantiomer difference in chemical shift. We only observe an additional fluorine resonance representing the analyte alongside the original peak and providing more facile discrimination using uncomplicated spectra for any amino alcohol and the amino acid analyte in question. To follow, taking even the farthest signal distance of each enantiomeric pair, a maximum of 1 ppm is found for both amino acids and amino alcohols, which again is less than the most significant values for our complexes—0.22 ppm for amino acids and 0.18 ppm for amino alcohols (both in the 1:50 ratio). Interestingly, when optimizing sensing conditions, Song et al. found that increasing the equivalence of the sensor and extending the reaction time to 2 h with a reading taken at 50 °C showed more precise fluorine signals, which is therefore indicative of equilibrium favoring the coordinated analyte complex product. A more general comparison of Zhao and Swager and Song et al.’s complexes^35,38^ versus ours highlights the incorporation of abundant, low-cost, and non-toxic metals and the use of commercially available ligands for the synthesis of **C^S^** and **C^R^**.

## Conclusions

We present the first example of a 3d/4f ^19^F-NMR chemosensory system and identify the scope and limitations of this method. These air-stable and easy-to-make complexes are built from non-toxic and inexpensive metals and retain their structure in the solution for a prolonged period; however, the crystallization solvent may impact a needless racemization process or improvise unnecessary impurities. Complexes **C^S^** and **C^R^** are applicable to sense a specific type of analytes bearing an NH_2_CX-CH_X_-OH pocket via a dynamic ligand exchange mechanism (C, Scheme 4). Despite the limited analyte library, our method imposes an extreme sensor:analyte ratio (1:20 or 1:50) and a broad sensing window (8 ppm over 0.8 ppm, as seen in other studies), which are advantageous over other techniques and thus can be used to detect and recognize organic or biological molecules bearing this specific motif at millimolar concentrations. Future work will focus on modifying the existing propeller-shaped motif by (a) replacing the central Y^III^ unit of the propeller-shaped structure with Gd^III^ or Eu^III^/Tb^III^ ions to allow sensing investigations of the related species with EPR or fluorescence, (b) modifying the organic ligand to enhance the F signal or remove aromatic interactions, and (c) overcoming the racemization effect during crystallization, which will become prone to investigate the chemosensing amine abilities of this 3d/4f system with CD spectroscopy.

## Data Availability

CCDC deposition numbers 2195856–2195858 contain the supplementary crystallographic data for this paper.

## Supporting Information Available

Copies of ESI-MS data, ^1^H and ^19^F NMR, TGA, UV–vis, and coordinates for theoretical calculations (PDF)

## References

[ref1] Willia K.; Lee E. Importance of Drug Enantiomers in Clinical Pharmacology. Drugs. Springer October 1985, 30, 333–354. 10.2165/00003495-198530040-00003. 3905334

[ref2] Nguyen L. A.; He H.; Pham-Huy C. Chiral Drugs: An Overview. Int. J. Biomed. Sci. 2006, 2, 85–100. 23674971 PMC3614593

[ref3] Brooks W. H.; Guida W. C.; Daniel K. G. The Significance of Chirality in Drug Design and Development. Curr. Top. Med. Chem. 2011, 11, 760–770. 10.2174/156802611795165098. 21291399 PMC5765859

[ref4] Polavarapu P. L.; Scalmani G.; Hawkins E. K.; Rizzo C.; Jeirath N.; Ibnusaud I.; Habel D.; Nair D. S.; Haleema S. Importance of Solvation in Understanding the Chiroptical Spectra of Natural Products in Solution Phase: Garcinia Acid Dimethyl Ester. J. Nat. Prod. 2011, 74, 321–328. 10.1021/np100512w. 21114277 PMC3699186

[ref5] Allred T. K.; Manoni F.; Harran P. G. Exploring the Boundaries of “Practical:” De Novo Syntheses of Complex Natural Product-Based Drug Candidates. Chem. Rev. 2017, 117, 11994–12051. 10.1021/acs.chemrev.7b00126. 28603978

[ref6] Li X.; Duan M.; Yu P.; Houk K. N.; Sun J. Organocatalytic Enantioselective Dearomatization of Thiophenes by 1,10-Conjugate Addition of Indole Imine Methides. Nat. Commun. 2021, 12, 4881 10.1038/s41467-021-25165-7. 34385441 PMC8361129

[ref7] Bentley K. W.; Wolf C. Stereodynamic Chemosensor with Selective Circular Dichroism and Fluorescence Readout for in Situ Determination of Absolute Configuration, Enantiomeric Excess, and Concentration of Chiral Compounds. J. Am. Chem. Soc. 2013, 135, 12200–12203. 10.1021/ja406259p. 23909867

[ref8] Shcherbakova E. G.; Brega V.; Minami T.; Sheykhi S.; James T. D.; Anzenbacher P. Toward Fluorescence-Based High-Throughput Screening for Enantiomeric Excess in Amines and Amino Acid Derivatives. Chem. – Eur. J. 2016, 22, 10074–10080. 10.1002/chem.201601614. 27271215

[ref9] De los Santos Z. A.; MacAvaney S.; Russell K.; Wolf C. Tandem Use of Optical Sensing and Machine Learning for the Determination of Absolute Configuration, Enantiomeric and Diastereomeric Ratios, and Concentration of Chiral Samples. Angew. Chem., Int. Ed. 2020, 59, 2440–2448. 10.1002/anie.201912904. 31714669

[ref10] De los Santos Z. A.; Lynch C. C.; Wolf C. Optical Chirality Sensing with an Auxiliary-Free Earth-Abundant Cobalt Probe. Angew. Chem., Int. Ed. 2019, 58, 1198–1202. 10.1002/anie.201811761. 30500091

[ref11] Bentley K. W.; Proano D.; Wolf C. Chirality Imprinting and Direct Asymmetric Reaction Screening Using a Stereodynamic Brønsted/Lewis Acid Receptor. Nat. Commun. 2016, 7, 12539 10.1038/ncomms12539. 27549926 PMC4996974

[ref12] Dragna J. M.; Pescitelli G.; Tran L.; Lynch V. M.; Anslyn E. V.; Di Bari L. In Situ Assembly of Octahedral Fe(II) Complexes for the Enantiomeric Excess Determination of Chiral Amines Using Circular Dichroism Spectroscopy. J. Am. Chem. Soc. 2012, 134, 4398–4407. 10.1021/ja211768v. 22272943 PMC3329375

[ref13] Chaudhari S. R.; Suryaprakash N. Simple and Efficient Methods for Discrimination of Chiral Diacids and Chiral Alpha-Methyl Amines. Org. Biomol. Chem. 2012, 10, 6410–6419. 10.1039/c2ob25599e. 22735343

[ref14] Xu Z.; Liu C.; Zhao S.; Chen S.; Zhao Y. Molecular Sensors for NMR-Based Detection. Chem. Rev. 2019, 119, 195–230. 10.1021/acs.chemrev.8b00202. 30080024

[ref15] Chen Z.; Yang M.; Sun Z.; Zhang X.; Xu J.; Bian G.; Song L. Chiral Discrimination by a Binuclear Pd Complex Sensor Using 31P{1H} NMR. Anal. Chem. 2019, 91, 14591–14596. 10.1021/acs.analchem.9b03661. 31657901

[ref16] Dong C.; Xu Z.; Wen L.; He S.; Wu J.; Deng Q. H.; Zhao Y. Tailoring Sensors and Solvents for Optimal Analysis of Complex Mixtures Via Discriminative 19F NMR Chemosensing. Anal. Chem. 2021, 93, 2968–2973. 10.1021/acs.analchem.0c04768. 33503366

[ref17] Seo M. S.; Kim H. 1H NMR Chiral Analysis of Charged Molecules via Ion Pairing with Aluminum Complexes. J. Am. Chem. Soc. 2015, 137, 14190–14195. 10.1021/jacs.5b09555. 26479579

[ref18] Puentes C. M.; Wenzel T. J. Phosphated Cyclodextrins as Water-Soluble Chiral NMR Solvating Agents for Cationic Compounds. Beilstein J. Org. Chem. 2017, 13, 43–53. 10.3762/bjoc.13.6. 28179947 PMC5238554

[ref19] Sun Z.; Chen Z.; Wang Y.; Zhang X.; Xu J.; Bian G.; Song L. Chiral Discrimination of Varied Ammonium Compounds through 1h Nmr Using a Binuclear Ti Complex Sensor. Org. Lett. 2020, 22, 589–593. 10.1021/acs.orglett.9b04373. 31913635

[ref20] Hinckley C. C. Paramagnetic Shifts in Solutions of Cholesterol and the Dipyridine Adduct of Trisdipivalomethanatoeuropium(III). A Shift Reagent. J. Am. Chem. Soc. 1969, 91, 5160–5162. 10.1021/ja01046a038. 5798101

[ref21] Dale J. A.; Dull D. L.; Mosher H. S. α-Methoxy-α-Trifluoromethylphenylacetic Acid, a Versatile Reagent for the Determination of Enantiomeric Composition of Alcohols and Amines. J. Org. Chem. 1969, 34, 2543–2549. 10.1021/jo01261a013.

[ref22] Pirkle W. H.; Sikkenga D. L.; Pavlin M. S. Nuclear Magnetic Resonance Determination of Enantiomeric Composition and Absolute Configuration of γ-Lactones Using Chiral 2,2,2-Trifluoro-1-(9-Anthryl)Ethanol. J. Org. Chem. 1977, 42, 384–387. 10.1021/jo00422a061.

[ref23] Bravo J.; Cativiela C.; Chaves J. E.; Navarro R.; Urriolabeitia E. P. 31P NMR Spectroscopy as a Powerful Tool for the Determination of Enantiomeric Excess and Absolute Configurations of α-Amino Acids. Inorg. Chem. 2003, 42, 1006–1013. 10.1021/ic0204878. 12588132

[ref24] Gimenez D.; Phelan A.; Murphy C. D.; Cobb S. L. 19F NMR as a Tool in Chemical Biology. Beilstein J. Org. Chem. 2021, 17, 293–318. 10.3762/BJOC.17.28. 33564338 PMC7849273

[ref25] Chen H.; Viel S.; Ziarelli F.; Peng L. 19F NMR: A Valuable Tool for Studying Biological Events. Chem. Soc. Rev. 2013, 42, 7971–7982. 10.1039/c3cs60129c. 23864138

[ref26] Rosenau C. P.; Jelier B. J.; Gossert A. D.; Togni A. Exposing the Origins of Irreproducibility in Fluorine NMR Spectroscopy. Angew. Chem., Int. Ed. 2018, 57, 9528–9533. 10.1002/anie.201802620. 29663671

[ref27] Norton R. S.; Leung E. W. W.; Chandrashekaran I. R.; MacRaild C. A. Applications of 19F-NMR in Fragment-Based Drug Discovery. Molecules 2016, 21, 860 10.3390/molecules21070860. 27438818 PMC6273323

[ref28] Buchholz C. R.; Pomerantz W. C. K. 19F NMR Viewed through Two Different Lenses: Ligand-Observed and Protein-Observed 19F NMR Applications for Fragment-Based Drug Discovery. RSC Chem. Biol. 2021, 2, 1312–1330. 10.1039/d1cb00085c. 34704040 PMC8496043

[ref29] Cobb S. L.; Murphy C. D. 19F NMR Applications in Chemical Biology. J. Fluorine Chem. 2009, 130, 132–143. 10.1016/j.jfluchem.2008.11.003.

[ref30] Boeszoermenyi A.; Ogórek B.; Jain A.; Arthanari H.; Wagner G. The Precious Fluorine on the Ring: Fluorine NMR for Biological Systems. J. Biomol. NMR 2020, 74, 365–379. 10.1007/s10858-020-00331-z. 32651751 PMC7539674

[ref31] Dolbier W. R. Guide to Fluorine NMR for Organic Chemists; John Wiley & Sons, Inc., 2016, 10.1002/9781118831106.

[ref32] Yu J. X.; Hallac R. R.; Chiguru S.; Mason R. P. New Frontiers and Developing Applications in 19F NMR. Prog. Nucl. Magn. Reson. Spectrosc. 2013, 70, 25–49. 10.1016/J.PNMRS.2012.10.001. 23540575 PMC3613763

[ref33] Yu J.; Kodibagkar V.; Cui W.; Mason R. 19F: A Versatile Reporter for Non-Invasive Physiology and Pharmacology Using Magnetic Resonance. Curr. Med. Chem. 2005, 12, 819–848. 10.2174/0929867053507342. 15853714

[ref34] Danielson M. A.; Falke J. J. Use of 19F NMR to Probe Protein Structure and Conformational Changes. Annu. Rev. Biophys. Biomol. Struct. 1996, 25, 163–195. 10.1146/annurev.bb.25.060196.001115. 8800468 PMC2899692

[ref35] Zhao Y.; Swager T. M. Simultaneous Chirality Sensing of Multiple Amines by 19F NMR. J. Am. Chem. Soc. 2015, 137, 3221–3224. 10.1021/jacs.5b00556. 25723526 PMC5818995

[ref36] Reed J. E.; White A. J. P.; Neidle S.; Vilar R. Effect of Metal Coordination on the Interaction of Substituted Phenanthroline and Pyridine Ligands with Quadruplex DNA. Dalton Trans. 2009, 14, 2558–2568. 10.1039/b820086f. 19319401

[ref37] Yamnitz C. R.; Negin S.; Carasel I. A.; Winter R. K.; Gokel G. W. Dianilides of Dipicolinic Acid Function as Synthetic Chloride Channels. Chem. Commun. 2010, 46, 2838–2840. 10.1039/b924812a. 20369200

[ref38] Wang W.; Xia X.; Bian G.; Song L. A Chiral Sensor for Recognition of Varied Amines Based on 19 F NMR Signals of Newly Designed Rhodium Complexes. Chem. Commun. 2019, 55, 6098–6101. 10.1039/c9cc01942a. 31069349

[ref39] Wang C.; Chen L. A.; Huo H.; Shen X.; Harms K.; Gong L.; Meggers E. Asymmetric Lewis Acid Catalysis Directed by Octahedral Rhodium Centrochirality. Chem. Sci. 2015, 6, 1094–1100. 10.1039/c4sc03101f. 29560197 PMC5811158

[ref40] Huo H.; Shen X.; Wang C.; Zhang L.; Röse P.; Chen L. A.; Harms K.; Marsch M.; Hilt G.; Meggers E. Asymmetric Photoredox Transition-Metal Catalysis Activated by Visible Light. Nature 2014, 515, 100–103. 10.1038/nature13892. 25373679

[ref41] Jankolovits J.; Kampf J. W.; Pecoraro V. L. Insight into the Structural Versatility of the Ln(III)[15-Metallacrown-5] Platform by Comparing Analogs with Ni(II), Cu(II), and Zn(II) Ring Ions. Polyhedron 2013, 52, 491–499. 10.1016/j.poly.2012.08.046.

[ref42] Bo L.; Wang S.; Schipper D.; Yang X.; Zhu T.; Tao J. Self-Assembly of Luminescent Zn-Ln (Ln = Sm and Nd) Nanoclusters with a Long-Chain Schiff Base Ligand. New J. Chem. 2018, 42, 7241–7246. 10.1039/c7nj04967f.

[ref43] Zhang F.; Li Z.; Ge T.; Yao H.; Li G.; Lu H.; Zhu Y. Four Novel Frameworks Built by Imidazole-Based Dicarboxylate Ligands: Hydro(Solvo)Thermal Synthesis, Crystal Structures, and Properties. Inorg. Chem. 2010, 49, 3776–3788. 10.1021/ic902483m. 20230024

[ref44] Liu J. L.; Wu J. Y.; Chen Y. C.; Mereacre V.; Powell A. K.; Ungur L.; Chibotaru L. F.; Chen X. M.; Tong M. L. A Heterometallic FeII-DyIII Single-Molecule Magnet with a Record Anisotropy Barrier. Angew. Chemie - Int. Ed. 2014, 53, 12966–12970. 10.1002/anie.201407799. 25256293

[ref45] Schmitz S.; Kovalchuk A.; Martín-Rodríguez A.; van Leusen J.; Izarova N. V.; Bourone S. D. M.; Ai Y.; Ruiz E.; Chiechi R. C.; Kögerler P.; Monakhov K. Y. Element-Selective Molecular Charge Transport Characteristics of Binuclear Copper(II)-Lanthanide(III) Complexes. Inorg. Chem. 2018, 57, 9274–9285. 10.1021/acs.inorgchem.8b01279. 30040402

[ref46] Dey A.; Acharya J.; Chandrasekhar V. Heterometallic 3d–4f Complexes as Single-Molecule Magnets. Chem. - Asian J. 2019, 14, 4433–4453. 10.1002/asia.201900897. 31328881

[ref47] Wilson L. R. B.; Coletta M.; Evangelisiti M.; Piligkos S.; Dalgarno S. J.; Brechin E. K. The Coordination Chemistry of P-Tert-Butylcalix[4]Arene with Paramagnetic Transition and Lanthanide Metal Ions: An Edinburgh Perspective. Dalton Trans. 2022, 51, 4213–4226. 10.1039/d2dt00152g. 35170617

[ref48] Griffiths K.; Kumar P.; Akien G. R.; Chilton N. F.; Abdul-Sada A.; Tizzard G. J.; Coles S. J.; Kostakis G. E. Tetranuclear Zn/4f Coordination Clusters as Highly Efficient Catalysts for Friedel Crafts Alkylation. Chem. Commun. 2016, 52, 7866–7869. 10.1039/C6CC03608B. 27248829

[ref49] Griffiths K.; Kostakis G. E. Transformative 3d-4f Coordination Cluster Carriers. Dalton Trans. 2018, 47, 12011–12034. 10.1039/C8DT02362J. 30051130

[ref50] Griffiths K.; Gallop C. W. D.; Abdul-Sada A.; Vargas A.; Navarro O.; Kostakis G. E. Heteronuclear 3 d/DyIII Coordination Clusters as Catalysts in a Domino Reaction. Chem. - A Eur. J. 2015, 21, 6358–6361. 10.1002/chem.201500505. 25766091

[ref51] Griffiths K.; Tsipis A. C.; Kumar P.; Townrow O. P. E.; Abdul-Sada A.; Akien G. R.; Baldansuren A.; Spivey A. C.; Kostakis G. E. 3d/4f Coordination Clusters as Cooperative Catalysts for Highly Diastereoselective Michael Addition Reactions. Inorg. Chem. 2017, 56, 9563–9573. 10.1021/acs.inorgchem.7b01011. 28783350

[ref52] Sampani S. I.; McGown A.; Vargas A.; Abdul-Sada A.; Tizzard G. J.; Coles S. J.; Spencer J.; Kostakis G. E. Solvent-Free Synthesis and Key Intermediate Isolation in Ni 2 Dy 2 Catalyst Development in the Domino Ring-Opening Electrocyclization Reaction of Furfural and Amines. J. Org. Chem. 2019, 84, 6858–6867. 10.1021/acs.joc.9b00608. 31074278

[ref53] Wang L.; Xu C.; Han Q.; Tang X.; Zhou P.; Zhang R.; Gao G.; Xu B.; Qin W.; Liu W. Ambient Chemical Fixation of CO2 Using a Highly Efficient Heterometallic Helicate Catalyst System. Chem. Commun. 2018, 54, 2212–2215. 10.1039/C7CC09092G. 29336442

[ref54] Xu R.; Hua L.; Li X.; Yao Y.; Leng X.; Chen Y. Rare-Earth/Zinc Heterometallic Complexes Containing Both Alkoxy-Amino-Bis(Phenolato) and Chiral Salen Ligands: Synthesis and Catalytic Application for Copolymerization of CO2 with Cyclohexene Oxide. Dalt. Trans. 2019, 48, 10565–10573. 10.1039/c9dt00064j. 31215925

[ref55] Ramirez B. L.; Lu C. C. Rare-Earth Supported Nickel Catalysts for Alkyne Semihydrogenation: Chemo- And Regioselectivity Impacted by the Lewis Acidity and Size of the Support. J. Am. Chem. Soc. 2020, 142, 5396–5407. 10.1021/jacs.0c00905. 32091218

[ref56] Chen S.; Mereacre V.; Zhao Z.; Zhang W.; Zhang M.; He Z. Targeted Replacement: Systematic Studies of Dodecanuclear {MIII6LnIII6} Coordination Clusters (M = Cr, Co; Ln = Dy, Y). Dalton Trans. 2018, 47, 7456–7462. 10.1039/C8DT01289J. 29785423

[ref57] Goura J.; Guillaume R.; Rivière E.; Chandrasekhar V.; Rivie E. Hexanuclear, Heterometallic, Ni_3_n_3_ Complexes Possessing O-Capped Homo- and Heterometallic Structural Subunits: SMM Behavior of the Dysprosium Analogue. Inorg. Chem. 2014, 53, 7815–7823. 10.1021/ic403090z. 25050753

[ref58] Chandrasekhar V.; Dey A.; Das S.; Rouzières M.; Clérac R. Syntheses, Structures, and Magnetic Properties of a Family of Heterometallic Heptanuclear [Cu5Ln2] (Ln = Y(III), Lu(III), Dy(III), Ho(III), Er(III), and Yb(III)) Complexes: Observation of SMM Behavior for the Dy(III) and Ho(III) Analogues. Inorg. Chem. 2013, 52, 2588–2598. 10.1021/ic302614k. 23428002

[ref59] Moreno Pineda E.; Chilton N. F.; Tuna F.; Winpenny R. E. P.; McInnes E. J. L. Systematic Study of a Family of Butterfly-Like {M2Ln2} Molecular Magnets (M = MgII, MnIII, CoII, NiII, and CuII; Ln = YIII, GdIII, TbIII, DyIII, HoIII, and ErIII). Inorg. Chem. 2015, 54, 5930–5941. 10.1021/acs.inorgchem.5b00746. 26016421

[ref60] Hooper T. N.; Schnack J.; Piligkos S.; Evangelisti M.; Brechin E. K. The Importance of Being Exchanged: [Gd(III)4M(II)8(OH)8(L)8(O2CR)8]4+ Clusters for Magnetic Refrigeration. Angew. Chem., Int. Ed. 2012, 51, 4633–4636. 10.1002/anie.201200072. 22473842

[ref61] Zhu Y.-Y.; Zhang Y.-Q.; Yin T.-T.; Gao C.; Wang B.-W.; Gao S. A Family of Co(II)Co(III)3 Single-Ion Magnets with Zero-Field Slow Magnetic Relaxation: Fine Tuning of Energy Barrier by Remote Substituent and Counter Cation. Inorg. Chem. 2015, 54, 5475–5486. 10.1021/acs.inorgchem.5b00526. 25984913

[ref62] Mayans J.; Font-Bardia M.; Di Bari L.; Górecki M.; Escuer A. Chiral [MnIIMnIII3M′] (M′=NaI, CaII, MnII) and [MnIIMnIII6NaI2] Clusters Built from an Enantiomerically Pure Schiff Base: Synthetic, Chiroptical, and Magnetic Properties. Chem. - A Eur. J. 2018, 24, 18705–18717. 10.1002/chem.201803730. 30230054

[ref63] Pilichos E.; Escuer A.; Font-Bardia M.; Mayans J. Chiral Versus Non-Chiral [MnIII6MnIINaI], [MnIII6MnII2NaI2] and [MnIII3MnIINaI] Clusters Derived from Schiff Bases or the Fight for Symmetry. Chem. - A Eur. J. 2020, 26, 13053–13062. 10.1002/chem.202001656. 32428307

[ref64] Escuer A.; Mayans J.; Font-Bardia M.; Górecki M.; Bari L. D. Syntheses, Structures, and Chiroptical and Magnetic Properties of Chiral Clusters Built from Schiff Bases: A Novel [MnIIMnIII6NaI2] Core. Dalton Trans. 2017, 46, 6514–6517. 10.1039/C7DT00811B. 28426054

[ref65] Hu P.; Wang X. N.; Jiang C. G.; Yu F.; Li B.; Zhuang G. L.; Zhang T. Nanosized Chiral [Mn6Ln2] Clusters Modeled by Enantiomeric Schiff Base Derivatives: Synthesis, Crystal Structures, and Magnetic Properties. Inorg. Chem. 2018, 57, 8639–8645. 10.1021/acs.inorgchem.8b01423. 29962201

[ref66] Mayans J.; Font-Bardia M.; Escuer A. Chiroptical and Magnetic Properties of Star-Shaped Fe III 4 Complexes from Chiral Schiff Bases. Structural and Magnetic Correlations Based on Continuous Shape Measures. Dalton Trans. 2018, 47, 8392–8401. 10.1039/c8dt01684d. 29897079

[ref67] Spivey A. C.; Kostakis G. E., to be submitted.

[ref68] Veits G. K.; Read de Alaniz J. Dysprosium(III) Catalysis in Organic Synthesis. Tetrahedron 2012, 68, 2015–2026. 10.1016/j.tet.2011.11.042.

[ref69] Yu D.; Thai V. T.; Palmer L. I.; Veits G. K.; Cook J. E.; Read De Alaniz J.; Hein J. E. Importance of Off-Cycle Species in the Acid-Catalyzed Aza-Piancatelli Rearrangement. J. Org. Chem. 2013, 78, 12784–12789. 10.1021/jo402155b. 24304006

[ref70] Singh R.; Banerjee A.; Colacio E.; Rajak K. K. Enantiopure Tetranuclear Iron(III) Complexes Using Chiral Reduced Schiff Base Ligands: Synthesis, Structure, Spectroscopy, Magnetic Properties, and DFT Studies. Inorg. Chem. 2009, 48, 4753–4762. 10.1021/ic802206q. 19466801

[ref71] Clemens J. B.; Kibar O.; Chachisvilis M. A Molecular Propeller Effect for Chiral Separation and Analysis. Nat. Commun. 2015, 6, 1–10. 10.1038/ncomms8868. PMC4525176 26216219

[ref72] Zhang Y.; Calupitan J. P.; Rojas T.; Tumbleson R.; Erbland G.; Kammerer C.; Ajayi T. M.; Wang S.; Curtiss L. A.; Ngo A. T.; Ulloa S. E.; Rapenne G.; Hla S. W. A Chiral Molecular Propeller Designed for Unidirectional Rotations on a Surface. Nat. Commun. 2019, 10, 1–9. 10.1038/s41467-019-11737-1. 31431627 PMC6702202

[ref73] Shimizu Y.; Shoji Y.; Hashizume D.; Nagata Y.; Fukushima T. Sensing the Chirality of Various Organic Solvents by Helically Arranged π-Blades. Chem. Commun. 2018, 54, 12314–12317. 10.1039/C8CC06277C. 30221285

[ref74] Cornia A.; Mannini M.; Sessoli R.; Gatteschi D. Propeller-Shaped Fe4 and Fe3M Molecular Nanomagnets: A Journey from Crystals to Addressable Single Molecules. Eur. J. Inorg. Chem. 2019, 552–568. 10.1002/ejic.201801266.

[ref75] Zhu Y.-Y.; Guo X.; Cui C.; Wang B.-W.; Wang Z.-M.; Gao S. An Enantiopure FeIII4 Single-Molecule Magnet. Chem. Commun. 2011, 47, 8049–8051. 10.1039/c1cc12831k. 21677989

[ref76] Ninova S.; Lanzilotto V.; Malavolti L.; Rigamonti L.; Cortigiani B.; Mannini M.; Totti F.; Sessoli R. Valence Electronic Structure of Sublimated Fe4 Single-Molecule Magnets: An Experimental and Theoretical Characterization. J. Mater. Chem. C 2014, 2, 9599–9608. 10.1039/c4tc01647e.

[ref77] Katoono R.; Kawai H.; Fujiwara K.; Suzuki T. Dynamic Molecular Propeller: Supramolecular Chirality Sensing by Enhanced Chiroptical Response through the Transmission of Point Chirality to Mobile Helicity. J. Am. Chem. Soc. 2009, 131, 16896–16904. 10.1021/ja906810b. 19874032

[ref78] Martinez A.; Guy L.; Dutasta J. P. Reversible, Solvent-Induced Chirality Switch in Atrane Structure: Control of the Unidirectional Motion of the Molecular Propeller. J. Am. Chem. Soc. 2010, 132, 16733–16734. 10.1021/ja102873x. 20536136

[ref79] Flack H. D. On Enantiomorph-polarity Estimation. Acta Crystallogr. Sect. A 1983, 39, 876–881. 10.1107/S0108767383001762.

[ref80] Watkin D. J.; Cooper R. I. Howard Flack and the Flack Parameter. Chemistry 2020, 2, 796–804. 10.3390/chemistry2040052.

[ref81] Coles S. J.; Gale P. A. Changing and Challenging Times for Service Crystallography. Chem. Sci. 2012, 3, 683–689. 10.1039/c2sc00955b.

