# Supplementary material for: Chiral Co3Y Propeller-Shaped Chemosensory Platforms Based on 19F-NMR
Source: Inorg Chem. 2023 Jan 30;62(6):2680–93. doi: 10.1021/acs.inorgchem.2c03737 (PMC9930122; doi:10.1021/acs.inorgchem.2c03737)
Supplement: Supplementary file 1 — ic2c03737_si_001.pdf [file ic2c03737_si_001.pdf]

Chiral Co_3Y propeller-shaped chemosensory platforms based on ^{19}F-NMR

Gabrielle Audsley,[a] Harry Carpenter,[a] Nsikak B. Essien,[a] James Lai-Morrice,[a] Youssra Al-Hilaly,[b] Louise C. Serpell,[b] Geoffrey R. Akien,[c] Graham J. Tizzard,[d] Simon J. Coles,[d] Cristina Pubill Ulldemolins[a*±] and George E. Kostakis[a*]

[a]Department of Chemistry, School of Life Sciences, University of Sussex, Brighton BN1 9QJ, UK. E-mail: G.Kostakis@sussex.ac.uk, C.Pubill-Ulldemolins@sussex.ac.uk

[b]Sussex Neuroscience, School of Life Sciences, University of Sussex, Brighton BN1 9QG, UK

[c]Department of Chemistry, Lancaster University, Lancaster LA1 4YB, UK

[d]UK National Crystallography Service, Chemistry, University of Southampton, Southampton SO1 71BJ, UK

± Current address : Department of Nutrition, Food Sciences and Gastronomy, Faculty of Pharmacy and Nutrition, University of Barcelona, Barcelona, email: cristina.pubill@ub.edu

Contents

Materials. 2
Instrumentation. 2
Ligand Synthesis 3
Complex Synthesis 4
Single-crystal X-ray Diffraction studies. 8
NMR Characterisation in solution. 12
Thermogravimetric analysis 14
UV-Vis in solution 15
^{19}F-NMR studies 16
Additional synthetic studies 21
Computational Studies 25
Computational details 25
Computational studies on the Rh complexes with the different auxiliaries, as reported by Bian and Song in 2019.[12] 26
Computational studies on the simplified $CoYL_2$ model system with the different analytes reported in this work. 29
Computed NMR values for Co_3YL_6 with the different auxiliaries reported in this work. 31
Molecular structures, cartesian coordinates and corresponding energies for all the computed species. 34
References 125

Materials.

All reagents were purchased from Sigma Aldrich, Fluorochem, Tokyo Chemical Industry, Apollo Scientific, Fischer Scientific or Alfa Aesar and used without further purification. Experiments were performed under aerobic conditions.

Instrumentation.

NMR spectra were recorded with a Varian VNMRS 400 at 25 °C, at either 600 MHz or 151 MHz in Chloroform-d or DMSO-d_6. ^{19}F-NMR data were recorded using a VNMR400 at 30 °C on solution-state samples in either $CDCl_3$ or CD_3OD. Chemical shifts are quoted in parts per million (ppm). Coupling constants (J) are recorded in units of Hz. FT-IR spectra were recorded over 4000–650 cm^{-1} on a PerkinElmer Spectrum One FT-IR spectrometer fitted with a UATR polarisation accessory. HRMS data were obtained with a Bruker Daltonics Fourier Transform (FTMS) Apex II spectrometer with electrospray ionisation (ESI) and methanol as solvent. HR-MS data were obtained on a VG Autospec Fissions instrument (EI at 70 eV). Molecular ions are reported as mass/charge (m/z) ratios. Circular dichroism measurements were carried out with a JASCO J-715 CD on a 1 mM sample dissolved in acetonitrile between 180 – 750nm. Thermogravimetric analysis was carried out with a Thermogravimetric analyser Q-50 V20.13 using a platinum pan in an N_2 atmosphere from 25 – 1000 °C, at a scan rate of 5 °C/min. UV−Vis measurements (280-750 nm) were performed at room temperature (15-20˚C) using a Thermo Scientific Evolution 300 UV-Vis spectrophotometer equipped with 5mm path length quartz cells, and the collected data were processed using the Vision Pro software. Elemental analysis was carried out at the Institute of Inorganic Chemistry, Karlsruhe Institute of Technology, Germany.

Ligand Synthesis

F-substituted salicylaldehyde (5 mmol) and amino alcohol (5 mmol) were added to methanol (20 mL). The solution was stirred and refluxed at 70 °C for 3 hours, then cooled, filtered and evaporated in vacuo to give the final product. (Scheme S1 & Table S1)

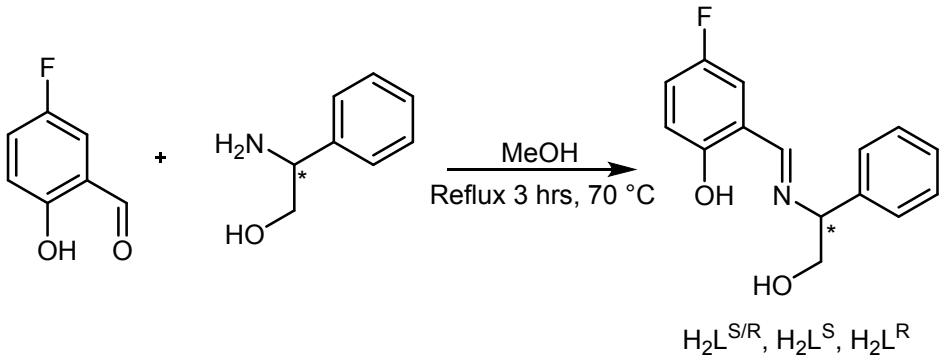

Scheme S1. Ligand synthesis.

Table S1. Ligand synthesis details

Aim	Solvent	Volume(mL)	Conc.(M)	Yield (%)	Comments
**$H_2L^{S/R}$**	MeOH	20	0.495	95	Yellow powdery solid
**H_2L^{S}**	MeOH	20	0.495	97	Yellow powdery solid
**H_2L^{R}**	MeOH	20	0.495	100	Yellow powdery solid

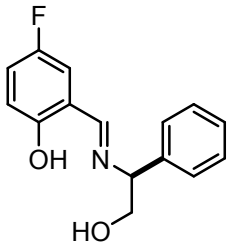

H_2L^{S} Yield: 97% (5 mmol scale – 1.944 g of yellow powder/solid). EI-MS: calcd (MH+ = $C_{15}H_{15}NO_2F$+), 260.2829 Da; found (MH+), m/z 260.. ^{1}H NMR (600 MHz, $CDCl_3$) δ 13.05 (s, 1H), 8.41 (s, 1H), 7.37 (d, *J* = 4.4 Hz, 3H), 7.37 – 7.26 (m, 1H), 7.04 (td, *J* = 8.7, 3.1 Hz, 1H), 6.97 (dd, *J* = 8.3, 3.1 Hz, 1H), 6.92 (dd, *J* = 9.0, 4.4 Hz, 1H), 4.48 (t, *J* = 6.5 Hz, 1H), 3.97 – 3.89 (m, 2H).^{19}F NMR (376 MHz, $CDCl_3$) δ -116.90, -123.54, -125.53, -125.55, -125.57, -125.58, -125.60.

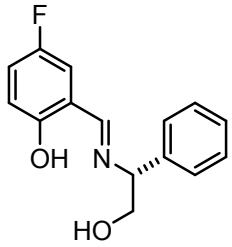

H_2L^{R} Yield: 100% (5 mmol scale – 2.061 g of yellow powder/solid). EI-MS: calcd (MH+ = $C_{15}H_{15}NO_2F$+), 260.2829 Da; found (MH+), m/z 260. ^{1}H NMR (600 MHz, cdcl$_3$) δ δ 13.09 (s, 1H), 8.40 (s, 1H), 7.37 (d, *J* = 4.4 Hz, 4H), 7.31 (h, *J* = 4.1 Hz, 1H), 7.04 (ddd, *J* = 9.1, 8.1, 3.1 Hz, 1H), 6.95 (dd, *J* = 8.3, 3.1 Hz, 1H), 6.91 (dd, *J* = 9.0, 4.4 Hz, 1H), 4.47 (dd, *J* = 7.2, 5.8 Hz, 1H), 3.96 – 3.88 (m, 2H)). ^{19}F NMR (376 MHz, cdcl$_3$) δ -116.93, -123.56, -125.56, -125.57, -125.58, -125.59, -125.60, -125.61.

Complex Synthesis

The ligand (0.3 mmol) and Et_3N (0.5 mmol) were added to solvent (10 mL). The solution was heated to 70 °C whilst stirring for 15 minutes. Y(salt) (0.1 mmol) and Co(salt) (0.2 mmol) were added simultaneously, and the mixture was then stirred and refluxed at 70 °C for a further 3 hours. The solution was left to cool to room temperature before being filtered. The product was then obtained by slow evaporation after 1 week. (Scheme S2)

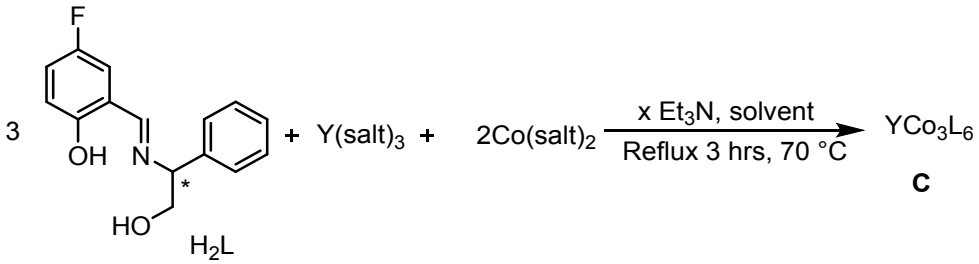

Scheme S2. Complex synthesis scheme. H_2**L** = ligand and **C** = complex with para-substituted F.

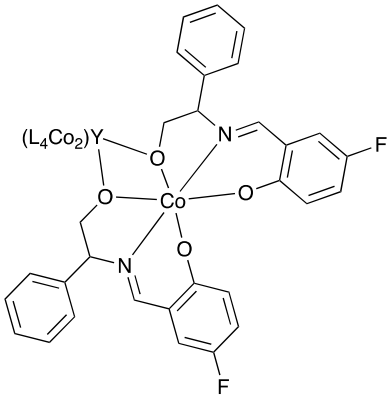

C^{S} Yield: 36% (3 mmol scale – 44 mg of purple/black crystals). ^{1}H NMR (399 MHz, $CDCl_3$) δ 7.76 (d, *J* = 2.0 Hz, 1H), 7.34 – 7.18 (m, 2H), 7.09 (dtd, *J* = 12.1, 9.2, 3.8 Hz, 2H), 6.91 (d, *J* = 7.2 Hz, 1H), 6.86 (dd, *J* = 8.4, 3.0 Hz, 1H), 5.24 (ddd, *J* = 9.9, 6.4, 2.0 Hz, 1H), 3.68 (s, 1H), 3.44 (t, *J* = 10.4 Hz, 1H), 2.88 (dd, *J* = 10.8, 6.5 Hz, 1H). ^{19}F NMR (376 MHz, $CDCl_3$) δ -130.06, -130.07, -130.08, -130.09, -130.10, -130.11. Elemental analysis (%) calcd (found) for $C_{100.66}Co_3F_6H_{87.99}N_{11.33}O_{12}Y$: C, 59.61 (57.63); H,4.37(3.65); N, 7.82 (6.04). The values of the elemental analysis match formula of $C_{90}Co_3F_6H_{72}N_6O_{12}Y(CH_3CN)_3(H_2O)_3$ calc C, 58.04; H,4.41; N, 6.35, indicating that the targeted compound loses lattice CH_3CN molecules and is susceptible to moisture.

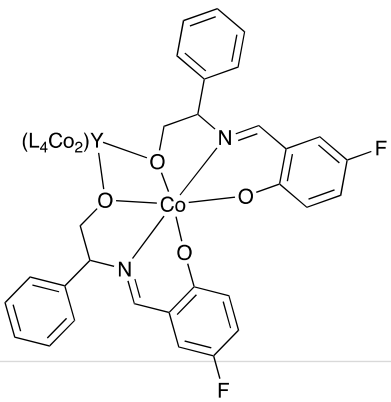

C^{R} Yield: 32% (3 mmol scale – 39 mg of purple/black crystals). ^{1}H NMR (399 MHz, $CDCl_3$) δ 7.76 (s, 1H), 7.32 – 7.19 (m, 2H), 7.16 – 7.03 (m, 2H), 6.92 (s, 1H), 6.92 – 6.83 (m, 2H), 5.24 (t, *J* = 8.2 Hz, 1H), 3.68 (s, 1H), 3.44 (t, *J* = 10.4 Hz, 1H), 2.88 (dd, *J* = 10.7, 6.5 Hz, 1H). ^{19}F NMR (376 MHz, $CDCl_3$) δ -130.06, -130.07, -130.08, -130.09, -130.10, -130.11. Elemental analysis (%) calcd

(found) for $C_{101}Co_3F_6H_{88.5}N_{11.5}O_{12}Y$: C, 59.61 (57.33); H,4.38(3.48); N, 7.91 (5.49). The values of the elemental analysis match formula of $C_{90}Co_3F_6H_{72}N_6O_{12}Y(CH_3CN)_2(H_2O)_4$ calc C, 57.50; H,4.41; N, 5.71, indicating that the targeted compound loses lattice CH_3CN molecules and is susceptible to moisture.

ESI-MS data

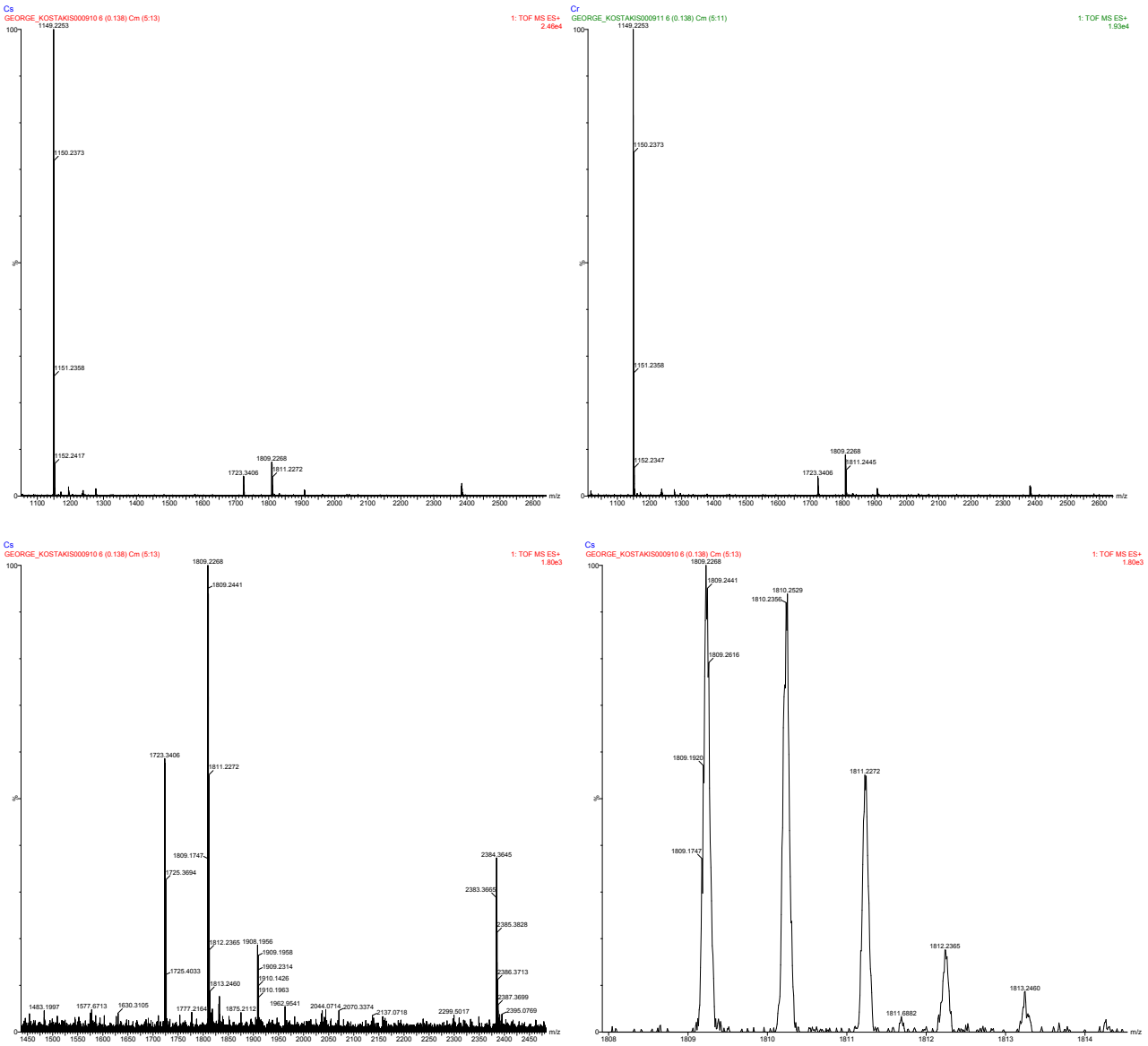

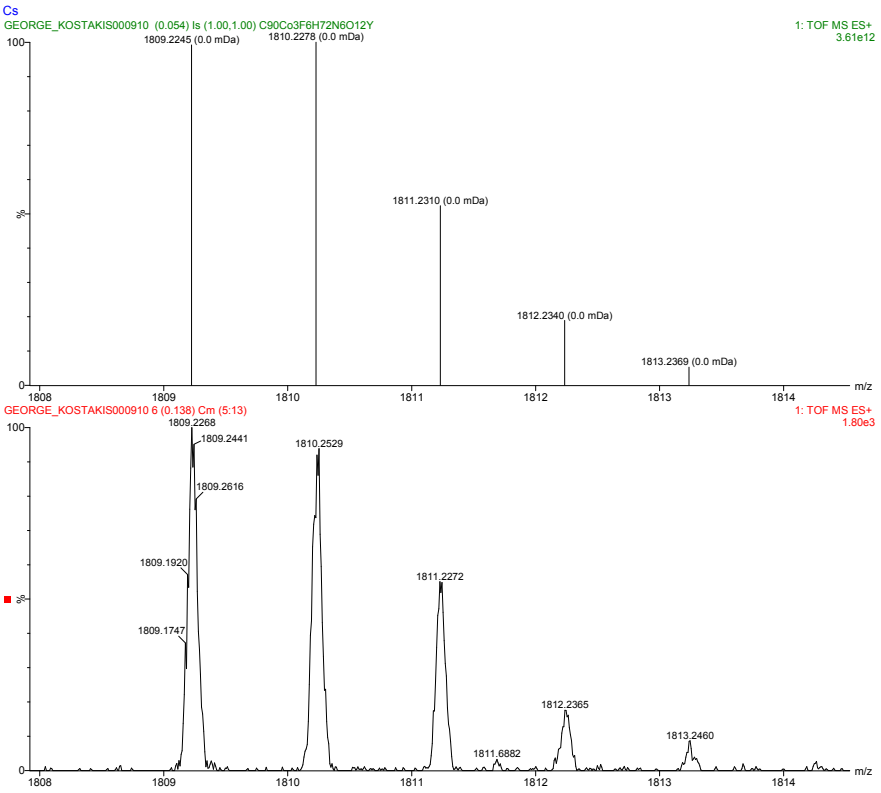

Elemental Composition Report

Single Mass Analysis
Tolerance = 100.0 PPM / DBE: min = -10.0, max = 100.0
Element prediction: Off

Monoisotopic Mass, Odd and Even Electron Ions
28 formula(e) evaluated with 1 results within limits (up to 50 closest results for each mass)
Elements Used:
C: 90-90 H: 0-1000 N: 6-6 O: 12-12 F: 6-6 Si: 0-1 P: 0-1 S: 0-6 Co: 3-3 Y: 1-1

Minimum:				-10.0	
Maximum:		5.0	100.0	100.0	
Mass	Calc. Mass	mDa	PPM		DBE Formula
1809.2268	1809.2245	2.3	1.3	55.0	C90 H73 N6 O12 F6 Co3 Y

Table S2. Screening experiments for complex synthesis varying concentration, salts, and solvents for optimum reaction conditions. The yields are calculated based on Co and based on obtaining a $[Co_3YL_6]$ species.

Aim	Salts (Y and Co)	Solvent	Volume (mL)	Conc. (M)	Yield (%)
**C**	Cl^-	EtOH	20	0.043	12
**C**	Cl^-	EtOH	10	0.171	14
**C**	Cl^-	MeCN	10	0.192	17
**C**	Cl^-	MeOH	10	0.495	11
**C**	Cl^-	EtOAc	10	0.1025	2
**C**	Cl^-	$CHCl_3$	10	0.125	n/a
$\mathbf{C^S}$	Cl^-	MeCN	10	0.192	18
$\mathbf{C^R}$	Cl^-	MeCN	10	0.192	18
$\mathbf{C^S}$	NO_3^-	MeCN	10	0.192	36
$\mathbf{C^R}$	NO_3^-	MeCN	10	0.192	32
**C**	NO_3^-	EtOH	10	0.171	5
**C**	Cl^-	MeCN	5	0.767	n/a
$\mathbf{C^S}$	NO_3^-	MeCN	5	0.767	n/a

Single-crystal X-ray Diffraction studies.

A "What is this" measurement at the University of Sussex in a Rigaku rotating anode diffractometer ran for both crystals obtained from EtOH to identify the components for the synthetic screening experiments. EtOH lattice molecules could be identified in both cases, but most importantly, one non-coordinating ligand molecule could be identified in the lattice (Figure S1); therefore, we did not proceed with complete data collection.

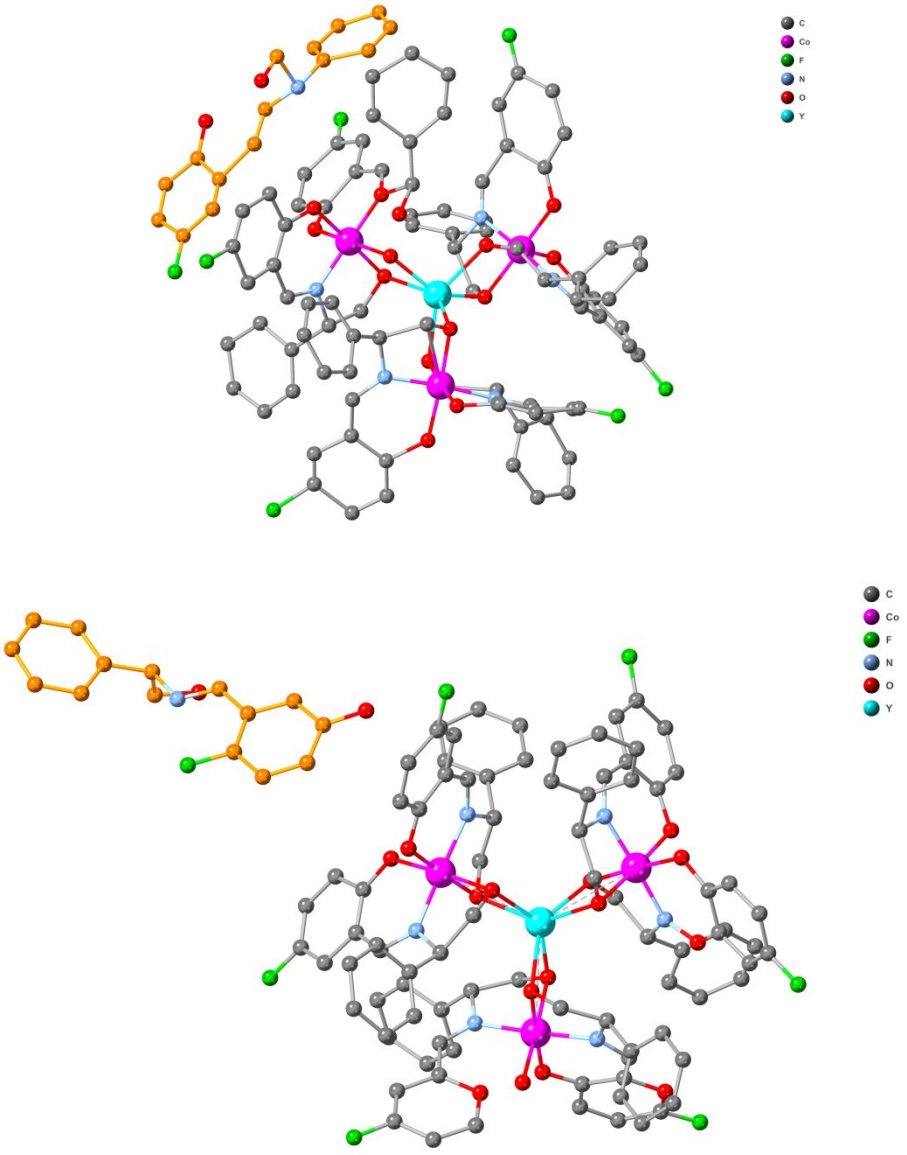

Figure S1. A picture of the crystallised products obtained in ethanol GA005_EtOH_S (upper) and GA010_EtOH_R (lower)

Data for **C^{R}** and **C^{S}** were collected at the National Crystallography Service, University of Southampton.[1] For **C^{R}**, a suitable orange block-shaped crystal (0.13 × 0.1 × 0.02) mm^3 was selected and for **C^{S}** a suitable orange block-shaped crystal (0.08 × 0.06 × 0.025) mm^3 was selected. Both crystals were mounted on a MITIGEN holder in perfluoro ether oil on a Rigaku FRE+ diffractometer equipped with arc)sec VHF Varimax confocal mirrors, an AFC12 goniometer and HyPix 6000 detector and kept at 100(2) K during data collection. For **C^{R}(new dataset)** a suitable brown block-shaped crystal (0.2 × 0.15 × 0.06) mm^3 was selected and mounted on a MITIGEN holder in perfluoro ether oil on a Rigaku 007HF diffractometer equipped with HF Varimax confocal mirrors, a UG2 goniometer and HyPix 6000 detector and kept at 100(2) K during data collection. The data were processed with CrysAlisPro and solved by intrinsic phasing methods with SHELXT[2] using Olex2.[3] All crystal structures were then refined on F_o^2 by full-matrix least-squares refinements using SHELXL.[2] All structures were modelled as inversion twins to obtain accurate Flack parameters. A solvent mask was applied to each structure to eliminate electronic contribution from disordered solvent MeCN. Thermal restarints were applied to one of the phenyl rings of **C^{R}** and **C^{S}** and the equivalent phenyl ring of **C^{R}(new dataset)** was modelled with disorder (~71:29) and thermal and geometric restraints were applied to both disorder components. Geometric/crystallographic calculations were performed using Olex2[3]. Images and video generated using CrystalMaker®: a crystal and molecular structures program for Mac and Windows. CrystalMaker Software Ltd, Oxford, England (www.crystalmaker.com). Structures have been given CCDC deposition numbers 2195856 – 2195858.

Table S3 Crystal data and structure refinement for **C^R**, **C^S** and **C^R(new dataset)**

Identification code	**C^R[5.5MeCN]**	**C^S[5.25MeCN]**	**C^R[4.25MeCN] (new dataset)**
Empirical formula	$C_{101}Co_3F_6H_{88.5}N_{11.5}O_{12}Y$	$C_{100.5}Co_3F_6H_{84.75}N_{11.25}O_{12}Y$	$C_{98.5}Co_3F_6H_{84.75}N_{10.25}O_{12}Y$
Formula weight	2035.03	2021.74	1983.71
Temperature/K	100(2)	100(2)	100(2)
Crystal system	cubic	cubic	cubic
Space group	$P2_13$	$P2_13$	$P2_13$
a/Å	21.4078(4)	21.39580(10)	21.3614(2)
b/Å	21.4078(4)	21.39580(10)	21.3614(2)
c/Å	21.4078(4)	21.39580(10)	21.3614(2)
α/°	90	90	90
β/°	90	90	90
γ/°	90	90	90
Volume/Å^3	9811.1(5)	9794.57(14)	9747.4(3)
Z	4	4	4
ρ_{calc}g/cm^3	1.378	1.371	1.352
μ/mm^{-1}	1.161	1.162	5.327
F(000)	4180.0	4146.0	4048.0
Crystal size/mm^3	0.13 × 0.1 × 0.02	0.08 × 0.06 × 0.025	0.19 × 0.15 × 0.06
Radiation	Mo Kα (λ = 0.71075)	Mo Kα (λ = 0.71075)	Cu Kα (λ = 1.54178)
2Θ range for data collection/°	3.296 to 51.342	3.298 to 57.366	5.85 to 153
Index ranges	-26 ≤ h ≤ 17, -23 ≤ k ≤ 23, -23 ≤ l ≤ 22	-28 ≤ h ≤ 28, -28 ≤ k ≤ 27, -28 ≤ l ≤ 28	-26 ≤ h ≤ 25, -20 ≤ k ≤ 23, -21 ≤ l ≤ 23
Reflections collected	21222	141527	17470
Independent reflections	5980 [R_{int} = 0.0527, R_{sigma} = 0.0572]	8441 [R_{int} = 0.0477, R_{sigma} = 0.0205]	[R_{int} = 0.0230, R_{sigma} = 0.0201]
Data/restraints/parameters	5980/84/356	8441/84/356	6300/303/387
Goodness-of-fit on F^2	1.038	1.035	1.026
Final R indexes [I>=2σ (I)]	R_1 = 0.0543, wR_2 = 0.1268	R_1 = 0.0491, wR_2 = 0.1358	R_1 = 0.0703, wR_2 = 0.2022
Final R indexes [all data]	R_1 = 0.0794, wR_2 = 0.1368	R_1 = 0.0607, wR_2 = 0.1432	R_1 = 0.0762, wR_2 = 0.2095
Largest diff. peak/hole / e Å^{-3}	0.252/-0.653	0.447/-0.984	0.503/-1.274
Flack parameter	0.131(13)	0.210(10)	0.071(8)
Hooft Parameter	0.031(4)	0.0260(11)	-0.0373(12)
CCDC dep. No.	2195856	2195857	2195858

Table S4. Selected bond lengths for C^R and C^S

Atom	Atom	Length/Å	Atom	Atom	Length/Å
		C^R			
Y1	$O2^1$	2.191(4)	Co1	O1	1.891(5)
Y1	O2	2.191(4)	Co1	O2	1.913(4)
Y1	$O2^2$	2.191(4)	Co1	O3	1.896(5)
Y1	O4	2.203(4)	Co1	O4	1.909(4)
Y1	$O4^1$	2.203(4)	Co1	N1	1.910(6)
Y1	$O4^2$	2.203(4)	Co1	N2	1.915(6)
Y1	$Co1^1$	3.2095(9)	Y1	$Co1^2$	3.2094(9)
Y1	Co1	3.2094(9)			
		^{1}3/2-Y,2-Z,1/2+X; ^{2}-1/2+Z,3/2-X,2-Y			
		C^S			
Y1	$O2^2$	2.170(3)	Co1	O2	1.906(3)
Y1	O2	2.170(3)	Co1	O3	1.894(4)
Y1	$O2^1$	2.170(3)	Co1	O4	1.892(4)
Y1	$O006^2$	2.171(3)	Co1	O006	1.907(3)
Y1	O006	2.171(3)	Co1	N1	1.907(4)
Y1	$O006^1$	2.171(3)	Co1	N2	1.913(4)
Y1	$Co1^1$	3.1849(6)	Y1	Co1	3.1849(6)
Y1	$Co1^2$	3.1849(6)			
		^{1}3/2-Y,2-Z,1/2+X; ^{2}-1/2+Z,3/2-X,2-Y			

NMR Characterisation in solution.

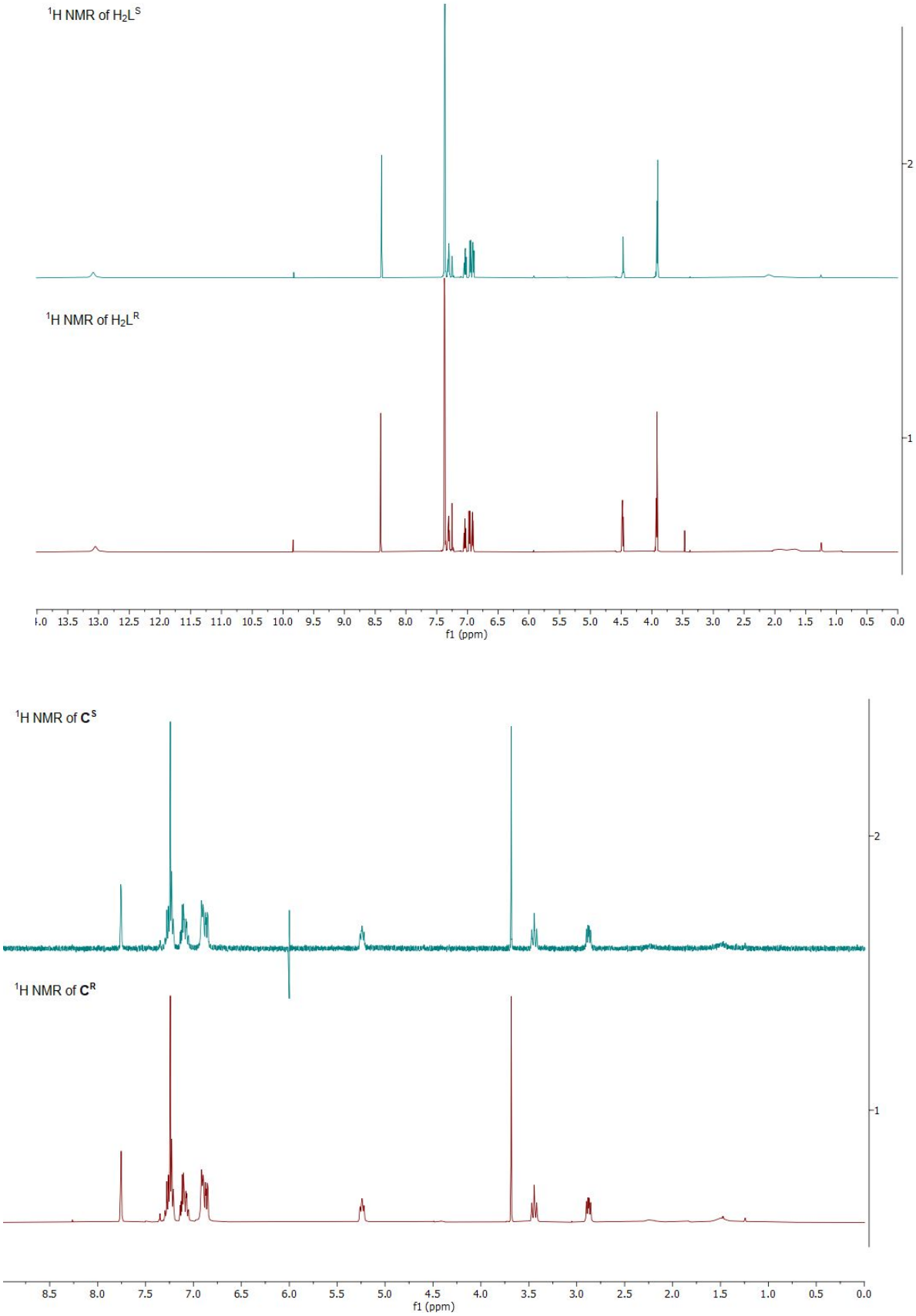

Figure S2. Overlayed ^{1}H NMR of H_2**L** (top) vs **C** (bottom) in $CDCl_3$; red = *R*- derivative and blue = *S*-derivative.

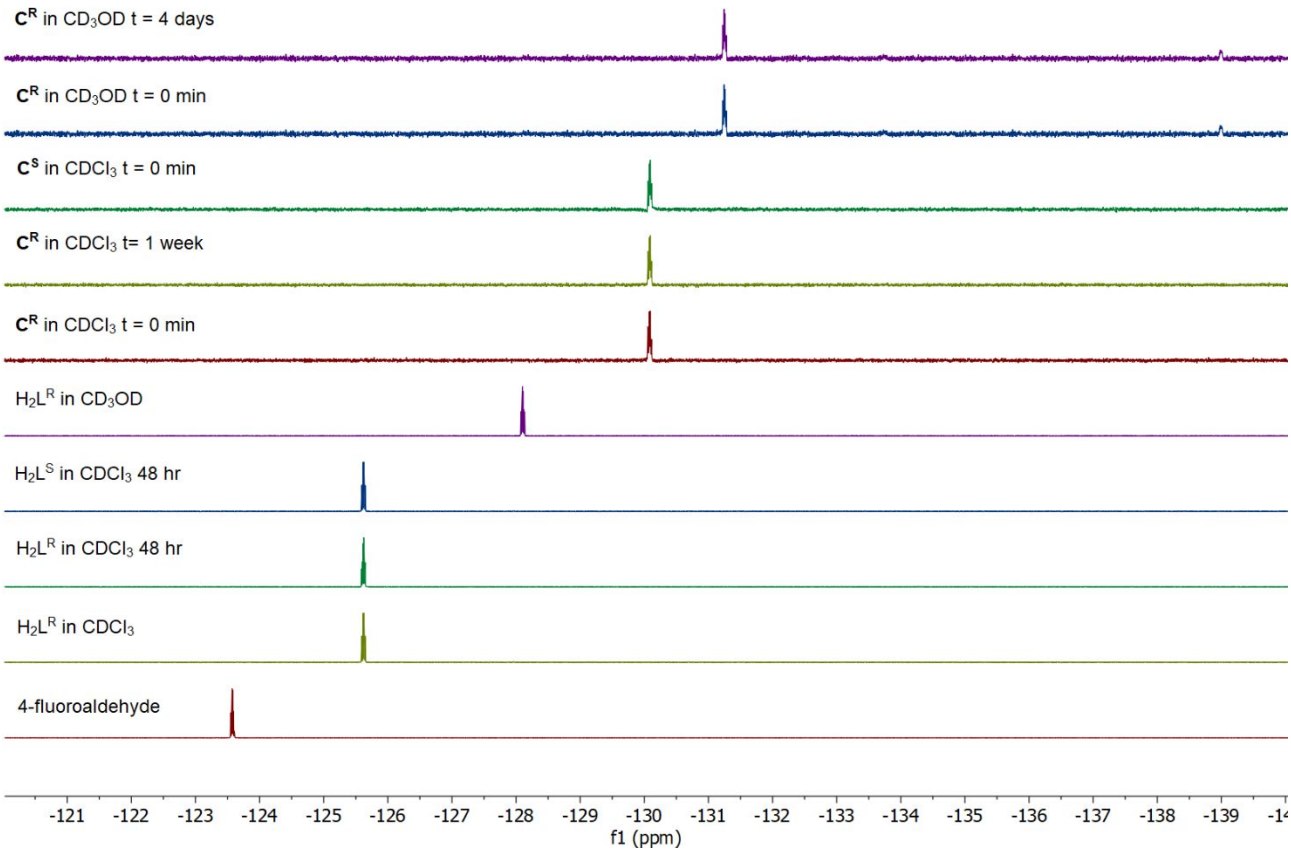

Figure S3. Overlayed ^{19}F NMR spectra of starting material, ligand and complexes in various solvents and periods.

Thermogravimetric analysis

TGA was carried out with a Thermogravimetric analyser Q50 V20.13 using a platinum pan. Thermal analysis (20-1000 °C) under N_2 atmosphere (10 mL per minute)

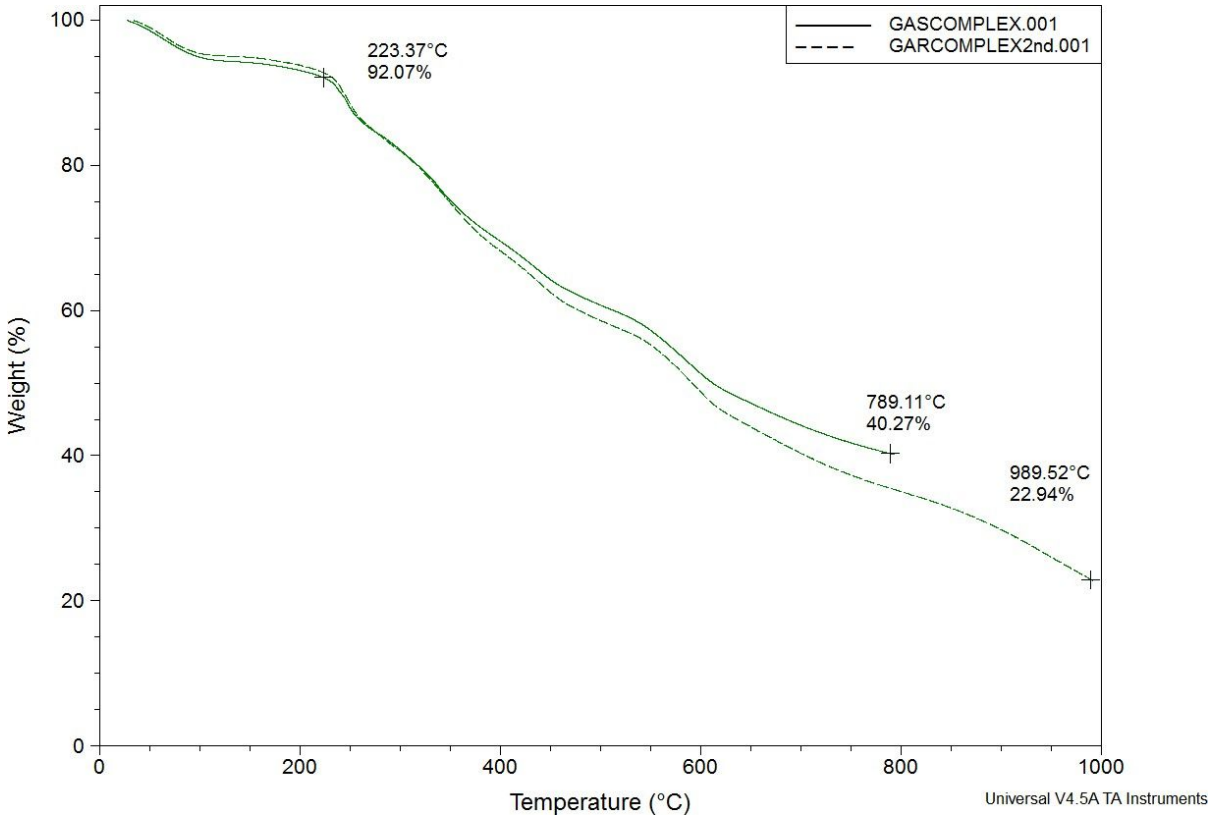

Figure S4. Thermogravimetric analysis of $\mathbf{C^S}$ (solid line) and $\mathbf{C^R}$ (dashed line).

UV-Vis in solution

Due to high concentration, UV/Vis data is presented from 550 nm to 900 nm and demonstrates a broad peak characteristic of a Co(III) d^6 electronic configuration.

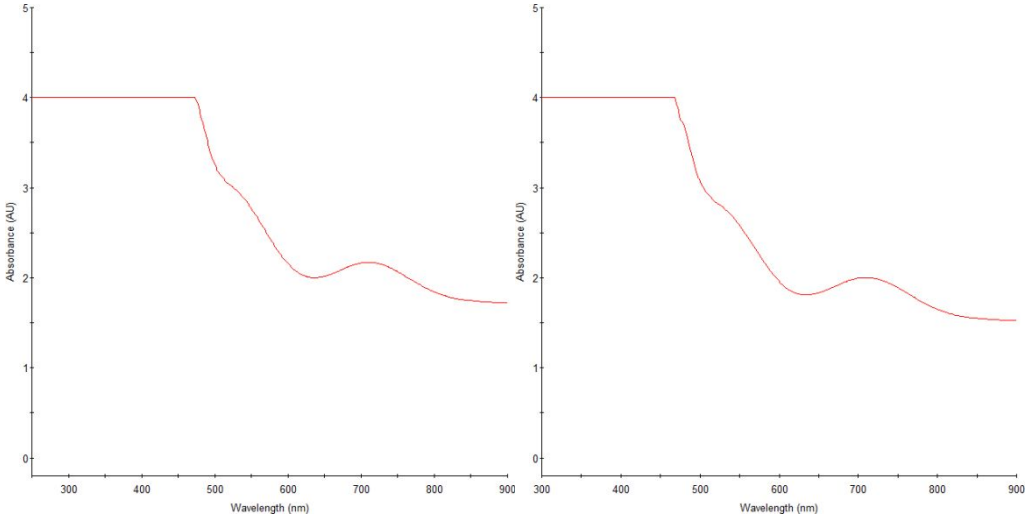

Figure S5. UV/Vis spectra for $\mathbf{C^S}$ (left) versus $\mathbf{C^R}$ (right) from 550-900 nm.

^{19}F-NMR studies

Table S5. Samples (1 mM) of $\mathbf{C^S}$ and $\mathbf{C^R}$ in $CDCl_3$ demonstrate no changes in peaks over time and corresponding ^{19}F NMR spectra in the table.

Complex	Solvent	Time / hr	Scans	Complex peak	Upfield peak
$\mathbf{C^S}$	$CDCl_3$	0	64	-130.06	
$\mathbf{C^S}$	$CDCl_3$	168	64	-130.06	
$\mathbf{C^S}$	$CDCl_3$	720	64	-130.06	
$\mathbf{C^R}$	$CDCl_3$	0	64	-130.06	
$\mathbf{C^R}$	$CDCl_3$	168	64	-130.06	
$\mathbf{C^R}$	$CDCl_3$	720	64	-130.06	

Table S6. Samples (1 mM) of $\mathbf{C^S}$ and $\mathbf{C^R}$ in CD_3OD demonstrate no changes in peaks over time and corresponding ^{19}F NMR spectra in the table.

Complex	Solvent	Time / hr	Scans	Complex peak	Upfield peak
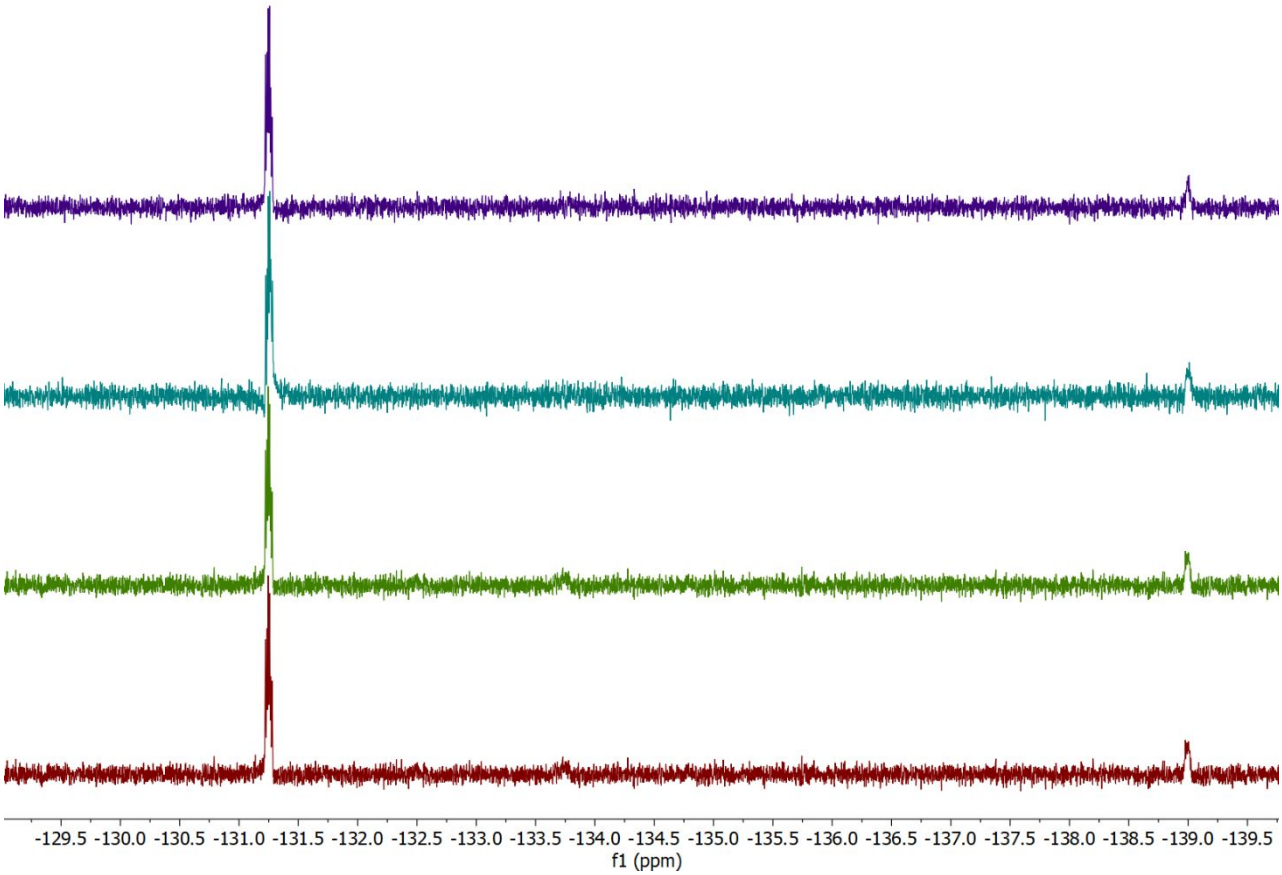					
$\mathbf{C^S}$	CD_3OD	0	64	-131.22	-138.99
$\mathbf{C^S}$	CD_3OD	96	64	-131.22	-139.00
$\mathbf{C^R}$	CD_3OD	0	64	-131.22	-138.99
$\mathbf{C^R}$	CD_3OD	96	64	-131.22	-138.99

Table S7. Results of NMR runs at both 64 and 128 scans at T = 0 and 30 °C to compare changes in intensity or chemical shift differences. The corresponding ^{19}F NMR spectra are given in the order of the table.

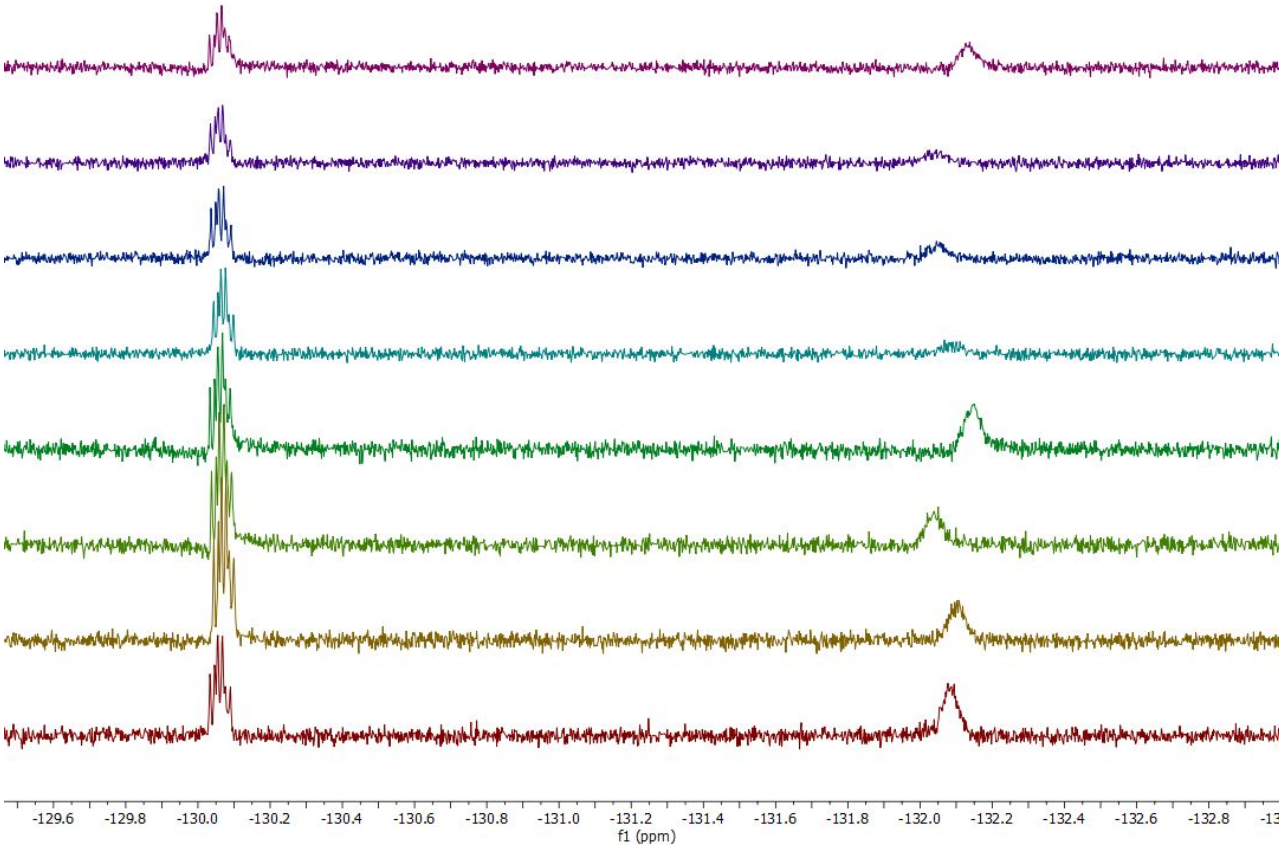

Complex	Amine	Ratio	Solvent	Time / hr	Complex peak	Complex+ Amine peak	Scans	Enantiomer difference
**C^S**	**$A1^S$**	1:20	$CDCl_3$	0	-130.03	-132.13	**64**	0.08
**C^S**	**$A1^R$**	1:20	$CDCl_3$	0	-130.03	-132.05	**64**	
**C^R**	**$A1^R$**	1:20	$CDCl_3$	0	-130.04	-132.05	**64**	0.04
**C^R**	**$A1^S$**	1:20	$CDCl_3$	0	-130.04	-132.09	**64**	
**C^S**	**$A1^S$**	1:20	$CDCl_3$	0	-130.03	-132.15	**128**	0.12
**C^S**	**$A1^R$**	1:20	$CDCl_3$	0	-130.03	-132.03	**128**	
**C^R**	**$A1^R$**	1:20	$CDCl_3$	0	-130.04	-132.11	**128**	0.02
**C^R**	**$A1^S$**	1:20	$CDCl_3$	0	-130.03	-132.08	**128**	

Table S8. Investigating solvent impact on analyte sensing; run at 64 scans and 30°C. The corresponding ^{19}F NMR spectra are given in the order of the table.

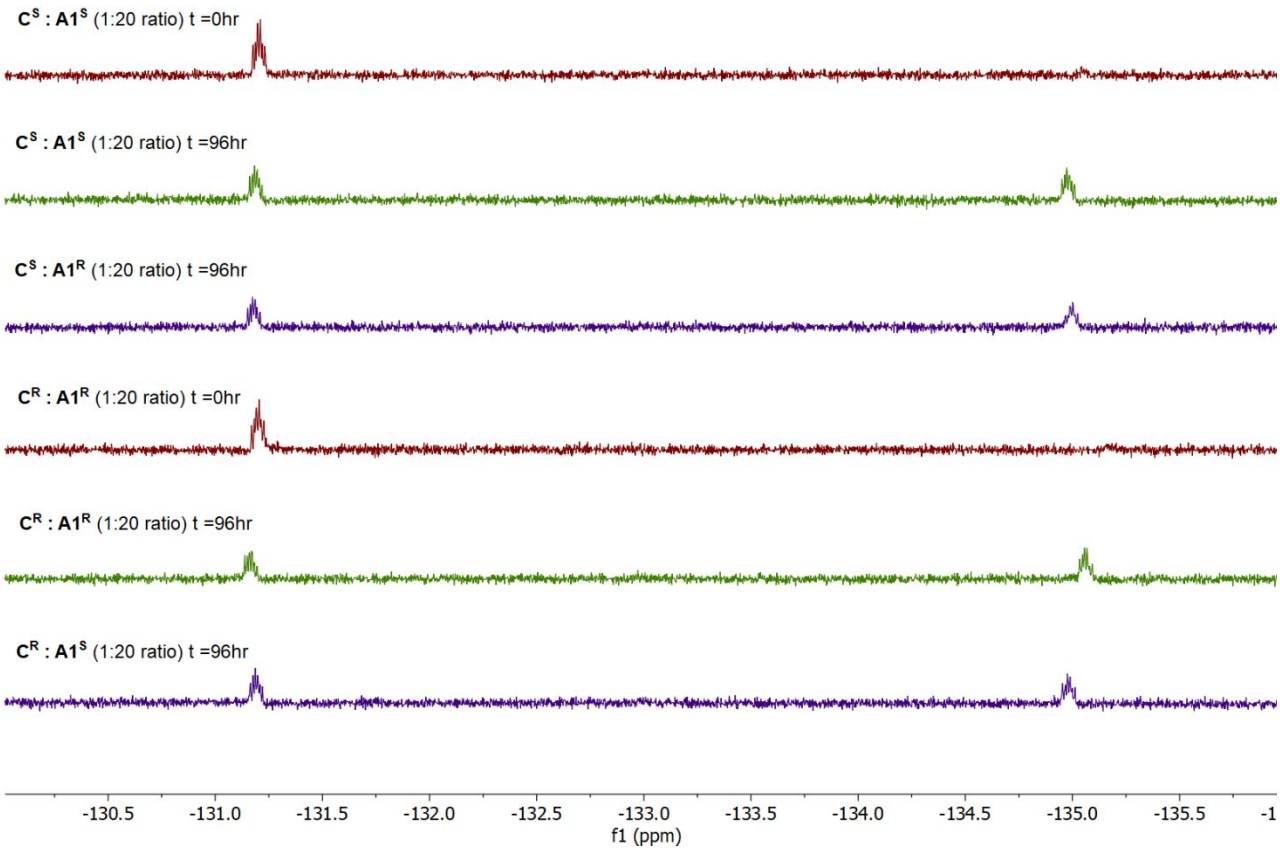

Complex	Amine	Ratio	Solvent	Time / hr	Complex peak	Amine peak	Enantiomer difference
$\mathbf{C^S}$	$\mathbf{A1^S}$	1:20	CD_3OD	0	-131.19		
$\mathbf{C^S}$	$\mathbf{A1^S}$	1:20	CD_3OD	96	-131.18	-134.97	0.0.3
$\mathbf{C^S}$	$\mathbf{A1^R}$	1:20	CD_3OD	96	-131.18	-135.00	
$\mathbf{C^R}$	$\mathbf{A1^R}$	1:20	CD_3OD	0	-131.20		
$\mathbf{C^R}$	$\mathbf{A1^R}$	1:20	CD_3OD	96	-131.16	-135.06	0.09
$\mathbf{C^R}$	$\mathbf{A1^S}$	1:20	CD_3OD	96	-131.19	-134.97	

Table S9. Results of increasing sensor:amine ratio to 1:50. The corresponding ^{19}F NMR spectra are given in the order of the table.

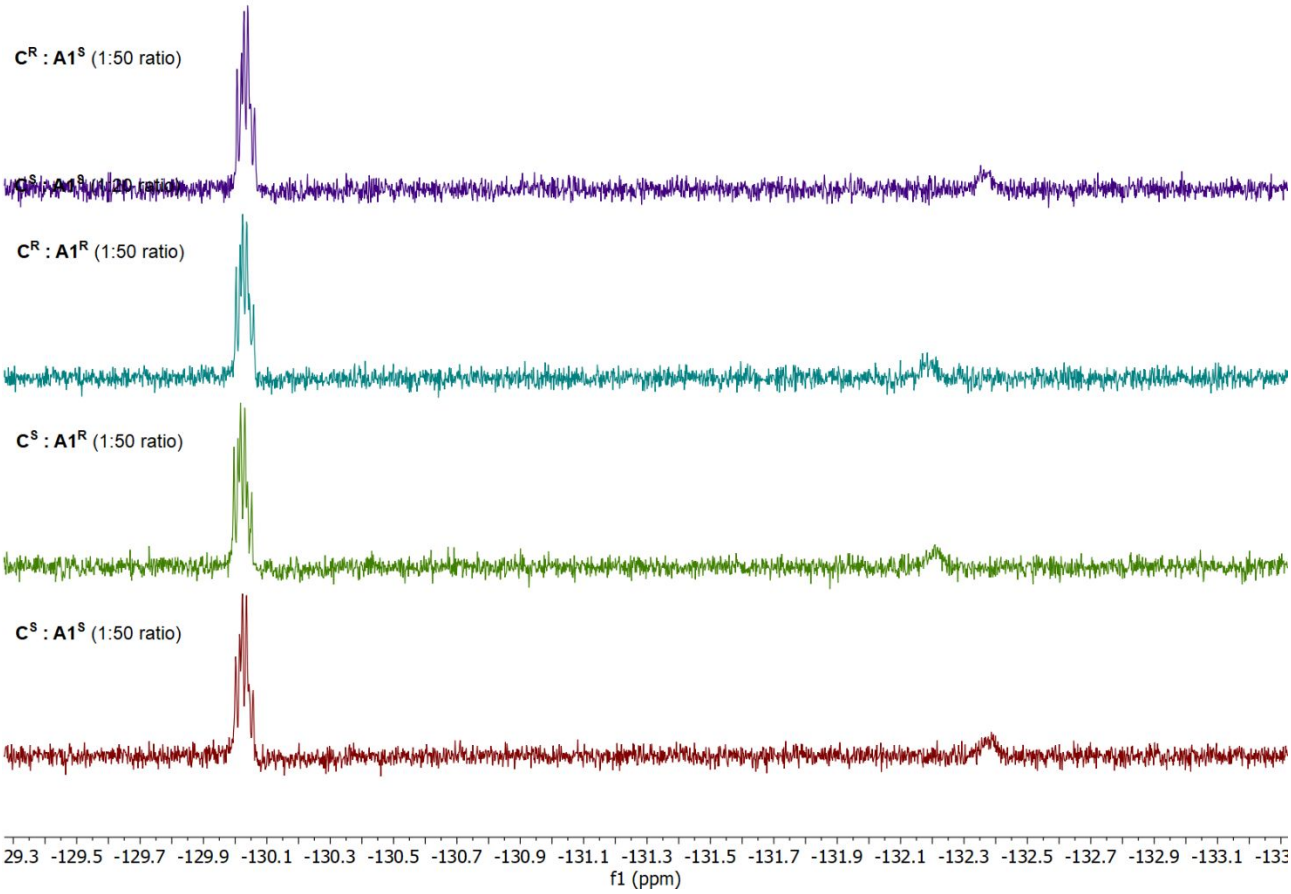

Complex	Amine	Ratio	Solvent	Time / hr	Complex peak	Amine peak	Enantiomer difference
C^S	$A1^S$	1:50	$CDCl_3$	0	-130.02	-132.37	0.18
C^S	$A1^R$	1:50	$CDCl_3$	0	-130.02	-132.19	
C^R	$A1^R$	1:50	$CDCl_3$	0	-130.02	-132.21	0.17
C^R	$A1^S$	1:50	$CDCl_3$	0	-130.02	-132.38	

Additional synthetic studies

Ligand synthesis and characterisation. 0.457g, 3.26mmol of 2-hydroxy-5-fluorobenzaldehyde was placed into a 100ml RB flask containing 10ml of methanol and stirred to obtain a clear solution before adding 0.254ml, 3.26mmol of L-Alaninol and reflux for three hours at 65°C, the solution was cooled and the volatiles removed and dried to obtain an oily compound.

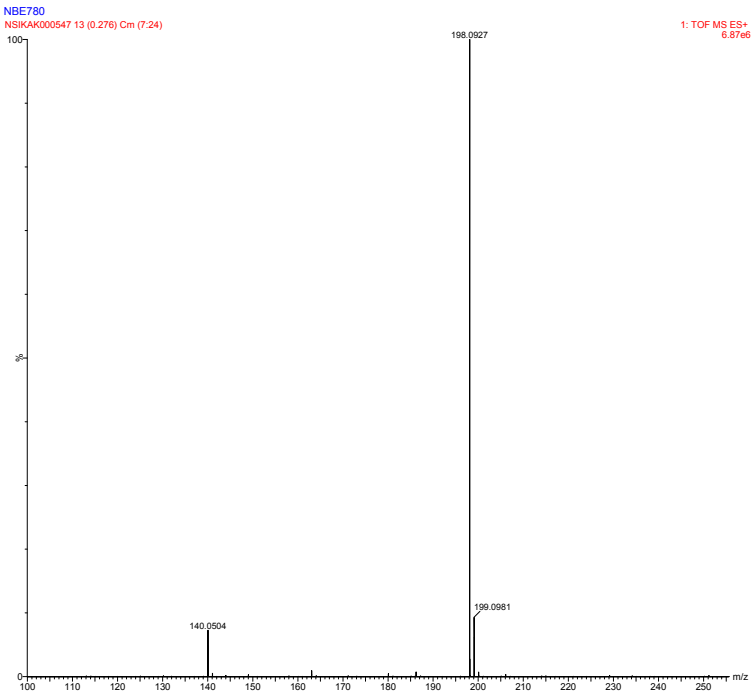

Elemental Composition Report
Single Mass Analysis
Tolerance = 100.0 PPM / DBE: min = -1.5, max = 50.0
Element prediction: Off
Number of isotope peaks used for i-FIT = 3
Monoisotopic Mass, Odd and Even Electron Ions
1 formula(e) evaluated with 1 results within limits (up to 50 closest results for each mass)
Elements Used:
C: 10-10 H: 0-1000 N: 1-1 O: 2-2 F: 1-1

Minimum:				-1.5				
Maximum:		5.0	100.0	50.0				
Mass	Calc. Mass	mDa	PPM	DBE	i-FIT	Norm	Conf(%)	Formula
198.0936	198.0930	0.6	3.0	4.5	1090.8	n/a	n/a	C10 H13 N O2 F

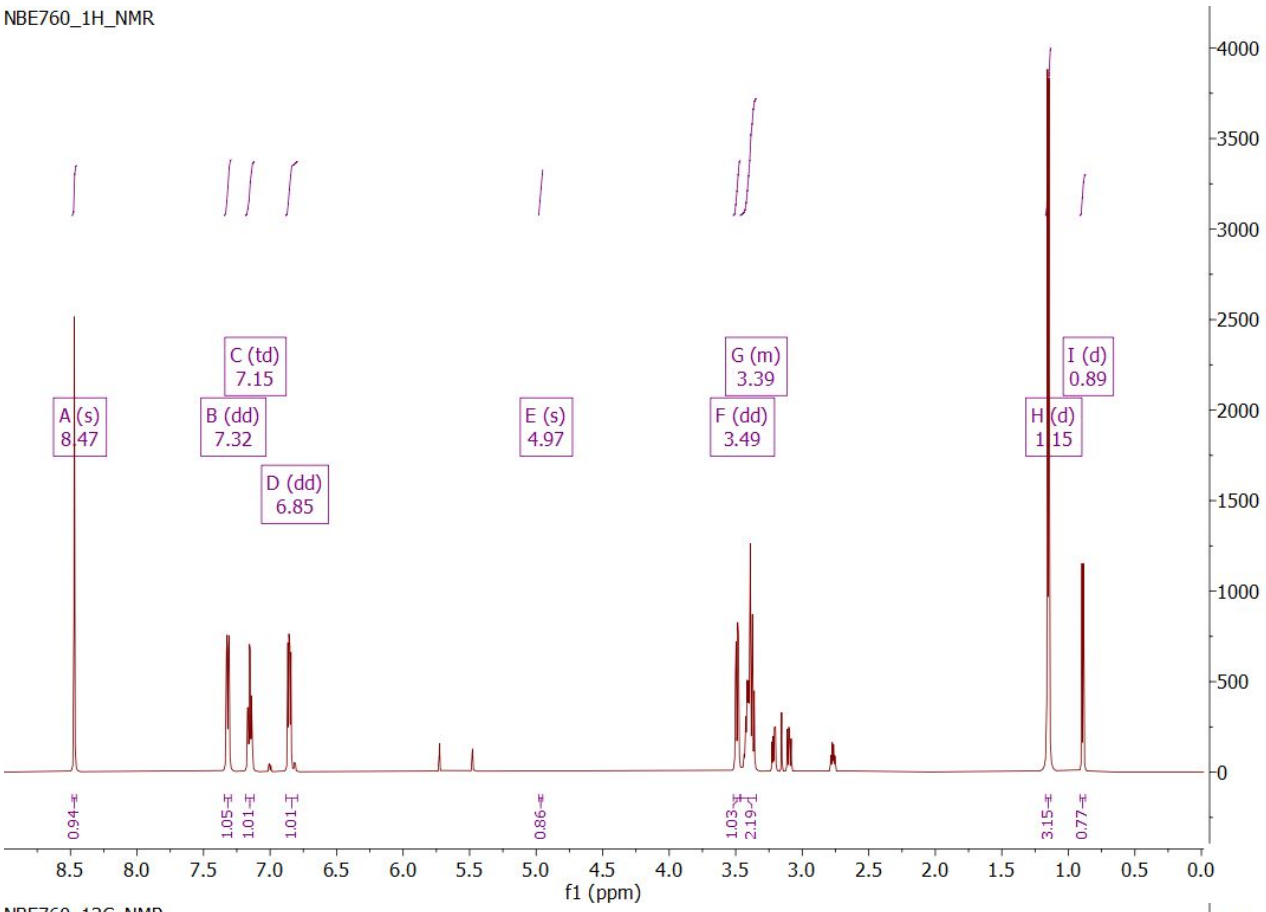
NBE760_1H_NMR
A (s) 8.47
B (dd) 7.32
C (td) 7.15
D (dd) 6.85
E (s) 4.97
F (dd) 3.49
G (m) 3.39
H (d) 1.15
I (d) 0.89
0.94
1.05
1.01
1.01
0.86
1.03
2.19
3.15
0.77
f1 (ppm)

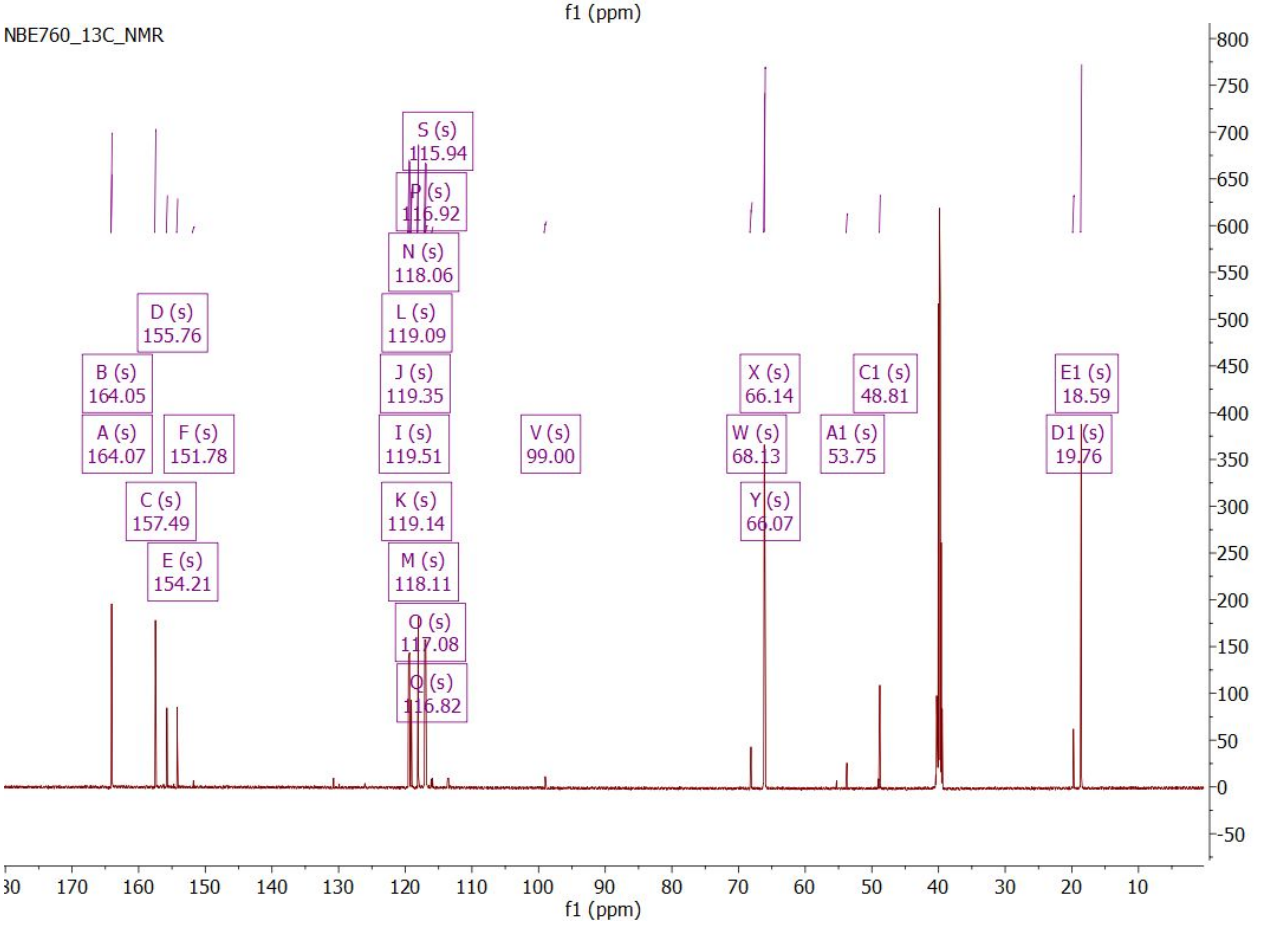
NBE760_13C_NMR
A (s) 164.07
B (s) 164.05
C (s) 157.49
D (s) 155.76
E (s) 154.21
F (s) 151.78
I (s) 119.51
J (s) 119.35
K (s) 119.14
L (s) 119.09
M (s) 118.11
N (s) 118.06
O (s) 117.08
P (s) 116.92
Q (s) 116.82
S (s) 115.94
V (s) 99.00
W (s) 68.13
X (s) 66.14
Y (s) 66.07
A1 (s) 53.75
C1 (s) 48.81
D1 (s) 19.76
E1 (s) 18.59
f1 (ppm)

Complex synthesis. 0.368g, 1.866mmol of (E)-4-fluoro-2-(((1-hydroxypropan-2-yl)imino)methyl)phenol was placed in a 100ml RB flask containing 10ml of methanol under stirring to obtain a clear solution before adding 0.520ml, 1.244mmol of triethylamine and stir for 30 minutes, 0.121g, 0.622mmol of $YCl_3.xH_2O$ was slowly added and stirring continued for 10 mins before adding 0.296g, 1.244mmol of $CoCl_2.6H_2O$ and refluxed for three hours at 68°C, after which the solution was allowed to cool to room temperature, filtered, and placed in a vial for the slow growth of crystals.

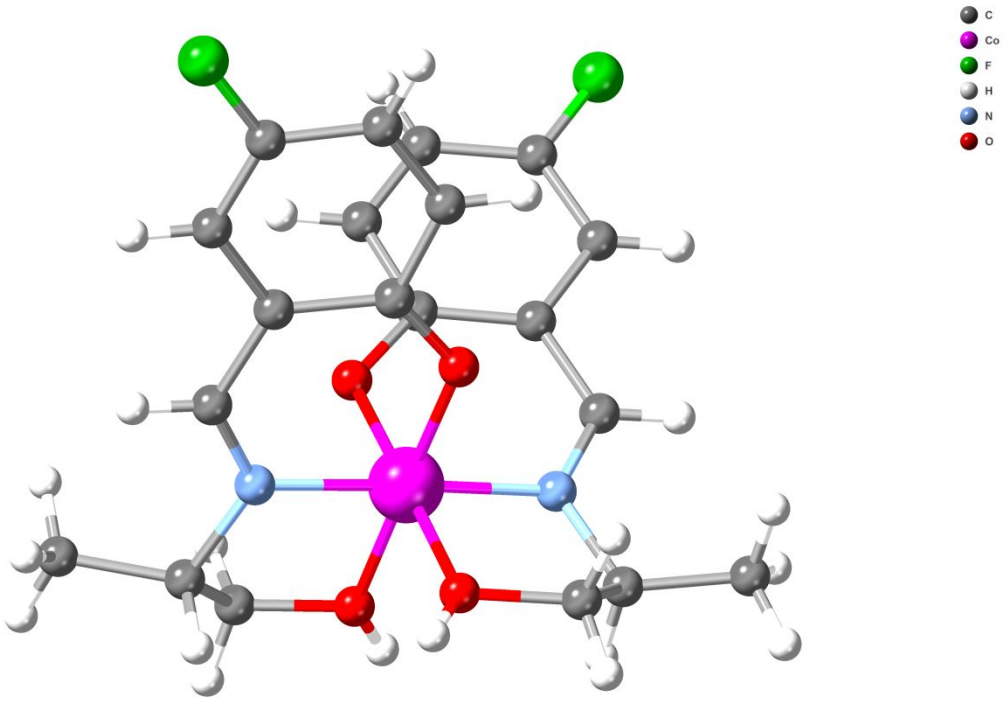

Figure S6. The byproduct compound Co(III) obtained with the methyl substituted ligand. Data were recorded at the University of Sussex in a Rigaku rotating anode diffractometer Crystal Data for $C_{20.66}CoF_2H_{23.64}N_2O_{4.66}$ (M =471.46 g/mol): tetragonal, space group $P4_12_12$ (no. 92), a = 17.3925(3) Å, c = 13.8603(5) Å, V = 4192.7(2) Å^3, Z = 8, T = 100.00 K, μ(Cu Kα) = 6.875 mm^{-1}, *Dcalc* = 1.494 g/cm^3, 8985 reflections measured (10.172° ≤ 2Θ ≤ 135.614°), 3702 unique (R_{int} = 0.0448, R_{sigma} = 0.0593) which were used in all calculations. The final R_1 was 0.0582 (I > 2σ(I)) and wR_2 was 0.1483 (all data).

$^{1}H,^{89}Y$-HMBC experiments were acquired on a Bruker AVIII 400 equipped with a broadband-observe probe at 298 K. Specifically the use of the library pulse sequence hmbcgpndqf, and the AU program gradratio to configure the appropriate gradient strengths for coherence selection (GPZ1 70%, GPZ2 30%, and GPZ3 44.9%), and the indirect dimension configured to span a 89Y chemical shift range of -400 to 900 ppm. Adequate signal to noise could be acquired within ca. 10 min with 256 increments, four transients per increment, an acquisition time of 0.21 s (2 AQ), and a relatively short relaxation delay (D1) of 0.25 s. The experiment was optimised for a nominal ^{1}H-^{89}Y J-coupling of 3.5 Hz as a compromise value for small couplings without excessive relaxation losses. Spectra were referenced indirectly to the deuterium lock shift of $CDCl_3$.

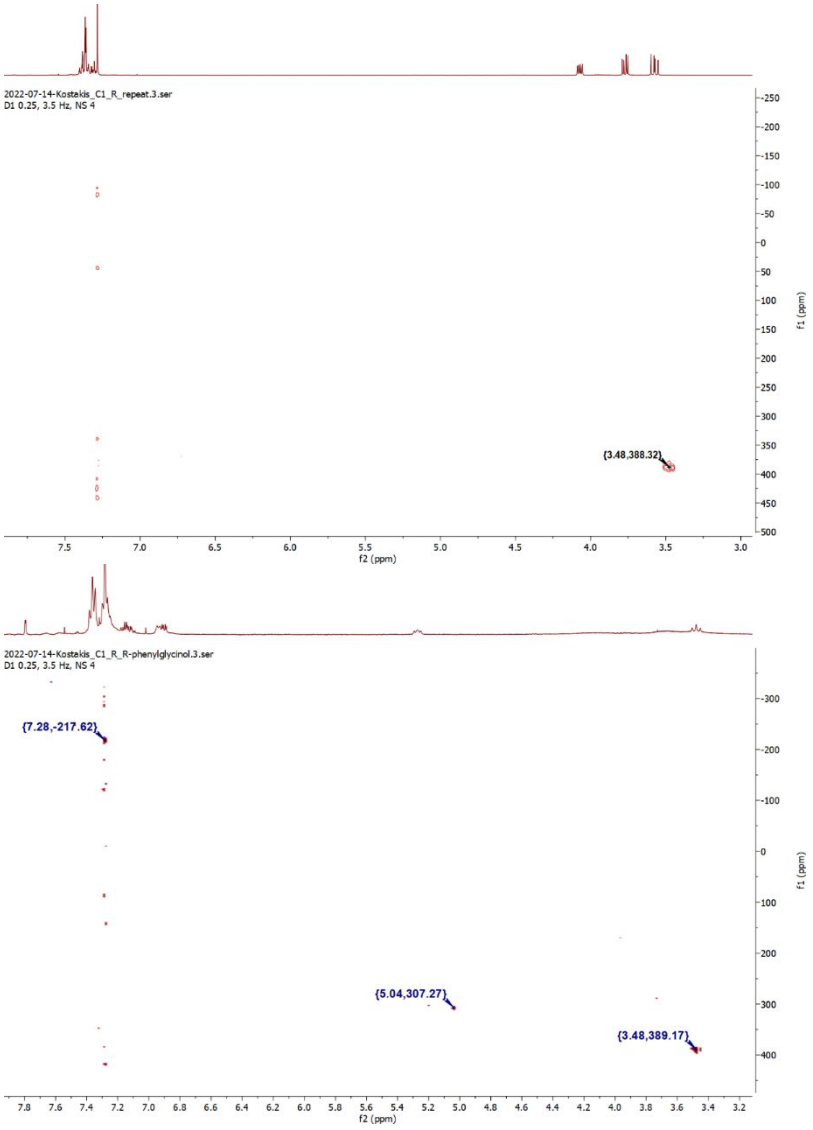

Figure S7. ^{1}H-^{89}Y NMR of the complex (up) and the complex plus phenylglycinol in 1:20 ratio (down)

Computational Studies

Computational details

Molecular structures for all the species were optimized without constraints by using Density Functional Theory (DFT) based methods as implemented in the in the Gaussian16 program (Version C.01).[4] Geometry optimizations were performed using the hybrid B3LYP[5] functional and the metahybrid M06[6] and the combined DZ with the Stuttgart-Dresden ECP basis set (SDD)[7] and a balanced polarized triple-zeta (such as Def2-TZVP[8,9]) basis set respectively. The polarizable continuum model (PCM)[10,11] was used as the implicit solvent model, where methanol (ε =32.613), dimethyl sulfoxide (ε= 46.826), chloroform (ε =4.7113) were applied as the solvents. Vibrational frequency calculations were performed at 298.15 K and 1 atm to calculate zeropoint energies and to confirm the nature of the optimized stationary point (i.e., no imaginary frequency for local minimum). Nuclear Magnetic Resonance chemical shifts were computed at the same level of theory.

Computational studies on the Rh complexes with the different auxiliaries, as reported by Bian and Song in 2019.[12]

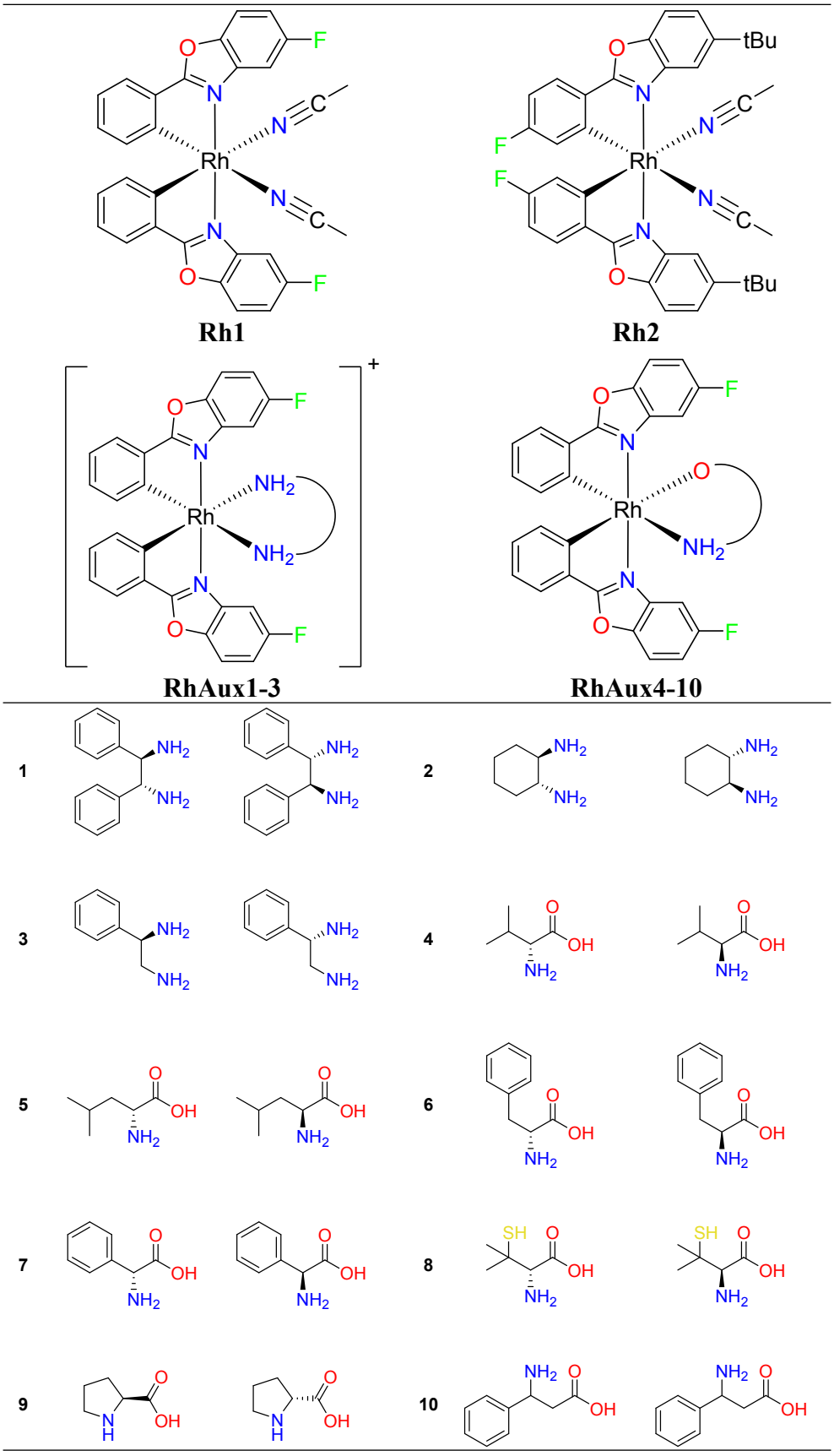

Figure S8. Rh Chiral Sensors and Auxiliaries.[12]

Table S10. Computed ^{19}F NMR values for the shown complexes in Figure S8 at the B3LYP/SDD level of theory and comparison to the previously reported experimental NMR chemical shifts.[12]

Complex	Literature Experimental ^{19}F NMR Shift (ppm)	Solvent	Computed ^{19}F NMR NMR Shift (ppm)[a]	error %
Rh1	-112.25	$CDCl_3$	-107.69	4.1%
Rh2	-103.79	$CDCl_3$	-95.60	7.9%
RhAux1a	-115.12	DMSO-d6	-106.75	7.3%
	-115.12		-106.76	7.3%
RhAux1b	-115.39	DMSO-d6	-107.41	6.9%
	-115.39		-107.42	6.9%
RhAux2a	-115.02	DMSO-d6	-107.97	6.1%
	-115.02		-107.98	6.1%
RhAux2b	-115.03	DMSO-d6	-108.08	6.0%
	-115.03		-108.08	6.0%
RhAux3a	-115.08	DMSO-d6	-107.20	6.8%
	-115.10		-107.54	6.6%
RhAux3b	-115.26	DMSO-d6	-107.60	6.6%
	-115.28		-107.90	6.4%
RhAux4a	-116.05	MeOD	-108.04	6.9%
	-117.00		-108.78	7.0%
RhAux4b	-116.40	MeOD	-108.38	6.9%
	-116.95		-109.30	6.5%
RhAux5a	-116.28	MeOD	-108.96	6.3%
	-116.66		-109.74	5.9%
RhAux5b	-116.55	MeOD	-108.32	7.1%
	-116.60		-109.04	6.5%
RhAux6a	-114.00	MeOD	-109.00	4.4%
	-115.90		-109.96	5.1%
RhAux6b	-116.00	MeOD	-108.19	6.7%
	-116.20		-109.27	6.0%
RhAux7a	-116.10	MeOD	-107.46	7.4%
	-116.30		-109.06	6.2%
RhAux7b	-116.04	MeOD	-107.85	7.1%
	-116.40		-109.03	6.3%
RhAux8a	-116.60	MeOD	-107.87	7.5%
	-117.75		-108.49	7.9%
RhAux8b	-117.00	MeOD	-107.88	7.8%
	-117.00		-109.15	6.7%
RhAux9a	-115.62	MeOD	-108.55	6.1%
	-116.10		-108.91	6.2%
RhAux9b	-115.04	MeOD	-108.26	5.9%
	-116.64		-109.74	5.9%
RhAux10a	-116.10	MeOD	-107.47	7.4%
	-116.19		-108.99	6.2%
RhAux10b	-116.25	MeOD	-108.60	6.6%
	-116.73		-109.86	5.9%

a)Fluorobenzene (C_6H_5F) was used as the ^{19}F NMR reference calculated at the same level of theory.

Table S11. Computed ^{19}F NMR values for the shown complexes in Figure **S8** at the M06/Def2-TZVP level of theory and comparison to the previously reported experimental NMR chemical shifts.[12]

Complex	Literature Experimental ^{19}F NMR Shift (ppm)	Solvent	Computed ^{19}F NMR Shift (ppm)[a]	error %
Rh1	-112.25	$CDCl_3$	-110.61	1.5%
Rh2	-103.79	$CDCl_3$	-97.54	6.0%
RhAux1a	-115.12	DMSO-d6	-109.52	4.9%
	-115.12		-109.71	4.7%
RhAux1b	-115.39	DMSO-d6	-110.08	4.6%
	-115.39		-110.17	4.5%
RhAux2a	-115.02	DMSO-d6	-110.45	4.0%
	-115.02		-110.45	4.0%
RhAux2b	-115.03	DMSO-d6	-110.68	3.8%
	-115.03		-110.68	3.8%
RhAux3a	-115.08	DMSO-d6	-109.98	4.4%
	-115.10		-111.36	3.2%
RhAux3b	-115.26	DMSO-d6	-111.08	3.6%
	-115.28		-111.36	3.4%
RhAux4a	-116.05	MeOD	-111.49	3.9%
	-117.00		-112.51	3.8%
RhAux4b	-116.40	MeOD	-111.34	4.3%
	-116.95		-111.36	4.8%
RhAux5a	-116.28	MeOD	-111.40	4.2%
	-116.66		-113.07	3.1%
RhAux5b	-116.55	MeOD	-111.69	4.2%
	-116.60		-111.76	4.2%
RhAux6a	-114.00	MeOD	-112.043	1.7%
	-115.90		-112.25	3.2%
RhAux6b	-116.00	MeOD	-110.12	5.1%
	-116.20		-111.70	3.9%
RhAux7a	-116.10	MeOD	-112.77	2.9%
	-116.30		-113.14	2.7%
RhAux7b	-116.04	MeOD	-111.76	3.7%
	-116.40		-111.92	3.8%
RhAux8a	-116.60	MeOD	-111.04	4.8%
	-117.75		-111.20	5.6%
RhAux8b	-117.00	MeOD	-111.59	4.6%
	-117.00		-112.03	4.3%
RhAux9a	-115.62	MeOD	-111.34	3.7%
	-116.10		-111.79	3.7%
RhAux9b	-115.04	MeOD	-111.32	3.2%
	-116.64		-113.39	2.8%
RhAux10a	-116.10	MeOD	-111.47	4.0%
	-116.19		-111.72	3.8%
RhAux10b	-116.25	MeOD	-111.60	4.0%
	-116.73		-112.61	3.5%

a)Fluorobenzene (C_6H_5F) was used as the ^{19}F NMR reference calculated at the same level of theory.

Computational studies on the simplified $CoYL_2$ model system with the different analytes reported in this work.

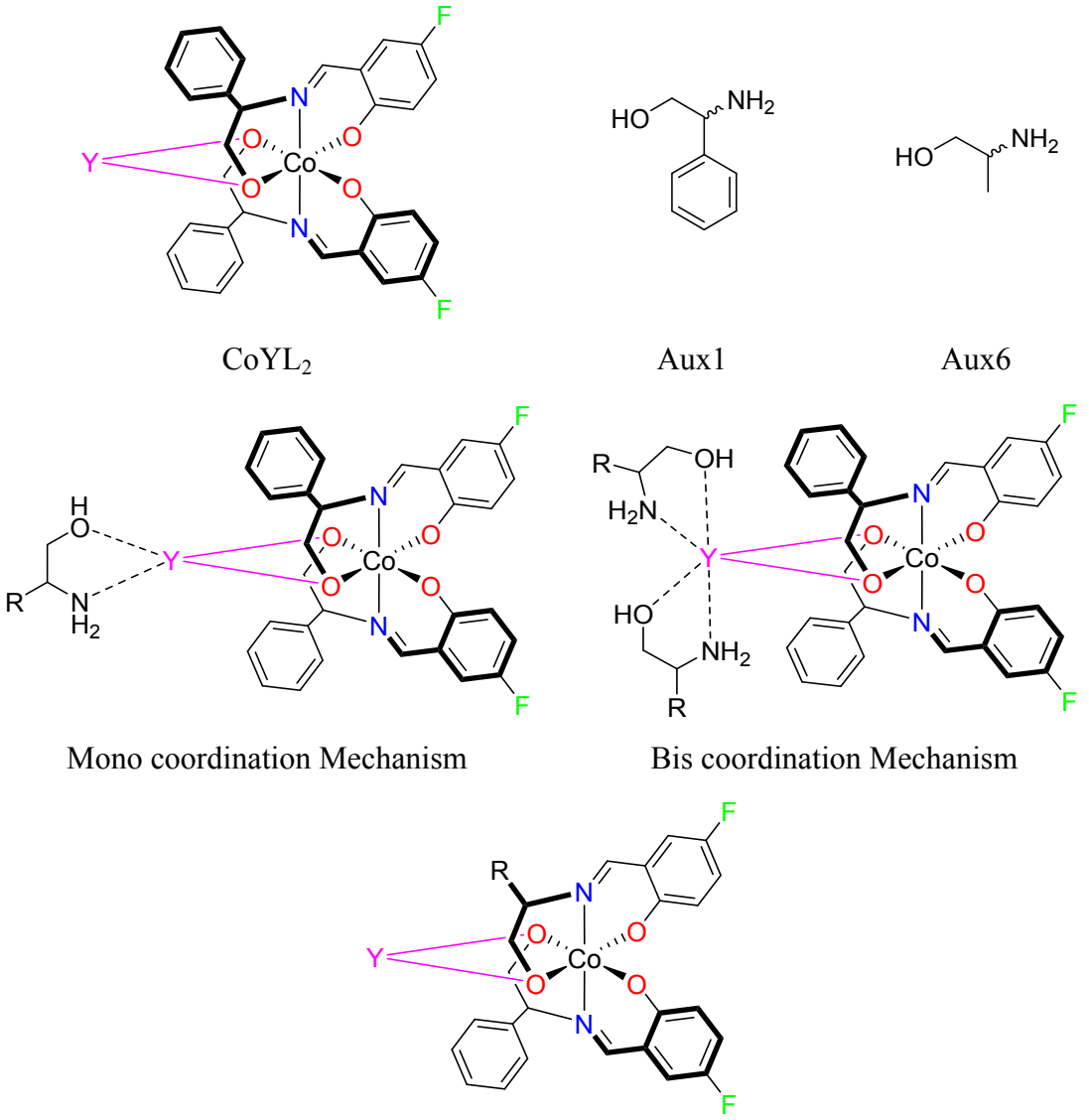

Figure **S9**. $CoYL_2$ model, analytes used and resulting complexes from the plausible mechanisms as shown in Scheme 4 within the manuscript.

Table S21. Computed ^{19}F NMR values for the shown $CoYL_2$ complexes in Figure **S10** at the B3YLP/SDD level of theory and comparison to the reported experimental NMR chemical shifts in this work.

**Complex with analyte**	**Mechanism**	**Experimental ^{19}F NMR Shift (ppm)**	**Solvent**	**Computed ^{19}F NMR Shift (ppm)[a]**	**error %**
Complex only ($CoYL_2$)[b]	None	-131.23	MeOD	-147.19	12.2%
Complex only ($CoYL_2$)	None	-131.23	MeOD	-138.75	5.7%
A1S	Mono Coordination	-132.12	$CDCl_3$	-138.28	4.7%
A1R	Mono Coordination	-132.05	$CDCl_3$	-139.27	5.5%
A6S	Mono Coordination	-132.62	$CDCl_3$	-139.01	4.8%
A6R	Mono Coordination	-132.72	$CDCl_3$	-138.81	4.6%
A1S	Bis Coordination	-132.12	$CDCl_3$	-141.33	7.0%
A1R	Bis Coordination	-132.05	$CDCl_3$	-143.12	8.4%
A6S	Bis Coordination	-132.62	$CDCl_3$	-142.20	7.2%
A6R	Bis Coordination	-132.72	$CDCl_3$	-142.38	7.3%
A1S	Partial Ligand Exchange	-132.12	$CDCl_3$	-138.48	4.8%
A1R	Partial Ligand Exchange	-132.05	$CDCl_3$	-137.84	4.4%
A6S	Partial Ligand Exchange	-132.62	$CDCl_3$	-138.04	4.1%
A6R	Partial Ligand Exchange	-132.72	$CDCl_3$	-138.06	4.0%
MeOH	Mono Coordination	-138.99	MeOH	-138.46	<0.1%
MeOH	Bis Coordination	-138.99	MeOH	-138.03	<0.1%
MeOH + A1S	Bis Coordination	-132.12	$CDCl_3$	-142.14	<0.1%

a) Fluorobenzene (C_6H_5F) was used as the ^{19}F NMR reference calculated at the same level of theory;
b)This calculation was performed at the M06/Def2-TZVP level of theory.

Computed NMR values for Co_3YL_6 with the different auxiliaries reported in this work.

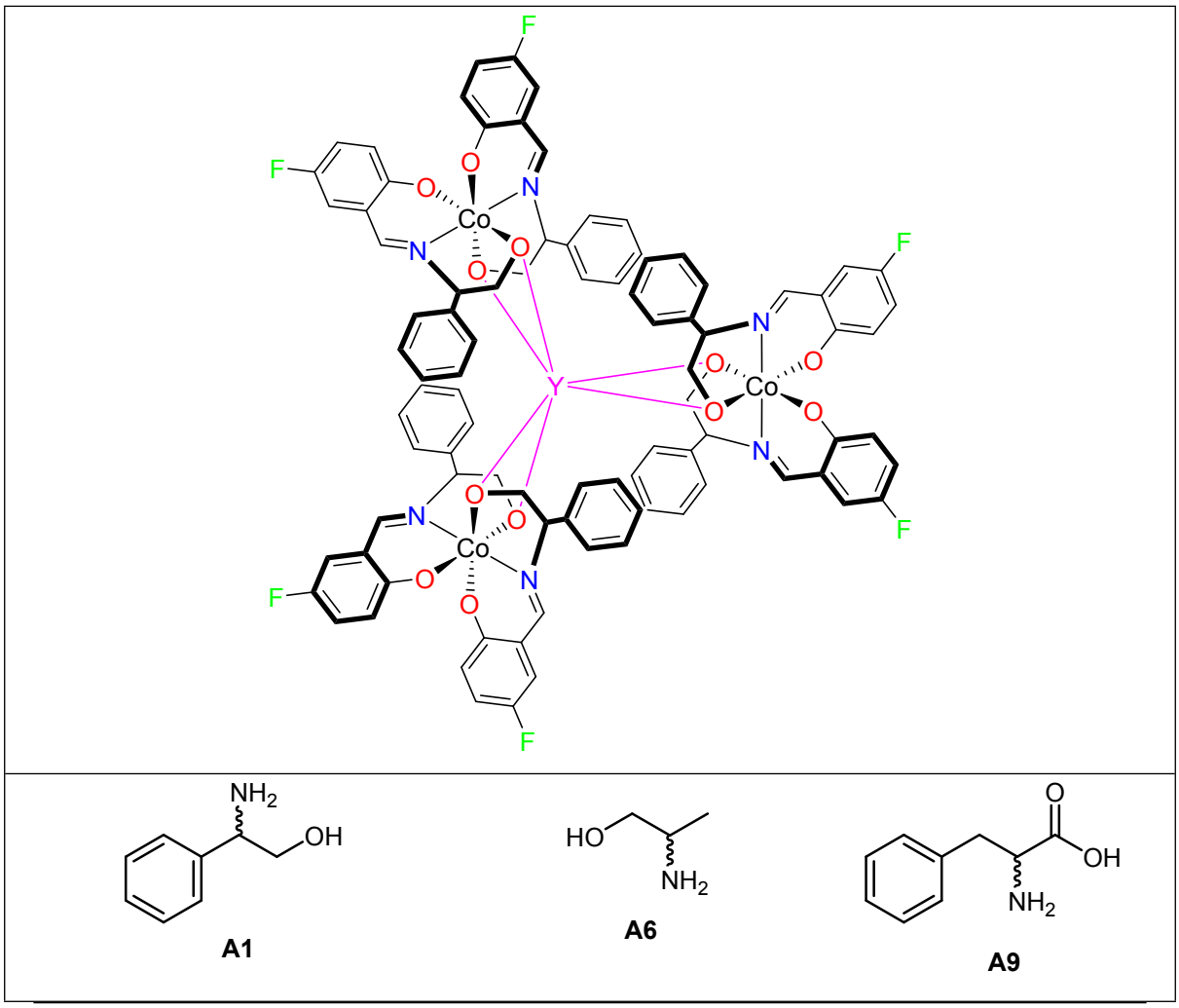

Figure **S10**. Co_3YL_6 model and analytes used in this work.

Table S13. Computed ^{19}F NMR values for the shown Co_3YL_6 complexes in Figure **S10** at the B3LYP/SDD level of theory and comparison to the reported experimental NMR shifts in this work.

Complex with auxiliary	Mechanism	Experimental ^{19}F NMR Shift (ppm)	Solvent	Computed ^{19}F NMR Shift (ppm)[a]	error %
Complex Only (Co_3YL_6)	None	-131.23	MeOD	-130.30	0.7%
Complex Only[b] (Co_3YL_6)	None	-131.23	MeOD	-133.32	1.6%
A1R	Partial Ligand Exchange	-132.05	$CDCl_3$	-129.99	<0.1%
A1S	Partial Ligand Exchange	-132.12	$CDCl_3$	-130.43	1.3%
A1S[b]	Partial Ligand Exchange	-132.12	$CDCl_3$	-127.81	3.3%
A6R	Partial Ligand Exchange	-132.72	$CDCl_3$	-130.45	1.6%
A6S	Partial Ligand Exchange	-132.62	$CDCl_3$	-130.64	1.6%
A6S[b]	Partial Ligand Exchange	-132.62	$CDCl_3$	-127.92	3.6%
A9R	Partial Ligand Exchange	-134.66	MeOD	-129.29	4.0%
A9S	Partial Ligand Exchange	-134.69	MeOD	-129.64	3.8%
A1R	6x Partial Ligand Exchange[c]	-132.05	$CDCl_3$	-129.11	0.7%
A1S	6x Partial Ligand Exchange	-132.12	$CDCl_3$	-130.43	1.3%
A6R	6x Partial Ligand Exchange	-132.72	$CDCl_3$	-130.64	1.5%
A6S	6x Partial Ligand Exchange	-132.62	$CDCl_3$	-131.64	0.8%
A1R	Ligand Substitution	-132.05	$CDCl_3$	-131.25	0.9%
A1S	Ligand Substitution	-132.12	$CDCl_3$	-131.11	0.8%
A6R	Ligand Substitution	-132.72	$CDCl_3$	-131.38	0.9%
A6S	Ligand Substitution	-132.62	$CDCl_3$	-131.30	1.1%
A9R	Ligand Substitution	-134.66	MeOD	-129.21	4.0%
A9S	Ligand Substitution	-134.69	MeOD	-129.36	7.1%

a) Fluorobenzene (C_6H_5F) was used as the ^{19}F NMR reference calculated at the same level of theory; b)This calculation was performed at the M06/Def2-TZVP level; c) Saturated system with 6 ligands partially exchanged

Table S14. Comparison between the computed ^{19}F NMR values for the shown complexes in Figure **SX** at the B3LYP/SDD level of theory and MeOH and Chloroform.

Complex with analyte	Mechanism	Methanol[b]	Chloroform[b]
Complex only (Co_3YL_6)	None	0.71%	0.31%
Complex only (Co_3YL_6)[a]	None	1.59%	2.67%
A1R	Partial Ligand Exchange	0.14%	0.04%
A1S	Partial Ligand Exchange	1.38%	1.28%
A6R	Partial Ligand Exchange	1.73%	1.64%
A6S	Partial Ligand Exchange	1.66%	1.57%
A9R	Partial Ligand Exchange	3.98%	3.90%
A9S	Partial Ligand Exchange	3.75%	3.65%
A1R	6x Partial Ligand Exchange	1.11%	0.73%
A1S	6x Partial Ligand Exchange	1.38%	1.28%
A6R	6x Partial Ligand Exchange	1.68%	1.49%
A6S	6x Partial Ligand Exchange	0.96%	0.82%
A1R	Ligand Substitution	0.73%	0.93%
A1S	Ligand Substitution	0.92%	0.76%
A6R	Ligand Substitution	1.09%	0.93%
A6S	Ligand Substitution	1.23%	1.07%
A9R	Ligand Substitution	4.05%	3.79%
A9S	Ligand Substitution	7.12%	6.94%

a)This calculation was performed at the M06/Def2-TZVP level; b) average of errors

Molecular structures, cartesian coordinates and corresponding energies for all the computed species.

Co_3YL_6

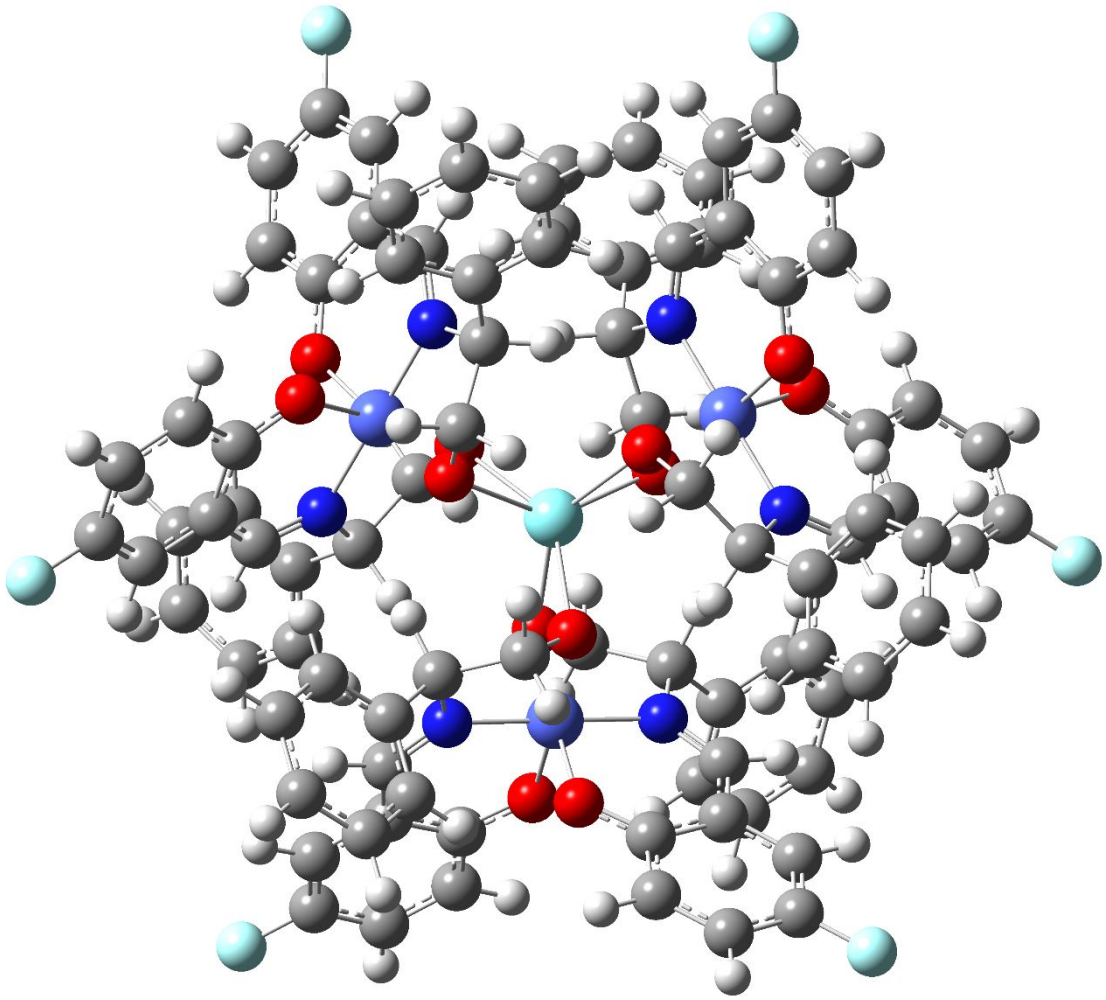

	B3LYP/SDD	B3LYP/SDD, pcm=methanol	M06/Def2-TZVP
**Electronic Energy (EE)**	-5779.148221	-5779.204993	-9488.791487
**EE + Zero-point Energy**	-5777.688200	-5777.745779	-9487.340899
**EE + Thermal Energy Correction**	-5777.588323	-5777.646118	-9487.243076
**EE + Thermal Enthalpy Correction**	-5777.587379	-5777.645174	-9487.242131
**EE + Thermal Free Energy Correction**	-5777.834548	-5777.89004	-9487.474082

	B3LYP/SDD	B3LYP/SDD, pcm=methanol	M06/Def2-TZVP

Tag	Symbol	X	Y	Z	Symbol	X	Y	Z	Symbol	X	Y	Z
1	Y	10.49667	10.49667	-10.49667	Y	10.50 47	10.50468	-10.50 47	Y	0.006787	0.003786	0.012948
2	Co	9.706991	8.625599	-13.15 51	Co	9.698412	8.620403	-13.16 74	Co	0.289417	-3.237 24	0.009373
3	Co	13.15513	9.706991	-8.625 6	Co	13.16735	9.698436	-8.620 42	Co	-2.949 66	1.366148	-0.001 23
4	Co	8.625599	13.15513	-9.706 99	Co	8.620418	13.16733	-9.698 41	Co	2.663768	1.877396	0.002816
5	F	11.23986	1.878136	-13.62 16	F	11.29694	1.868309	-13.15 26	F	-4.713 31	-6.145 11	-3.462 06
6	F	13.62164	11.23986	-1.878 14	F	13.15236	11.29706	-1.868 35	F	-2.986 62	7.156907	-3.463 17
7	F	1.878136	13.62164	-11.23 99	F	1.868319	13.15257	-11.29 69	F	7.671 13	-0.958 38	-3.519 38
8	F	6.391568	11.14046	-18.70 29	F	6.518945	11.56052	-18.58 74	F	5.709665	-5.275 33	3.472488
9	F	18.70286	6.391567	-11.14 05	F	18.58748	6.518987	-11.56 04	F	-7.414 61	-2.334 12	3.447 77
10	F	11.14046	18.70286	-6.391 57	F	11.56059	18.58733	-6.518 96	F	1.693187	7.555 04	3.515064
11	O	8.899311	6.921081	-13.37 96	O	8.911714	6.896 79	-13.39 93	O	0.014014	-4.577 63	-1.281 74
12	O	13.37962	8.899311	-6.921 08	O	13.39926	8.911768	-6.896 79	O	-3.973 87	2.269683	-1.295 29
13	O	6.921081	13.37962	-8.899 31	O	6.896807	13.39925	-8.911 71	O	3.953794	2.323807	-1.291 44
14	O	10.59215	10.32475	-12.77 59	O	10.57111	10.31894	-12.80 54	O	0.439904	-1.805 35	1.312045
15	O	12.77593	10.59215	-10.32 48	O	12.80536	10.57111	-10.31 9	O	-1.784 15	0.525556	1.303487

16	O	10.32475	12.77593	-10.59021	O	10.31895	12.80535	-10.5711	O	1.358663	1.279355	1.310028
17	O	9.891333	8.849805	-15.0323	O	9.856243	8.832923	-15.058	O	0.785149	-4.51976	1.294566
18	O	15.03229	9.891333	-8.84981	O	15.05804	9.856294	-8.83292	O	-4.31268	1.570186	1.280662
19	O	8.849805	15.03229	-9.89133	O	8.832941	15.05802	-9.85624	O	3.526794	2.946406	1.288325
20	O	9.510583	8.561077	-11.2132	O	9.505484	8.553021	-11.236	O	-0.09914	-1.84748	-1.2878
21	O	11.21317	9.510583	-8.56108	O	11.23602	9.505478	-8.55305	O	-1.54478	1.017244	-1.29374
22	O	8.561078	11.21317	-9.51058	O	8.553035	11.236	-9.50549	O	1.654675	0.845513	-1.29447
23	N	11.52096	7.950338	-13.2247	N	11.51626	7.956195	-13.2272	N	-1.50167	-3.33373	0.69516
24	N	13.22466	11.52096	-7.95034	N	13.22713	11.51629	-7.95624	N	-2.14963	2.969495	0.690987
25	N	7.950338	13.22466	-11.521	N	7.956205	13.22715	-11.5163	N	3.653728	0.375719	0.674561
26	N	7.890768	9.295581	-13.0942	N	7.884336	9.293214	-13.1001	N	2.07102	-3.02951	-0.67609
27	N	13.09416	7.890767	-9.29558	N	13.10014	7.884349	-9.29321	N	-3.65199	-0.28362	-0.69083
28	N	9.295581	13.09416	-7.89077	N	9.293235	13.10008	-7.88434	N	1.581941	3.314091	-0.66883
29	C	9.521202	5.741426	-13.4355	C	9.541525	5.719446	-13.3267	C	-1.14296	-4.89419	-1.75594
30	C	13.43551	9.521203	-5.74143	C	13.32666	9.541594	-5.71946	C	-3.67597	3.433018	-1.76548
31	C	5.741426	13.43551	-9.5212	C	5.719461	13.3267	-9.54152	C	4.806294	1.486674	-1.77679

32	C	8.712369	4.566624	-13.5428	C	8.744254	4.532064	-13.3632	C	-1.18616	-5.5959	-2.98397
33	C	13.54277	8.712369	-4.56662	C	13.36308	8.74434	-4.53206	C	-4.25715	3.819201	-2.99661
34	C	4.566624	13.54277	-8.71237	C	4.532081	13.36314	-8.74425	C	5.426452	1.81102	-3.00663
35	H	7.635666	4.698448	-13.5839	H	7.667378	4.644805	-13.4405	H	-0.2388	-5.80335	-3.46849
36	H	13.58386	7.635667	-4.69845	H	13.44048	7.667464	-4.64479	H	-4.90178	3.098267	-3.48649
37	H	4.698448	13.58386	-7.63567	H	4.644824	13.44051	-7.66737	H	5.126115	2.73769	-3.48245
38	C	9.275962	3.293062	-13.6018	C	9.318138	3.261454	-13.3035	C	-2.36785	-6.00761	-3.54382
39	C	13.60178	9.275962	-3.29306	C	13.30331	9.318239	-3.26146	C	-4.02776	5.050827	-3.55313
40	C	3.293062	13.60178	-9.27596	C	3.26147	13.30343	-9.31813	C	6.372328	1.000441	-3.57864
41	H	8.658285	2.403507	-13.6844	H	8.705671	2.36502	-13.3285	H	-2.38377	-6.54369	-4.48595
42	H	13.68438	8.658286	-2.40351	H	13.32838	8.705784	-2.36502	H	-4.47956	5.330652	-4.49805
43	H	2.403508	13.68438	-8.65829	H	2.365037	13.32851	-8.70566	H	6.837752	1.262988	-4.52188
44	C	10.6792	3.167692	-13.5606	C	10.71924	3.159285	-13.2116	C	-3.57114	-5.73929	-2.89851
45	C	13.56056	10.6792	-3.16769	C	13.21142	10.71934	-3.15932	C	-3.20414	5.963571	-2.90126
46	C	3.167692	13.56056	-10.6792	C	3.159297	13.21157	-10.7192	C	6.749167	-0.17954	-2.9444
47	C	11.51247	4.266529	-13.4685	C	11.54493	4.267922	-13.1816	C	-3.59097	-5.06327	-1.716

48	C	13.46852	11.51248	-4.26653	C	13.18147	11.54501	-4.26797	C	-2.61496	5.645598	-1.71501
49	C	4.266529	13.46852	-11.5125	C	4.267931	13.18161	-11.5449	C	6.182951	-0.54499	-1.76045
50	H	12.59097	4.130248	-13.4537	H	12.62336	4.147758	-13.1204	H	-4.53995	-4.85926	-1.23025
51	H	13.45374	12.59097	-4.13025	H	13.12025	12.62345	-4.14782	H	-1.97083	6.368677	-1.22475
52	H	4.130248	13.45374	-12.591	H	4.147765	13.12042	-12.6234	H	6.485805	-1.47191	-1.28369
53	C	10.94829	5.573747	-13.3978	C	10.96865	5.56954	-13.2323	C	-2.39176	-4.61623	-1.13307
54	C	13.39782	10.94829	-5.57375	C	13.23216	10.96872	-5.56958	C	-2.8226	4.38134	-1.13511
55	C	5.573747	13.39782	-10.9483	C	5.569551	13.23224	-10.9686	C	5.198153	0.263313	-1.16468
56	C	11.85666	6.696632	-13.3405	C	11.87005	6.703319	-13.241	C	-2.47547	-3.91312	0.106306
57	C	13.3405	11.85666	-6.69663	C	13.24096	11.8701	-6.70337	C	-2.17529	4.104 66	0.106511
58	C	6.696632	13.3405	-11.8567	C	6.703328	13.24102	-11.87	C	4.641453	-0.1698	0.076327
59	H	12.91869	6.453914	-13.4333	H	12.93525	6.464656	-13.2877	H	-3.4629	-3.87832	0.58301
60	H	13.43325	12.91869	-6.45391	H	13.28756	12.93531	-6.46472	H	-1.66269	4.946427	0.588321
61	H	6.453913	13.43325	-12.9187	H	6.464663	13.28765	-12.9352	H	5.112854	-1.04239	0.545378
62	C	12.58312	9.023106	-13.2529	C	12.57105	9.034044	-13.2986	C	-1.71071	-2.60774	1.95932
63	C	13.25287	12.58312	-9.02311	C	13.29853	12.57107	-9.0341	C	-1.41908	2.790452	1.956709

64	C	9.023106	13.25287	-12.5831	C	9.03405	13.29854	-12.571	C	3.141586	-0.17643	1.940032
65	H	12.99793	9.096729	-12.239	H	13.02062	9.111703	-12.3004	H	-2.28248	-1.70202	1.712803
66	H	12.23896	12.99793	-9.09673	H	12.30038	13.02066	-9.11177	H	-0.34814	2.844953	1.714633
67	H	9.096729	12.23896	-12.9979	H	9.111706	12.30039	-13.0206	H	2.650961	-1.12951	1.69566
68	C	11.83315	10.34648	-13.5381	C	11.80935	10.35334	-13.57	C	-0.32898	-2.15673	2.426717
69	C	13.53806	11.83315	-10.3465	C	13.56997	11.80935	-10.3534	C	-1.70785	1.364374	2.419975
70	C	10.34648	13.53806	-11.8332	C	10.35335	13.56996	-11.8094	C	2.055088	0.784565	2.418037
71	H	11.60669	10.43858	-14.603	H	11.58229	10.45372	-14.6345	H	0.154958	-2.95912	2.997459
72	H	14.603	11.60669	-10.4386	H	14.6345	11.5823	-10.4538	H	-2.643	1.338284	2.993234
73	H	10.43858	14.603	-11.6067	H	10.45373	14.6345	-11.5823	H	2.50628	1.602359	2.993822
74	H	12.45178	11.19603	-13.2229	H	12.42051	11.20582	-13.2498	H	-0.4465	-1.2993	3.105018
75	H	13.2229	12.45178	-11.196	H	13.24978	12.4205	-11.2059	H	-0.90213	1.041139	3.09512
76	H	11.19603	13.2229	-12.4518	H	11.20583	13.24977	-12.4205	H	1.376622	0.245163	3.094594
77	C	13.43174	8.524481	-15.6061	C	13.33919	8.504266	-15.6751	C	-2.10265	-4.68555	3.324539
78	C	15.60609	13.43174	-8.52448	C	15.67506	13.33919	-8.5043	C	-3.03891	4.157838	3.314347
79	C	8.524481	15.60609	-13.4317	C	8.504273	15.67507	-13.3392	C	5.143955	0.53837	3.286548

80	H	12.39568	8.466342	-15.9331	H	12.29347	8.429775	-15.9644	H	-1.3091	-5.16963	2.761184
81	H	15.93306	12.39568	-8.46634	H	15.96432	12.29346	-8.42979	H	-3.84832	3.717823	2.737546
82	H	8.466341	15.93306	-12.3957	H	8.429784	15.96433	-12.2935	H	5.158259	1.464447	2.717699
83	C	14.48009	8.33795	-16.522	C	14.35497	8.320855	-16.6291	C	-2.76955	-5.36016	4.330534
84	C	16.52196	14.48009	-8.33795	C	16.62909	14.35496	-8.32088	C	-3.30124	5.062249	4.32688
85	C	8.337949	16.52196	-14.4801	C	8.320861	16.62991	-14.3549	C	6.06909	0.308302	4.288079
86	H	14.25643	8.144725	-17.5685	H	14.09418	8.108137	-17.663	H	-2.49983	-6.38399	4.563006
87	H	17.56853	14.25643	-8.14473	H	17.663	14.09415	-8.10814	H	-4.32532	5.338806	4.549986
88	H	8.144724	17.56853	-14.2564	H	8.108145	17.66302	-14.0941	H	6.819676	1.05807	4.510485
89	C	15.8214	8.397877	-16.0891	C	15.71151	8.414019	-16.2499	C	-3.78278	-4.73291	5.040569
90	C	16.08907	15.8214	-8.39788	C	16.24995	15.71149	-8.41407	C	-2.25974	5.617426	5.055649
91	C	8.397876	16.08907	-15.8214	C	8.414022	16.24995	-15.7115	C	6.041388	-0.87799	5.006548
92	H	16.63163	8.251862	-16.7993	H	16.49634	8.272964	-16.9889	H	-4.30675	-5.26704	5.82454
93	H	16.79933	16.63163	-8.25186	H	16.98894	16.49632	-8.27301	H	-2.46932	6.329771	5.844994
94	H	8.251861	16.79933	-16.6316	H	8.272966	16.98893	-16.4963	H	6.771693	-1.05675	5.787005
95	C	16.10582	8.641007	-14.7326	C	16.04235	8.687503	-14.9086	C	-4.12639	-3.42649	4.742509

96	C	14.7326	16.10582	-8.64101	C	14.90862	16.04235	-8.68757	C	-0.95325	5.263739	4.76991
97	C	8.641006	14.7326	-16.10058	C	8.687504	14.90861	-16.04023	C	5.082759	-1.83387	4.721862
98	H	17.13631	8.680542	-14.3888	H	17.08443	8.757495	-14.6071	H	-4.92293	-2.9311	5.286137
99	H	14.38877	17.13631	-8.68054	H	14.60715	17.08444	-8.75758	H	-0.13304	5.698542	5.329531
100	H	8.680541	14.38877	-17.13063	H	8.757493	14.60714	-17.08044	H	5.058259	-2.76685	5.273316
101	C	15.05426	8.824586	-13.8139	C	15.02265	8.865594	-13.9523	C	-3.46047	-2.75049	3.732783
102	C	13.81386	15.05426	-8.82459	C	13.95236	15.02266	-8.86567	C	-0.68898	4.360294	3.753052
103	C	8.824586	13.81386	-15.0543	C	8.865596	13.95235	-15.0226	C	4.157134	-1.60562	3.71617
104	H	15.28037	9.008009	-12.7652	H	15.28475	9.072291	-12.9168	H	-3.73129	-1.72536	3.490944
105	H	12.76519	15.28037	-9.00801	H	12.91683	15.28477	-9.07238	H	0.336851	4.083533	3.52051
106	H	9.008009	12.76519	-15.2804	H	9.07229	12.91682	-15.2848	H	3.405517	-2.3567	3.484624
107	C	13.7115	8.771313	-14.2414	C	13.665	8.778345	-14.3256	C	-2.44946	-3.3738	3.015063
108	C	14.24137	13.7115	-8.77131	C	14.32558	13.66501	-8.7784	C	-1.726	3.805367	3.016575
109	C	8.771312	14.24137	-13.7115	C	8.77835	14.32559	-13.665	C	4.183187	-0.42366	2.989654
110	C	9.006637	9.405533	-15.863	C	9.017288	9.503798	-15.8543	C	1.979005	-4.64143	1.767274
111	C	15.86296	9.006637	-9.40553	C	15.85431	9.017335	-9.50376	C	-5.01202	0.59359	1.750623

112	C	9.405532	15.86296	-9.00664	C	9.503832	15.85426	-9.01729	C	3.028516	4.032915	1.772862
113	C	9.37526	9.535218	-17.2389	C	9.401513	9.696631	-17.2188	C	2.140583	-5.33402	2.990615
114	C	17.23889	9.375259	-9.53522	C	17.21881	9.401574	-9.69658	C	-5.69227	0.793734	2.975152
115	C	9.535218	17.23889	-9.37526	C	9.696673	17.21876	-9.40151	C	3.551617	4.513771	2.996506
116	H	10.35114	9.164124	-17.5361	H	10.35797	9.295575	-17.5394	H	1.242299	-5.70281	3.472575
117	H	17.53606	10.35114	-9.16412	H	17.53943	10.35804	-9.29554	H	-5.56474	1.754644	3.460532
118	H	9.164124	17.53605	-10.3511	H	9.29561	17.53941	-10.358	H	4.328773	3.923178	3.468243
119	C	8.516752	10.10851	-18.1756	C	8.582598	10.37561	-18.122	C	3.374781	-5.54182	3.549 99
120	C	18.17558	8.516752	-10.1085	C	18.12205	8.582659	-10.3755	C	-6.48574	-0.17581	3.531843
121	C	10.10851	18.17558	-8.51675	C	10.37567	18.12197	-8.5826	C	3.107877	5.677917	3.568273
122	H	8.800084	10.20423	-19.2197	H	8.882872	10.52046	-19.1556	H	3.481027	-6.07363	4.488672
123	H	19.21971	8.800083	-10.2042	H	19.1556	8.882943	-10.5204	H	-6.99858	-0.00733	4.471974
124	H	10.20423	19.21971	-8.80008	H	10.52052	19.15552	-8.88287	H	3.51836	6.032148	4.507012
125	C	7.252487	10.5638	-17.7503	C	7.346625	10.8732	-17.6674	C	4.515317	-5.06742	2.908991
126	C	17.75033	7.252486	-10.5638	C	17.66743	7.346669	-10.8731	C	-6.64222	-1.39857	2.886214
127	C	10.5638	17.75033	-7.25249	C	10.87327	17.66732	-7.34663	C	2.11586	6.424733	2.939775

128	C	6.836281	10.45521	-16.43669	C	6.914034	10.71094	-16.3641	C	4.42054	-4.39194	1.729887
129	C	16.43685	6.836281	-10.4552	C	16.36418	6.914064	-10.7109	C	-6.01082	-1.64775	1.705151
130	C	10.45521	16.43685	-6.83628	C	10.711	16.36406	-6.91404	C	1.574075	6.00986	1.760658
131	H	5.847647	10.80486	-16.15	H	5.948256	11.10069	-16.0532	H	5.320761	-4.02455	1.247368
132	H	16.15	5.847647	-10.8049	H	16.05332	5.948274	-11.1006	H	-6.14214	-2.60916	1.218655
133	H	10.80486	16.15	-5.84765	H	11.10076	16.05318	-5.94827	H	0.798152	6.603535	1.287581
134	C	7.708613	9.879623	-15.4674	C	7.746324	10.02742	-15.4321	C	3.163097	-4.15354	1.147026
135	C	15.46738	7.708613	-9.87962	C	15.43216	7.746355	-10.0274	C	-5.17823	-0.67381	1.125704
136	C	9.879623	15.46738	-7.70861	C	10.02747	15.43207	-7.74633	C	2.003196	4.810259	1.165166
137	C	7.218833	9.750966	-14.1135	C	7.237759	9.840426	-14.0887	C	3.127743	-3.44139	-0.0892
138	C	14.1135	7.218832	-9.75097	C	14.08875	7.237776	-9.84039	C	-4.53961	-0.9944	-0.10982
139	C	9.750966	14.1135	-7.21883	C	9.840463	14.08866	-7.23777	C	1.402389	4.428703	-0.07201
140	H	6.17641	10.04067	-13.9558	H	6.211279	10.17367	-13.9177	H	4.095498	-3.2408	-0.5653
141	H	13.95577	6.17641	-10.0407	H	13.9178	6.211289	-10.1736	H	-4.84614	-1.93242	-0.58877
142	H	10.04067	13.95577	-6.17641	H	10.17371	13.91769	-6.21129	H	0.735764	5.164292	-0.53906
143	C	7.219088	9.149916	-11.7498	C	7.203444	9.116834	-11.7643	C	2.156487	-2.27605	-1.93796

144	C	11.74982	7.219088	-9.14992	C	11.76432	7.203443	-9.11684	C	-3.0288	-0.73494	-1.94619
145	C	9.149917	11.74982	-7.21909	C	9.116845	11.76425	-7.20345	C	0.882186	-3.01294	-1.92873
146	H	7.249836	10.13322	-11.2622	H	7.200263	10.09859	-11.2741	H	2.577164	-1.29041	-1.69163
147	H	11.26221	7.249836	-10.1332	H	11.27412	7.200256	-10.0986	H	-2.37925	-1.58542	-1.69039
148	H	10.13322	11.26221	-7.24984	H	10.0986	11.27404	-7.20026	H	-0.17916	2.873979	-1.67663
149	C	8.127596	8.20329	-10.9291	C	8.128195	8.189509	-10.939	C	0.718964	-2.05464	-2.40307
150	C	10.92907	8.127596	-8.20329	C	10.93898	8.128186	-8.18953	C	-2.12602	0.406105	-2.40948
151	C	8.20329	10.92907	-8.1276	C	8.189521	10.93894	-8.1282	C	1.416873	-1.66362	-2.40396
152	H	7.953713	7.162289	-11.2118	H	7.957867	7.143008	-11.2055	H	0.375938	-2.91964	-2.9844
153	H	11.21182	7.953713	-7.16229	H	11.20548	7.957866	-7.14303	H	-2.70861	1.130592	-2.99242
154	H	7.162289	11.21182	-7.95371	H	7.14302	11.20545	-7.95787	H	2.334 99	1.809518	-2.98636
155	H	7.919959	8.329228	-9.85912	H	7.930188	8.326597	-9.86913	H	0.694244	-1.18197	-3.07145
156	H	9.859115	7.919959	-8.32923	H	9.869148	7.930163	-8.32663	H	-1.35335	-0.00332	-3.07608
157	H	8.329228	9.859115	-7.91996	H	8.326609	9.869102	-7.9302	H	0.675645	1.205102	-3.07402
158	C	5.779236	8.663022	-11.8107	C	5.779905	8.584936	-11.8477	C	3.002444	-2.91104	-3.00069
159	C	11.81068	5.779236	-8.66302	C	11.84777	5.779906	-8.58494	C	-3.98728	-1.16388	-3.01648

160	C	8.663022	11.81068	-5.77924	C	8.584937	11.84771	-5.77991	C	0.995881	4.068873	-2.98713
161	C	4.760711	9.375842	-11.1451	C	4.738234	9.233569	-11.1519	C	-3.873765	-2.12514	-3.74139
162	C	11.14506	4.760711	-9.37584	C	11.15193	4.738228	-9.23358	C	-3.72208	-2.31152	-3.75028
163	C	9.375842	11.14506	-4.76071	C	9.233555	11.15185	-4.73823	C	-0.12627	4.423703	-3.72232
164	H	5.001681	10.29802	-10.6197	H	4.948083	10.14293	-10.5927	H	3.964175	-1.06647	-3.50958
165	H	10.61972	5.001681	-10.298	H	10.59277	4.94807	-10.143	H	-2.84666	-2.90945	-3.5071
166	H	10.29802	10.61972	-5.00168	H	10.14291	10.59267	-4.94807	H	-1.08399	3.964747	-3.48845
167	C	3.43342	8.904971	-11.1554	C	3.428528	8.713979	-11.1741	C	4.628555	-2.68108	-4.7618
168	C	11.15537	3.43342	-8.90497	C	11.17411	3.428524	-8.71399	C	-4.56531	-2.69948	-4.77898
169	C	8.904972	11.15537	-3.43342	C	8.713955	11.17404	-3.42853	C	-0.03347	5.359129	-4.74035
170	H	2.657283	9.466542	-10.6416	H	2.635407	9.223995	-10.6332	H	5.314678	-2.05751	-5.32355
171	H	10.64161	2.657283	-9.46654	H	10.63323	2.635397	-9.22401	H	-4.35308	-3.6056	-5.33502
172	H	9.466543	10.64161	-2.65728	H	9.22396	10.63314	-2.6354	H	-0.92072	5.636176	-5.29832
173	C	3.113152	7.716405	-11.8371	C	3.14736	7.540202	-11.8998	C	4.5145	-4.0298	-5.04751
174	C	11.83711	3.113152	-7.71641	C	11.89983	3.147364	-7.5402	C	-5.68096	-1.93912	-5.08021
175	C	7.716405	11.83711	-3.11315	C	7.54018	11.89978	-3.14738	C	1.18566	5.945522	-5.02927

176	H	2.088346	7.353254	-11.8498	H	2.137033	7.139291	-11.9212	H	5.109213	-4.46955	-5.83957
177	H	11.8498	2.088346	-7.35326	H	11.92129	2.137038	-7.13929	H	-6.34699	-2.24424	-5.87878
178	H	7.353255	11.8498	-2.08835	H	7.139262	11.92125	-2.13705	H	1.259975	6.683246	-5.81964
179	C	4.12664	7.001704	-12.5095	C	4.184255	6.888879	-12.6021	C	3.640387	-4.81967	-4.31524
180	C	12.50952	4.12664	-7.0017	C	12.60209	4.184267	-6.88887	C	-5.94823	-0.78725	-4.3551
181	C	7.001704	12.50952	-4.12664	C	6.888873	12.60206	-4.18428	C	2.312414	5.59078	-4.30225
182	H	3.881782	6.085678	-13.0421	H	3.972332	5.983363	-13.1655	H	3.549639	-5.8765	-4.53868
183	H	13.04208	3.881782	-6.08568	H	13.16547	3.972351	-5.98334	H	-6.82164	-0.19003	-4.59067
184	H	6.085678	13.04208	-3.88178	H	5.983359	13.16546	-3.97237	H	3.268427	6.049198	-4.52795
185	C	5.450824	7.469673	-12.496	C	5.491205	7.405263	-12.574	C	2.884389	-4.26526	-3.29879
186	C	12.49602	5.450824	-7.46967	C	12.57405	5.491215	-7.40526	C	-5.10545	-0.39724	-3.33041
187	C	7.469673	12.49602	-5.45082	C	7.405267	12.57402	-5.49122	C	2.221652	4.655112	-3.28811
188	H	6.233402	6.928535	-13.0237	H	6.290856	6.906267	-13.1165	H	2.19635	-4.87646	-2.72044
189	H	13.0237	6.233402	-6.92854	H	13.11654	6.290871	-6.90626	H	-5.30662	0.504494	-2.75795
190	H	6.928536	13.0237	-6.2334	H	6.906283	13.11653	-6.29088	H	3.099589	4.370279	-2.71407

Partial Ligand Exchange (Co_3YL_5L')

Aux1R – Partial Ligand Exchange

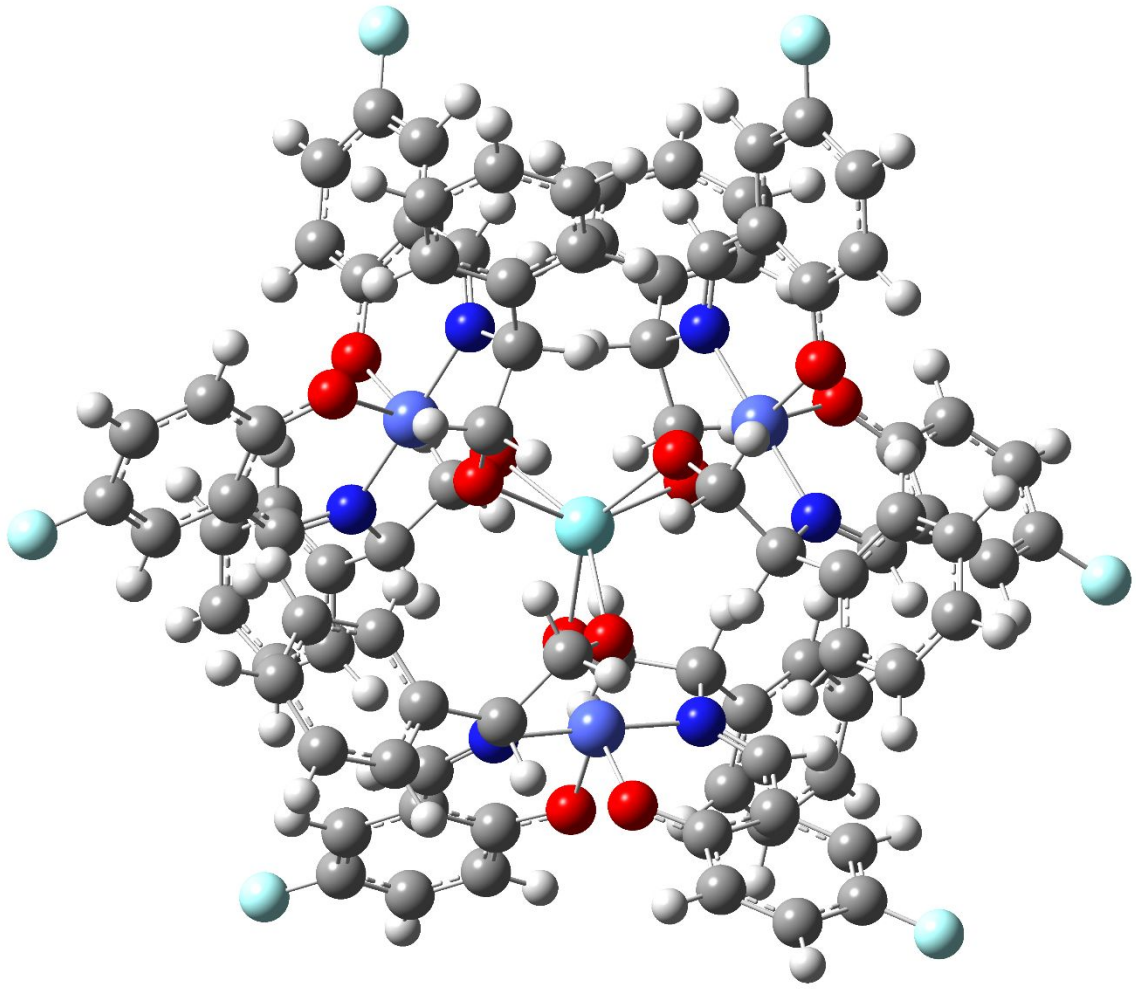

	B3LYP/SDD
**Electronic Energy (EE)**	-5779.138318
**EE + Zero-point Energy**	-5777.678200
**EE + Thermal Energy Correction**	-5777.578311
**EE + Thermal Enthalpy Correction**	-5777.577367
**EE + Thermal Free Energy Correction**	-5777.823489

	B3LYP/SDD			
Tag	Symbol	X	Y	Z
1	Y	10.4927	10.54856	-10.4649
2	Co	9.686479	8.64745	-13.1065
3	Co	13.25135	9.699592	-8.75958
4	Co	8.455742	13.10437	-9.68308
5	F	11.3966	1.9366	-13.4884

6	F	12.66773	11.43342	-2.12275
7	F	1.738903	13.23019	-11.4162
8	F	6.194377	11.067	-18.5898
9	F	18.38393	5.978217	-11.6026
10	F	9.924033	18.88464	-6.13719
11	O	8.923505	6.918817	-13.3065
12	O	13.55086	8.96073	-7.03415
13	O	6.716524	13.21989	-8.92895
14	O	10.53712	10.37055	-12.7499
15	O	12.77356	10.62681	-10.4117
16	O	10.20414	12.81861	-10.5276
17	O	9.823791	8.869626	-14.9884
18	O	15.10621	9.754438	-9.16852
19	O	8.563832	14.99167	-9.8735
20	O	9.529693	8.589694	-11.1596
21	O	11.31007	9.609266	-8.53752
22	O	8.516784	11.16421	-9.48052
23	N	11.51524	8.020717	-13.2139
24	N	13.50429	11.50582	-8.14752
25	N	7.829412	13.13462	-11.5184
26	N	7.852054	9.262331	-13.0128
27	N	13.03974	7.866171	-9.36358
28	N	9.04895	13.1008	-7.83657
29	C	9.57607	5.754735	-13.3465
30	C	13.31813	9.612768	-5.88909
31	C	5.553835	13.22049	-9.58513
32	C	8.798001	4.556637	-13.4153
33	C	13.20876	8.838628	-4.6915
34	C	4.352414	13.26192	-8.81045
35	H	7.717512	4.658022	-13.4407
36	H	13.30921	7.761211	-4.77652
37	H	4.450696	13.30196	-7.73009
38	C	9.395313	3.297619	-13.4592
39	C	12.99453	9.436398	-3.44953
40	C	3.094434	13.2624	-9.41142
41	H	8.800874	2.390226	-13.5136
42	H	12.91337	8.843954	-2.54286
43	H	2.184133	13.29646	-8.81993
44	C	10.8017	3.211123	-13.4411
45	C	12.89091	10.83934	-3.37934
46	C	3.012645	13.22725	-10.8179
47	C	11.6059	4.334004	-13.3854
48	C	13.00533	11.64282	-4.49933
49	C	4.138857	13.19617	-11.6187
50	H	12.68784	4.227171	-13.3898
51	H	12.94217	12.72371	-4.40147
52	H	4.035102	13.18392	-12.7008

53	C	11.00742	5.626306	-13.33
54	C	13.20877	11.04373	-5.77486
55	C	5.430473	13.18516	-11.0162
56	C	11.88449	6.775335	-13.3134
57	C	13.43533	11.91079	-6.9126
58	C	6.581876	13.18914	-11.8902
59	H	12.9509	6.561444	-13.4256
60	H	13.61206	12.9675	-6.69701
61	H	6.366479	13.27685	-12.9584
62	C	12.54743	9.120012	-13.287
63	C	14.11769	12.35454	-9.22865
64	C	8.927286	13.23016	-12.5506
65	H	12.99591	9.207568	-12.2892
66	H	9.07057	12.22359	-12.9644
67	C	11.75445	10.42327	-13.5484
68	C	13.54052	11.8526	-10.59
69	C	10.20974	13.59086	-11.7641
70	H	11.49366	10.50903	-14.606
71	H	14.37114	11.67194	-11.2833
72	H	10.23015	14.65694	-11.5263
73	H	12.35864	11.28988	-13.253
74	H	12.88334	12.61611	-11.0227
75	H	11.09358	13.33702	-12.3632
76	C	13.3167	8.635666	-15.6705
77	C	8.3357	15.55789	-13.4111
78	H	12.2702	8.557105	-15.9571
79	H	8.247136	15.8829	-12.3767
80	C	14.33319	8.467301	-16.6249
81	C	8.123631	16.463	-14.4639
82	H	14.07413	8.268349	-17.6623
83	H	7.880067	17.50011	-14.2453
84	C	15.68847	8.552026	-16.2427
85	C	8.223278	16.03152	-15.8032
86	H	16.47403	8.419558	-16.9828
87	H	8.0575	16.73338	-16.617
88	C	16.01893	8.800358	-14.8978
89	C	8.531579	14.68705	-16.0813
90	H	17.06073	8.856164	-14.5923
91	H	8.601569	14.34397	-17.1104
92	C	14.99939	8.965335	-13.9403
93	C	8.740877	13.77901	-15.0253
94	H	15.2617	9.148699	-12.8999
95	H	8.973917	12.73913	-15.2463
96	C	13.64264	8.889358	-14.3173
97	C	8.648878	14.20552	-13.6842
98	C	8.90935	9.402689	-15.8019
99	C	15.84115	8.808937	-9.75199

100	C	8.892107	15.87828	-8.93053
101	C	9.247927	9.542667	-17.1845
102	C	17.21911	9.096154	-10.0073
103	C	8.858225	17.26484	-9.27796
104	H	10.22717	9.197563	-17.5012
105	H	17.59674	10.06583	-9.69759
106	H	8.548048	17.52503	-10.2852
107	C	8.35641	10.09303	-18.1041
108	C	18.05721	8.165785	-10.62
109	C	9.200093	18.25752	-8.36
110	H	8.61688	10.19628	-19.1535
111	H	19.1039	8.385009	-10.81
112	H	9.169611	19.31001	-8.62639
113	C	7.088876	10.51396	-17.6544
114	C	17.5289	6.911574	-10.9877
115	C	9.583615	17.87689	-7.05875
116	C	6.70116	10.39392	-16.3332
117	C	16.20865	6.574001	-10.7567
118	C	9.62893	16.55246	-6.6643
119	H	5.708877	10.7161	-16.0274
120	H	15.84194	5.590527	-11.0401
121	H	9.921818	16.29826	-5.6488
122	C	7.607201	9.842151	-15.3812
123	C	15.33917	7.520292	-10.1402
124	C	9.287854	15.52916	-7.59479
125	C	7.147512	9.698096	-14.0184
126	C	13.97574	7.120083	-9.8809
127	C	9.319528	14.16105	-7.12971
128	H	6.099648	9.954733	-13.8405
129	H	13.73213	6.081368	-10.1197
130	H	9.562502	14.02725	-6.07266
131	C	7.207332	9.092298	-11.6586
132	C	11.68017	7.270485	-9.09127
133	C	9.014228	11.74225	-7.17933
134	H	7.205364	10.07675	-11.1737
135	H	11.12433	7.29064	-10.0376
136	H	10.03697	11.34267	-7.20193
137	C	8.165206	8.186349	-10.8487
138	C	10.97674	8.251849	-8.12334
139	C	8.147846	10.85887	-8.10751
140	H	8.023265	7.137744	-11.1214
141	H	11.30903	8.086525	-7.0958
142	H	7.086632	11.07238	-7.95711
143	H	7.971891	8.312145	-9.77645
144	H	9.890746	8.101902	-8.17348
145	H	8.336808	9.799757	-7.8922
146	C	5.789001	8.542824	-11.6981

147	C	11.70946	5.848069	-8.55258
148	C	8.500273	11.73976	-5.74703
149	C	4.752038	9.205283	-11.0094
150	C	10.94311	4.839645	-9.17252
151	C	9.26208	11.14552	-4.7207
152	H	4.960812	10.13466	-10.4832
153	H	10.36298	5.07336	-10.0635
154	H	10.237	10.72199	-4.95011
155	C	3.447199	8.675492	-10.9988
156	C	10.92578	3.531318	-8.65154
157	C	8.774359	11.10003	-3.40023
158	H	2.656338	9.199082	-10.4676
159	H	10.3339	2.762446	-9.14169
160	H	9.377554	10.6475	-2.61747
161	C	3.167476	7.478104	-11.6828
162	C	11.68178	3.219181	-7.50654
163	C	7.515095	11.64873	-3.09597
164	H	2.159681	7.06989	-11.6797
165	H	11.67364	2.208587	-7.10542
166	H	7.137617	11.6179	-2.07674
167	C	4.199229	6.813719	-12.3786
168	C	12.45727	4.221403	-6.8867
169	C	6.747874	12.24639	-4.11792
170	H	3.985513	5.891249	-12.9135
171	H	13.04958	3.981787	-6.00666
172	H	5.776347	12.67635	-3.8857
173	C	5.501308	7.340145	-12.3856
174	C	12.47083	5.527071	-7.40405
175	C	7.235535	12.29203	-5.43415
176	H	6.297767	6.838207	-12.9309
177	H	13.08083	6.298634	-6.9388
178	H	6.652811	12.76674	-6.22085
179	H	15.17187	12.06128	-9.20184
180	C	14.03324	13.85927	-9.01795
181	C	15.21331	14.57855	-8.72928
182	C	12.80905	14.5616	-9.11138
183	C	15.18208	15.97365	-8.54261
184	H	16.15988	14.04547	-8.65446
185	C	12.77646	15.95457	-8.92227
186	H	11.88889	14.02662	-9.33809
187	C	13.96023	16.6652	-8.63969
188	H	16.10039	16.51334	-8.32462
189	H	11.83276	16.48693	-8.99923
190	H	13.92768	17.74239	-8.49627

**Aux1S – Partial Ligand Exchange**
see Co_3YL_6

Aux6R – Partial Ligand Exchange

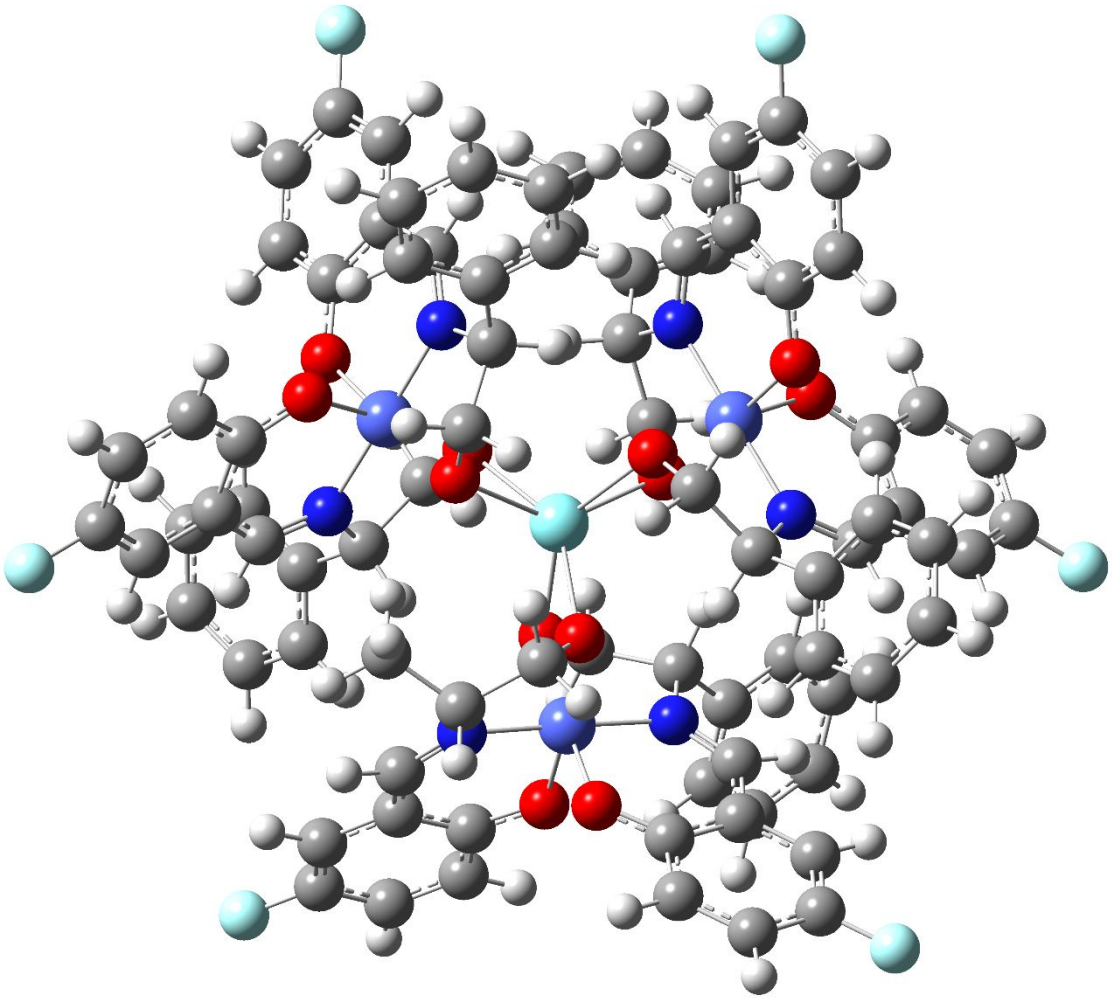

	B3LYP/SDD
**Electronic Energy (EE)**	-5587.433386
**EE + Zero-point Energy**	-5586.026784
**EE + Thermal Energy Correction**	-5585.930155
**EE + Thermal Enthalpy Correction**	-5585.929211
**EE + Thermal Free Energy Correction**	-5586.168032

	B3LYP/SDD			
Tag	Symbol	X	Y	Z
1	Y	10.53576	10.53092	-10.4651
2	Co	9.734929	8.648892	-13.1236
3	Co	13.23087	9.677891	-8.67201
4	Co	8.577125	13.14021	-9.69858
5	F	11.29855	1.907233	-13.5929
6	F	12.97348	11.23277	-1.94963
7	F	1.835821	13.51061	-11.2934
8	F	6.361451	11.12194	-18.6569
9	F	18.6067	6.152039	-11.2806
10	F	10.82524	18.78148	-6.34501
11	O	8.935255	6.939142	-13.342

12	O	13.4814	8.877617	-6.96695
13	O	6.859642	13.32273	-8.90736
14	O	10.61613	10.35151	-12.7483
15	O	12.80959	10.60868	-10.3308
16	O	10.28969	12.79398	-10.5735
17	O	9.905984	8.874094	-15.0027
18	O	15.09946	9.802785	-8.9778
19	O	8.757104	15.02213	-9.88969
20	O	9.551074	8.587995	-11.1808
21	O	11.2864	9.54246	-8.53631
22	O	8.569436	11.19718	-9.49226
23	N	11.55116	7.981461	-13.2073
24	N	13.41918	11.48323	-8.01881
25	N	7.91525	13.19357	-11.5201
26	N	7.913585	9.30439	-13.0536
27	N	13.09251	7.858791	-9.32706
28	N	9.224727	13.10635	-7.87258
29	C	9.562119	5.76213	-13.3993
30	C	13.34485	9.500917	-5.79425
31	C	5.685057	13.36607	-9.53979
32	C	8.75829	4.583123	-13.4975
33	C	13.28355	8.69627	-4.61284
34	C	4.501529	13.45339	-8.74136
35	H	7.680639	4.709323	-13.5306
36	H	13.34524	7.619281	-4.73308
37	H	4.622773	13.49109	-7.66329
38	C	9.327874	3.312226	-13.5587
39	C	13.15968	9.264192	-3.3457
40	C	3.232548	13.49853	-9.31656
41	H	8.714024	2.419461	-13.6351
42	H	13.11339	8.649379	-2.45156
43	H	2.336323	13.56662	-8.70679
44	C	10.73193	3.193937	-13.5287
45	C	13.10202	10.66771	-3.23226
46	C	3.120776	13.46299	-10.7211
47	C	11.56034	4.297135	-13.4448
48	C	13.17312	11.4979	-4.33539
49	C	4.228433	13.38915	-11.5444
50	H	12.63958	4.166159	-13.439
51	H	13.14273	12.57678	-4.2044
52	H	4.10233	13.37774	-12.6242
53	C	10.99013	5.601486	-13.3718
54	C	13.28485	10.92952	-5.63724
55	C	5.531071	13.33259	-10.9683
56	C	11.89271	6.729413	-13.3235
57	C	13.43172	11.82761	-6.76215
58	C	6.663	13.29294	-11.8664

59	H	12.95526	6.492361	-13.4248
60	H	13.60693	12.87678	-6.50818
61	H	6.428298	13.38563	-12.9302
62	C	12.60822	9.058574	-13.2477
63	C	13.8421	12.47012	-9.0758
64	C	8.995778	13.24382	-12.5737
65	H	13.03389	9.131277	-12.2384
66	H	9.09394	12.23145	-12.9868
67	C	11.84913	10.37923	-13.5222
68	C	13.68531	11.7624	-10.4569
69	C	10.3067	13.55661	-11.8129
70	H	11.61177	10.47213	-14.5847
71	H	14.66338	11.43174	-10.8194
72	H	10.37471	14.62311	-11.5845
73	H	12.46694	11.23153	-13.2126
74	H	13.25132	12.46012	-11.1859
75	H	11.16756	13.25929	-12.4251
76	C	13.42772	8.576428	-15.6142
77	C	8.460252	15.58723	-13.4273
78	H	12.38753	8.525062	-15.9288
79	H	8.388049	15.91314	-12.3918
80	C	14.46423	8.391905	-16.5439
81	C	8.265505	16.49933	-14.4775
82	H	14.22731	8.208062	-17.5892
83	H	8.051482	17.54226	-14.2557
84	C	15.81086	8.440906	-16.1264
85	C	8.343803	16.06726	-15.8181
86	H	16.61187	8.295931	-16.8472
87	H	8.191115	16.7745	-16.6298
88	C	16.1124	8.670354	-14.7712
89	C	8.613548	14.7153	-16.1
90	H	17.14699	8.699315	-14.4388
91	H	8.666897	14.37184	-17.13
92	C	15.07258	8.852043	-13.8388
93	C	8.805441	13.8003	-15.0466
94	H	15.31226	9.021896	-12.7908
95	H	9.008973	12.75498	-15.2708
96	C	13.72458	8.810977	-14.2509
97	C	8.734052	14.22708	-13.7045
98	C	9.011018	9.419373	-15.8294
99	C	15.89001	8.883736	-9.53199
100	C	9.259837	15.87543	-8.99381
101	C	9.369901	9.549716	-17.2078
102	C	17.27235	9.209915	-9.70177
103	C	9.348796	17.25393	-9.36354
104	H	10.34729	9.187186	-17.5105
105	H	17.60432	10.18348	-9.35438

106	H	8.992236	17.53543	-10.3495
107	C	8.49999	10.11247	-18.1405
108	C	18.16822	8.31218	-10.2803
109	C	9.867229	18.21273	-8.49462
110	H	8.775835	10.20843	-19.1866
111	H	19.21805	8.56079	-10.4059
112	H	9.933643	19.25874	-8.77934
113	C	7.233996	10.55609	-17.7085
114	C	17.69408	7.052402	-10.6995
115	C	10.30564	17.80725	-7.21819
116	C	6.827027	10.44627	-16.3921
117	C	16.37184	6.677806	-10.5518
118	C	10.23384	16.49161	-6.80068
119	H	5.836722	10.78664	-16.0999
120	H	16.04767	5.691505	-10.8744
121	H	10.5659	16.22033	-5.80149
122	C	7.710989	9.881682	-15.4269
123	C	15.44334	7.590202	-9.97066
124	C	9.714776	15.50027	-7.68368
125	C	7.230955	9.750646	-14.0697
126	C	14.07776	7.148362	-9.80058
127	C	9.627649	14.14353	-7.19471
128	H	6.186296	10.02888	-13.9063
129	H	13.87816	6.108294	-10.0721
130	H	9.903559	13.99813	-6.14682
131	C	7.249618	9.151317	-11.7064
132	C	11.73359	7.230034	-9.13809
133	C	9.114356	11.76058	-7.19755
134	H	7.270558	10.13485	-11.2196
135	H	11.21937	7.270683	-10.1071
136	H	10.11358	11.30491	-7.21441
137	C	8.17326	8.217589	-10.8881
138	C	10.96729	8.167564	-8.17348
139	C	8.202948	10.90928	-8.11341
140	H	8.008289	7.173797	-11.1662
141	H	11.26423	7.981504	-7.13852
142	H	7.15258	11.16422	-7.95282
143	H	7.970023	8.345268	-9.81777
144	H	9.888277	7.995437	-8.27389
145	H	8.354488	9.844005	-7.8983
146	C	5.8163	8.644227	-11.761
147	C	11.76391	5.791846	-8.64295
148	C	8.606852	11.81082	-5.764
149	C	4.791696	9.340084	-11.0868
150	C	11.04126	4.791813	-9.32577
151	C	9.327303	11.17369	-4.73299
152	H	5.022419	10.26412	-10.5603

153	H	10.49532	5.044419	-10.2328
154	H	10.26844	10.67723	-4.95979
155	C	3.471317	8.850125	-11.0904
156	C	11.02245	3.467998	-8.84556
157	C	8.840545	11.17683	-3.41135
158	H	2.690424	9.399019	-10.5702
159	H	10.46459	2.706114	-9.38409
160	H	9.409766	10.68738	-2.62524
161	C	3.163791	7.659328	-11.774
162	C	11.73271	3.132034	-7.67823
163	C	7.626269	11.82061	-3.10998
164	H	2.144194	7.281636	-11.7817
165	H	11.72322	2.109624	-7.3083
166	H	7.250395	11.82768	-2.08971
167	C	4.183268	6.961501	-12.4551
168	C	12.46359	4.126286	-6.99461
169	C	6.902387	12.46301	-4.13639
170	H	3.948258	6.04383	-12.9893
171	H	13.01974	3.868843	-6.09621
172	H	5.966198	12.96626	-3.90619
173	C	5.500663	7.448384	-12.448
174	C	12.47862	5.44727	-7.47151
175	C	7.38742	12.45817	-5.45444
176	H	6.287881	6.920389	-12.9821
177	H	13.05327	6.213837	-6.95583
178	H	6.83959	12.96429	-6.24651
179	C	13.07858	13.80866	-9.00879
180	H	13.49936	14.49711	-9.7537
181	H	12.01793	13.66793	-9.23617
182	H	13.17758	14.29957	-8.03275
183	H	14.91145	12.65666	-8.90862

Aux6S – Partial Ligand Exchange
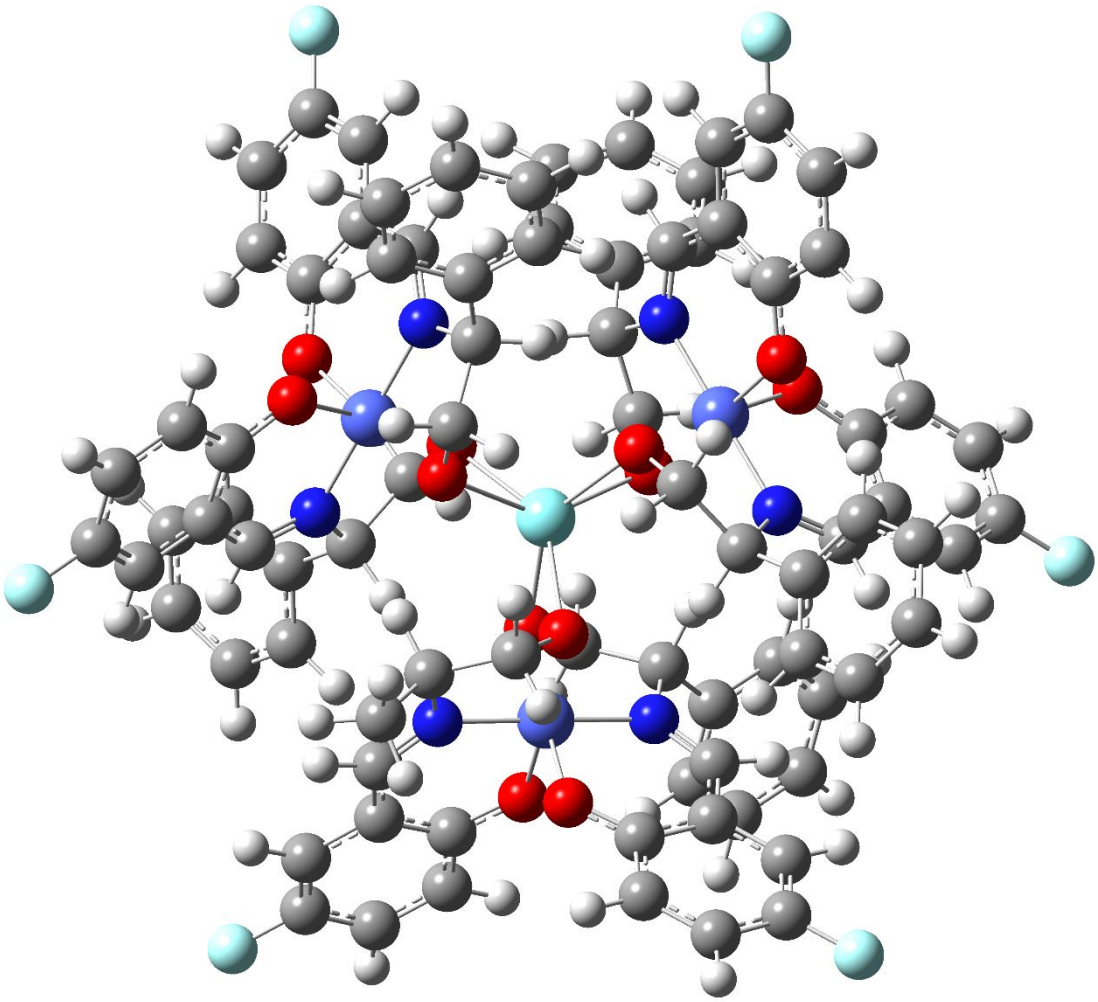

	B3LYP/SDD	B3LYP/SDD, pcm=chloroform	B3LYP/SDD, pcm=methanol
**Electronic Energy (EE)**	-5587.439318	-5587.476801	-5587.4962380
**EE + Zero-point Energy**	-5586.032902	-5586.070427	-5586.0905000
**EE + Thermal Energy Correction**	-5585.936155	-5586.974014	-5585.9940220
**EE + Thermal Enthalpy Correction**	-5585.935211	-5585.973070	-5585.9930780
**EE + Thermal Free Energy Correction**	-5586.175191	-5586.209973	-5586.2302560

	B3LYP/SDD	B3LYP/SDD, pcm=chloroform	B3LYP/SDD, pcm=methanol

Tag	Symbol	X	Y	Z	Symbol	X	Y	Z	Symbol	X	Y	Z
1	Y	10.47891	10.4871	-10.5016	Y	10.4984	10.50001	-10.509	Y	10.50066	10.50039	-10.4993
2	Co	9.699165	8.617782	-13.164	Co	9.694773	8.624004	-13.1682	Co	9.697129	8.618776	-13.1662
3	Co	13.13153	9.719318	-8.61554	Co	13.14997	9.708978	-8.62238	Co	13.15496	9.689979	-8.60845
4	Co	8.614733	13.14756	-9.70832	Co	8.625589	13.15522	-9.69853	Co	8.6193	13.16696	-9.70062
5	F	11.23595	1.871267	-13.6284	F	11.2832	1.87429	-13.4003	F	11.29578	1.866297	-13.1782
6	F	13.66019	11.26853	-1.87708	F	13.29315	11.28469	-1.86956	F	13.19307	11.23352	-1.84324
7	F	1.859865	13.66568	-11.1944	F	1.861091	13.40611	-11.2229	F	1.861332	13.16957	-11.2763
8	F	6.399229	11.13655	-18.7191	F	6.421287	11.30251	-18.6679	F	6.520347	11.54897	-18.5937
9	F	18.73178	6.469107	-11.0906	F	18.69323	6.495339	-11.278	F	18.59042	6.549459	-11.5582
10	F	11.29389	18.62773	-6.41176	F	11.58775	18.55417	-6.51229	F	11.71798	18.54476	-6.60803
11	O	8.892763	6.913271	-13.3928	O	8.902159	6.908236	-13.3963	O	8.909937	6.895612	-13.4006
12	O	13.34932	8.914098	-6.90919	O	13.37253	8.913283	-6.90729	O	13.38422	8.886303	-6.8918
13	O	6.916849	13.38151	-8.88894	O	6.917474	13.38935	-8.88949	O	6.898601	13.40443	-8.90829
14	O	10.58183	10.31734	-12.7812	O	10.56693	10.32533	-12.8003	O	10.56864	10.31639	-12.8008
15	O	12.75769	10.59132	-10.3203	O	12.78738	10.57497	-10.3266	O	12.79894	10.56591	-10.3049

16	O	10.3051	12.76311	-10.6061	O	10.31654	12.78759	-10.5876	O	10.31317	12.80065	-10.5764
17	O	9.889118	8.843713	-15.0405	O	9.866421	8.839007	-15.0527	O	9.858552	8.832794	-15.0565
18	O	15.00483	9.920354	-8.82307	O	15.03196	9.894408	-8.82225	O	15.04396	9.852168	-8.81356
19	O	8.855686	15.02395	-9.8822	O	8.853257	15.03951	-9.85666	O	8.83658	15.05746	-9.86002
20	O	9.497541	8.550732	-11.2228	O	9.501554	8.558496	-11.2328	O	9.501567	8.550044	-11.2354
21	O	11.18989	9.505 15	-8.56373	O	11.21474	9.501466	-8.56775	O	11.22322	9.493649	-8.5476
22	O	8.538545	11.20445	-9.52105	O	8.552908	11.21878	-9.51326	O	8.548115	11.23577	-9.50231
23	N	11.51352	7.943402	-13.228	N	11.51311	7.960409	-13.2322	N	11.51509	7.954533	-13.2248
24	N	13.19268	11.5419	-7.94694	N	13.19919	11.53085	-7.95888	N	13.21295	11.50657	-7.93206
25	N	7.927343	13.2297	-11.5173	N	7.944528	13.23056	-11.5099	N	7.948635	13.22529	-11.516
26	N	7.882756	9.287406	-13.1076	N	7.877606	9.290237	-13.1044	N	7.883068	9.291991	-13.1014
27	N	13.09177	7.903217	-9.28557	N	13.10389	7.892807	-9.29298	N	13.09264	7.879805	-9.29357
28	N	9.296205	13.06886	-7.89751	N	9.314006	13.07304	-7.8914	N	9.304106	13.10035	-7.89178
29	C	9.515304	5.733925	-13.4471	C	9.5324	5.730427	-13.3866	C	9.53992	5.718088	-13.3338
30	C	13.42319	9.538732	-5.73254	C	13.34389	9.540 92	-5.72846	C	13.32648	9.506614	-5.70871
31	C	5.733442	13.44824	-9.50244	C	5.733683	13.38322	-9.50839	C	5.719 01	13.33471	-9.53396

32	C	8.707286	4.558898	-13.55581	C	8.732824	4.546146	-13.45473	C	8.742753	4.530769	-13.37552
33	C	13.53856	8.732956	-4.55624	C	13.40787	8.739827	-4.54482	C	13.36958	8.700046	-4.52789
34	C	4.564844	13.56156	-8.6854	C	4.556936	13.45403	-8.69767	C	4.534288	13.37634	-8.73288
35	H	7.630705	4.690427	-13.6031	H	7.655381	4.664949	-13.5145	H	7.665829	4.64382	-13.4516
36	H	13.57217	7.655697	-4.68561	H	13.47708	7.663254	-4.66525	H	13.44124	7.623737	-4.64965
37	H	4.703979	13.59854	-7.60949	H	4.685529	13.50843	-7.62126	H	4.650841	13.45521	-7.65652
38	C	9.271509	3.285575	-13.6156	C	9.305619	3.274398	-13.4589	C	9.316753	3.259951	-13.3216
39	C	13.61483	9.299641	-3.28505	C	13.38869	9.309746	-3.2718	C	13.3235	9.264138	-3.25213
40	C	3.287983	13.63094	-9.2402	C	3.279881	13.45872	-9.25849	C	3.261589	13.31956	-9.30239
41	H	8.654457	2.395842	-13.7008	H	8.692558	2.379549	-13.5121	H	8.704356	2.363582	-13.3505
42	H	13.70407	8.684171	-2.39464	H	13.43698	8.695392	-2.37758	H	13.35368	8.644694	-2.36066
43	H	2.403208	13.71786	-8.61629	H	2.390926	13.51163	-8.63689	H	2.367283	13.34851	-8.68698
44	C	10.67465	3.160645	-13.569	C	10.70843	3.165651	-13.3955	C	10.71792	3.157497	-13.2309
45	C	13.5821	10.70336	-3.16374	C	13.31004	10.7114	-3.16166	C	13.23841	10.66452	-3.13895
46	C	3.152777	13.59429	-10.6427	C	3.15781	13.39838	-10.6603	C	3.154442	13.2255	-10.703
47	C	11.50722	4.25969	-13.4733	C	11.53527	4.271702	-13.3348	C	11.5435	4.266066	-13.1962

48	C	13.48133	11.53347	-4.2643	C	13.25444	11.53956	-4.26731	C	13.20201	11.49865	-4.2415
49	C	4.245256	13.49701	-11.4836	C	4.255967	13.33807	-11.4976	C	4.260222	13.19062	-11.5323
50	H	12.58569	4.123759	-13.4543	H	12.61444	4.146572	-13.3	H	12.62198	4.145721	-13.136
51	H	13.47097	12.6123	-4.12929	H	13.2071	12.61797	-4.13941	H	13.14591	12.57634	-4.11217
52	H	4.10164	13.48608	-12.5612	H	4.120694	13.30595	-12.5757	H	4.136286	13.12794	-12.6102
53	C	10.94233	5.56673	-13.4043	C	10.96049	5.575561	-13.322	C	10.96712	5.567883	-13.2406
54	C	13.3947	10.96628	-5.56922	C	13.26382	10.96785	-5.57227	C	13.23957	10.9325	-5.54768
55	C	5.555767	13.41599	-10.9285	C	5.565141	13.3223	-10.9351	C	5.563945	13.23821	-10.9605
56	C	11.85011	6.689854	-13.3432	C	11.86222	6.707265	-13.2976	C	11.86861	6.701658	-13.2439
57	C	13.32511	11.87352	-6.69401	C	13.24439	11.87348	-6.70321	C	13.23781	11.84478	-6.6751
58	C	6.672093	13.35381	-11.8444	C	6.688294	13.29862	-11.8474	C	6.69464	13.24186	-11.8658
59	H	12.91256	6.44749	-13.432	H	12.92634	6.469181	-13.3695	H	12.93379	6.463022	-13.2909
60	H	13.41468	12.93336	-6.44201	H	13.29324	12.93564	-6.45189	H	13.27748	12.90535	-6.41803
61	H	6.422729	13.44992	-12.9046	H	6.440469	13.37302	-12.9092	H	6.452506	13.28677	-12.9303
62	C	12.57513	9.01677	-13.2512	C	12.56791	9.039481	-13.2828	C	12.56996	9.032687	-13.2914
63	C	13.22101	12.59179	-9.0267	C	13.26682	12.57943	-9.03972	C	13.27835	12.56237	-9.00676

64	C	8.992738	13.25142	-12.58 66	C	9.013548	13.27849	-12.57 49	C	9.023511	13.29102	-12.57 41
65	H	12.98571	9.090384	-12.23 55	H	12.9986	9.117725	-12.27 58	H	13.01667	9.108881	-12.29 18
66	H	9.057181	12.23737	-13.00 28	H	9.084005	12.27219	-13.00 84	H	9.097786	12.29139	-13.02 09
67	C	11.82528	10.33972	-13.53 89	C	11.80792	10.35818	-13.56 2	C	11.80868	10.35237	-13.56 23
68	C	13.50967	11.839	-10.34 17	C	13.54192	11.82265	-10.35 44	C	13.5532	11.81407	-10.32 53
69	C	10.32278	13.52712	-11.84 52	C	10.34025	13.55137	-11.82 7	C	10.34581	13.56116	-11.81 69
70	H	11.60262	10.4319	-14.60 46	H	11.5829	10.4546	-14.62 71	H	11.58436	10.4547	-14.62 73
71	H	14.57803	11.61887	-10.43 06	H	14.60919	11.6019	-10.45 38	H	14.6209	11.59539	-10.42 75
72	H	10.42262	14.59116	-11.61 72	H	10.44284	14.61575	-11.60 1	H	10.44983	14.62625	-11.59 38
73	H	12.44253	11.18948	-13.22 13	H	12.41875	11.21124	-13.24 18	H	12.41897	11.20424	-13.23 86
74	H	13.18918	12.44849	-11.19 68	H	13.21619	12.4295	-11.20 88	H	13.22774	12.42512	-11.17 64
75	H	11.16553	13.20731	-12.47 08	H	11.18626	13.22933	-12.44 64	H	11.19567	13.23697	-12.42 96
76	C	13.43418	8.522105	-15.60 14	C	13.38338	8.529775	-15.64 69	C	13.34414	8.508142	-15.66 71
77	C	8.502395	15.60854	-13.42 87	C	8.500289	15.64555	-13.37 92	C	8.494122	15.66619	-13.34 68
78	H	12.3996	8.465581	-15.93 33	H	12.34352	8.460422	-15.95 84	H	12.29916	8.433748	-15.95 91
79	H	8.450916	15.93414	-12.39 18	H	8.437743	15.95364	-12.33 79	H	8.419687	15.95748	-12.30 16

80	C	14.48657	8.335969	-16.5127	C	14.41866	8.348817	-16.5797	C	14.36229	8.327261	-16.6191
81	C	8.315792	16.52733	-14.4745	C	8.313611	16.58215	-14.41	C	8.311388	16.61848	-14.3643
82	H	14.26754	8.144906	-17.5607	H	14.17957	8.145887	-17.6209	H	14.10405	8.116619	-17.6541
83	H	8.129065	17.57445	-14.248	H	8.112927	17.62256	-14.166	H	8.099136	17.65297	-14.1054
84	C	15.82594	8.393141	-16.0735	C	15.76685	8.42896	-16.17	C	15.71787	8.420242	-16.2365
85	C	8.36697	16.09658	-15.8169	C	8.385074	16.17711	-15.7601	C	8.404649	16.23683	-15.7201
86	H	16.63927	8.247098	-16.7802	H	16.56675	8.288554	-16.8928	H	16.50454	8.281096	-16.9739
87	H	8.220686	16.80907	-16.6251	H	8.240262	16.90281	-16.5566	H	8.264128	16.97445	-16.5063
88	C	16.10437	8.633146	-14.7152	C	16.07049	8.686669	-14.8195	C	16.04535	8.691016	-14.8938
89	C	8.601545	14.73936	-16.105	C	8.639921	14.82752	-16.0701	C	8.677505	14.89475	-16.0485
90	H	17.13328	8.669946	-14.3663	H	17.10613	8.744161	-14.4942	H	17.08667	8.760799	-14.5896
91	H	8.634237	14.39719	-17.1363	H	8.690967	14.50587	-17.1072	H	8.74753	14.59133	-17.09
92	C	15.04873	8.816578	-13.801	C	15.0315	8.863201	-13.8843	C	15.02326	8.866599	-13.9396
93	C	8.785277	13.81769	-15.056	C	8.822 08	13.88849	-15.0355	C	8.854884	13.94024	-15.027
94	H	15.27019	8.997104	-12.7509	H	15.27249	9.057319	-12.841	H	15.28278	9.071058	-12.903
95	H	8.962138	12.76851	-15.2849	H	9.014062	12.84593	-15.2815	H	9.061033	12.90414	-15.2872

96	C	13.70788	8.766028	-14.23 49	C	13.68201	8.789519	-14.28 85	C	13.66655	8.779465	-14.31 62
97	C	8.740534	14.243	-13.71 22	C	8.757031	14.28792	-13.68 43	C	8.767588	14.31596	-13.67
98	C	9.006533	9.399574	-15.87 32	C	8.998593	9.442203	-15.86 94	C	9.019281	9.500 84	-15.85 46
99	C	15.84876	9.046274	-9.372 45	C	15.85933	9.037 4	-9.423 78	C	15.84358	9.022065	-9.489 31
100	C	9.448925	15.83785	-9.006 02	C	9.524 03	15.83085	-9.014 96	C	9.543 89	15.84404	-9.041 61
101	C	9.378854	9.530159	-17.24 81	C	9.371 16	9.596019	-17.24 22	C	9.404816	9.692568	-17.21 9
102	C	17.22194	9.430 93	-9.486 74	C	17.22962	9.424185	-9.566 89	C	17.20676	9.414094	-9.677 54
103	C	9.605446	17.21142	-9.372 87	C	9.715426	17.19 72	-9.393 55	C	9.755195	17.20245	-9.437 03
104	H	10.35553	9.159215	-17.54 29	H	10.33 89	9.212931	-17.55 06	H	10.36242	9.292855	-17.53 78
105	H	17.50168	10.40868	-9.106 84	H	17.52386	10.39375	-9.176 82	H	17.52028	10.37 03	-9.269 62
106	H	9.225464	17.52032	-10.34 17	H	9.307333	17.52101	-10.34 6	H	9.337671	17.52715	-10.38 5
107	C	8.522919	10.10405	-18.18 68	C	8.526251	10.21074	-18.16 61	C	8.585726	10.36872	-18.12 41
108	C	18.17245	8.584411	-10.05 46	C	18.16501	8.590679	-10.17 97	C	18.11441	8.603 6	-10.36 06
109	C	10.21603	18.13155	-8.522 36	C	10.39744	18.09 64	-8.573 88	C	10.47295	18.09503	-8.639 63
110	H	8.809 09	10.20048	-19.23 01	H	8.815625	10.32468	-19.20 67	H	8.886972	10.51269	-19.15 75
111	H	19.21466	8.878285	-10.13 91	H	19.20362	8.889835	-10.28 58	H	19.14689	8.909048	-10.50 23

112	H	10.33353	19.17353	-8.8053	H	10.5404	19.13186	-8.86846	H	10.63201	19.12391	-8.94863
113	C	7.257487	10.55903	-17.7647	C	7.274211	10.68262	-17.7264	C	7.348242	10.86456	-17.6716
114	C	17.76442	7.317183	-10.519	C	17.7396	7.336501	-10.66	C	17.66554	7.368708	-10.8668
115	C	10.68134	17.69226	-7.26663	C	10.89995	17.63813	-7.34062	C	10.99133	17.63568	-7.41406
116	C	6.837612	10.44938	-16.4524	C	6.852816	10.55483	-16.4161	C	6.914444	10.70341	-16.3687
117	C	16.45488	6.885765	-10.4239	C	16.4326	6.90195	-10.5426	C	16.36381	6.929395	-10.7099
118	C	10.54704	16.38086	-6.85156	C	10.73731	16.33356	-6.91307	C	10.81127	16.33835	-6.97018
119	H	5.848174	10.79876	-16.168	H	5.873194	10.91963	-16.1182	H	5.947623	11.09189	-16.0595
120	H	16.1822	5.895258	-10.7797	H	16.14642	5.921199	-10.9136	H	16.05773	5.965009	-11.1069
121	H	10.8999	16.08461	-5.86662	H	11.12377	16.02295	-5.94561	H	11.21622	16.02474	-6.01151
122	C	7.707266	9.873086	-15.4809	C	7.711683	9.9372	-15.4615	C	7.746884	10.02279	-15.4347
123	C	15.47072	7.745605	-9.85377	C	15.46604	7.749022	-9.92701	C	15.42693	7.753134	-10.0226
124	C	9.934356	15.42753	-7.71671	C	10.05114	15.40377	-7.7469	C	10.0872	15.41741	-7.78034
125	C	7.213643	9.743438	-14.1285	C	7.215203	9.785708	-14.1104	C	7.237229	9.837463	-14.0914
126	C	14.12105	7.242337	-9.73594	C	14.1184	7.240439	-9.78506	C	14.08469	7.238937	-9.84148
127	C	9.78025	14.07645	-7.2279	C	9.862724	14.0602	-7.24301	C	9.879804	14.08175	-7.25915

128	H	6.170846	10.03325	-13.9735	H	6.179627	10.09472	-13.9475	H	6.210468	10.17059	-13.9219
129	H	13.97524	6.199184	-10.0293	H	13.96604	6.204631	-10.0988	H	13.91795	6.213559	-10.1804
130	H	10.07577	13.90978	-6.1883	H	10.19288	13.88579	-6.21565	H	10.22403	13.91067	-6.23622
131	C	7.207994	9.141497	-11.7648	C	7.203656	9.131984	-11.7627	C	7.20098	9.117182	-11.7659
132	C	11.75276	7.219246	-9.1504	C	11.76645	7.208719	-9.14543	C	11.75827	7.195016	-9.12285
133	C	9.13216	11.72603	-7.22726	C	9.130267	11.73729	-7.21244	C	9.118438	11.76988	-7.20295
134	H	7.239301	10.12431	-11.2762	H	7.218405	10.11573	-11.2758	H	7.199436	10.09906	-11.2759
135	H	11.27167	7.245895	-10.1371	H	11.28613	7.221728	-10.1326	H	11.26999	7.194493	-10.1055
136	H	10.10967	11.22688	-7.25584	H	10.10714	11.23655	-7.21574	H	10.09722	11.27366	-7.1969
137	C	8.11362	8.19291	-10.9431	C	8.122283	8.19724	-10.9391	C	8.123547	8.188248	-10.94
138	C	10.91685	8.119232	-8.20873	C	10.92935	8.118592	-8.21361	C	10.92804	8.113679	-8.19307
139	C	8.178834	10.91651	-8.13903	C	8.191845	10.9209	-8.13448	C	8.186571	10.94412	-8.12312
140	H	7.940108	7.152434	-11.228	H	7.950 03	7.152835	-11.2114	H	7.952005	7.142 07	-11.2071
141	H	11.19512	7.947555	-7.16611	H	11.19636	7.944642	-7.16806	H	11.19211	7.937917	-7.14682
142	H	7.139965	11.20587	-7.96351	H	7.148447	11.19555	-7.96019	H	7.141154	11.21391	-7.9514
143	H	7.903147	8.317181	-9.87347	H	7.922387	8.330762	-9.86897	H	7.924789	8.325401	-9.87022

144	H	9.849827	7.901163	-8.34169	H	9.861675	7.911098	-8.35489	H	9.858937	7.914357	-8.33432
145	H	8.296996	9.844603	-7.93662	H	8.322569	9.849475	-7.93944	H	8.321553	9.874464	-7.92245
146	C	5.767433	8.657378	-11.8291	C	5.771872	8.620935	-11.8295	C	5.776543	8.587877	-11.8503
147	C	11.82311	5.779642	-8.66395	C	11.84011	5.776466	-8.63634	C	11.84377	5.769497	-8.59644
148	C	8.643417	11.79313	-5.78835	C	8.608944	11.82211	-5.78523	C	8.588033	11.86664	-5.77978
149	C	4.748778	9.372508	-11.1661	C	4.745195	9.295599	-11.1364	C	4.735098	9.239492	-11.1569
150	C	11.16939	4.755 98	-9.38041	C	11.15364	4.746924	-9.31339	C	11.14903	4.729362	-9.24876
151	C	9.344437	11.11566	-4.76937	C	9.255572	11.11277	-4.75154	C	9.231078	11.17124	-4.73426
152	H	4.990513	10.29419	-10.6402	H	4.972 68	10.20872	-10.59	H	4.945836	10.14928	-10.5988
153	H	10.64654	4.993	-10.305	H	10.60795	4.972477	-10.2274	H	10.58965	4.941834	-10.1574
154	H	10.25888	10.57648	-5.00959	H	10.15 5	10.54065	-4.97028	H	10.13 52	10.60248	-4.94116
155	C	3.420 5	8.904481	-11.1796	C	3.427251	8.797 54	-11.145	C	3.424424	8.722 37	-11.1803
156	C	11.18819	3.428643	-8.9099	C	11.16795	3.428566	-8.81657	C	11.17258	3.417822	-8.73385
157	C	8.871775	11.13194	-3.44276	C	8.747058	11.13829	-3.43783	C	8.712632	11.20639	-3.42437
158	H	2.644306	9.467651	-10.6676	H	2.645419	9.328316	-10.6077	H	2.631478	9.234691	-10.6413
159	H	10.68357	2.648526	-9.47425	H	10.63588	2.644496	-9.34931	H	10.63253	2.625948	-9.24671

160	H	9.423865	10.60854	-2.66624	H	9.256121	10.58818	-2.65048	H	9.218122	10.6657	-2.6282
161	C	3.099411	7.716466	-11.8619	C	3.123401	7.61952	-11.8538	C	3.142067	7.548088	-11.9047
162	C	11.86651	3.113572	-7.71798	C	11.87576	3.127189	-7.63735	C	11.8986	3.13322	-7.56107
163	C	7.693546	11.83195	-3.1236	C	7.58698	11.88062	-3.14524	C	7.545842	11.94476	-3.14693
164	H	2.07389	7.355432	-11.877	H	2.106408	7.235331	-11.8651	H	2.131003	7.14909	-11.9271
165	H	11.88577	2.088774	-7.35508	H	11.89155	2.109866	-7.25418	H	11.92113	2.121489	-7.16375
166	H	7.329017	11.84926	-2.09934	H	7.194934	11.90512	-2.13146	H	7.145858	11.97616	-2.13648
167	C	4.113022	6.999486	-12.5317	C	4.145116	6.942585	-12.5535	C	4.178748	6.893753	-12.6045
168	C	12.52686	4.132314	-6.99951	C	12.56871	4.151863	-6.9579	C	12.59976	4.168572	-6.90607
169	C	6.991126	12.51662	-4.13747	C	6.938483	12.5967	-4.17406	C	6.900347	12.64666	-4.18772
170	H	3.867535	6.083862	-13.0647	H	3.91465	6.034527	-13.1055	H	3.965933	5.987833	-13.1669
171	H	13.05665	3.89151	-6.08081	H	13.11986	3.923362	-6.04885	H	13.16331	3.953997	-6.00126
172	H	6.083319	13.06341	-3.89331	H	6.044386	13.17444	-3.95222	H	6.000242	13.21972	-3.97863
173	C	5.438174	7.46464	-12.5151	C	5.460186	7.437534	-12.5399	C	5.486625	7.407702	-12.5753
174	C	12.50487	5.45653	-7.46713	C	12.54933	5.46736	-7.45158	C	12.57029	5.477364	-7.41776
175	C	7.460651	12.497	-5.46105	C	7.443393	12.56603	-5.48509	C	7.415431	12.60573	-5.49487

176	H	6.220837	6.921695	-13.0408	H	6.247604	6.921253	-13.0843	H	6.286044	6.906404	-13.116
177	H	13.02313	6.243348	-6.92303	H	13.08837	6.257219	-6.93333	H	13.11163	6.276191	-6.91622
178	H	6.929539	13.03368	-6.24432	H	6.949178	13.12137	-6.27894	H	6.920958	13.14737	-6.29788
179	C	14.236	13.72345	-8.78812	C	14.31522	13.67718	-8.78836	C	14.3231	13.66187	-8.74724
180	H	14.29753	14.34933	-9.68777	H	14.3882	14.31665	-9.67737	H	14.38801	14.31155	-9.62937
181	H	13.94965	14.38547	-7.96272	H	14.05341	14.3284	-7.94706	H	14.06159	14.30027	-7.89621
182	H	15.23232	13.31284	-8.58384	H	15.30077	13.23537	-8.598	H	15.31297	13.22477	-8.56825
183	H	12.20762	13.00932	-9.10313	H	12.26708	13.02809	-9.11785	H	12.27799	13.00979	-9.07876

Aux9R – Partial Ligand Exchange

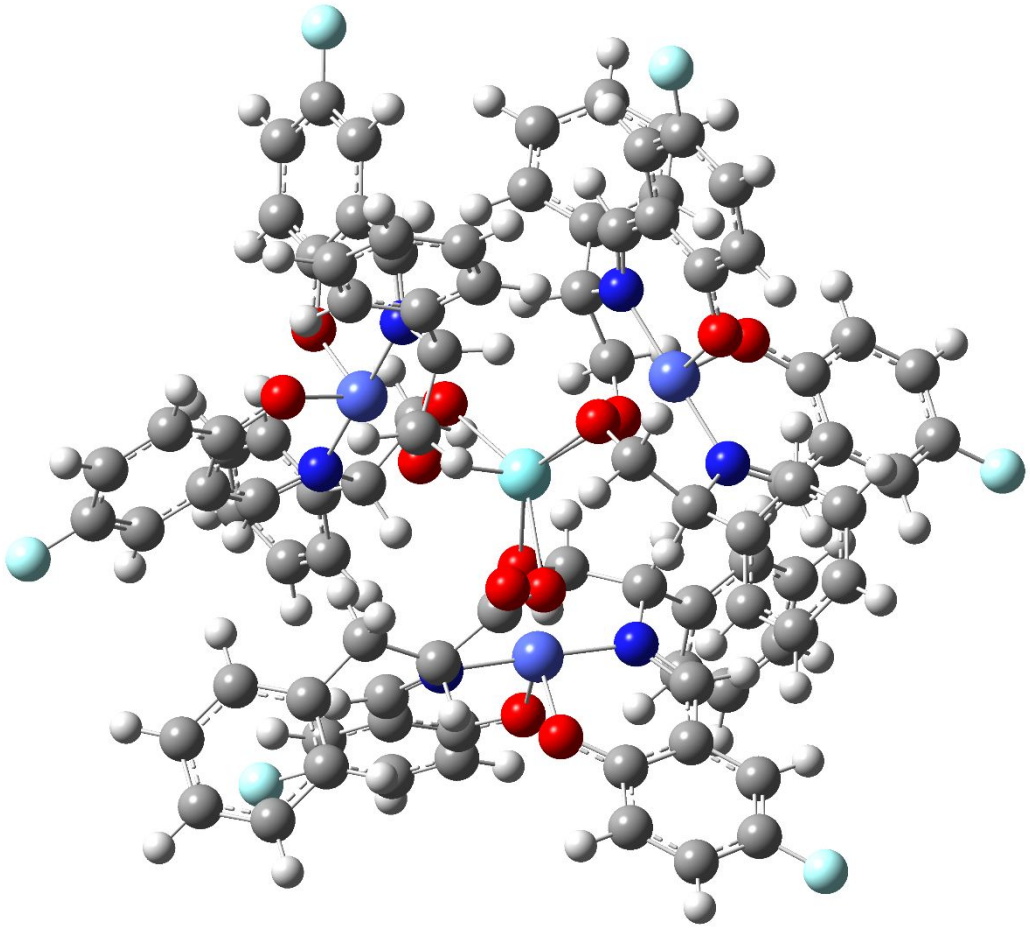

	B3LYP/SDD
**Electronic Energy (EE)**	-5892.480045
**EE + Zero-point Energy**	-5891.010585
**EE + Thermal Energy Correction**	-5890.908744
**EE + Thermal Enthalpy Correction**	-5890.907800
**EE + Thermal Free Energy Correction**	-5891.159971

	B3LYP/SDD			
Tag	Symbol	X	Y	Z
1	Y	10.35564	10.45638	-10.6028
2	Co	9.513685	8.488466	-13.1547
3	Co	13.18177	9.799481	-8.82716
4	Co	8.457962	13.06432	-9.7766
5	F	11.19292	1.762061	-13.3275
6	F	12.60587	11.75429	-2.25453
7	F	1.777565	13.32199	-11.6189
8	F	6.024955	10.99621	-18.5955
9	F	18.67078	6.336486	-11.2755
10	F	10.27514	18.67449	-6.1213
11	O	8.740245	6.757733	-13.2924

12	O	13.47088	9.132412	-7.08876
13	O	6.712353	13.23432	-9.04797
14	O	10.38267	10.21313	-12.8592
15	O	12.72207	10.61291	-10.5595
16	O	10.20471	12.72307	-10.6021
17	O	9.610432	8.653214	-15.0424
18	O	15.03071	9.989208	-9.19322
19	O	8.648149	14.94732	-9.91989
20	O	9.384641	8.481298	-11.1964
21	O	11.2464	9.601706	-8.6731
22	O	8.42427	11.11554	-9.61523
23	N	11.33772	7.851908	-13.2818
24	N	13.30101	11.64784	-8.2954
25	N	7.866885	13.14673	-11.6196
26	N	7.689996	9.127847	-13.0421
27	N	13.10552	7.947091	-9.3889
28	N	9.023726	12.99247	-7.92255
29	C	9.388097	5.590791	-13.3015
30	C	13.23719	9.822192	-5.96453
31	C	5.560648	13.25339	-9.72422
32	C	8.604946	4.3938	-13.315
33	C	13.17491	9.087351	-4.74036
34	C	4.347227	13.30901	-8.96978
35	H	7.524562	4.498384	-13.3276
36	H	13.30962	8.011495	-4.79026
37	H	4.427942	13.3448	-7.88778
38	C	9.196968	3.131692	-13.3209
39	C	12.96504	9.722533	-3.51646
40	C	3.099363	13.32886	-9.59161
41	H	8.598511	2.225385	-13.3323
42	H	12.92199	9.160971	-2.58782
43	H	2.179862	13.37371	-9.01526
44	C	10.6033	3.040213	-13.3207
45	C	12.81783	11.12346	-3.49324
46	C	3.040703	13.29958	-10.9992
47	C	11.41232	4.161003	-13.3206
48	C	12.88289	11.88865	-4.64294
49	C	4.17995	13.25681	-11.7811
50	H	12.49368	4.049988	-13.338
51	H	12.7905	12.96998	-4.5819
52	H	4.094275	13.25224	-12.8649
53	C	10.81934	5.457021	-13.3035
54	C	13.08114	11.2505	-5.90109
55	C	5.460993	13.22611	-11.1574
56	C	11.70059	6.602341	-13.3468
57	C	13.23744	12.08802	-7.07049
58	C	6.627197	13.22206	-12.0124

59	H	12.76329	6.380445	-13.4779
60	H	13.35574	13.15674	-6.88775
61	H	6.431918	13.32171	-13.0835
62	C	12.37004	8.946087	-13.4265
63	C	13.7527	12.51103	-9.42276
64	C	8.98603	13.22955	-12.6333
65	H	12.85955	9.059473	-12.4506
66	H	9.098801	12.227	-13.0677
67	C	11.58042	10.24749	-13.7003
68	C	13.29813	11.83702	-10.7283
69	C	10.26667	13.53044	-11.82
70	H	11.28198	10.30002	-14.7494
71	H	10.31046	14.58719	-11.548
72	H	12.1945	11.11406	-13.4369
73	H	11.16065	13.26549	-12.394
74	C	13.0587	8.320195	-15.8029
75	C	8.473078	15.5898	-13.4539
76	H	12.00482	8.192226	-16.0403
77	H	8.383788	15.89812	-12.4145
78	C	14.04205	8.120181	-16.7857
79	C	8.297658	16.52017	-14.4914
80	H	13.74909	7.84203	-17.7955
81	H	8.082471	17.55978	-14.2556
82	C	15.40724	8.278093	-16.4681
83	C	8.398472	16.11111	-15.8376
84	H	16.16708	8.122835	-17.2303
85	H	8.262226	16.83282	-16.6394
86	C	15.78123	8.634404	-15.1591
87	C	8.670766	14.7637	-16.1382
88	H	16.83121	8.75569	-14.9048
89	H	8.743104	14.43863	-17.173
90	C	14.79541	8.830483	-14.1729
91	C	8.842922	13.83056	-15.0976
92	H	15.08988	9.110465	-13.1632
93	H	9.050088	12.78868	-15.3356
94	C	13.42919	8.676946	-14.4852
95	C	8.750055	14.23455	-13.7498
96	C	8.708902	9.226581	-15.8431
97	C	15.85771	9.065425	-9.68902
98	C	9.043274	15.79158	-8.96526
99	C	9.034856	9.353232	-17.2299
100	C	17.22383	9.434448	-9.88733
101	C	9.113046	17.18263	-9.2919
102	H	9.996418	8.972078	-17.559
103	H	17.51887	10.44145	-9.60958
104	H	8.832398	17.47876	-10.2977
105	C	8.15348	9.938417	-18.1377

106	C	18.15149	8.537065	-10.4133
107	C	9.520922	18.13397	-8.35877
108	H	8.40453	10.03271	-19.1902
109	H	19.18961	8.818632	-10.5635
110	H	9.574548	19.18875	-8.61167
111	C	6.909386	10.40892	-17.6716
112	C	17.72596	7.235358	-10.7485
113	C	9.864209	17.70841	-7.05956
114	C	6.534586	10.30442	-16.3454
115	C	16.42024	6.819885	-10.57
116	C	9.809081	16.37988	-6.68309
117	H	5.559411	10.66439	-16.0269
118	H	16.13294	5.804043	-10.8294
119	H	10.06597	16.09174	-5.66661
120	C	7.430985	9.717192	-15.4055
121	C	15.45986	7.731163	-10.04
122	C	9.406994	15.39556	-7.63296
123	C	6.984681	9.585981	-14.0375
124	C	14.11321	7.250688	-9.83772
125	C	9.344344	14.02164	-7.19089
126	H	5.947669	9.874468	-13.8447
127	H	13.94738	6.193494	-10.0607
128	H	9.56262	13.8537	-6.13312
129	C	7.058315	8.980746	-11.6794
130	C	11.76857	7.282493	-9.16851
131	C	8.920671	11.62427	-7.29574
132	H	7.063033	9.973442	-11.2114
133	H	11.24862	7.264157	-10.1357
134	H	9.926357	11.18387	-7.3167
135	C	8.022022	8.084572	-10.865
136	C	10.97827	8.234055	-8.2406
137	C	8.029226	10.7975	-8.25141
138	H	7.876985	7.032508	-11.1203
139	H	11.2913	8.109319	-7.20129
140	H	6.976192	11.05215	-8.10897
141	H	7.840371	8.224981	-9.79241
142	H	9.905416	8.020826	-8.32105
143	H	8.170506	9.726677	-8.05656
144	C	5.636811	8.43913	-11.6997
145	C	11.84654	5.867842	-8.61421
146	C	8.390835	11.61159	-5.86948
147	C	4.606397	9.127109	-11.0262
148	C	11.15435	4.817989	-9.25222
149	C	9.114223	10.96121	-4.84907
150	H	4.82359	10.06808	-10.5238
151	H	10.59678	5.015027	-10.1658
152	H	10.07354	10.50135	-5.07731

153	C	3.297027	8.60904	-11.0016
154	C	11.18091	3.515172	-8.71805
155	C	8.607484	10.90421	-3.53637
156	H	2.510963	9.151882	-10.4828
157	H	10.64675	2.714083	-9.22246
158	H	9.179247	10.40588	-2.7578
159	C	3.006533	7.39807	-11.6567
160	C	11.90559	3.250963	-7.54119
161	C	7.369724	11.50037	-3.23295
162	H	1.995289	6.998693	-11.6431
163	H	11.931	2.244845	-7.12984
164	H	6.978455	11.46118	-2.21926
165	C	4.031528	6.708937	-12.3382
166	C	12.6055	4.295659	-6.90188
167	C	6.642494	12.15628	-4.24827
168	H	3.808803	5.776595	-12.8519
169	H	13.17271	4.093702	-5.99631
170	H	5.688181	12.62328	-4.01648
171	C	5.338082	7.22386	-12.3595
172	C	12.57582	5.595623	-7.43277
173	C	7.147887	12.21155	-5.55742
174	H	6.129269	6.704142	-12.8956
175	H	13.1277	6.399718	-6.9502
176	H	6.596218	12.73001	-6.33893
177	O	13.42896	12.3738	-11.8426
178	H	14.84785	12.43587	-9.43406
179	C	13.33114	14.0032	-9.39353
180	H	13.43268	14.35209	-10.43
181	H	12.26505	14.07131	-9.14645
182	C	14.15694	14.91392	-8.48849
183	C	15.57005	14.89859	-8.51445
184	C	13.51119	15.84963	-7.6507
185	C	16.31493	15.78325	-7.71405
186	H	16.09731	14.20727	-9.1696
187	C	14.25255	16.74103	-6.85124
188	H	12.42448	15.89468	-7.63408
189	C	15.65957	16.70763	-6.87628
190	H	17.40162	15.7569	-7.74812
191	H	13.73318	17.45792	-6.21973
192	H	16.23591	17.39302	-6.25986

Aux9S – Partial Ligand Exchange

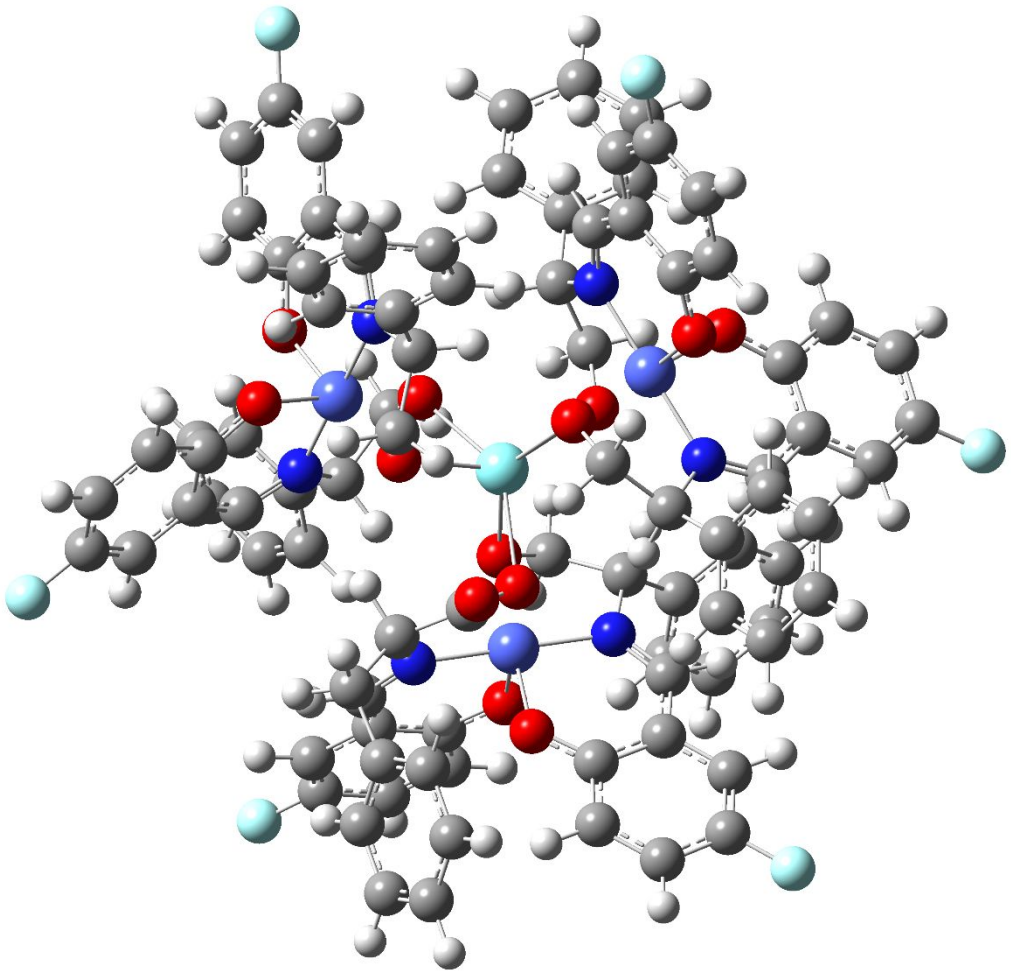

	B3LYP/SDD
**Electronic Energy (EE)**	-5892.474930
**EE + Zero-point Energy**	-5891.005707
**EE + Thermal Energy Correction**	-5890.903861
**EE + Thermal Enthalpy Correction**	-5890.902917
**EE + Thermal Free Energy Correction**	-5891.155370

	B3LYP/SDD			
Tag	Symbol	X	Y	Z
1	Y	10.41546	10.40777	-10.4792
2	Co	9.652694	8.563225	-13.1347
3	Co	13.12645	9.689028	-8.57927
4	Co	8.64166	13.09556	-9.65148
5	F	11.11856	1.79707	-13.5093
6	F	13.37205	11.25059	-1.8429
7	F	1.971505	13.78824	-11.4101
8	F	6.518091	11.30898	-18.6746
9	F	18.7264	6.424734	-11.0072
10	F	11.27024	18.41973	-6.07109
11	O	8.82951	6.867842	-13.3714

12	O	13.35107	8.895287	-6.88349
13	O	6.918425	13.35364	-8.89811
14	O	10.55733	10.24702	-12.7359
15	O	12.77417	10.56736	-10.301
16	O	10.35956	12.68373	-10.4894
17	O	9.857817	8.787537	-15.0067
18	O	14.97709	9.909567	-8.83069
19	O	8.94146	14.96551	-9.76066
20	O	9.419679	8.493576	-11.1875
21	O	11.18078	9.480087	-8.53812
22	O	8.496615	11.14735	-9.523
23	N	11.46037	7.870701	-13.1872
24	N	13.18978	11.51399	-7.92371
25	N	8.037916	13.24099	-11.4845
26	N	7.846958	9.263248	-13.0931
27	N	13.07648	7.876981	-9.2517
28	N	9.241928	12.94812	-7.8155
29	C	9.439654	5.680812	-13.4018
30	C	13.35484	9.524455	-5.70495
31	C	5.763347	13.45775	-9.56052
32	C	8.61984	4.513446	-13.5062
33	C	13.43825	8.719129	-4.52661
34	C	4.563766	13.57757	-8.79151
35	H	7.545391	4.656058	-13.5661
36	H	13.50666	7.643606	-4.65546
37	H	4.657461	13.59048	-7.7101
38	C	9.170205	3.233118	-13.5393
39	C	13.44283	9.285132	-3.25303
40	C	3.313156	13.68416	-9.39838
41	H	8.544113	2.34925	-13.6195
42	H	13.50864	8.670315	-2.3602
43	H	2.404364	13.77643	-8.81083
44	C	10.57112	3.093321	-13.4749
45	C	13.36759	10.68747	-3.13145
46	C	3.237589	13.6794	-10.8056
47	C	11.41497	4.184427	-13.3859
48	C	13.2962	11.51718	-4.23408
49	C	4.363352	13.5782	-11.6013
50	H	12.49162	4.036974	-13.3544
51	H	13.25233	12.59503	-4.09817
52	H	4.265922	13.5942	-12.684
53	C	10.86417	5.498401	-13.3406
54	C	13.2842	10.95041	-5.54245
55	C	5.646898	13.45949	-10.9929
56	C	11.78397	6.612592	-13.2884
57	C	13.25413	11.85499	-6.66811
58	C	6.800716	13.39669	-11.8618

59	H	12.84404	6.358584	-13.3735
60	H	13.30992	12.91774	-6.41955
61	H	6.59972	13.522	-12.9292
62	C	12.53487	8.933124	-13.2274
63	C	13.24725	12.54883	-8.99482
64	C	9.150352	13.26539	-12.5078
65	H	12.9678	8.99582	-12.2205
66	H	12.25172	13.02541	-9.04215
67	H	9.205533	12.26103	-12.9487
68	C	11.80545	10.26964	-13.499
69	C	13.32075	11.82272	-10.3472
70	C	10.45333	13.48956	-11.7063
71	H	11.57641	10.37242	-14.5618
72	H	10.56156	14.54148	-11.4336
73	H	12.43134	11.10432	-13.1661
74	H	11.32443	13.17551	-12.2906
75	C	13.34901	8.368587	-15.5793
76	C	8.755675	15.65466	-13.3093
77	H	12.30848	8.280645	-15.8844
78	H	8.682422	15.95939	-12.2675
79	C	14.38436	8.173263	-16.5081
80	C	8.626622	16.60109	-14.339
81	H	14.14583	7.938927	-17.543
82	H	8.463786	17.64815	-14.0944
83	C	15.73149	8.279167	-16.1035
84	C	8.706186	16.19842	-15.6886
85	H	16.53182	8.126967	-16.8237
86	H	8.605698	16.93251	-16.4844
87	C	16.03476	8.579518	-14.7626
88	C	8.910951	14.84165	-16.0004
89	H	17.0701	8.660148	-14.4412
90	H	8.966604	14.52163	-17.0378
91	C	14.9969	8.771448	-13.8305
92	C	9.03673	13.8925	-14.9677
93	H	15.23753	9.008925	-12.7962
94	H	9.192505	12.84393	-15.2147
95	C	13.64862	8.668325	-14.2297
96	C	8.963826	14.28959	-13.6166
97	C	9.012158	9.402356	-15.8365
98	C	15.82735	9.025287	-9.35871
99	C	9.506771	15.74176	-8.83356
100	C	9.412142	9.554965	-17.2013
101	C	17.19868	9.412983	-9.46783
102	C	9.711015	17.12069	-9.15398
103	H	10.38	9.157717	-17.4904
104	H	17.47143	10.40005	-9.11297
105	H	9.38955	17.46343	-10.1325

106	C	8.593327	10.18482	-18.137
107	C	18.15202	8.55671	-10.0132
108	C	10.29558	18.00357	-8.24803
109	H	8.900506	10.29874	-19.1725
110	H	19.19388	8.85217	-10.0964
111	H	10.45024	19.04959	-8.49587
112	C	7.338596	10.67564	-17.7227
113	C	17.75123	7.279553	-10.4597
114	C	10.68565	17.52097	-6.98217
115	C	6.893498	10.54807	-16.4205
116	C	16.44338	6.846951	-10.37
117	C	10.50443	16.20222	-6.6116
118	H	5.912927	10.9263	-16.1423
119	H	16.17405	5.85096	-10.7123
120	H	10.79904	15.87237	-5.61822
121	C	7.725689	9.914698	-15.4516
122	C	15.45405	7.71664	-9.81958
123	C	9.918313	15.28579	-7.53393
124	C	7.206867	9.765157	-14.1115
125	C	14.10719	7.215658	-9.70352
126	C	9.713752	13.92546	-7.09358
127	H	6.170654	10.08172	-13.9641
128	H	13.95868	6.173274	-9.9975
129	H	9.955734	13.72245	-6.04664
130	C	7.145184	9.10726	-11.7658
131	C	11.73944	7.190976	-9.1131
132	C	9.022167	11.59027	-7.19448
133	H	7.172326	10.0852	-11.2671
134	H	11.25634	7.207548	-10.0994
135	H	9.988918	11.07006	-7.20696
136	C	8.027425	8.143899	-10.9359
137	C	10.90365	8.094144	-8.17591
138	C	8.080556	10.83384	-8.16214
139	H	7.852613	7.107805	-11.2342
140	H	11.1805	7.92751	-7.13224
141	H	7.044985	11.15005	-8.01727
142	H	7.798579	8.258484	-9.86894
143	H	9.836209	7.878495	-8.30813
144	H	8.157341	9.753387	-7.98484
145	C	5.702751	8.634978	-11.8658
146	C	11.81153	5.754144	-8.61775
147	C	8.485706	11.62357	-5.77155
148	C	4.675133	9.353319	-11.2203
149	C	11.14528	4.728899	-9.32019
150	C	9.14623	10.91348	-4.74773
151	H	4.912374	10.26906	-10.6816
152	H	10.61288	4.96194	-10.2403

153	H	10.06372	10.37201	-4.9716
154	C	3.343526	8.897265	-11.2692
155	C	11.1646	3.404522	-8.8416
156	C	8.630057	10.90043	-3.43744
157	H	2.560158	9.462551	-10.7707
158	H	10.65072	2.622917	-9.39532
159	H	9.150843	10.35188	-2.65667
160	C	3.028436	7.718514	-11.9702
161	C	11.85594	3.094182	-7.65595
162	C	7.448454	11.60401	-3.13936
163	H	2.000453	7.366833	-12.0129
164	H	11.87596	2.071524	-7.28725
165	H	7.050187	11.59871	-2.12766
166	C	4.051097	6.999227	-12.6235
167	C	12.5291	4.114561	-6.95191
168	C	6.786853	12.32208	-4.15753
169	H	3.809928	6.091529	-13.1717
170	H	13.06994	3.877084	-6.03884
171	H	5.877023	12.87218	-3.92915
172	C	5.379522	7.452308	-12.5716
173	C	12.50665	5.435911	-7.42747
174	C	7.299933	12.3317	-5.46494
175	H	6.169615	6.908791	-13.0854
176	H	13.03585	6.223293	-6.89493
177	H	6.800769	12.8956	-6.25013
178	C	14.26862	13.71147	-8.83572
179	H	14.07134	14.3658	-9.69361
180	H	13.98943	14.28875	-7.94424
181	C	15.75488	13.38855	-8.7891
182	C	16.50556	13.30908	-9.98441
183	C	16.43349	13.25653	-7.55771
184	C	17.89743	13.10918	-9.94683
185	H	15.99644	13.40938	-10.9392
186	C	17.82459	13.04855	-7.51551
187	H	15.8813	13.33169	-6.62262
188	C	18.56415	12.98032	-8.71196
189	H	18.45947	13.06284	-10.8768
190	H	18.32829	12.95329	-6.55647
191	H	19.64205	12.83869	-8.68186
192	O	13.68145	12.36742	-11.4018

6x Partial Ligand Exchange

Aux1R – 6x Partial Ligand Exchange

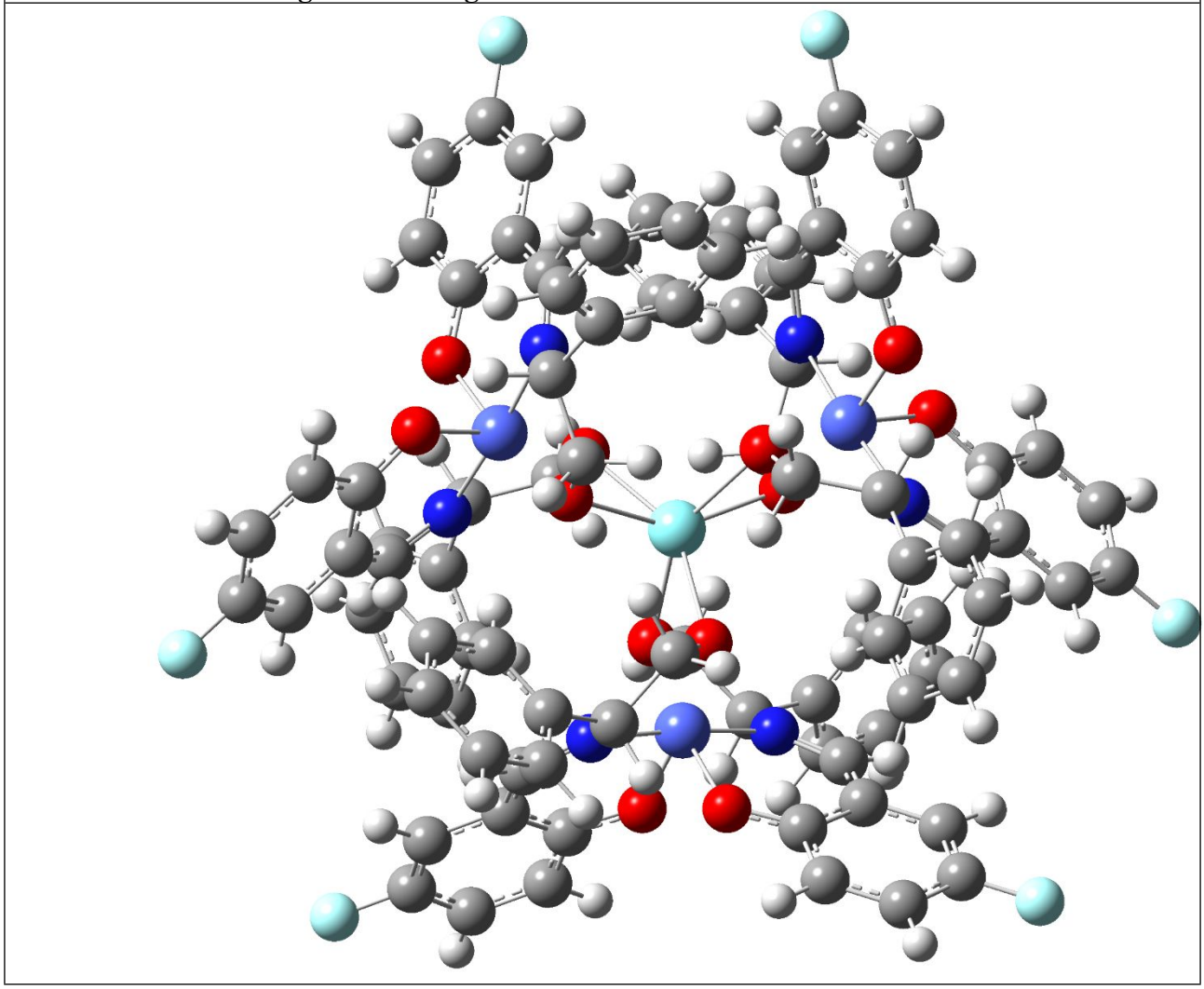

	B3LYP/SDD
**Electronic Energy (EE)**	-5779.06918
**EE + Zero-point Energy**	-5777.60847
**EE + Thermal Energy Correction**	-5777.50835
**EE + Thermal Enthalpy Correction**	-5777.5074
**EE + Thermal Free Energy Correction**	-5777.74938

	B3LYP/SDD			
Tag	Symbol	X	Y	Z
1	Y	10.45043	10.46426	-10.4812
2	Co	9.643032	8.601912	-13.2292
3	Co	13.15192	9.606538	-8.57385
4	Co	8.556581	13.1801	-9.6399
5	F	12.27264	2.181434	-13.399
6	F	13.24234	12.17152	-2.12536
7	F	2.160873	13.35838	-12.3292
8	F	5.586347	11.71554	-17.9223
9	F	17.84703	5.516347	-11.6398
10	F	11.59347	17.81394	-5.45933

11	O	9.144757	6.782127	-13.4938
12	O	13.37755	9.087549	-6.7545
13	O	6.729917	13.42022	-9.15459
14	O	10.38369	10.37937	-12.7954
15	O	12.76097	10.3693	-10.3518
16	O	10.34479	12.77561	-10.3707
17	O	9.517793	8.97772	-15.0928
18	O	15.02018	9.460598	-8.91765
19	O	8.916648	15.04363	-9.47406
20	O	9.606089	8.453272	-11.2615
21	O	11.18146	9.594589	-8.46162
22	O	8.423392	11.21102	-9.64414
23	N	11.50127	8.215946	-13.603
24	N	13.5425	11.45587	-8.16285
25	N	8.186088	13.58811	-11.4939
26	N	7.750014	8.907449	-12.9739
27	N	12.8773	7.719753	-8.90128
28	N	8.844856	12.88907	-7.74934
29	C	9.947395	5.717481	-13.4568
30	C	13.33218	9.88015	-5.68273
31	C	5.673482	13.39232	-9.9683
32	C	9.340254	4.421984	-13.4862
33	C	13.3312	9.260106	-4.39302
34	C	4.371942	13.40008	-9.37354
35	H	8.257026	4.37534	-13.545
36	H	13.37529	8.175809	-4.3561
37	H	4.314295	13.43597	-8.28985
38	C	10.10578	3.256258	-13.4691
39	C	13.30349	10.01445	-3.22023
40	C	3.21391	13.3908	-10.1508
41	H	9.642837	2.274145	-13.5015
42	H	13.31282	9.54163	-2.24234
43	H	2.227113	13.40682	-9.69704
44	C	11.50989	3.363699	-13.4202
45	C	13.27449	11.42007	-3.31459
46	C	3.335444	13.3719	-11.5545
47	C	12.15368	4.587677	-13.4181
48	C	13.30199	12.07568	-4.53197
49	C	4.565647	13.39146	-12.186
50	H	13.24008	4.630868	-13.425
51	H	13.32337	13.16226	-4.56423
52	H	4.619396	13.42086	-13.2715
53	C	11.38322	5.78505	-13.4377
54	C	13.33277	11.31665	-5.7364
55	C	5.755268	13.40342	-11.4034
56	C	12.06897	7.043675	-13.6285
57	C	13.55437	12.01186	-6.98472

58	C	7.019203	13.61674	-12.0726
59	H	13.12716	6.983723	-13.8931
60	H	13.83108	13.06598	-6.90975
61	H	6.967711	13.90199	-13.1258
62	C	12.13599	9.400763	-14.2698
63	C	14.23821	12.09402	-9.32914
64	H	11.69521	9.37859	-15.2714
65	C	11.59559	10.67113	-13.5606
66	C	13.54638	11.57422	-10.6176
67	C	10.64281	13.56833	-11.5631
68	H	11.3876	11.43869	-14.3154
69	H	14.31294	11.36398	-11.3728
70	H	11.40031	14.32621	-11.3307
71	H	12.36211	11.05659	-12.8801
72	H	12.88278	12.35266	-11.0085
73	H	11.04407	12.90759	-12.3387
74	C	14.21434	9.470843	-15.7048
75	C	9.448534	15.74868	-14.1559
76	H	13.56017	9.52024	-16.5737
77	H	9.482854	16.60626	-13.486
78	C	15.61068	9.498586	-15.8805
79	C	9.488288	15.94944	-15.5486
80	H	16.03076	9.570829	-16.8807
81	H	9.554733	16.95757	-15.9502
82	C	16.45824	9.426575	-14.7597
83	C	9.435731	14.84314	-16.4164
84	H	17.53783	9.44126	-14.8882
85	H	9.459739	14.99091	-17.4934
86	C	15.90148	9.326788	-13.4689
87	C	9.343303	13.54173	-15.8835
88	H	16.55124	9.262593	-12.6017
89	H	9.294259	12.68563	-16.5491
90	C	14.50698	9.307264	-13.2948
91	C	9.311778	13.34273	-14.4926
92	H	14.08494	9.227422	-12.2951
93	H	9.237828	12.335	-14.089
94	C	13.64848	9.381011	-14.4151
95	C	9.36592	14.44839	-13.6138
96	C	8.54349	9.644617	-15.7133
97	C	15.63969	8.484025	-9.58214
98	C	9.569276	15.64925	-8.48093
99	C	8.738762	9.984969	-17.0894
100	C	17.02403	8.66436	-9.89656
101	C	9.901831	17.0313	-8.64557
102	H	9.668199	9.674673	-17.5573
103	H	17.49811	9.584988	-9.56993
104	H	9.597852	17.51517	-9.56886

105	C	7.764056	10.66568	-17.8187
106	C	17.75267	7.686199	-10.5729
107	C	10.56715	17.74592	-7.64964
108	H	7.910588	10.91405	-18.8659
109	H	18.80593	7.821322	-10.8016
110	H	10.80951	18.79757	-7.77298
111	C	6.561325	11.02531	-17.1787
112	C	17.10385	6.494924	-10.9542
113	C	10.91879	17.08488	-6.45596
114	C	6.304894	10.69948	-15.8593
115	C	15.77579	6.252763	-10.6538
116	C	10.59988	15.75847	-6.22897
117	H	5.347304	10.95494	-15.4122
118	H	15.32114	5.303128	-10.9252
119	H	10.84865	15.29433	-5.27773
120	C	7.291083	10.00227	-15.105
121	C	15.02184	7.242674	-9.96144
122	C	9.918468	15.0188	-7.23709
123	C	6.933632	9.503628	-13.7957
124	C	13.69937	6.897833	-9.48938
125	C	9.426121	13.69904	-6.9107
126	H	5.877267	9.5674	-13.5247
127	H	13.41624	5.845584	-9.56695
128	H	9.481053	13.40773	-5.85926
129	C	7.237705	8.007005	-11.8883
130	C	11.7691	7.214427	-8.02491
131	C	7.947818	11.78661	-7.26826
132	H	7.244186	7.023255	-12.368
133	H	12.2306	7.207641	-7.03254
134	H	6.960486	12.25881	-7.27502
135	C	8.31318	7.977751	-10.77
136	C	10.66415	8.303804	-8.00805
137	C	7.937888	10.69017	-8.36655
138	H	8.408046	6.951955	-10.3945
139	H	10.26916	8.394223	-6.98918
140	H	6.915865	10.30948	-8.47994
141	H	7.989382	8.618348	-9.94311
142	H	9.846463	7.996328	-8.6682
143	H	8.581026	9.861413	-8.05278
144	C	5.833143	8.277162	-11.3752
145	C	11.24309	5.818557	-8.31467
146	C	8.207738	11.24707	-5.87167
147	C	4.862299	7.256966	-11.4652
148	C	11.29875	4.840694	-7.29871
149	C	9.441728	10.6574	-5.51489
150	H	5.125606	6.307406	-11.9281
151	H	11.7457	5.092114	-6.33832

152	H	10.24981	10.61304	-6.24213
153	C	3.561577	7.451404	-10.9634
154	C	10.78325	3.54819	-7.51114
155	C	9.63076	10.13791	-4.22272
156	H	2.825975	6.654292	-11.038
157	H	10.83138	2.807098	-6.71708
158	H	10.58486	9.693156	-3.95765
159	C	3.216735	8.68053	-10.3716
160	C	10.21212	3.218599	-8.75413
161	C	8.59177	10.19322	-3.27215
162	H	2.212229	8.840028	-9.98722
163	H	9.817255	2.22046	-8.92744
164	H	8.743843	9.789566	-2.27406
165	C	4.177381	9.708194	-10.2867
166	C	10.16155	4.186174	-9.77753
167	C	7.36088	10.78039	-3.61853
168	H	3.913751	10.66097	-9.83824
169	H	9.729327	3.934281	-10.7409
170	H	6.554987	10.8322	-2.89057
171	C	5.477859	9.509485	-10.7812
172	C	10.66972	5.478523	-9.56099
173	C	7.176045	11.30723	-4.9107
174	H	6.212916	10.30886	-10.7141
175	H	10.62924	6.218904	-10.3572
176	H	6.225051	11.76666	-5.17503
177	H	15.23414	11.64159	-9.29298
178	C	14.39989	13.60452	-9.29161
179	C	15.6972	14.15688	-9.34884
180	C	13.28761	14.47459	-9.23249
181	C	15.88865	15.55143	-9.35862
182	H	16.55974	13.49364	-9.38685
183	C	13.47719	15.86718	-9.2341
184	H	12.28195	14.06291	-9.1778
185	C	14.7758	16.41057	-9.30125
186	H	16.89462	15.96112	-9.40574
187	H	12.61596	16.52589	-9.18147
188	H	14.91633	17.48876	-9.30216
189	C	9.371765	14.27627	-12.104
190	H	9.336387	15.26953	-11.6457

**Aux1S – 6x Partial Ligand Exchange**
See Co_3YL_6

Aux6R – 6x Partial Ligand Exchange

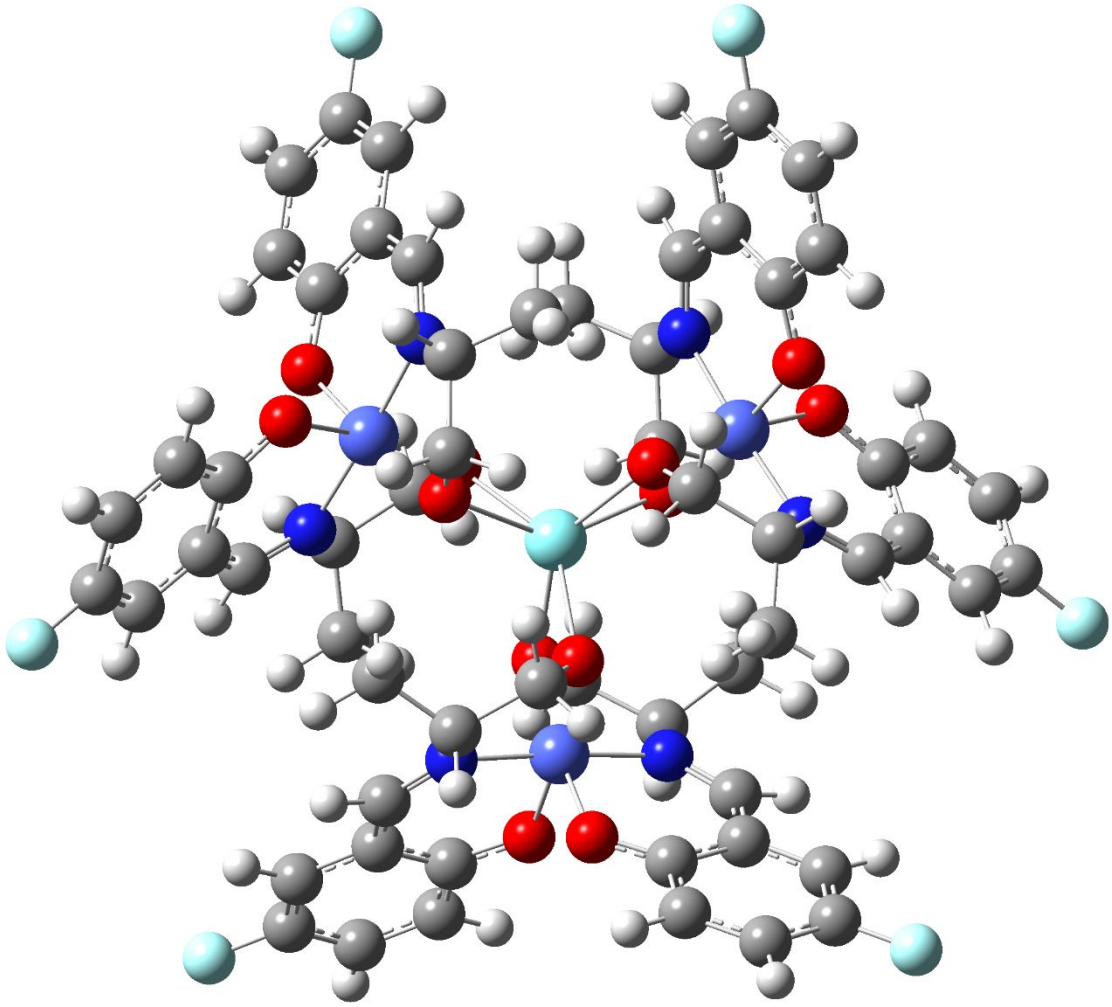

	B3LYP/SDD
**Electronic Energy (EE)**	-4628.85535
**EE + Zero-point Energy**	-4627.71619
**EE + Thermal Energy Correction**	-4627.63563
**EE + Thermal Enthalpy Correction**	-4627.63468
**EE + Thermal Free Energy Correction**	-4627.83376

	B3LYP/SDD			
Tag	Symbol	X	Y	Z
1	Y	10.50471	10.49779	-10.4961
2	Co	9.713942	8.61439	-13.185
3	Co	13.18401	9.697044	-8.60328
4	Co	8.622942	13.1883	-9.70651
5	F	11.59977	1.959306	-13.022
6	F	13.01415	11.59741	-1.95242
7	F	1.976672	13.04911	-11.6255
8	F	6.351245	11.75556	-18.3526
9	F	18.34192	6.301009	-11.7242
10	F	11.75962	18.33799	-6.31241
11	O	9.000694	6.864368	-13.39

12	O	13.38024	8.986302	-6.85122
13	O	6.869887	13.3948	-9.00118
14	O	10.57294	10.31751	-12.7804
15	O	12.78879	10.55463	-10.3093
16	O	10.32942	12.78262	-10.5584
17	O	9.81665	8.909209	-15.0602
18	O	15.0605	9.789172	-8.89304
19	O	8.92234	15.06314	-9.80181
20	O	9.544262	8.554555	-11.2433
21	O	11.24124	9.537771	-8.54855
22	O	8.558147	11.24617	-9.54323
23	N	11.54706	8.040429	-13.3916
24	N	13.3988	11.53023	-8.03247
25	N	8.058449	13.40214	-11.5418
26	N	7.853967	9.124135	-13.0672
27	N	13.05766	7.836695	-9.10954
28	N	9.123217	13.06346	-7.84444
29	C	9.679916	5.720807	-13.2911
30	C	13.28216	9.668395	-5.70931
31	C	5.729416	13.30136	-9.68633
32	C	8.931981	4.503091	-13.2234
33	C	13.20715	8.923342	-4.49025
34	C	4.507877	13.23416	-8.94461
35	H	7.849264	4.576759	-13.2574
36	H	13.23537	7.840305	-4.56163
37	H	4.576326	13.26413	-7.86143
38	C	9.561922	3.263024	-13.1304
39	C	13.11452	9.556326	-3.25171
40	C	3.27067	13.14674	-9.58094
41	H	8.992045	2.339866	-13.0791
42	H	13.05769	8.988633	-2.32753
43	H	2.344599	13.09582	-9.01577
44	C	10.97067	3.215473	-13.1133
45	C	13.1051	10.96524	-3.20707
46	C	3.229979	13.13478	-10.99
47	C	11.7463	4.357199	-13.1941
48	C	13.19307	11.73807	-4.35016
49	C	4.375701	13.21528	-11.7597
50	H	12.8307	4.27626	-13.2036
51	H	13.20837	12.82257	-4.2714
52	H	4.3001	13.22884	-12.8444
53	C	11.11421	5.631544	-13.2741
54	C	13.27284	11.10294	-5.62301
55	C	5.647143	13.28968	-11.1211
56	C	11.95032	6.801263	-13.4274
57	C	13.43358	11.9358	-6.79403
58	C	6.821356	13.44263	-11.9509

59	H	13.00589	6.601405	-13.6314
60	H	13.64284	12.99066	-6.5958
61	H	6.627218	13.65081	-13.0068
62	C	12.47284	9.137662	-13.8449
63	C	13.8596	12.45154	-9.13036
64	C	9.161318	13.85517	-12.461
65	C	11.7221	10.49089	-13.652
66	C	13.66696	11.69881	-10.4825
67	C	10.51029	13.65752	-11.7039
68	H	11.38472	10.87022	-14.6224
69	H	14.63677	11.35545	-10.858
70	H	10.88949	14.6262	-11.3614
71	H	12.39962	11.22975	-13.2029
72	H	13.22382	12.37704	-11.2243
73	H	11.25191	13.20935	-12.379
74	C	8.948326	9.603647	-15.7968
75	C	15.7943	8.915655	-9.58394
76	C	9.614327	15.79538	-8.92785
77	C	9.321766	9.930505	-17.1386
78	C	17.13894	9.281375	-9.90799
79	C	9.946124	17.13761	-9.2955
80	H	10.29415	9.593723	-17.4849
81	H	17.48946	10.25256	-9.57214
82	H	9.614857	17.48769	-10.2684
83	C	8.469839	10.64294	-17.9812
84	C	17.97882	8.423655	-10.6166
85	C	10.6564	17.97581	-8.43751
86	H	8.75551	10.8899	-18.9995
87	H	18.9993	8.70347	-10.8615
88	H	10.90712	18.99445	-8.71879
89	C	7.207327	11.03887	-17.495
90	C	17.48696	7.16298	-11.0114
91	C	11.04508	17.48472	-7.17463
92	C	6.784631	10.73658	-16.2137
93	C	16.20272	6.747639	-10.7117
94	C	10.73776	16.20287	-6.75742
95	H	5.792961	11.03822	-15.8848
96	H	15.86937	5.757135	-11.0123
97	H	11.03381	15.87016	-5.76533
98	C	7.652246	10.02228	-15.338
99	C	15.32972	7.621235	-10.0013
100	C	10.02563	15.33154	-7.63122
101	C	7.156677	9.662287	-14.0282
102	C	14.01638	7.133279	-9.64382
103	C	9.660164	14.02105	-7.14151
104	H	6.087936	9.829166	-13.8675
105	H	13.8505	6.065071	-9.80901

106	H	9.8214	13.85665	-6.07247
107	C	7.147843	8.551346	-11.8672
108	C	11.85256	7.138117	-8.5382
109	C	8.544037	11.86265	-7.14493
110	C	8.242074	8.01287	-10.8953
111	C	10.88482	8.238623	-8.00508
112	C	8.009162	10.89526	-8.2449
113	H	8.28057	6.919649	-10.9479
114	H	10.93434	8.279234	-6.91178
115	H	6.916258	10.95024	-8.28877
116	H	7.9995	8.306101	-9.86474
117	H	9.853903	8.000816	-8.30093
118	H	8.29909	9.863368	-8.00403
119	C	13.1601	13.82481	-9.11316
120	H	13.5788	14.45093	-9.9123
121	H	12.08655	13.71233	-9.28561
122	H	13.31739	14.36613	-8.17265
123	H	14.93722	12.59353	-8.97335
124	C	11.16871	6.182282	-9.53522
125	H	10.33032	5.680263	-9.03415
126	H	10.77998	6.735847	-10.3939
127	H	11.84171	5.393563	-9.892
128	H	12.21642	6.561762	-7.67709
129	C	6.191101	9.549082	-11.1856
130	H	5.683524	9.047199	-10.3511
131	H	6.74488	10.40535	-10.7919
132	H	5.406756	9.90959	-11.8617
133	H	6.571116	7.692676	-12.2362
134	C	9.535168	11.17596	-6.18501
135	H	9.028785	10.34094	-5.68272
136	H	10.3935	10.7821	-6.73548
137	H	9.892965	11.84884	-5.39665
138	H	7.683167	12.23191	-6.57166
139	C	13.84281	9.115618	-13.1391
140	H	14.47239	9.914864	-13.5524
141	H	13.72566	9.284951	-12.0656
142	H	14.3831	8.174588	-13.2969
143	H	12.61948	8.983393	-14.9223
144	C	9.144505	13.15233	-13.8326
145	H	9.947597	13.56534	-14.4574
146	H	9.311136	12.0782	-13.7169
147	H	8.206386	13.31307	-14.377
148	H	9.010191	14.93321	-12.6061

Aux6S – 6x Partial Ligand Exchange

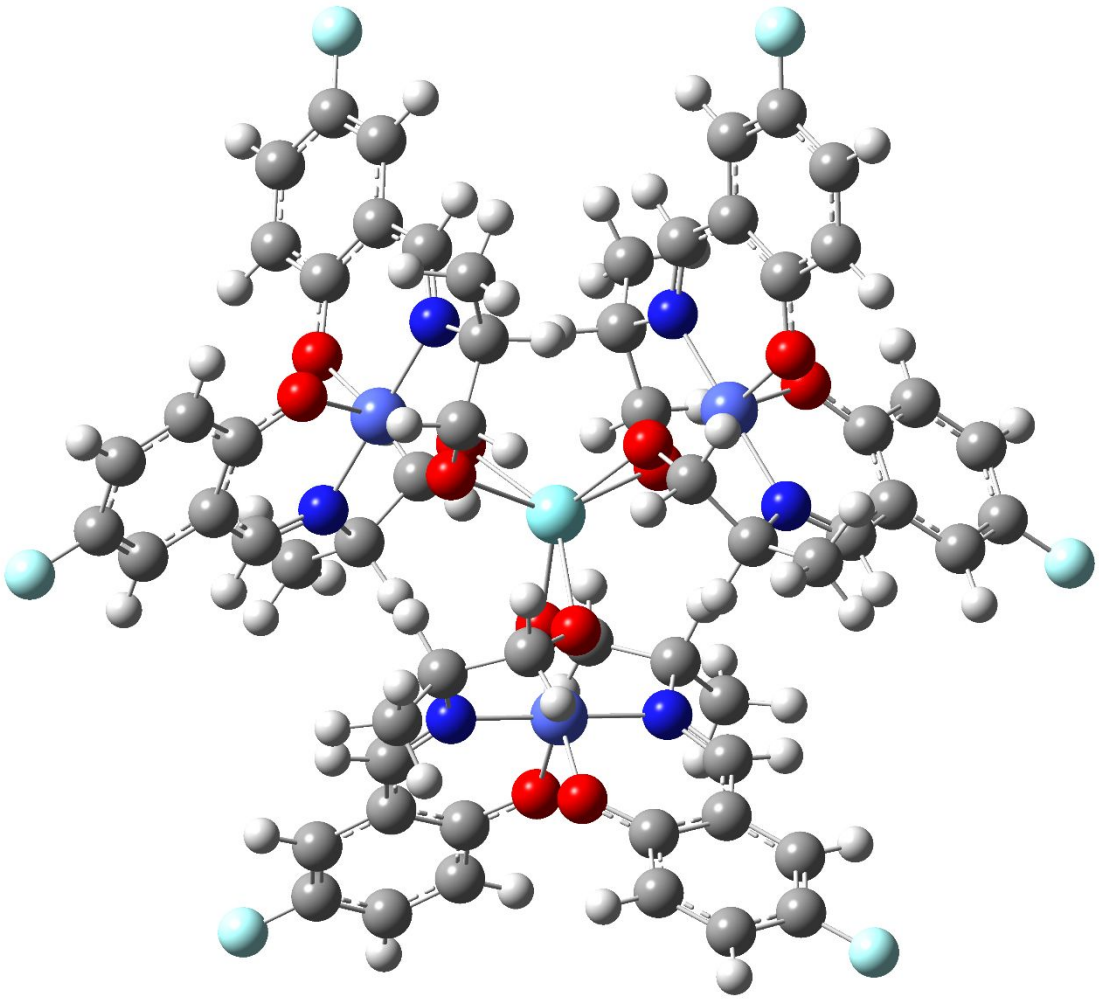

	B3LYP/SDD
**Electronic Energy (EE)**	-4628.89409
**EE + Zero-point Energy**	-4627.75575
**EE + Thermal Energy Correction**	-4627.67464
**EE + Thermal Enthalpy Correction**	-4627.67369
**EE + Thermal Free Energy Correction**	-4627.87829

	B3LYP/SDD			
Tag	Symbol	X	Y	Z
1	Y	10.50831	10.51092	-10.5093
2	Co	9.696629	8.657017	-13.1665
3	Co	13.16649	9.699937	-8.65648
4	Co	8.655111	13.16879	-9.69791
5	F	11.2042	1.898561	-13.3836
6	F	13.3991	11.20902	-1.89869
7	F	1.89636	13.40683	-11.2023
8	F	6.377921	11.08674	-18.7465
9	F	18.74361	6.381205	-11.0926
10	F	11.09505	18.74588	-6.38199
11	O	8.888056	6.956662	-13.3891

12	O	13.38995	8.891542	-6.95615
13	O	6.955356	13.39208	-8.88826
14	O	10.58402	10.35547	-12.7893
15	O	12.78847	10.58679	-10.355
16	O	10.3531	12.79096	-10.5858
17	O	9.888019	8.876368	-15.04
18	O	15.0399	9.890946	-8.87703
19	O	8.875355	15.04223	-9.88917
20	O	9.502254	8.587441	-11.2263
21	O	11.22632	9.505829	-8.58574
22	O	8.584836	11.2286	-9.50362
23	N	11.51361	7.982903	-13.2311
24	N	13.23199	11.51711	-7.98275
25	N	7.980066	13.23426	-11.5146
26	N	7.875537	9.326062	-13.1069
27	N	13.10619	7.878743	-9.32511
28	N	9.325038	13.10865	-7.8772
29	C	9.503975	5.774477	-13.3862
30	C	13.39069	9.507775	-5.77413
31	C	5.77293	13.39425	-9.5037
32	C	8.689919	4.600275	-13.4635
33	C	13.47004	8.693956	-4.59991
34	C	4.599329	13.47434	-8.68906
35	H	7.615528	4.739958	-13.5327
36	H	13.5379	7.619464	-4.73948
37	H	4.739674	13.54163	-7.61464
38	C	9.246715	3.322774	-13.4595
39	C	13.46971	9.251095	-3.32256
40	C	3.321603	13.47538	-9.24535
41	H	8.625959	2.433474	-13.5178
42	H	13.52962	8.630523	-2.43323
43	H	2.432751	13.53587	-8.62416
44	C	10.64858	3.192122	-13.3858
45	C	13.39754	10.65306	-3.1921
46	C	3.190149	13.40388	-10.6472
47	C	11.48671	4.28931	-13.3242
48	C	13.33396	11.49095	-4.28936
49	C	4.286786	13.33961	-11.4859
50	H	12.56422	4.146212	-13.2893
51	H	13.30037	12.56852	-4.14647
52	H	4.14315	13.30649	-12.5634
53	C	10.92958	5.601911	-13.318
54	C	13.32413	10.93347	-5.6018
55	C	5.599587	13.32838	-10.9293
56	C	11.84192	6.723491	-13.2978
57	C	13.30167	11.84557	-6.72353
58	C	6.720666	13.30506	-11.8422

59	H	12.90209	6.464531	-13.3682
60	H	13.37307	12.90574	-6.46488
61	H	6.461289	13.37672	-12.9021
62	C	12.56932	9.054328	-13.3081
63	C	13.3068	12.57258	-9.05454
64	C	9.051205	13.30814	-12.5709
65	H	13.03696	9.124618	-12.316
66	H	9.120847	12.31498	-13.0362
67	C	11.81707	10.37761	-13.5633
68	C	13.56201	11.82013	-10.3777
69	C	10.37476	13.56408	-11.8194
70	H	11.57692	10.48118	-14.626
71	H	14.62478	11.58034	-10.4815
72	H	10.47835	14.62698	-11.5801
73	H	12.43871	11.225	-13.2442
74	H	13.24246	12.44143	-11.2252
75	H	11.2219	13.24456	-12.4411
76	C	9.000472	9.40993	-15.8793
77	C	15.87846	9.003514	-9.41186
78	C	9.411226	15.88077	-9.00234
79	C	9.371481	9.523142	-17.2564
80	C	17.25549	9.374491	-9.52659
81	C	9.52583	17.25774	-9.37357
82	H	10.35107	9.153202	-17.543
83	H	17.54256	10.35399	-9.15677
84	H	9.155077	17.54479	-10.3527
85	C	8.511259	10.07778	-18.2022
86	C	18.20057	8.514353	-10.0825
87	C	10.08278	18.20281	-8.51408
88	H	8.795716	10.16228	-19.2469
89	H	19.24525	8.798785	-10.1682
90	H	10.16834	19.24745	-8.7987
91	C	7.242476	10.52935	-17.7852
92	C	17.78308	7.24568	-10.5339
93	C	10.53532	17.78537	-7.24581
94	C	6.823853	10.4347	-16.4715
95	C	16.46946	6.827081	-10.4378
96	C	10.43939	16.47181	-6.82698
97	H	5.830249	10.77862	-16.1939
98	H	16.19137	5.833538	-10.7815
99	H	10.78407	16.19375	-5.83375
100	C	7.697525	9.87708	-15.4922
101	C	15.49078	7.70068	-9.87884
102	C	9.879425	15.49314	-7.69993
103	C	7.207083	9.763833	-14.1366
104	C	14.13532	7.210251	-9.76403
105	C	9.764761	14.13777	-7.20922

106	H	6.164999	10.06153	-13.9928
107	H	13.99122	6.168139	-10.0615
108	H	10.06307	13.99373	-6.16734
109	C	7.21193	9.184782	-11.7628
110	C	11.76222	7.215116	-9.18222
111	C	9.18231	11.76485	-7.2132
112	H	7.239504	10.17064	-11.2781
113	H	11.27681	7.241844	-10.1677
114	H	10.1678	11.27941	-7.24008
115	C	8.116339	8.242957	-10.9417
116	C	10.94176	8.120118	-8.2404
117	C	8.240173	10.94417	-8.11771
118	H	7.935581	7.199773	-11.2197
119	H	11.22041	7.939879	-7.19729
120	H	7.197121	11.22275	-7.93703
121	H	7.915129	8.377202	-9.87051
122	H	9.870471	7.918944	-8.37392
123	H	8.373873	9.87292	-7.91646
124	C	14.37883	13.64796	-8.80291
125	H	14.46598	14.28928	-9.68974
126	H	14.13309	14.30461	-7.96028
127	H	15.35324	13.18146	-8.61489
128	H	12.3141	13.03881	-9.12436
129	C	11.81937	5.766241	-8.66674
130	H	10.80112	5.419528	-8.4478
131	H	12.23826	5.06889	-9.40144
132	H	12.41085	5.706878	-7.74501
133	C	13.64298	8.801355	-14.3815
134	H	14.28481	9.687682	-14.4701
135	H	14.29935	7.95837	-14.1363
136	H	13.17483	8.613089	-15.3551
137	C	5.762641	8.670397	-11.8193
138	H	5.415932	8.452592	-10.8008
139	H	5.065801	9.405303	-12.2387
140	H	5.702412	7.74822	-12.41
141	C	8.798989	14.37904	-13.6472
142	H	9.685578	14.46563	-14.289
143	H	7.956173	14.13252	-14.3033
144	H	8.611025	15.35392	-13.1817
145	C	8.667202	11.82246	-5.76421
146	H	8.44845	10.8043	-5.41707
147	H	9.402047	12.24168	-5.0672
148	H	7.745444	12.41388	-5.70482

Ligand Substitution

Aux1R – Ligand Substitution

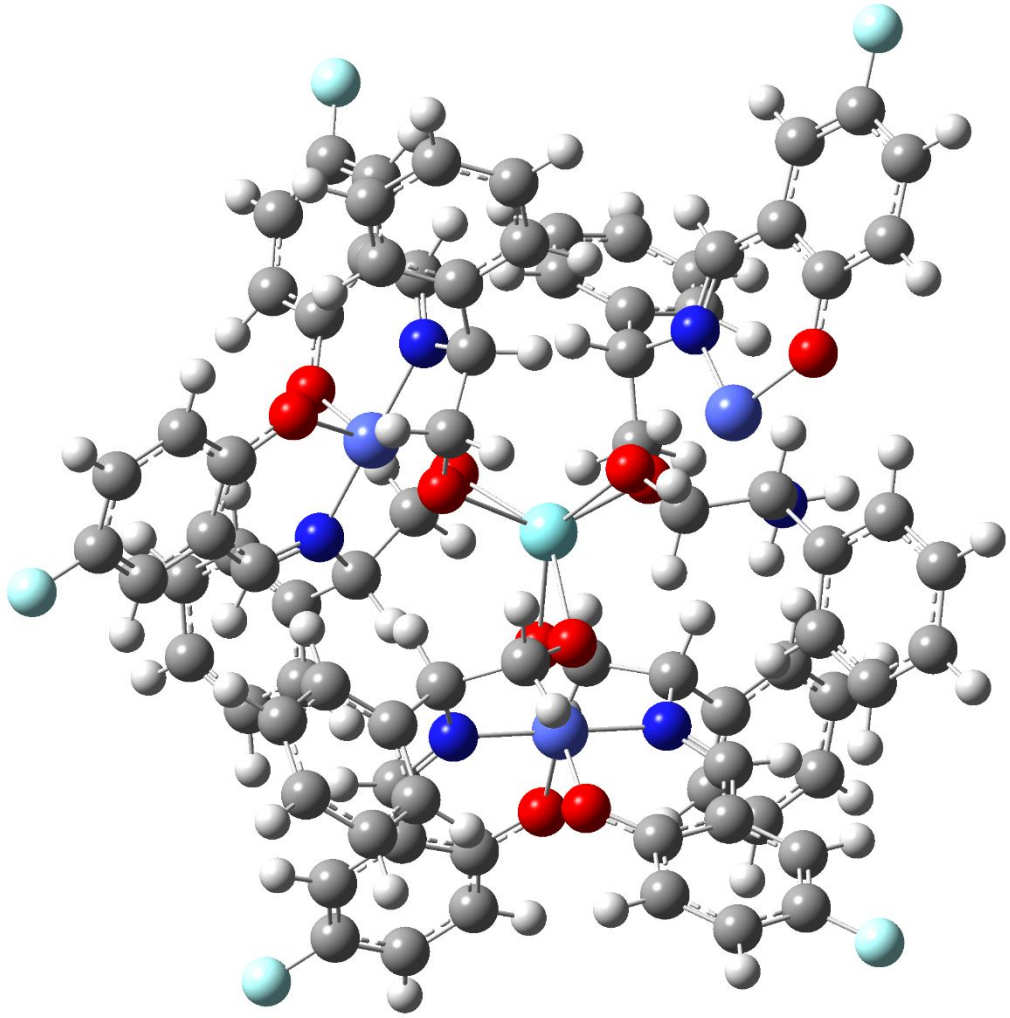

	B3LYP/SDD
**Electronic Energy (EE)**	-5336.711057
**EE + Zero-point Energy**	-5335.313583
**EE + Thermal Energy Correction**	-5335.219260
**EE + Thermal Enthalpy Correction**	-5335.218316
**EE + Thermal Free Energy Correction**	-5335.455648

	B3LYP/SDD			
Tag	Symbol	X	Y	Z
1	Y	10.36412	10.57403	-10.8079
2	Co	9.52571	8.65823	-13.4156
3	Co	13.34826	9.704292	-8.78346
4	Co	8.355825	13.06067	-9.79489
5	F	11.38114	1.982099	-13.4177
6	F	1.513611	13.23039	-10.9128
7	F	5.903552	10.97827	-18.8569
8	F	19.15558	5.973305	-8.94257
9	F	10.56379	18.67009	-6.36806
10	O	8.802347	6.910822	-13.5935

11	O	6.692253	13.12592	-8.87875
12	O	10.34639	10.40329	-13.09
13	O	12.6467	11.21055	-11.0267
14	O	10.02004	12.84494	-10.776
15	O	9.628195	8.864534	-15.3026
16	O	15.19438	9.862465	-8.5401
17	O	8.425951	14.95452	-9.91533
18	O	9.403844	8.613146	-11.471
19	O	11.37015	9.598572	-9.06393
20	O	8.448485	11.1138	-9.67988
21	N	11.36452	8.071958	-13.5245
22	N	13.37637	11.68932	-8.38606
23	N	7.569975	13.14208	-11.5623
24	N	7.681677	9.237429	-13.2967
25	N	13.34666	7.858903	-9.00454
26	N	9.142265	12.98269	-8.02528
27	C	9.480444	5.761911	-13.5518
28	C	5.476264	13.15535	-9.42866
29	C	8.729343	4.544682	-13.5701
30	C	4.348016	13.17046	-8.5499
31	H	7.647971	4.620835	-13.6261
32	H	4.541781	13.17029	-7.48177
33	C	9.353075	3.299064	-13.5238
34	C	3.041882	13.19277	-9.03623
35	H	8.778897	2.377212	-13.537
36	H	2.187649	13.20525	-8.36558
37	C	10.76031	3.244458	-13.4648
38	C	2.835494	13.20678	-10.4304
39	C	11.54036	4.385598	-13.4598
40	C	3.885965	13.20324	-11.3286
41	H	12.62404	4.302996	-13.4307
42	H	3.686665	13.22703	-12.3971
43	C	10.91407	5.66576	-13.4987
44	C	5.226409	13.17176	-10.8442
45	C	11.76572	6.832251	-13.5442
46	C	6.293779	13.20295	-11.8186
47	H	12.83834	6.637603	-13.6305
48	H	5.982339	13.31573	-12.8604
49	C	12.36914	9.189919	-13.654
50	C	14.07474	12.51158	-9.44606
51	C	8.576899	13.25699	-12.682
52	H	12.85799	9.302946	-12.6766
53	H	8.697897	12.25526	-13.1162
54	C	11.54524	10.47424	-13.9167
55	C	13.21221	12.5393	-10.7301
56	C	9.91531	13.62927	-12.0001
57	H	11.2604	10.53713	-14.9694

58	H	13.83579	12.85916	-11.5733
59	H	9.938243	14.69414	-11.7552
60	H	12.13644	11.35288	-13.6362
61	H	12.36606	13.22336	-10.626
62	H	10.75086	13.3891	-12.6695
63	C	13.08277	8.628843	-16.0376
64	C	7.881703	15.57978	-13.4744
65	H	12.0316	8.49993	-16.2862
66	H	7.866215	15.89706	-12.4337
67	C	14.07636	8.456619	-17.0154
68	C	7.575654	16.48919	-14.5
69	H	13.79422	8.200863	-18.0341
70	H	7.3324	17.52026	-14.2544
71	C	15.4381	8.611036	-16.6813
72	C	7.580928	16.06957	-15.8469
73	H	16.20535	8.476028	-17.4399
74	H	7.342777	16.77471	-16.6395
75	C	15.79945	8.933761	-15.36
76	C	7.890152	14.73288	-16.1595
77	H	16.84709	9.047112	-15.0924
78	H	7.889247	14.3992	-17.1941
79	C	14.80325	9.102388	-14.3788
80	C	8.193826	13.82047	-15.1305
81	H	15.08778	9.345739	-13.3562
82	H	8.429276	12.78717	-15.3781
83	C	13.44018	8.955214	-14.7086
84	C	8.195337	14.23505	-13.7826
85	C	8.6916	9.375438	-16.1037
86	C	16.11201	8.895127	-8.64046
87	C	8.946649	15.79425	-9.01978
88	C	9.0044	9.512111	-17.4931
89	C	17.49027	9.233282	-8.4707
90	C	8.926438	17.19153	-9.32724
91	H	9.984495	9.18277	-17.8236
92	H	17.73051	10.27098	-8.25693
93	H	8.475059	17.49294	-10.2673
94	C	8.089071	10.0417	-18.4011
95	C	18.49768	8.274335	-8.57151
96	C	9.458541	18.14026	-8.45661
97	H	8.330792	10.14335	-19.4551
98	H	19.54538	8.531213	-8.4449
99	H	9.441773	19.19991	-8.69415
100	C	6.822141	10.44474	-17.933
101	C	18.13192	6.939391	-8.84159
102	C	10.02392	17.70551	-7.24042
103	C	6.457944	10.32635	-16.605
104	C	16.8161	6.548724	-9.00373

105	C	10.06077	16.37154	-6.88195
106	H	5.465805	10.63447	-16.2846
107	H	16.57981	5.50568	-9.20002
108	H	10.49246	16.0769	-5.92856
109	C	7.388447	9.795312	-15.6645
110	C	15.7735	7.519252	-8.90784
111	C	9.525903	15.38969	-7.76731
112	C	6.952679	9.652671	-14.2938
113	C	14.40904	7.074073	-9.04994
114	C	9.549052	14.0112	-7.33461
115	H	5.903541	9.893069	-14.1009
116	H	14.26354	5.999982	-9.18353
117	H	9.919419	13.83901	-6.32022
118	C	7.066724	9.068326	-11.9285
119	C	11.97974	7.245406	-9.15985
120	C	9.151405	11.60298	-7.4139
121	H	7.058493	10.05642	-11.4499
122	H	11.76845	7.174664	-10.2365
123	H	10.16231	11.19154	-7.54676
124	C	8.055595	8.183672	-11.1315
125	C	11.00006	8.279751	-8.56713
126	C	8.199522	10.75428	-8.29167
127	H	7.928928	7.130954	-11.3949
128	H	11.06555	8.272345	-7.46994
129	H	7.15745	10.96559	-8.04012
130	H	7.879516	8.312133	-10.0568
131	H	9.9754	8.017522	-8.86174
132	H	8.402018	9.687717	-8.13537
133	C	5.655903	8.49905	-11.9347
134	C	11.79021	5.870387	-8.53375
135	C	8.782532	11.55457	-5.93969
136	C	4.625386	9.147976	-11.2237
137	C	11.25876	4.811574	-9.29949
138	C	9.622092	10.89266	-5.02012
139	H	4.83289	10.08058	-10.7025
140	H	11.02382	4.972419	-10.3501
141	H	10.55525	10.449	-5.36472
142	C	3.328744	8.599663	-11.1825
143	C	11.03084	3.54813	-8.72091
144	C	9.262547	10.79605	-3.66188
145	H	2.542776	9.112489	-10.6338
146	H	10.62545	2.742024	-9.32734
147	H	9.918038	10.28199	-2.96327
148	C	3.051176	7.396555	-11.8574
149	C	11.33446	3.32904	-7.36436
150	C	8.058082	11.36717	-3.21094
151	H	2.049878	6.973502	-11.8304

152	H	11.16295	2.353441	-6.91591
153	H	7.778898	11.29638	-2.16253
154	C	4.076724	6.74503	-12.5742
155	C	11.86957	4.380389	-6.59171
156	C	7.216075	12.03481	-4.12476
157	H	3.864836	5.817562	-13.1011
158	H	12.11305	4.215639	-5.54474
159	H	6.286366	12.48032	-3.77899
160	C	5.370569	7.290175	-12.6121
161	C	12.09487	5.640021	-7.17164
162	C	7.573322	12.12699	-5.47995
163	H	6.161858	6.797262	-13.1729
164	H	12.5259	6.439755	-6.57271
165	H	6.930935	12.64956	-6.18567
166	H	14.99799	11.95966	-9.64256
167	C	14.42563	13.92536	-8.98824
168	C	15.77906	14.29446	-8.83497
169	C	13.42279	14.87738	-8.6899
170	C	16.12819	15.58476	-8.39317
171	H	16.55915	13.56902	-9.05828
172	C	13.76784	16.16439	-8.24132
173	H	12.36979	14.62575	-8.80384
174	C	15.12244	16.52307	-8.09317
175	H	17.1756	15.85424	-8.28116
176	H	12.98581	16.88343	-8.012
177	H	15.38782	17.51937	-7.74841
178	H	13.9629	11.67247	-7.54528
179	H	12.45948	12.08093	-8.15193
180	H	13.15308	10.46498	-10.4332

Aux1S – Ligand Substitution

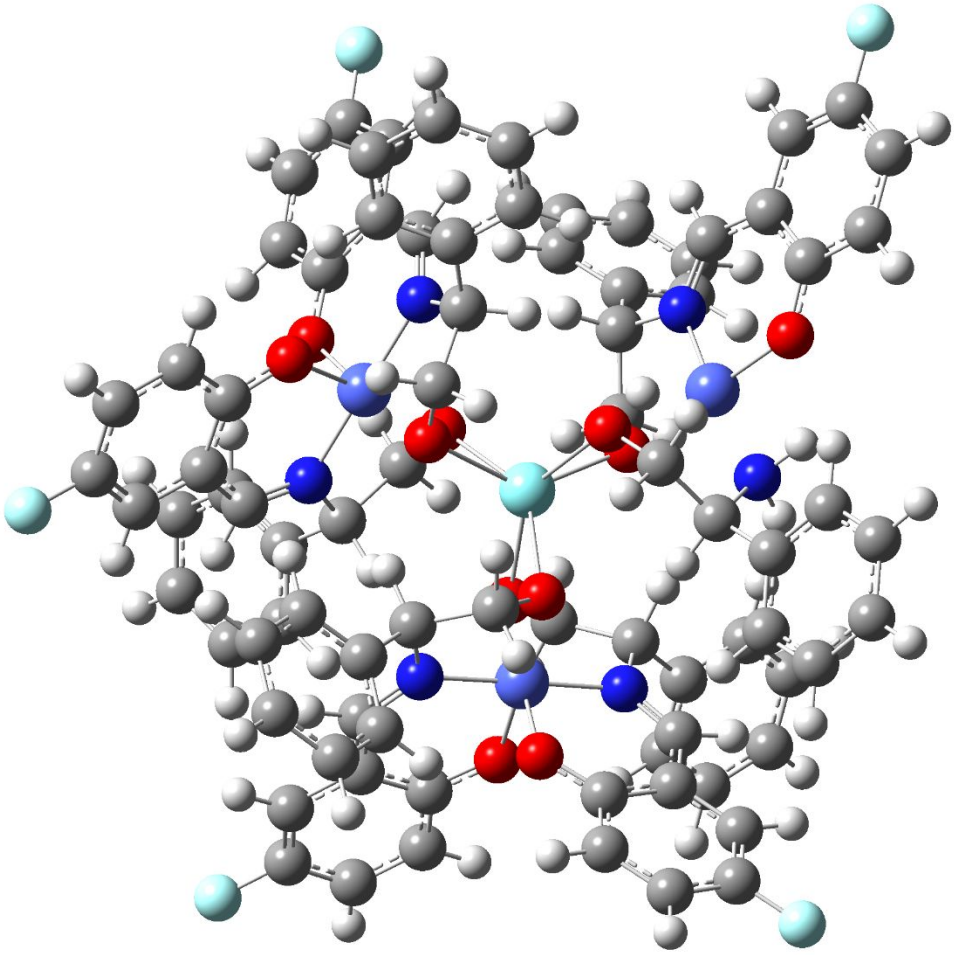

	B3LYP/SDD
**Electronic Energy (EE)**	-5336.712235
**EE + Zero-point Energy**	-5335.314768
**EE + Thermal Energy Correction**	-5335.220480
**EE + Thermal Enthalpy Correction**	-5335.219536
**EE + Thermal Free Energy Correction**	-5335.456875

	B3LYP/SDD			
Tag	Symbol	X	Y	Z
1	Y	0.062776	-0.14156	-0.25391
2	Co	-3.28662	-0.02036	-0.07371
3	Co	2.032549	2.985923	-0.18235
4	Co	1.636923	-3.09124	-0.14015
5	F	-7.41016	5.057001	-2.38384
6	F	8.443624	5.308141	-1.29331
7	F	-1.09874	-8.92314	-2.71179
8	F	-7.13713	-4.99683	2.841101
9	F	7.858577	-3.45175	2.888323
10	O	-4.67362	0.330253	-1.32267
11	O	2.980734	4.252454	-1.18798

12	O	1.971559	-4.46816	-1.40762
13	O	-1.76252	-0.29185	1.114519
14	O	0.988905	1.598718	0.813021
15	O	1.151947	-1.64599	1.074546
16	O	-4.54106	-0.31488	1.322873
17	O	2.583653	-4.0335	1.211211
18	O	-1.89049	0.185817	-1.41665
19	O	1.064567	1.13676	-1.99272
20	O	0.71828	-1.97627	-1.45854
21	N	-3.22189	1.7958	0.605967
22	N	3.496104	2.40192	0.800465
23	N	0.059662	-3.94003	0.594824
24	N	-3.36837	-1.83275	-0.74994
25	N	0.465315	3.832815	-1.17436
26	N	3.214959	-2.24081	-0.8625
27	C	-5.29768	1.491802	-1.52423
28	C	4.299847	4.478192	-1.17941
29	C	1.193579	-5.5197	-1.67048
30	C	-6.33381	1.528938	-2.50993
31	C	4.838487	5.457747	-2.06851
32	C	1.589675	-6.39395	-2.73149
33	H	-6.56761	0.604905	-3.02935
34	H	4.144701	5.986297	-2.71617
35	H	2.507907	-6.15999	-3.26099
36	C	-7.02952	2.702025	-2.79577
37	C	6.204858	5.733994	-2.11154
38	C	0.835339	-7.51374	-3.0778
39	H	-7.81658	2.725954	-3.54386
40	H	6.616219	6.477118	-2.7884
41	H	1.140234	-8.17499	-3.8837
42	C	-6.70228	3.876746	-2.08819
43	C	7.062494	5.024447	-1.24589
44	C	-0.34325	-7.78976	-2.35588
45	C	-5.71804	3.900468	-1.11861
46	C	6.598857	4.072055	-0.35781
47	C	-0.77072	-6.98897	-1.31365
48	H	-5.50043	4.823894	-0.58778
49	H	7.296242	3.556066	0.297633
50	H	-1.67743	-7.24315	-0.77028
51	C	-4.99383	2.708467	-0.82164
52	C	5.203582	3.774602	-0.30504
53	C	-0.01359	-5.8353	-0.95609
54	C	-3.99382	2.772671	0.219482
55	C	4.749189	2.794266	0.650903
56	C	-0.47413	-5.04847	0.16555
57	H	-3.91129	3.732226	0.736665
58	H	5.513546	2.368969	1.304798

59	H	-1.33291	-5.45195	0.709075
60	C	-2.27556	1.9826	1.765544
61	C	3.180032	1.360271	1.842592
62	C	-0.4878	-3.26642	1.829614
63	H	-1.3374	2.387394	1.360057
64	H	3.353803	0.374753	1.387322
65	H	-1.36435	-2.68227	1.52075
66	C	-1.96846	0.55536	2.279363
67	C	1.663587	1.485715	2.098865
68	C	0.599197	-2.25788	2.273882
69	H	-2.80625	0.172239	2.867233
70	H	1.45725	2.38343	2.698797
71	H	1.393524	-2.76591	2.825667
72	H	-1.06509	0.570604	2.901127
73	H	1.316639	0.604019	2.653619
74	H	0.14834	-1.49007	2.913929
75	C	-4.02927	2.65758	3.491078
76	C	4.02706	2.649137	3.883242
77	C	0.033157	-5.18167	3.431933
78	H	-4.65366	1.841359	3.133549
79	H	3.534683	3.541786	3.502628
80	H	1.010892	-5.26525	2.962206
81	C	-4.46498	3.474946	4.546874
82	C	4.726358	2.705611	5.100429
83	C	-0.32326	-6.02795	4.494678
84	H	-5.42743	3.285246	5.015953
85	H	4.759643	3.63599	5.662444
86	H	0.386424	-6.76703	4.85915
87	C	-3.65928	4.540049	5.000821
88	C	5.388819	1.561758	5.591773
89	C	-1.59832	-5.9246	5.089295
90	H	-3.99815	5.170966	5.818861
91	H	5.932277	1.606605	6.53249
92	H	-1.87282	-6.58173	5.910978
93	C	-2.41605	4.783627	4.388229
94	C	5.348344	0.364208	4.85374
95	C	-2.51561	-4.97143	4.609658
96	H	-1.7899	5.603509	4.731339
97	H	5.862412	-0.52103	5.219785
98	H	-3.5034	-4.89037	5.056319
99	C	-1.98214	3.965832	3.326907
100	C	4.647465	0.311109	3.633511
101	C	-2.15925	-4.12544	3.541553
102	H	-1.01808	4.15685	2.857429
103	H	4.622822	-0.61813	3.067059
104	H	-2.87454	-3.39311	3.172093
105	C	-2.78072	2.897042	2.870294

106	C	3.978584	1.44906	3.135871
107	C	-0.88442	-4.22049	2.946334
108	C	-5.13512	-1.46916	1.634388
109	C	3.859297	-3.8529	1.560388
110	C	-6.0241	-1.47916	2.754554
111	C	4.344562	-4.56523	2.701992
112	H	-6.17708	-0.54374	3.283795
113	H	3.651427	-5.22196	3.218258
114	C	-6.68279	-2.64044	3.155612
115	C	5.66047	-4.43293	3.14224
116	H	-7.35856	-2.64374	4.005896
117	H	6.024571	-4.97497	4.010172
118	C	-6.46697	-3.82949	2.430401
119	C	6.531858	-3.57961	2.435744
120	C	-5.62704	-3.87899	1.333806
121	C	6.125393	-2.87755	1.316484
122	H	-5.49602	-4.81255	0.792338
123	H	6.831418	-2.24226	0.787351
124	C	-4.94051	-2.70052	0.918998
125	C	4.779904	-2.99807	0.861045
126	C	-4.0985	-2.79061	-0.25283
127	C	4.408917	-2.28997	-0.34287
128	H	-4.10998	-3.75222	-0.77275
129	H	5.221725	-1.77876	-0.86628
130	C	-2.59232	-2.05361	-2.02693
131	C	-0.38977	3.134502	-2.2048
132	C	2.993599	-1.52199	-2.17006
133	H	-1.64261	-2.53159	-1.751
134	H	-1.17918	2.600048	-1.66692
135	H	2.982356	-0.44571	-1.95246
136	C	-2.25823	-0.63884	-2.55952
137	C	0.444404	2.065413	-2.94609
138	C	1.571856	-1.91719	-2.63917
139	H	-3.12452	-0.20204	-3.06248
140	H	1.239399	2.525628	-3.54561
141	H	1.587952	-2.89401	-3.12788
142	H	-1.41824	-0.69901	-3.26339
143	H	-0.20725	1.481303	-3.60007
144	H	1.191982	-1.159	-3.33282
145	C	-3.30224	-2.91293	-3.06149
146	C	-1.02907	4.096896	-3.20389
147	C	4.055677	-1.79405	-3.22496
148	C	-2.65853	-4.043	-3.60674
149	C	-2.42222	4.073353	-3.42382
150	C	4.688362	-0.72078	-3.8855
151	H	-1.66605	-4.32039	-3.25669
152	H	-3.0442	3.377216	-2.86616

153	H	4.441433	0.303368	-3.60979
154	C	-3.28975	-4.81649	-4.60014
155	C	-3.01711	4.942177	-4.35818
156	C	5.638793	-0.96249	-4.8964
157	H	-2.78375	-5.68734	-5.00909
158	H	-4.09321	4.915643	-4.50992
159	H	6.121462	-0.12633	-5.39629
160	C	-4.57496	-4.4668	-5.05407
161	C	-2.22196	5.845267	-5.08834
162	C	5.967666	-2.28393	-5.25145
163	H	-5.06557	-5.06464	-5.81835
164	H	-2.67994	6.519008	-5.80828
165	H	6.704301	-2.47348	-6.02849
166	C	-5.22651	-3.34074	-4.50859
167	C	-0.82825	5.872222	-4.87983
168	C	5.342351	-3.36143	-4.58978
169	H	-6.22192	-3.07065	-4.85336
170	H	-0.20794	6.566058	-5.44214
171	H	5.599147	-4.3838	-4.85729
172	C	-4.59539	-2.56786	-3.52013
173	C	-0.2364	5.00311	-3.94686
174	C	4.391383	-3.12016	-3.58455
175	H	-5.10011	-1.70645	-3.0877
176	H	0.842493	5.038701	-3.80015
177	H	3.913758	-3.94637	-3.0625
178	H	-0.14062	4.278454	-0.47923
179	H	1.051346	4.558233	-1.6078
180	H	1.777305	1.726532	-1.42636

Aux6R – Ligand Substitution

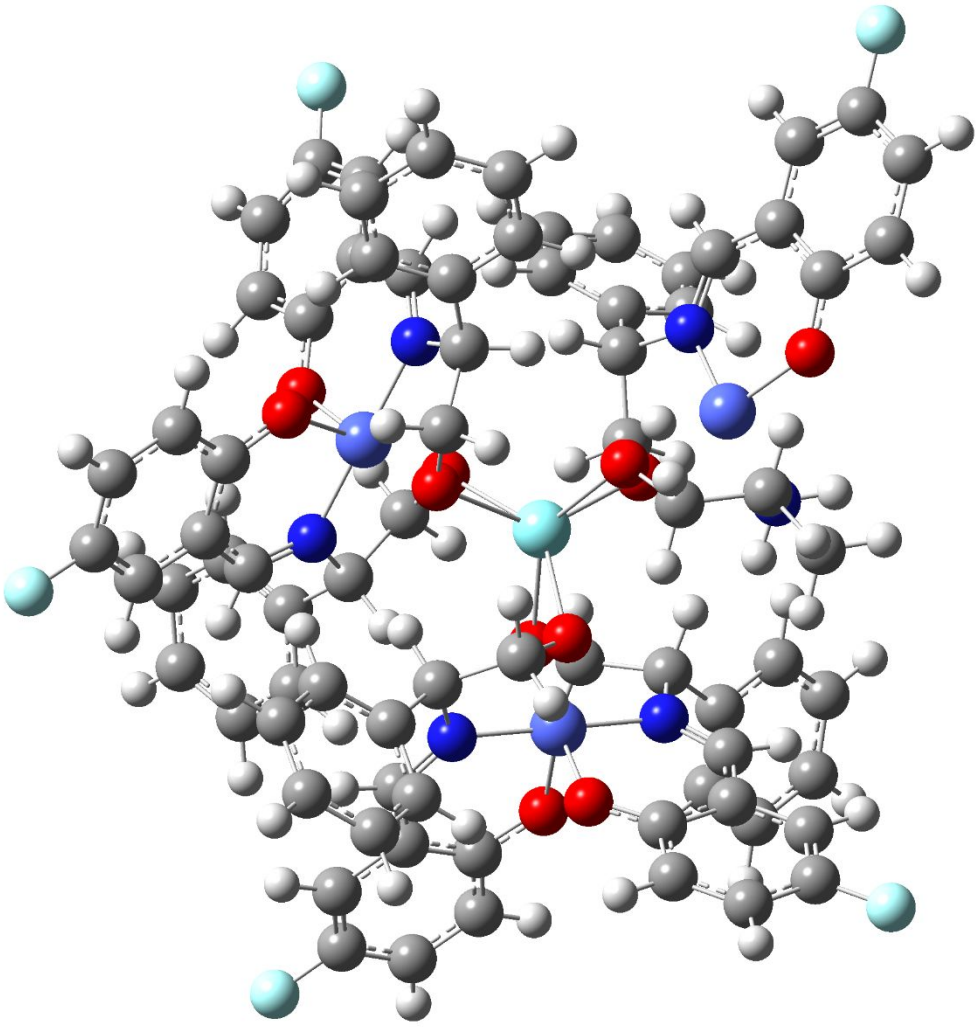

	B3LYP/SDD
**Electronic Energy (EE)**	-5145.005081
**EE + Zero-point Energy**	-5143.661223
**EE + Thermal Energy Correction**	-5143.570034
**EE + Thermal Enthalpy Correction**	-5143.569090
**EE + Thermal Free Energy Correction**	-5143.799079

	B3LYP/SDD			
Tag	Symbol	X	Y	Z
1	Y	10.46311	10.56008	-10.8099
2	Co	9.596297	8.680126	-13.4323
3	Co	13.40124	9.657013	-8.71968
4	Co	8.542506	13.10005	-9.77744
5	F	11.27372	1.957951	-13.4174
6	F	1.693705	13.57859	-10.7658
7	F	6.093645	11.1075	-18.9048
8	F	19.1986	5.907999	-8.67039
9	F	11.3197	18.50153	-6.43949
10	O	8.828464	6.953179	-13.6226
11	O	6.899403	13.22735	-8.83041

12	O	10.4589	10.40185	-13.0932
13	O	12.76372	11.14914	-10.9967
14	O	10.17675	12.83193	-10.79
15	O	9.72389	8.887649	-15.3177
16	O	15.23767	9.809142	-8.40802
17	O	8.700836	14.98993	-9.8718
18	O	9.453633	8.630924	-11.4897
19	O	11.43332	9.555629	-9.06366
20	O	8.555585	11.14837	-9.68646
21	N	11.4201	8.046022	-13.5229
22	N	13.43698	11.65102	-8.34471
23	N	7.727722	13.23509	-11.5285
24	N	7.768174	9.310311	-13.3303
25	N	13.40065	7.811153	-8.93315
26	N	9.360207	12.95716	-8.02724
27	C	9.475503	5.786873	-13.5734
28	C	5.676096	13.31706	-9.35665
29	C	8.692686	4.589909	-13.5999
30	C	4.565732	13.37418	-8.45691
31	H	7.614387	4.694849	-13.6676
32	H	4.778191	13.35543	-7.39255
33	C	9.282511	3.328244	-13.5464
34	C	3.253237	13.45807	-8.91852
35	H	8.684227	2.42196	-13.5653
36	H	2.412817	13.5018	-8.23192
37	C	10.68711	3.236354	-13.472
38	C	3.021989	13.49308	-10.3085
39	C	11.49712	4.356339	-13.4591
40	C	4.054552	13.45137	-11.2262
41	H	12.57788	4.244901	-13.4187
42	H	3.837035	13.49319	-12.2905
43	C	10.90551	5.652739	-13.5049
44	C	5.40107	13.35737	-10.767
45	C	11.7884	6.796075	-13.5403
46	C	6.450609	13.3507	-11.7608
47	H	12.85633	6.572971	-13.6158
48	H	6.124868	13.48252	-12.796
49	C	12.45499	9.137615	-13.639
50	C	14.16313	12.4568	-9.39062
51	C	8.717483	13.31029	-12.6661
52	H	12.93598	9.23639	-12.656
53	H	8.78936	12.30544	-13.1043
54	C	11.66741	10.44357	-13.9073
55	C	13.34207	12.47256	-10.6986
56	C	10.08182	13.62476	-12.008
57	H	11.39539	10.51621	-14.9628
58	H	13.9905	12.77049	-11.5318

59	H	10.15174	14.68699	-11.7599
60	H	12.2784	11.30597	-13.6186
61	H	12.50289	13.17144	-10.6199
62	H	10.89432	13.35349	-12.6936
63	C	13.18042	8.56355	-16.0158
64	C	8.09958	15.66097	-13.4394
65	H	12.12908	8.463079	-16.2766
66	H	8.108244	15.97381	-12.3973
67	C	14.18016	8.367491	-16.9828
68	C	7.815917	16.58653	-14.4571
69	H	13.90294	8.121732	-18.0053
70	H	7.614187	17.6246	-14.2035
71	C	15.54164	8.484983	-16.6329
72	C	7.789689	16.17398	-15.8059
73	H	16.3136	8.33147	-17.3832
74	H	7.568772	16.89161	-16.5923
75	C	15.89645	8.794829	-15.3068
76	C	8.045083	14.8283	-16.1285
77	H	16.94362	8.879664	-15.0271
78	H	8.019992	14.50029	-17.1646
79	C	14.89397	8.987441	-14.3364
80	C	8.32628	13.8998	-15.1074
81	H	15.17317	9.220767	-13.31
82	H	8.520714	12.85992	-15.363
83	C	13.53128	8.877221	-14.682
84	C	8.358624	14.30693	-13.7578
85	C	8.809217	9.424138	-16.1271
86	C	16.15567	8.839409	-8.47657
87	C	9.34271	15.77855	-9.00802
88	C	9.138711	9.552866	-17.5135
89	C	17.52858	9.174383	-8.26314
90	C	9.428718	17.17278	-9.3172
91	H	10.11228	9.196417	-17.835
92	H	17.76504	10.21263	-8.0477
93	H	8.959554	17.51399	-10.2347
94	C	8.247353	10.10877	-18.4295
95	C	18.53587	8.212195	-8.32816
96	C	10.08193	18.07059	-8.47517
97	H	8.501837	10.20456	-19.481
98	H	19.57972	8.466653	-8.16911
99	H	10.14551	19.12811	-8.71433
100	C	6.987926	10.5471	-17.9729
101	C	18.17508	6.877465	-8.60602
102	C	10.66415	17.5877	-7.28543
103	C	6.607826	10.43763	-16.6486
104	C	16.86426	6.490191	-8.81104
105	C	10.60284	16.2549	-6.92634

106	H	5.621723	10.77313	-16.3371
107	H	16.63156	5.447383	-9.0128
108	H	11.04897	15.92359	-5.99175
109	C	7.514004	9.879542	-15.6999
110	C	15.82194	7.464055	-8.75237
111	C	9.945929	15.32368	-7.78409
112	C	7.0611	9.747789	-14.3337
113	C	14.46183	7.02299	-8.94067
114	C	9.869531	13.94831	-7.3498
115	H	6.01775	10.01894	-14.1505
116	H	14.31777	5.949251	-9.07842
117	H	10.25358	13.74491	-6.3459
118	C	7.136751	9.158621	-11.9676
119	C	12.0388	7.201079	-9.13716
120	C	9.283116	11.57956	-7.41489
121	H	7.157297	10.14532	-11.4862
122	H	11.86706	7.12962	-10.221
123	H	10.27022	11.11102	-7.53645
124	C	8.08993	8.240588	-11.165
125	C	11.04076	8.238203	-8.5814
126	C	8.293459	10.78365	-8.30142
127	H	7.934229	7.193334	-11.4345
128	H	11.066	8.229739	-7.48239
129	H	7.261974	11.03642	-8.04525
130	H	7.907658	8.36988	-10.0914
131	H	10.02706	7.980001	-8.91475
132	H	8.449798	9.707293	-8.15865
133	C	5.707923	8.636872	-11.9861
134	C	11.8219	5.827135	-8.5174
135	C	8.898868	11.55914	-5.9439
136	C	4.694028	9.319222	-11.2825
137	C	11.30631	4.771994	-9.2989
138	C	9.679986	10.83717	-5.01789
139	H	4.928633	10.24342	-10.7577
140	H	11.1034	4.934323	-10.356
141	H	10.5801	10.32494	-5.35541
142	C	3.379374	8.814774	-11.2526
143	C	11.05196	3.510252	-8.72762
144	C	9.303959	10.76863	-3.6624
145	H	2.606732	9.352709	-10.709
146	H	10.6591	2.707089	-9.3461
147	H	9.913773	10.20739	-2.9587
148	C	3.0671	7.622485	-11.9316
149	C	11.31278	3.289229	-7.3625
150	C	8.142337	11.42885	-3.22073
151	H	2.052027	7.233151	-11.9131
152	H	11.1208	2.314946	-6.91951

153	H	7.850822	11.38016	-2.1744
154	C	4.075972	6.937735	-12.6411
155	C	11.83141	4.336972	-6.5739
156	C	7.359627	12.1569	-4.14113
157	H	3.837378	6.018511	-13.1709
158	H	12.04166	4.170728	-5.51998
159	H	6.463472	12.67122	-3.80223
160	C	5.387686	7.439137	-12.6676
161	C	12.0831	5.594926	-7.14662
162	C	7.732488	12.22086	-5.49379
163	H	6.166356	6.920401	-13.2228
164	H	12.50112	6.391852	-6.53489
165	H	7.137	12.78807	-6.20598
166	C	14.46131	13.89915	-8.93764
167	H	14.98968	14.45396	-9.72458
168	H	13.53615	14.44472	-8.70394
169	H	15.10035	13.90205	-8.04543
170	H	15.10112	11.91595	-9.54874
171	H	12.51904	12.05536	-8.13515
172	H	14.00339	11.62012	-7.49048
173	H	13.2485	10.40564	-10.3883

Aux6S – Ligand Substitution

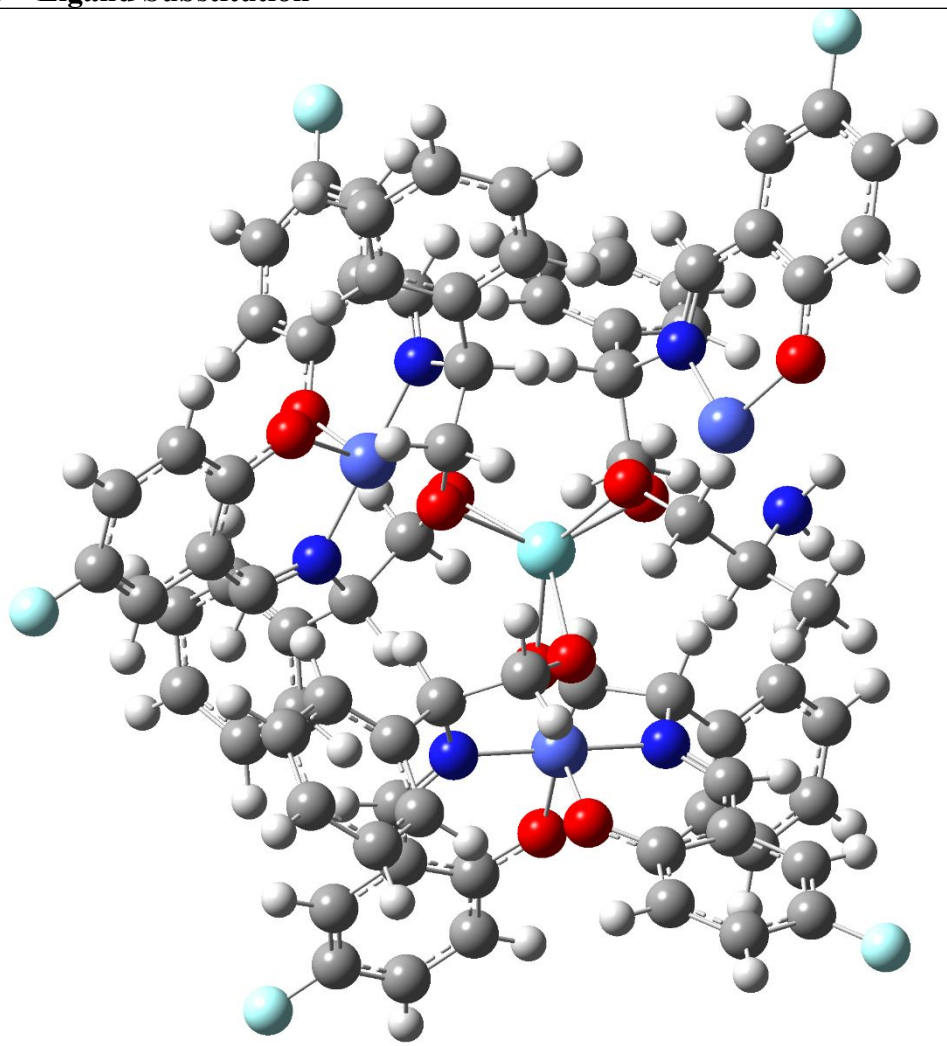

	B3LYP/SDD	B3LYP/SDD, pcm=chloroform
**Electronic Energy (EE)**	-5145.006674	-5145.043966
**EE + Zero-point Energy**	-5143.662841	-5143.698964
**EE + Thermal Energy Correction**	-5143.571682	-5143.608142
**EE + Thermal Enthalpy Correction**	-5143.570738	-5143.607197
**EE + Thermal Free Energy Correction**	-5143.800295	-5143.833109

	B3LYP/SDD				B3LYP/SDD, pcm=chloroform			
Tag	Symbol	X	Y	Z	Symbol	X	Y	Z
1	Y	10.54718	10.59296	-10.742	Y	10.5237	10.57938	-10.7547
2	Co	9.671878	8.693629	-13.3532	Co	9.662278	8.702558	-13.4013
3	Co	13.50342	9.581815	-8.76237	Co	13.48374	9.526944	-8.70674
4	Co	8.616243	13.15663	-9.77896	Co	8.625517	13.18371	-9.80234
5	F	11.25163	1.944774	-13.425	F	11.39036	1.989576	-13.3151
6	F	1.796651	13.61238	-10.959	F	1.785783	13.32282	-10.9539
7	F	6.208874	11.14714	-18.8393	F	6.158352	11.27188	-18.8166
8	F	19.11647	5.613375	-9.42984	F	19.07806	5.468791	-9.04335
9	F	11.06886	18.6748	-6.37044	F	11.1759	18.72308	-6.48482
10	O	8.877794	6.977194	-13.5357	O	8.907263	6.966255	-13.608
11	O	6.947894	13.29319	-8.87813	O	6.959223	13.35018	-8.89506

12	O	10.55769	10.40413	-13.0214	O	10.51741	10.41876	-13.0502
13	O	12.84969	11.15839	-10.9503	O	12.85184	11.10378	-10.9412
14	O	10.27693	12.86877	-10.7456	O	10.27631	12.8709	-10.7745
15	O	9.803627	8.890616	-15.2395	O	9.772703	8.921328	-15.2918
16	O	15.36972	9.64815	-8.62369	O	15.35081	9.570414	-8.4861
17	O	8.769432	15.0451	-9.90675	O	8.786495	15.07521	-9.94751
18	O	9.52679	8.661044	-11.4096	O	9.523152	8.637132	-11.4614
19	O	11.51234	9.577488	-8.98995	O	11.50557	9.531261	-9.01001
20	O	8.632149	11.20673	-9.6523	O	8.615095	11.24042	-9.65647
21	N	11.4861	8.031248	-13.4443	N	11.48995	8.077411	-13.5109
22	N	13.69454	11.53119	-8.21588	N	13.66657	11.48992	-8.19769
23	N	7.849359	13.26267	-11.5537	N	7.847287	13.26811	-11.5719
24	N	7.851482	9.347329	-13.2561	N	7.831305	9.325212	-13.2906
25	N	13.40628	7.750783	-9.05816	N	13.38354	7.691529	-8.96263
26	N	9.380007	13.05386	-8.00004	N	9.407905	13.10121	-8.03192
27	C	9.507846	5.800865	-13.509	C	9.56136	5.803595	-13.5295
28	C	5.739148	13.37312	-9.43879	C	5.744123	13.33688	-9.44973
29	C	8.707492	4.61589	-13.546	C	8.786475	4.60102	-13.5339
30	C	4.604769	13.43984	-8.57036	C	4.61207	13.36208	-8.57545
31	H	7.630306	4.737057	-13.6034	H	7.706923	4.693865	-13.5992
32	H	4.788691	13.43515	-7.50049	H	4.798855	13.39039	-7.50644
33	C	9.279568	3.345167	-13.5162	C	9.385295	3.343322	-13.4608
34	C	3.305168	13.5163	-9.06856	C	3.306795	13.35467	-9.06702
35	H	8.668164	2.447902	-13.5441	H	8.790743	2.43451	-13.4629
36	H	2.446309	13.56791	-8.40572	H	2.4516	13.37248	-8.39785
37	C	10.68319	3.232076	-13.4549	C	10.78976	3.267118	-13.3878
38	C	3.111983	13.53383	-10.4646	C	3.110832	13.32808	-10.4616
39	C	11.50921	4.340124	-13.4311	C	11.5942	4.391541	-13.3923
40	C	4.169288	13.48173	-11.3532	C	4.163204	13.3116	-11.3578
41	H	12.58846	4.21287	-13.3996	H	12.67573	4.291248	-13.3493
42	H	3.980703	13.50988	-12.4235	H	3.97075	13.302	-12.4275
43	C	10.93603	5.645286	-13.4531	C	10.99259	5.68151	-13.4583
44	C	5.502782	13.39477	-10.8565	C	5.500911	13.30893	-10.8666
45	C	11.83532	6.776232	-13.4772	C	11.86934	6.831398	-13.5132
46	C	6.579077	13.37623	-11.8214	C	6.573501	13.32376	-11.8384
47	H	12.8995	6.538245	-13.5579	H	12.93805	6.614675	-13.588
48	H	6.281538	13.49722	-12.8663	H	6.26657	13.41394	-12.8834
49	C	12.53737	9.108234	-13.5514	C	12.51876	9.173654	-13.6408
50	C	13.23625	12.69936	-9.04614	C	13.24317	12.66089	-9.04701
51	C	8.869315	13.32954	-12.6654	C	8.859945	13.34604	-12.6901
52	H	13.00984	9.200046	-12.5637	H	13.03171	9.251174	-12.6737
53	H	8.952263	12.32115	-13.0932	H	8.928275	12.34437	-13.134
54	C	11.77108	10.42405	-13.8299	C	11.72243	10.48231	-13.8675
55	C	13.49481	12.41228	-10.5363	C	13.50691	12.35941	-10.5323
56	C	10.21666	13.6481	-11.9733	C	10.216	13.64651	-12.0055
57	H	11.50459	10.49516	-14.8869	H	11.44576	10.5816	-14.92
58	H	14.57122	12.33111	-10.7413	H	14.58235	12.26834	-10.7342

59	H	10.28294	14.71296	-11.7353	H	10.2974	14.71127	-11.7717
60	H	12.39343	11.27788	-13.5413	H	12.33085	11.34028	-13.5616
61	H	13.06756	13.21133	-11.1461	H	13.08733	13.15234	-11.1542
62	H	11.0455	13.36674	-12.6353	H	11.0381	13.35622	-12.6711
63	C	13.28008	8.534846	-15.9229	C	13.17518	8.636726	-16.0494
64	C	8.275865	15.6749	-13.4733	C	8.25689	15.70585	-13.4508
65	H	12.23025	8.458357	-16.1976	H	12.11778	8.532424	-16.2808
66	H	8.260855	15.99622	-12.4338	H	8.243484	16.00708	-12.4057
67	C	14.28718	8.323192	-16.8789	C	14.14608	8.466219	-17.0511
68	C	8.018433	16.59218	-14.5053	C	7.996369	16.64395	-14.4641
69	H	14.01744	8.090584	-17.9064	H	13.83898	8.233648	-18.068
70	H	7.813381	17.63292	-14.2657	H	7.787677	17.67861	-14.2029
71	C	15.64639	8.40781	-16.511	C	15.51713	8.595495	-16.7426
72	C	8.022688	16.1679	-15.8507	C	8.004276	16.24778	-15.8186
73	H	16.42419	8.242142	-17.2526	H	16.26663	8.463519	-17.5191
74	H	7.821931	16.8791	-16.6483	H	7.80239	16.97461	-16.6015
75	C	15.99119	8.700407	-15.1784	C	15.90951	8.891391	-15.423
76	C	8.28195	14.81883	-16.1555	C	8.270283	14.90556	-16.1506
77	H	17.03629	8.759414	-14.8848	H	16.9637	8.98768	-15.1755
78	H	8.279675	14.48168	-17.1889	H	8.273323	14.59096	-17.1911
79	C	14.98125	8.909012	-14.2191	C	14.93542	9.057012	-14.4184
80	C	8.536849	13.89866	-15.1201	C	8.526519	13.96484	-15.1334
81	H	15.25287	9.129149	-13.1879	H	15.24464	9.280966	-13.3988
82	H	8.73374	12.85597	-15.3619	H	8.727249	12.92797	-15.3959
83	C	13.62101	8.831918	-14.5826	C	13.56334	8.934313	-14.7221
84	C	8.539002	14.31753	-13.7736	C	8.524857	14.35555	-13.778
85	C	8.89775	9.437502	-16.0518	C	8.865973	9.495531	-16.0866
86	C	16.23683	8.648917	-8.82318	C	16.21389	8.55626	-8.6252
87	C	9.332099	15.86375	-9.0162	C	9.373321	15.89992	-9.07658
88	C	9.231238	9.557618	-17.438	C	9.195598	9.656319	-17.4697
89	C	17.63634	8.922581	-8.73713	C	17.61259	8.814334	-8.48377
90	C	9.393084	17.25648	-9.33755	C	9.441744	17.28852	-9.41459
91	H	10.20044	9.186957	-17.7567	H	10.16538	9.30214	-17.8047
92	H	17.93671	9.941316	-8.50882	H	17.9202	9.833419	-8.26602
93	H	8.968504	17.57309	-10.2851	H	9.009347	17.60201	-10.3596
94	C	8.348702	10.12225	-18.3572	C	8.307625	10.24344	-18.371
95	C	18.59029	7.925468	-8.9386	C	18.56332	7.801256	-8.62224
96	C	9.967378	18.18343	-8.46988	C	10.03703	18.22055	-8.56498
97	H	8.606052	10.21118	-19.4087	H	8.564744	10.36336	-19.4193
98	H	19.65466	8.132278	-8.87565	H	19.62595	7.999931	-8.51787
99	H	10.01174	19.23991	-8.71785	H	10.08608	19.27311	-8.82791
100	C	7.094327	10.57828	-17.9041	C	7.054787	10.67971	-17.8978
101	C	18.14688	6.618319	-9.22794	C	18.11115	6.496454	-8.90334
102	C	10.49331	17.73176	-7.24232	C	10.57618	17.77314	-7.34291
103	C	6.710691	10.47794	-16.5801	C	6.673747	10.54352	-16.576
104	C	16.80689	6.291068	-9.31588	C	16.77198	6.181012	-9.04384
105	C	10.45367	16.40131	-6.87177	C	10.53323	16.44711	-6.95563

106	H	5.728485	10.82715	-16.2714	H	5.692788	10.88089	-16.2514
107	H	16.51131	5.26737	-9.53248	H	16.46994	5.157847	-9.25386
108	H	10.85566	16.09376	-5.90945	H	10.94984	16.14241	-5.99897
109	C	7.608021	9.911388	-15.6282	C	7.576848	9.953909	-15.6448
110	C	15.81788	7.301714	-9.11811	C	15.78931	7.207597	-8.90975
111	C	9.877165	15.441	-7.75462	C	9.933926	15.4848	-7.81946
112	C	7.151239	9.789372	-14.2623	C	7.125077	9.794173	-14.2795
113	C	14.42794	6.922431	-9.1907	C	14.39729	6.844815	-9.0352
114	C	9.826221	14.0674	-7.31169	C	9.879386	14.11415	-7.36131
115	H	6.11039	10.07191	-14.082	H	6.086226	10.074	-14.0874
116	H	14.22576	5.860679	-9.34794	H	14.18786	5.783796	-9.18289
117	H	10.1745	13.88531	-6.29111	H	10.2524	13.93744	-6.3494
118	C	7.214198	9.204413	-11.8952	C	7.201933	9.160017	-11.9284
119	C	12.00702	7.200035	-9.15842	C	11.98329	7.14979	-9.11482
120	C	9.323115	11.67977	-7.38001	C	9.366334	11.73013	-7.4048
121	H	7.238236	10.19287	-11.418	H	7.216103	10.1467	-11.4478
122	H	11.74395	7.153667	-10.2247	H	11.77255	7.087122	-10.1916
123	H	10.31921	11.22822	-7.49421	H	10.35966	11.28112	-7.54152
124	C	8.159253	8.284197	-11.0851	C	8.161427	8.245733	-11.13
125	C	11.09941	8.265991	-8.50788	C	11.0587	8.23351	-8.52306
126	C	8.350134	10.86426	-8.26637	C	8.36866	10.91112	-8.25852
127	H	7.995583	7.236529	-11.3482	H	8.004526	7.197485	-11.3965
128	H	11.20397	8.230765	-7.41423	H	11.11325	8.216825	-7.42536
129	H	7.314044	11.1179	-8.02965	H	7.339434	11.16909	-7.99728
130	H	7.975957	8.42149	-10.0126	H	7.986292	8.375644	-10.0553
131	H	10.053	8.057801	-8.76814	H	10.02289	8.029796	-8.82472
132	H	8.508314	9.791154	-8.10235	H	8.52648	9.838958	-8.09144
133	C	5.782703	8.689931	-11.9177	C	5.778032	8.623864	-11.9465
134	C	11.78955	5.825621	-8.54012	C	11.7297	5.786058	-8.48653
135	C	8.927434	11.65616	-5.91197	C	9.014201	11.71069	-5.92523
136	C	4.769578	9.379931	-11.2205	C	4.762017	9.283178	-11.2236
137	C	11.17214	4.802115	-9.28923	C	11.17206	4.744224	-9.25772
138	C	9.707971	10.94354	-4.97841	C	9.846461	11.03491	-5.00846
139	H	5.006876	10.30496	-10.6984	H	4.989891	10.20331	-10.6892
140	H	10.89075	4.987802	-10.3242	H	10.96161	4.910061	-10.3126
141	H	10.61632	10.4406	-5.30773	H	10.76236	10.56151	-5.35895
142	C	3.452189	8.882512	-11.1942	C	3.454166	8.76031	-11.1865
143	C	10.91844	3.542041	-8.71403	C	10.88678	3.491848	-8.6788
144	C	9.319474	10.8687	-3.62674	C	9.501454	10.96225	-3.64438
145	H	2.680123	9.426643	-10.656	H	2.680357	9.279211	-10.6264
146	H	10.44657	2.763021	-9.30764	H	10.45964	2.699379	-9.28823
147	H	9.929079	10.31416	-2.91756	H	10.15111	10.43705	-2.94878
148	C	3.136293	7.68971	-11.8706	C	3.149822	7.572472	-11.8784
149	C	11.28244	3.290734	-7.37809	C	11.15974	3.266299	-7.31596
150	C	8.144793	11.51292	-3.19618	C	8.318313	11.57136	-3.18416
151	H	2.119041	7.305944	-11.8551	H	2.140553	7.169001	-11.8542
152	H	11.09125	2.317394	-6.93269	H	10.94368	2.299967	-6.867

153	H	7.843341	11.45925	-2.15294	H	8.050243	11.51879	-2.13192
154	C	4.144369	6.997434	-12.5739	C	4.160886	6.910518	-12.6073
155	C	11.90392	4.306485	-6.62262	C	11.72083	4.300808	-6.53764
156	C	7.361308	12.23042	-4.12413	C	7.483296	12.25271	-4.09524
157	H	3.902929	6.077907	-13.102	H	3.93009	5.994675	-13.1461
158	H	12.19449	4.116339	-5.59207	H	11.93888	4.131983	-5.48588
159	H	6.454314	12.73147	-3.79442	H	6.570175	12.72673	-3.74379
160	C	5.458824	7.491771	-12.5967	C	5.465985	7.430304	-12.6394
161	C	12.15455	5.562989	-7.19888	C	12.00199	5.549678	-7.1182
162	C	7.747063	12.30048	-5.47284	C	7.826822	12.32061	-5.45615
163	H	6.237277	6.96765	-13.1471	H	6.244942	6.925182	-13.206
164	H	12.65185	6.334624	-6.61459	H	12.44782	6.335501	-6.51204
165	H	7.149402	12.85939	-6.18987	H	7.18663	12.85239	-6.15669
166	C	13.90858	14.01827	-8.6175	C	13.9469	13.96457	-8.62246
167	H	13.51762	14.86402	-9.19712	H	13.58652	14.81389	-9.21644
168	H	13.72044	14.22842	-7.55666	H	13.75068	14.18667	-7.56589
169	H	14.99675	13.97376	-8.7664	H	15.03455	13.88804	-8.75872
170	H	12.15305	12.79006	-8.9149	H	12.16319	12.78279	-8.9207
171	H	13.29868	11.59525	-7.27298	H	13.23729	11.56999	-7.27015
172	H	14.72086	11.50134	-8.14103	H	14.68765	11.47987	-8.07756
173	H	13.31349	10.37041	-10.3756	H	13.29648	10.33038	-10.3749

Aux9R – Ligand Substitution

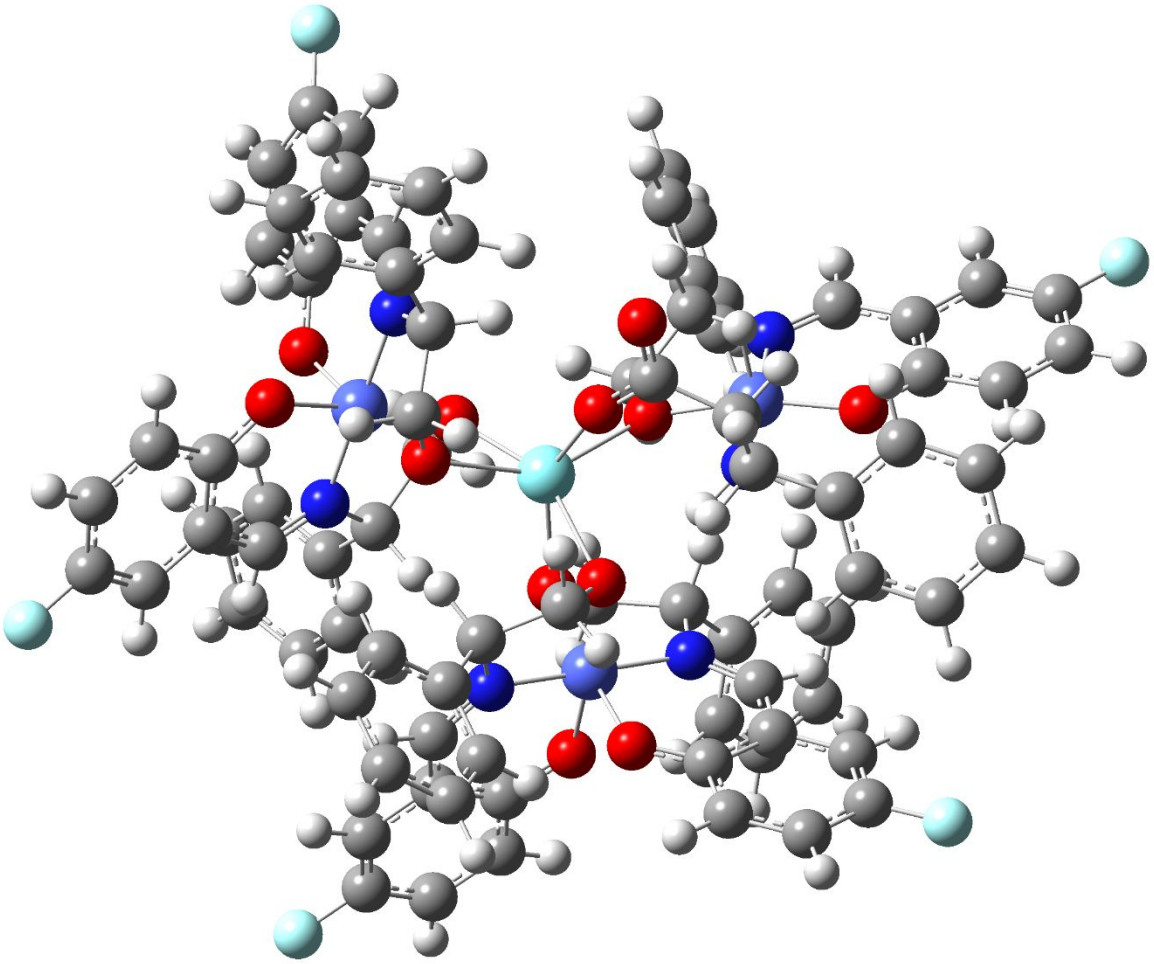

	B3LYP/SDD
**Electronic Energy (EE)**	-5450.07581
**EE + Zero-point Energy**	-5448.66849
**EE + Thermal Energy Correction**	-5448.57246
**EE + Thermal Enthalpy Correction**	-5448.57151
**EE + Thermal Free Energy Correction**	-5448.81333

	B3LYP/SDD			
Tag	Symbol	X	Y	Z
1	Y	10.46716	10.33688	-11.3743
2	Co	9.165059	8.53797	-13.8655
3	Co	13.11992	9.980382	-8.71477
4	Co	8.760326	12.86876	-9.90419
5	F	11.4954	2.049851	-14.5002
6	F	1.956981	13.38834	-11.1237
7	F	4.408701	10.99851	-18.2733
8	F	17.37509	8.610994	-3.44454
9	F	11.89247	17.74135	-6.10367
10	O	8.546342	6.746518	-14.0026
11	O	7.086743	12.96392	-9.00414

12	O	9.907734	10.32564	-13.5957
13	O	12.49302	10.8471	-11.998
14	O	10.41438	12.62322	-10.8811
15	O	8.854038	8.841124	-15.7134
16	O	14.27807	10.79035	-7.50948
17	O	8.991957	14.7536	-9.79418
18	O	9.454175	8.363342	-11.9337
19	O	11.56599	9.320273	-9.63406
20	O	8.685842	10.92252	-10.0172
21	N	10.97484	8.116879	-14.3946
22	N	13.11684	11.80017	-9.54719
23	N	8.021375	13.21326	-11.6532
24	N	7.357154	8.982326	-13.3389
25	N	13.22549	8.281765	-7.89502
26	N	9.524752	12.52559	-8.1626
27	C	9.309337	5.657523	-14.1336
28	C	5.883419	13.07309	-9.57384
29	C	8.669212	4.38183	-14.0444
30	C	4.736224	13.03844	-8.72068
31	H	7.595362	4.36769	-13.8851
32	H	4.905209	12.93975	-7.65276
33	C	9.386567	3.191682	-14.1622
34	C	3.441487	13.1392	-9.22874
35	H	8.896271	2.225063	-14.0915
36	H	2.573162	13.11454	-8.57672
37	C	10.77591	3.256046	-14.3841
38	C	3.266466	13.28505	-10.619
39	C	11.44725	4.459594	-14.4986
40	C	4.337011	13.33727	-11.4924
41	H	12.51741	4.469191	-14.6905
42	H	4.162671	13.46774	-12.5576
43	C	10.72731	5.681881	-14.3699
44	C	5.664789	13.22716	-10.9866
45	C	11.45812	6.917897	-14.5472
46	C	6.754654	13.33272	-11.9312
47	H	12.49928	6.817809	-14.8665
48	H	6.471955	13.56168	-12.9622
49	C	11.8355	9.319735	-14.6882
50	C	14.14985	11.98424	-10.6284
51	C	9.054904	13.41482	-12.7356
52	H	12.5331	9.43475	-13.8514
53	H	9.082832	12.4847	-13.3202
54	C	10.88773	10.54333	-14.659
55	C	13.7581	11.21299	-11.9084
56	C	10.41211	13.56227	-12.0003
57	H	10.36922	10.65005	-15.6149
58	H	10.53278	14.5818	-11.6258

59	H	11.47028	11.44601	-14.4452
60	H	11.23853	13.32093	-12.6801
61	C	12.03879	8.80427	-17.1879
62	C	8.530819	15.87721	-13.1534
63	H	11.0009	8.478612	-17.1805
64	H	8.501386	16.02146	-12.0754
65	C	12.78495	8.788955	-18.3786
66	C	8.314353	16.95738	-14.0246
67	H	12.32297	8.447575	-19.3025
68	H	8.126162	17.94937	-13.6206
69	C	14.12944	9.215215	-18.3799
70	C	8.33828	16.76043	-15.4212
71	H	14.70517	9.20273	-19.3027
72	H	8.170661	17.5981	-16.0939
73	C	14.72249	9.655383	-17.1812
74	C	8.573843	15.47367	-15.9397
75	H	15.75814	9.987361	-17.175
76	H	8.587233	15.31192	-17.0147
77	C	13.98002	9.665806	-15.9841
78	C	8.785747	14.38994	-15.0657
79	H	14.4332	10.01484	-15.0572
80	H	8.961005	13.39508	-15.4716
81	C	12.63461	9.238073	-15.9812
82	C	8.770719	14.58191	-13.6688
83	C	7.757606	9.360383	-16.2678
84	C	15.01285	10.22082	-6.54256
85	C	9.679161	15.41799	-8.86515
86	C	7.775066	9.604788	-17.6774
87	C	15.90266	11.04463	-5.79318
88	C	9.855427	16.82712	-9.04433
89	H	8.679407	9.350357	-18.2211
90	H	15.95756	12.09717	-6.05287
91	H	9.404584	17.28359	-9.91981
92	C	6.674436	10.14681	-18.3391
93	C	16.68244	10.5117	-4.76796
94	C	10.57976	17.59557	-8.13496
95	H	6.691537	10.33152	-19.4093
96	H	17.36618	11.13083	-4.1949
97	H	10.719	18.66291	-8.27954
98	C	5.516769	10.4536	-17.596
99	C	16.58087	9.13435	-4.47832
100	C	11.13957	16.96369	-7.00626
101	C	5.437218	10.23106	-16.2342
102	C	15.73393	8.291098	-5.17233
103	C	10.98216	15.61283	-6.76426
104	H	4.521445	10.46689	-15.6976
105	H	15.68627	7.23565	-4.91717

106	H	11.4177	15.16471	-5.87466
107	C	6.558986	9.685695	-15.5443
108	C	14.92747	8.823922	-6.22149
109	C	10.2541	14.81366	-7.69304
110	C	6.423515	9.432523	-14.1284
111	C	14.03878	7.932351	-6.9258
112	C	10.0905	13.41128	-7.39102
113	H	5.432636	9.617091	-13.7042
114	H	14.05354	6.885962	-6.6149
115	H	10.46336	13.0866	-6.41541
116	C	7.051477	8.71205	-11.8885
117	C	12.42652	7.252277	-8.65339
118	C	9.380853	11.10147	-7.6957
119	H	7.10503	9.675509	-11.3643
120	H	13.03397	6.997878	-9.53296
121	H	10.3589	10.6203	-7.83289
122	C	8.219305	7.835666	-11.3748
123	C	11.17667	7.998432	-9.14741
124	C	8.390359	10.43451	-8.68268
125	H	8.087153	6.799042	-11.6938
126	H	10.46206	8.105797	-8.31719
127	H	7.363033	10.69492	-8.41477
128	H	8.253721	7.8699	-10.2784
129	H	10.69932	7.434338	-9.9559
130	H	8.502048	9.343738	-8.64289
131	C	5.693998	8.07282	-11.6388
132	C	12.08823	5.97526	-7.89908
133	C	8.952614	10.95753	-6.24341
134	C	4.790997	8.65373	-10.7246
135	C	12.51473	4.729358	-8.40431
136	C	9.717481	10.18505	-5.34514
137	H	5.05235	9.586285	-10.2269
138	H	13.10553	4.693384	-9.31788
139	H	10.64328	9.7222	-5.68511
140	C	3.550843	8.042479	-10.4557
141	C	12.17648	3.531256	-7.7465
142	C	9.303706	10.01674	-4.00956
143	H	2.861827	8.503258	-9.75212
144	H	12.50781	2.579187	-8.15273
145	H	9.905024	9.422812	-3.32546
146	C	3.201382	6.844033	-11.1052
147	C	11.4095	3.567977	-6.56718
148	C	8.118346	10.62697	-3.55945
149	H	2.242582	6.372543	-10.9031
150	H	11.14632	2.64469	-6.05736
151	H	7.797274	10.50224	-2.52822
152	C	4.096884	6.262081	-12.0268

153	C	10.9824	4.80851	-6.05107
154	C	7.353734	11.40927	-4.45007
155	H	3.826725	5.340472	-12.537
156	H	10.38833	4.84311	-5.14128
157	H	6.442188	11.88969	-4.10276
158	C	5.334673	6.870412	-12.2921
159	C	11.31763	6.001759	-6.71268
160	C	7.766189	11.57446	-5.78242
161	H	6.021211	6.433512	-13.0144
162	H	10.98206	6.951494	-6.30027
163	H	7.189596	12.19119	-6.46847
164	O	14.63139	11.00951	-12.7904
165	H	15.08846	11.55907	-10.2633
166	C	14.39438	13.48929	-10.9649
167	H	15.07644	13.50718	-11.8237
168	H	13.44739	13.94403	-11.285
169	C	14.9879	14.26513	-9.79844
170	C	16.35665	14.12057	-9.47303
171	C	14.19397	15.12495	-9.00722
172	C	16.91623	14.81494	-8.38612
173	H	16.98607	13.47132	-10.08
174	C	14.74978	15.8229	-7.91595
175	H	13.14238	15.26523	-9.25199
176	C	16.11298	15.66865	-7.60196
177	H	17.97328	14.70003	-8.15681
178	H	14.12569	16.48656	-7.32285
179	H	16.54578	16.21303	-6.76599
180	H	13.40907	12.36672	-8.74054
181	H	12.20702	12.15003	-9.89169
182	H	14.07437	9.503842	-9.66349

Aux9S – Ligand Substitution

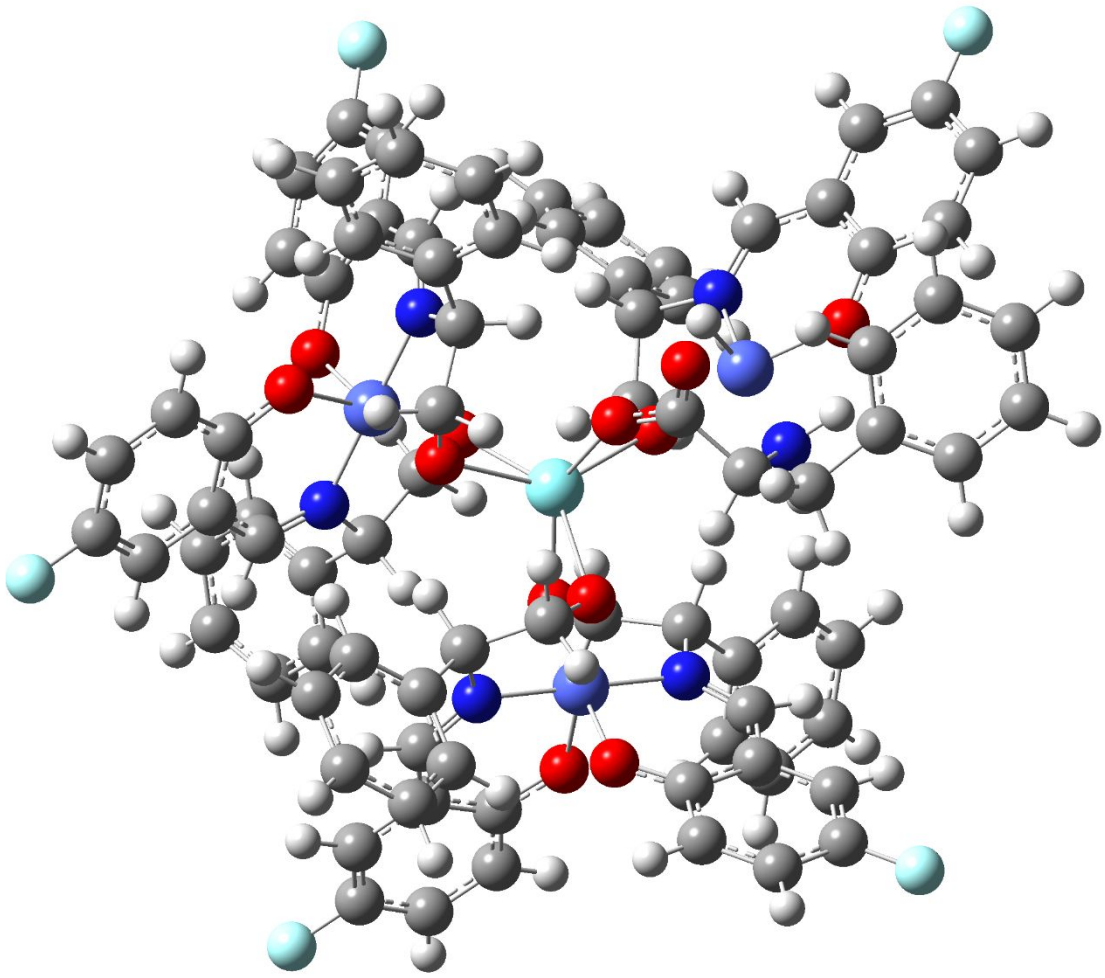

	B3LYP/SDD
**Electronic Energy (EE)**	-5450.05777
**EE + Zero-point Energy**	-5448.65107
**EE + Thermal Energy Correction**	-5448.55484
**EE + Thermal Enthalpy Correction**	-5448.5539
**EE + Thermal Free Energy Correction**	-5448.79735

	B3LYP/SDD			
Tag	Symbol	X	Y	Z
1	Y	10.63008	10.58456	-11.0154
2	Co	9.56301	8.744162	-13.5974
3	Co	13.49121	9.498155	-8.69545
4	Co	8.739005	13.09553	-9.78321
5	F	11.78214	2.198845	-13.3589
6	F	1.896187	13.44893	-10.8627
7	F	5.544551	11.04255	-18.766
8	F	18.7382	5.532023	-6.58614
9	F	11.40598	18.39466	-6.18905
10	O	8.920004	6.959365	-13.7401
11	O	7.079327	13.17795	-8.85193

12	O	10.31608	10.51489	-13.2962
13	O	12.71812	11.0534	-11.4807
14	O	10.3766	12.86368	-10.7821
15	O	9.524796	8.958228	-15.4839
16	O	15.1226	9.565928	-7.81095
17	O	8.887064	14.99127	-9.80007
18	O	9.577339	8.64379	-11.6456
19	O	11.63035	9.4849	-9.22886
20	O	8.761191	11.14091	-9.76566
21	N	11.41677	8.258618	-13.8466
22	N	13.72939	11.50445	-8.65825
23	N	7.948826	13.30126	-11.5355
24	N	7.717458	9.269418	-13.3576
25	N	13.36432	7.618219	-8.56645
26	N	9.537871	12.89724	-8.03001
27	C	9.664119	5.855566	-13.6541
28	C	5.862075	13.24965	-9.39542
29	C	8.984253	4.599892	-13.5574
30	C	4.735841	13.24487	-8.51378
31	H	7.898764	4.615271	-13.5679
32	H	4.931674	13.19725	-7.44699
33	C	9.678196	3.394734	-13.4584
34	C	3.428476	13.30786	-8.9943
35	H	9.156399	2.444904	-13.385
36	H	2.576323	13.30557	-8.32083
37	C	11.08692	3.42049	-13.464
38	C	3.218955	13.38436	-10.3855
39	C	11.80064	4.599871	-13.5786
40	C	4.267603	13.40394	-11.2863
41	H	12.88744	4.576607	-13.6085
42	H	4.066262	13.47858	-12.352
43	C	11.10332	5.839184	-13.6685
44	C	5.608293	13.33141	-10.8083
45	C	11.88628	7.044954	-13.8407
46	C	6.673907	13.39441	-11.7846
47	H	12.95848	6.903072	-14.0051
48	H	6.361806	13.56375	-12.8186
49	C	12.33529	9.426981	-14.0999
50	C	13.68095	12.48366	-9.81933
51	C	8.954608	13.45322	-12.6535
52	H	12.93017	9.575639	-13.1916
53	H	12.66817	12.90292	-9.83905
54	H	9.029988	12.47713	-13.1517
55	C	11.42377	10.67216	-14.238
56	C	13.81278	11.72657	-11.1678
57	C	10.31196	13.72406	-11.957
58	H	11.03142	10.75239	-15.2543

59	H	10.38587	14.77063	-11.6513
60	H	11.99836	11.56878	-13.9842
61	H	11.13937	13.48329	-12.6357
62	C	12.83997	8.71768	-16.5108
63	C	8.337401	15.8469	-13.2855
64	H	11.81516	8.364593	-16.5991
65	H	8.342247	16.0958	-12.2263
66	C	13.72198	8.633612	-17.6019
67	C	8.055336	16.8307	-14.2474
68	H	13.38007	8.20573	-18.5418
69	H	7.851484	17.85187	-13.9337
70	C	15.04601	9.104363	-17.484
71	C	8.034252	16.49895	-15.6184
72	H	15.72636	9.039164	-18.3301
73	H	7.815832	17.262	-16.3616
74	C	15.48216	9.659989	-16.266
75	C	8.291805	15.1749	-16.0194
76	H	16.50001	10.03012	-16.1683
77	H	8.271101	14.90938	-17.0734
78	C	14.60327	9.737593	-15.1686
79	C	8.570751	14.18803	-15.0541
80	H	14.93766	10.17944	-14.2307
81	H	8.765691	13.16422	-15.3687
82	C	13.27828	9.263665	-15.2828
83	C	8.600141	14.51471	-13.6825
84	C	8.532605	9.463081	-16.2175
85	C	15.97314	8.564311	-7.53968
86	C	9.50418	15.75287	-8.89697
87	C	8.754625	9.622544	-17.622
88	C	17.26235	8.884431	-7.0251
89	C	9.577988	17.15987	-9.14879
90	H	9.719786	9.320968	-18.0165
91	H	17.51316	9.93302	-6.90126
92	H	9.120761	17.53212	-10.0602
93	C	7.772684	10.1446	-18.4621
94	C	18.17609	7.87944	-6.71087
95	C	10.20664	18.03054	-8.26132
96	H	7.944704	10.26488	-19.5278
97	H	19.16428	8.111801	-6.32519
98	H	10.26202	19.09729	-8.45778
99	C	6.531068	10.51788	-17.9088
100	C	17.80543	6.532143	-6.90749
101	C	10.77592	17.50745	-7.08198
102	C	6.2557	10.38056	-16.5614
103	C	16.56662	6.167391	-7.40005
104	C	10.72634	16.16097	-6.77762
105	H	5.281511	10.66821	-16.1737

106	H	16.31966	5.117428	-7.53342
107	H	11.16202	15.79782	-5.84986
108	C	7.254437	9.855675	-15.6901
109	C	15.62331	7.185896	-7.72754
110	C	10.0946	15.25713	-7.68255
111	C	6.917463	9.693918	-14.2947
112	C	14.32167	6.794483	-8.2073
113	C	10.03014	13.86497	-7.30739
114	H	5.884917	9.930834	-14.0237
115	H	14.1235	5.722064	-8.25517
116	H	10.4081	13.62478	-6.3091
117	C	7.211446	9.106209	-11.9485
118	C	12.04815	7.094837	-9.08024
119	C	9.473232	11.49718	-7.47728
120	H	7.251875	10.0937	-11.4699
121	H	12.14257	7.028584	-10.1726
122	H	10.4667	11.0468	-7.6156
123	C	8.249823	8.209757	-11.2365
124	C	11.04722	8.220011	-8.77636
125	C	8.491105	10.7318	-8.39937
126	H	8.105855	7.162309	-11.5136
127	H	10.84338	8.265215	-7.69571
128	H	7.459234	10.97794	-8.13682
129	H	8.132518	8.314385	-10.1506
130	H	10.1125	8.02081	-9.3061
131	H	8.640477	9.650954	-8.28778
132	C	5.799651	8.549483	-11.8433
133	C	11.60922	5.744085	-8.53679
134	C	9.082759	11.41208	-6.00995
135	C	4.825185	9.221159	-11.0766
136	C	11.32258	4.691151	-9.43078
137	C	9.873694	10.67868	-5.10163
138	H	5.075294	10.16244	-10.5905
139	H	11.44251	4.843064	-10.5017
140	H	10.79094	10.2025	-5.44705
141	C	3.529574	8.686513	-10.9378
142	C	10.87576	3.444685	-8.95226
143	C	9.490986	10.55455	-3.7519
144	H	2.786525	9.217608	-10.3482
145	H	10.66124	2.644047	-9.65508
146	H	10.11046	9.986793	-3.06185
147	C	3.196639	7.474058	-11.5697
148	C	10.71142	3.238121	-7.56992
149	C	8.310259	11.1705	-3.29751
150	H	2.195622	7.061792	-11.4678
151	H	10.36793	2.27604	-7.1981
152	H	8.012762	11.07971	-2.25573

153	C	4.16527	6.800033	-12.3427
154	C	10.99659	4.284437	-6.66868
155	C	7.517212	11.91077	-4.19916
156	H	3.909856	5.866319	-12.8383
157	H	10.87246	4.129932	-5.59968
158	H	6.606944	12.39236	-3.85015
159	C	5.457937	7.332248	-12.4785
160	C	11.44134	5.527764	-7.14874
161	C	7.898211	12.03063	-5.5456
162	H	6.202384	6.822132	-13.0865
163	H	11.66754	6.323731	-6.44146
164	H	7.296097	12.6102	-6.2421
165	C	14.68776	13.66058	-9.63822
166	H	14.82499	14.11051	-10.6291
167	H	14.20542	14.41789	-9.00457
168	C	16.04715	13.32023	-9.03357
169	C	17.02005	12.58922	-9.75651
170	C	16.3658	13.76012	-7.72785
171	C	18.27553	12.31358	-9.18479
172	H	16.77808	12.24584	-10.7581
173	C	17.62241	13.48453	-7.15372
174	H	15.63638	14.34107	-7.16434
175	C	18.58352	12.75923	-7.88322
176	H	19.0142	11.75646	-9.75673
177	H	17.85115	13.84241	-6.15233
178	H	19.56018	12.55482	-7.44986
179	O	14.8404	11.79329	-11.8873
180	H	13.12425	11.86179	-7.91045
181	H	14.69244	11.4698	-8.29273
182	H	14.02724	9.363329	-10.0064

References

(1) Coles, S. J.; Gale, P. A. Changing and Challenging Times for Service Crystallography. *Chem. Sci.* **2012**, *3* (3), 683–689. https://doi.org/10.1039/c2sc00955b.

(2) Sheldrick, G. M. Crystal Structure Refinement with SHELXL. *Acta Crystallogr. Sect. C Struct. Chem.* **2015**, *71* (1), 3–8. https://doi.org/10.1107/S2053229614024218.

(3) Dolomanov, O. V.; Bourhis, L. J.; Gildea, R. J.; Howard, J. A. K.; Puschmann, H. OLEX2 : A Complete Structure Solution, Refinement and Analysis Program. *J. Appl. Crystallogr.* **2009**, *42* (2), 339–341. https://doi.org/10.1107/S0021889808042726.

(4) Frisch, M. J.; Trucks, G. W.; Schlegel, H. B.; Scuseria, G. E.; Robb, M. A.; Cheeseman, J. R.; Scalmani, G.; Barone, V.; Petersson, G. A.; Nakatsuji, H.; Li, X.; Caricato, M.; Marenich, A. V.; Bloino, J.; Janesko, B. G.; Gomperts, R.; Mennucci, B.; Hratchian, H. P.; Ortiz, J. V.; Izmaylov, A. F.; Sonnenberg, J. L.; Williams, D.; Ding, F.; Lipparini, F.; Egidi, F.; Goings, J.; Peng, B.; Petrone, A.; Henderson, T.; Ranasinghe, D.; Zakrzewski, V. G.; Gao, J.; Rega, N.; Zheng, G.; Liang, W.; Hada, M.; Ehara, M.; Toyota, K.; Fukuda, R.; Hasegawa, J.; Ishida, M.; Nakajima, T.; Honda, Y.; Kitao, O.; Nakai, H.; Vreven, T.; Throssell, K.; Montgomery Jr., J. A.; Peralta, J. E.; Ogliaro, F.; Bearpark, M. J.; Heyd, J. J.; Brothers, E. N.; Kudin, K. N.; Staroverov, V. N.; Keith, T. A.; Kobayashi, R.; Normand, J.; Raghavachari, K.; Rendell, A. P.; Burant, J. C.; Iyengar, S. S.; Tomasi, J.; Cossi, M.; Millam, J. M.; Klene, M.; Adamo, C.; Cammi, R.; Ochterski, J. W.; Martin, R. L.; Morokuma, K.; Farkas, O.; Foresman, J. B.; Fox, D. J. G16_C01. **2016**, Gaussian 16, Revision C.01, Gaussian, Inc., Wallin.

(5) Stephens, P. J.; Devlin, F. J.; Chabalowski, C. F.; Frisch, M. J. Ab Initio Calculation of Vibrational Absorption and Circular Dichroism Spectra Using Density Functional Force Fields. *J. Phys. Chem.* **1994**, *98* (45), 11623–11627. https://doi.org/10.1021/J100096A001/ASSET/J100096A001.FP.PNG_V03.

(6) Zhao, Y.; Truhlar, D. G. The M06 Suite of Density Functionals for Main Group Thermochemistry, Thermochemical Kinetics, Noncovalent Interactions, Excited States, and

Transition Elements: Two New Functionals and Systematic Testing of Four M06-Class Functionals and 12 Other Function. *Theor. Chem. Acc.* **2008**, *120* (1–3), 215–241. https://doi.org/10.1007/s00214-007-0310-x.

(7) Dunning, T. H.; Hay, P. J. Gaussian Basis Sets for Molecular Calculations. In *Methods of Electronic Structure Theory*; Springer, Boston, MA, 1977; pp 1–27. https://doi.org/10.1007/978-1-4757-0887-5_1.

(8) Weigend, F.; Ahlrichs, R. Balanced Basis Sets of Split Valence, Triple Zeta Valence and Quadruple Zeta Valence Quality for H to Rn: Design and Assessment of Accuracy. *Phys. Chem. Chem. Phys.* **2005**, *7* (18), 3297–3305. https://doi.org/10.1039/b508541a.

(9) Weigend, F. Accurate Coulomb-Fitting Basis Sets for H to Rn. *Phys. Chem. Chem. Phys.* **2006**, *8* (9), 1057–1065. https://doi.org/10.1039/b515623h.

(10) Miertuš̃, S.; Tomasi, J. Approximate Evaluations of the Electrostatic Free Energy and Internal Energy Changes in Solution Processes. *Chem. Phys.* **1982**, *65* (2), 239–245. https://doi.org/10.1016/0301-0104(82)85072-6.

(11) Miertuš, S.; Scrocco, E.; Tomasi, J. Electrostatic Interaction of a Solute with a Continuum. A Direct Utilizaion of AB Initio Molecular Potentials for the Prevision of Solvent Effects. *Chem. Phys.* **1981**, *55* (1), 117–129. https://doi.org/10.1016/0301-0104(81)85090-2.

(12) Wang, W.; Xia, X.; Bian, G.; Song, L. A Chiral Sensor for Recognition of Varied Amines Based on 19 F NMR Signals of Newly Designed Rhodium Complexes. *Chem. Commun.* **2019**, *55* (43), 6098–6101. https://doi.org/10.1039/c9cc01942a.
